# Nickel-Catalyzed Ethylene Copolymerization with Vinylalkoxysilanes: A Computational Study

**DOI:** 10.3390/polym16060762

**Published:** 2024-03-10

**Authors:** Zhihui Song, Rong Gao, Changjiang Wu, Qingqiang Gou, Gang Zheng, Junjie Liu, Shifang Yang, Huasheng Feng

**Affiliations:** 1 Department of Polyethylene, SINOPEC (Beijing) Research Institute of Chemical Industry Co., Ltd., Beijing 100013, China; gaor.bjhy@sinopec.com (R.G.); gouqq.bjhy@sinopec.com (Q.G.); zhenggang.bjhy@sinopec.com (G.Z.); 2 SINOPEC (Beijing) Research Institute of Chemical Industry Co., Ltd., Beijing 100013, China; wuchangjiang.bjhy@sinopec.com; 3 Department of Ethylene, SINOPEC (Beijing) Research Institute of Chemical Industry Co., Ltd., Beijing 100013, China; liujj.bjhy@sinopec.com (J.L.); yangshf.bjhy@sinopec.com (S.Y.); 4 Department of Catalytic Science, SINOPEC (Beijing) Research Institute of Chemical Industry Co., Ltd., Beijing 100013, China; fenghs.bjhy@sinopec.com

**Keywords:** ethylene polymerization, density functional theory (DFT), Brookhart-type catalyst

## Abstract

Since the discovery of α-diimine catalysts in 1995, an extensive series of Brookhart-type complexes have shown their excellence in catalyzing ethylene polymerizations with remarkable activity and a high molecular weight. However, although this class of palladium complexes has proven proficiency in catalyzing ethylene copolymerization with various polar monomers, the α-diimine nickel catalysts have generally exhibited a much worse performance in these copolymerizations compared to their palladium counterparts. Recently, Brookhart et al. reported a notable exception, demonstrating that α-diimine nickel catalysts could catalyze the ethylene copolymerization with some vinylalkoxysilanes effectively, producing functionalized polyethylene incorporating trialkoxysilane (-Si(OR)_3_) groups. This breakthrough is significant since Pd-catalyzed copolymerizations are commercially less usable due to the high cost of palladium. Thus, the utilization of Ni, given its abundance in raw materials and cost-effectiveness, is a landmark in ethylene/polar vinyl monomer copolymerization. Inspired by these findings, we used density functional theory (DFT) calculations to investigate the mechanistic study of ethylene copolymerization with vinyltrimethoxysilane (VTMoS) catalyzed by Brookhart-type nickel catalysts, aiming to elucidate the molecular-level understanding of this unique reaction. Initially, the nickel complexes and cationic active species were optimized through DFT calculations. Subsequently, we explored the mechanisms including the chain initiation, chain propagation, and chain termination of ethylene homopolymerization and copolymerization catalyzed by Brookhart-type complexes. Finally, we conducted an energetic analysis of both the in-chain and chain-end of silane enchainment. It was found that chain initiation is the dominant step in the ethylene homopolymerization catalyzed by the α-diimine Ni complex. The 1,2- and 2,1-insertion of vinylalkoxysilane exhibit similar barriers, explaining the fact that both five-membered and four-membered chelates were identified experimentally. After the VTMoS insertion, the barriers of ethylene reinsertion become higher, indicating that this step is the rate-determining step, which could be attributed to the steric hindrance between the incoming ethylene and the bulky silane substrate. We have also reported the energetic analysis of the distribution of polar substrates. The dominant pathway of chain-end -Si(OR)_3_ incorporation is suggested as chain-walking → ring-opening → ethylene insertion, and the preference of chain-end -Si(OR)_3_ incorporation is primarily attributed to the steric repulsion between the pre-inserted silane group and the incoming ethylene molecule, reducing the likelihood of in-chain incorporation.

## 1. Introduction

**Figure 1:**
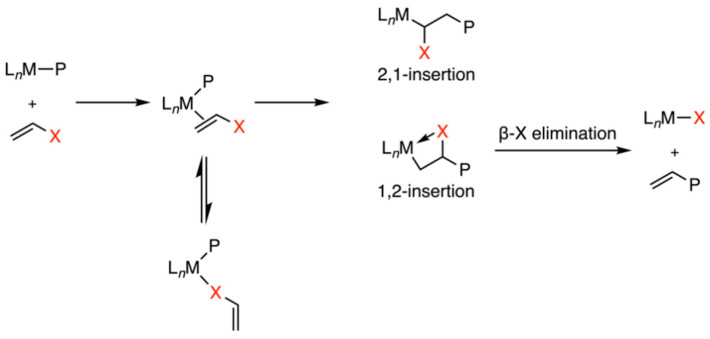
Several methods for retarding transition-metal-catalyzed ethylene copolymerization [8,9].

Polyolefins stand as a predominant class of plastics, constituting over half of the global plastic production at 360 million tons, playing an important role in modern society [1]. The incorporation of polar functional groups is an effective method to enhance surface properties, thereby widening their range of potential applications [2,3,4,5,6]. Transition-metal-catalyzed copolymerization emerges as a direct and cost-effective method for producing functionalized polyolefins under mild conditions [7]. However, Ziegler–Natta catalysts and metallocene-based catalysts encounter some challenges in ethylene copolymerization related to the poisoning of the metal center (Figure 1) [8,9]. These challenges could come from the deactivation of the Lewis acidic metal center, particularly due to the coordination with Lewis basic polar groups. Furthermore, 2,1-insertion may lead to the formation of stable metal–alkyl chelates, followed by β-X elimination, inhibiting the further insertion. Due to the weaker Lewis acidity and lower oxophilicity, late transition metal catalysts, such as Pd- and Ni-based catalysts, exhibit better tolerance of functional/polar groups, thus having been employed in ethylene copolymerizations for synthesizing polar polymers.

**Figure 2:**
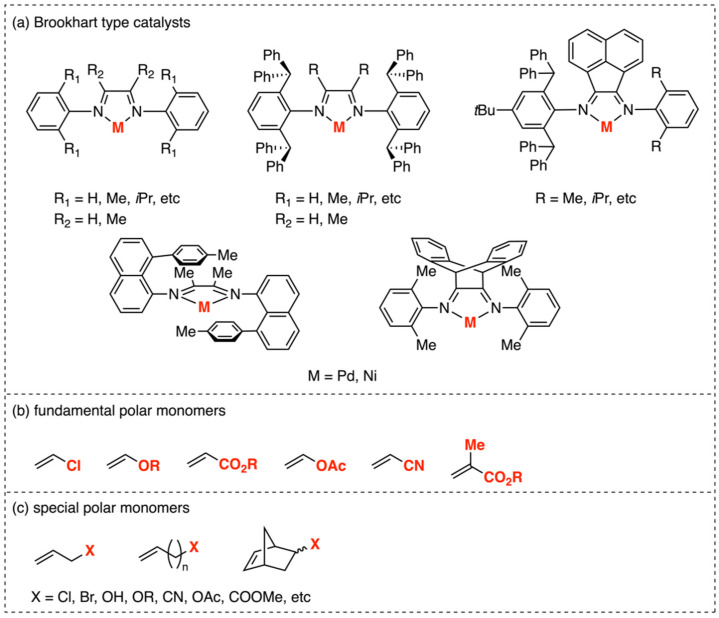
(**a**) Structures of Brookhart’s Pd and Ni α-diimine catalysts. (**b**) Fundamental polar monomers used in copolymerization reactions. (**c**) Special polar monomers used in copolymerization reactions.

Since the discovery of α-diimine catalysts in 1995 for the production of branched polymers [10], an extensive series of Brookhart-type complexes has been reported, demonstrating the excellence in catalyzing ethylene polymerizations with a high activity and high molecular weight (Figure 2a) [6,11,12,13,14,15,16,17,18,19]. However, although this class of palladium complexes has demonstrated proficiency in catalyzing ethylene copolymerization with fundamental polar monomers (Figure 2b) [16] (with a polar group directly attached to the vinyl double bond) such as acrylates [6,11,12,13], vinyl ketones [6], silyl vinyl ethers [14,15], vinyl alkoxysilanes [20], and some other special polar monomers (Figure 2c) (with a spacer between the polar group and the double bond) [17,18,19,21,22,23,24,25], the α-diimine nickel catalysts generally showed much worse properties in these copolymerizations compared with their palladium counterparts [26,27], mainly attributed to the higher oxophilicity of nickel compared to palladium (Figure 2b).

**Figure 3:**
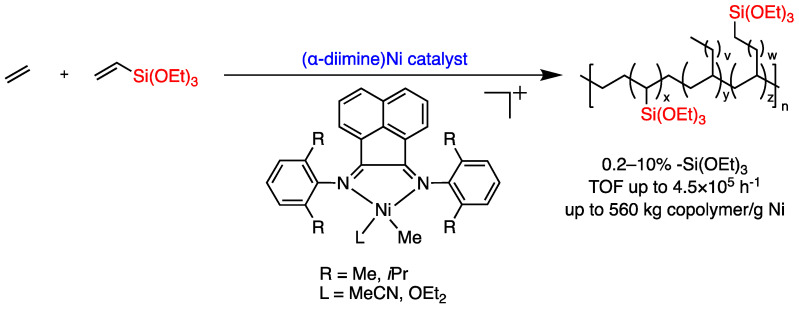
Reactions of ethylene copolymerization with vinyltrialkoxysilanes catalyzed by (α-diimine) nickel catalyst.

Recently, Brookhart et al. reported that α-diimine nickel catalysts could catalyze the ethylene copolymerization with some vinylalkoxysilanes (Figure 3) [28], producing functionalized polyethylene incorporating trialkoxysilane (-Si(OR)_3_) groups, which could be cross-linked to form PEX-b, a tough material that is widely used for power cable insulation and hot water piping systems [29,30]. Notably, this breakthrough is significant since Pd-catalyzed copolymerizations are commercially less usable due to the high cost of palladium. Therefore, the utilization of Ni, given its abundance in raw materials and cost-effectiveness, is a landmark in ethylene/polar vinyl monomer copolymerization.

With the rapid evolution of olefin polymerization, the mechanistic study of Brookhart-type catalytic copolymerization systems has become indispensable [9]. The inherent complexity of α-diimine catalytic systems necessitates a thorough exploration of the detailed factors that control catalytic activity, molecular weight, comonomer incorporation ratios, and branch content in these transformations. To advance the late transition-metal-catalyzed olefin copolymerization, many factors of catalysts were considered such as electronic inhibition, steric inhibition, metal center, etc. Despite significant development [9], numerous aspects of late transition-metal-catalyzed olefin copolymerization need further investigation. Catalyst-related factors such as electronic inhibition, steric hindrance, and metal center configuration, have been carefully studied to enhance the efficacy of these processes. However, the fundamental mechanisms driving such catalytic activity and the intricate reaction pathways involved have remained elusive. Clarifying these original aspects is essential for the future advancement and refinement of copolymerization systems, offering insights that could catalyze these transforms in polymerization.

In this manuscript, to elucidate the molecular-level understanding of ethylene polymerization catalyzed by Ni-based α-diimine complexes and to investigate the factors contributing to diverse performances associated with different polar monomers, density functional theory (DFT) calculations were performed to disclose this polymerization mechanism in detail. On the basis of these theoretical studies, we clarified the major pathways and rate-determining steps in this reaction. Furthermore, DFT calculations enabled the identification of active species and transition state structures, enhancing our comprehension of this reaction. The energetic analysis, coupled with an exploration of influencing factors, offers valuable insights for interpreting ethylene copolymerizations. These mechanistic studies could shed light on the future design of nickel-based α-diimine complexes used for ethylene copolymerization with fundamental polar monomers.

## 2. Computational Methods

All density functional theory (DFT) calculations were performed with the Gaussian 16 program [31]. Geometry optimizations employed the spin-unrestricted dispersion-corrected method (UB3LYP [32,33]-D3 [34,35,36,37]), with the 6-31G(d) basis set for nonmetal atoms (C, N, H, Si, O) and the LANL2DZ basis set along with the associated pseudopotential for metal atoms (Ni), denoted as BSI [38,39,40]. Frequency calculations were also conducted at the same level of theory to obtain vibrational frequencies to determine the identity of stationary points as intermediates (no imaginary frequencies) or transition states (only one imaginary frequency), as well as obtaining the thermal corrections to enthalpy (H_correction_) and free energy (G_correction_) at the temperature of 298 K. Single-point energies based on BSI geometries were refined using a higher-level method denoted as BSII. For BSII, the 6-311G(d,p) basis set was applied to nonmetal atoms (C, N, H, Si, O), and the SDD basis set with its associated pseudopotential was used for metal atoms (Ni) [40]. To explore the effect of toluene solvent on the catalytic system, single point energies were performed in implicit toluene solvent through the SMD model [41], labeled as UB3LYP-D3/BSII(SMD)//B3LYP-D3/BSI. Energy profiles were constructed at the UB3LYP-D3/BSII(SMD)//B3LYP-D3/BSI level, including Gibbs free energy corrections taken from frequency calculations in the gas phase. All optimized geometrical figures were generated with CYLview [42], with energies reported in kcal/mol.

## 3. Results and Discussion

### 3.1. Nickel Complexes and Cationic Active Species

**Figure 4:**
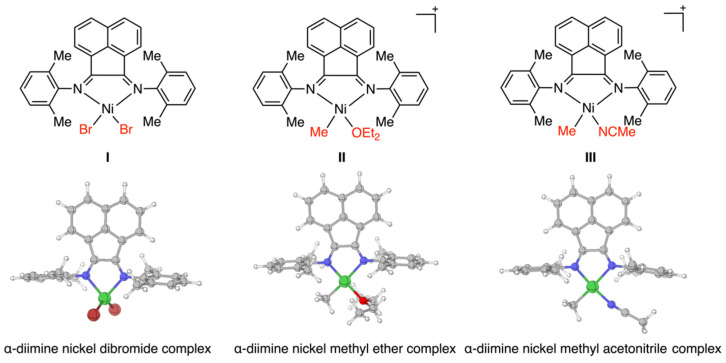
Optimized structures of complexes **I**, **II**, and **III**.

As reported in the previous study [28], the ethylene polymerization was catalyzed by α-diimine Ni catalysts. The α-diimine nickel dibromide complex **I**, the cationic and α-diimine nickel methyl ether complex **II**, and the nickel methyl acetonitrile complex **III** were regarded as the initial Ni complexes and active cationic active species, and thus were optimized by DFT calculations (Figure 4). Optimized structures exhibited distinct geometrical characteristics, where the Ni(II)Br_2_ complex **I** showed tetrahedral conformation while the Ni(II)(OEt)_2_ cationic complex **II** and the Ni(II)(MeCN)^+^ cationic complex **III** exhibited square planar conformations. Based on these optimized structures, ethylene polymerizations were investigated computationally in detail as follows.

### 3.2. Chain Initiation and Chain Propagation (Ethylene Insertion)

**Figure 5:**
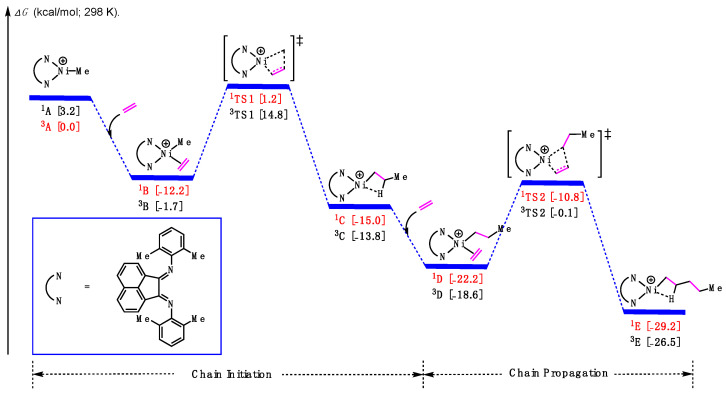
Calculated relative Gibbs free energies for the insertion of ethylene.

Based on previous studies [43,44], DFT calculations were conducted to explore the ethylene polymerization process, including both chain initiation and chain propagation. For all proposed intermediates and transition states, both singlet and triplet spin states were considered, and the lower-energy structures were identified and colored. As shown in Figure 5 (and Figure S1 in Supplementary Materials), starting with the cationic methylated α-diimine Ni complex **^3^A**, one molecule of ethylene coordinated to the Ni center, forming the π-complex **^1^B** and releasing an energy of 2.0 kcal/mol. Subsequently, the π-complex could undergo the first ethylene insertion via transition states **^1^TS1** with a barrier of 14.4 kcal/mol, leading to the chain initiation product **^3^C**. In turn, the Ni-alkyl species will then be poised to coordinate the second ethylene molecule and undergo the second ethylene insertion via transition states **^1^TS2** to form the product **^3^E**. Notably, the barrier for the chain propagation step was found to be 13.1 kcal/mol, lower than that of the first step, which suggests that the chain initiation is the rate-determining step in the ethylene homopolymerization catalyzed by this cationic α-diimine Ni complex.

### 3.3. Chain Propagation (Vinylalkoxysilane Insertion) and Chain Termination 

**Figure 6:**
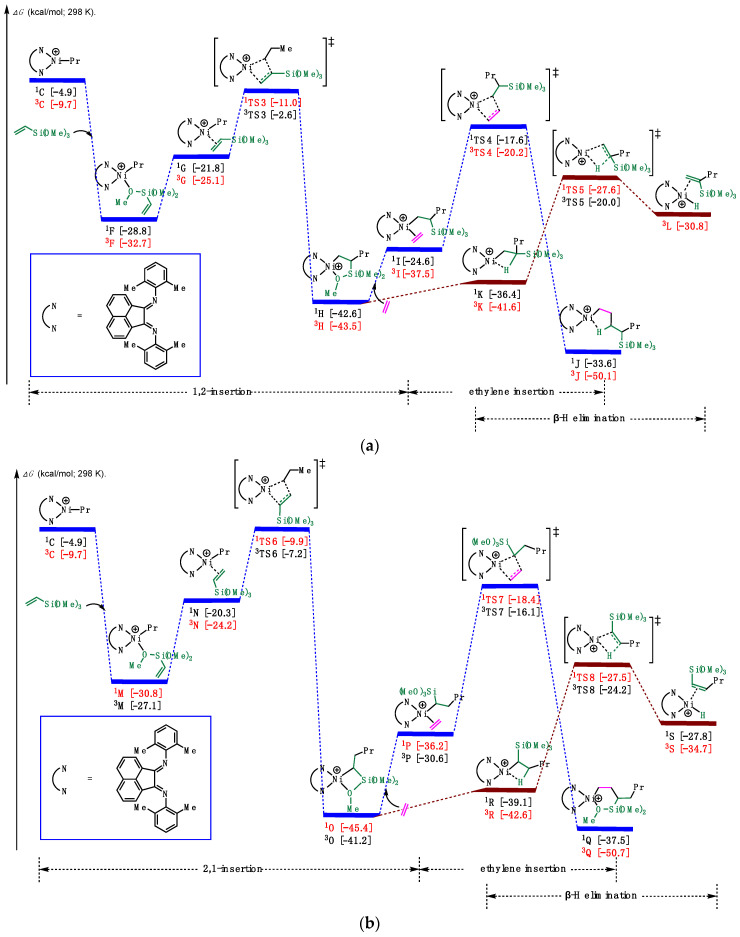
Calculated relative Gibbs free energies for (**a**) 1,2-insertion of vinyltrimethoxysilane (**b**) 2,1-insertion of vinyltrimethoxysilane, ethylene reinsertion, and β-H elimination.

After the chain initiation step to form the Ni-alkyl species, we proceeded to explore the insertion of vinylalkoxysilane (Figure 6). As shown in Figure 6a (and Figure S2a in Supplementary Materials), based on the relative stability of π-complexes and O-complexes [45], the Ni-alkyl complex **^3^C** coordinated with vinyltrimethoxysilane (VTMoS), releasing an energy of 23.0 kcal/mol to generate the O-complex **^3^F**, which could equilibrate to the π-complex **^3^G**. Then, **^3^G** could undergo 1,2-insertion via **^1^TS3** with an overall barrier of 21.7 kcal/mol, leading to the formation of a Ni(II) five-membered chelate complex **^3^H** (10.8 kcal/mol downhill in energy). Notably, the five-membered chelate complex could be isolated and identified successfully by the NMR spectrum [28]. In turn, excess ethylene induced the chelate opening of **^3^H**, and then another molecule of ethylene inserted into the Ni-alkyl species with a barrier of 23.3 kcal/mol, resulting in the desired product **^3^J**. Considering prior computational studies [43,45,46,47], we also investigated the commonly proposed chain termination pathway that β-H elimination. However, this pathway was found to be much lower in energy (~16 kcal/mol) compared to the 1,2-insertion. Despite this computational result, experimental results [28] supported the predominance of 1,2-insertion over β-H elimination. We attribute this observation to the excess ethylene favoring the chain propagation pathway over the chain termination pathway. And the small energetic difference among **^3^I**, **^3^K**, and **^3^H** allowed for their mutual transfer, thereby giving the possibility of proceeding the chain propagation step.

To obtain a comprehensive understanding of this reaction, we also considered the 2,1-insertion pathway (Figure 6b and Figure S2b in Supplementary Materials). Similar to the 1,2-insertion, starting from the coordination of **^3^C**, the Ni(II) species **^1^N** could undergo 2,1-insertion with VTMoS via **^1^TS6** (the barrier was 20.9 kcal/mol), leading directly to the four-membered chelate complex **^1^O**, accompanied by the release of 14.6 kcal/mol in energy. The four-membered chelate complex **^1^O** could also be detectable by the NMR spectrum [28], supporting the rigidity of the computational results. Then, excess ethylene could foster the ring-opening of **^3^H**, and then another molecule of ethylene inserted into the Ni-C bond with a barrier of 27.0 kcal/mol, resulting in the six-membered chelate product **^3^Q**. Additionally, from **^1^O**, we located the transition state for β-H elimination, denoted as ^1^**TS8**, leading to the formation of the Ni(II)-H species **^3^S**. Since the pathways and barriers were similar for both 1,2- and 2,1-insertion pathways, and the chelates could be found in experiments, it is obvious that both 1,2- and 2,1-insertion merged in this reaction, which is special and different from other catalysts.

Given the similarity in pathways and barriers for both 1,2- and 2,1-insertion, combined with the experimental identification of the five-membered and four-membered chelates, it is evident that both 1,2- and 2,1-insertion pathways emerged in this reaction, which is a distinctive feature, setting it apart from reactions involving other catalysts [48,49]. Notably, the barrier of 1,2-insertion was 21.7 kcal/mol, which is higher than that of 2,1-insertion, suggesting that 1,2-insertion is preferred, consistent with the preference of the four-membered chelate experimentally [28]. Additionally, the four-membered and five-membered chelate complexes, **^1^H** and **^1^O**, were favored compared with not only Ni-ethylene complexes **^3^I** and **^1^P** but also O-complexes **^3^F** and **^1^M**.

Notably, according to a pertinent article, the experimentally measured barrier for polar monomer insertion from the π-complex to the insertion product was reported as 15.5 kcal/mol [50], similar to the barrier calculated in our manuscript (14.1 and 14.3 kcal/mol). Despite the difference in the polar monomer studied in the reference (vinyl acetate), we employed the same catalysts in our research, so the minor difference observed is reasonable. This consistency verified the validity and reliability of our computational methodology.

### 3.4. Energetic Analysis of In-Chain and Chain-End Silane Enchainment

**Figure 7:**
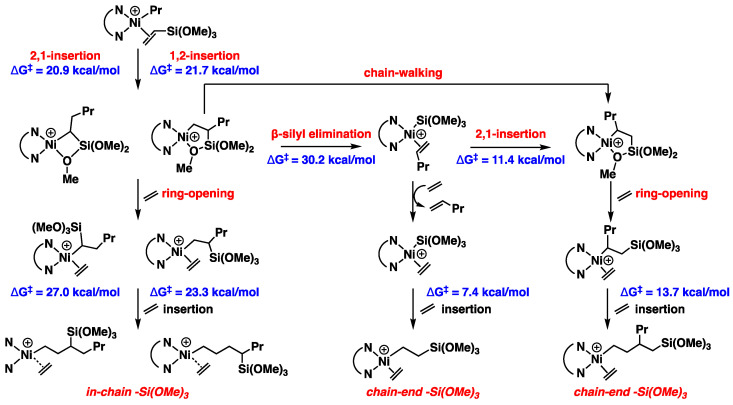
Calculated relative Gibbs free energies for the silane enchainment.

As for the observation indicating copolymers with the incorporation of -Si(OR)_3_ groups at both in-chain and chain-end positions, the ratios of in-chain to chain-end incorporation ranged from 1.0:1.1 to 1.0:2.8, varying based on temperature and ethylene pressure [28]. We conducted an energetic analysis of both the in-chain and chain-end of silane enchainment (Figure 7 and Figure S4 in Supplementary Materials). We proposed that the silane products emerged through three pathways, starting with the VTMoS insertion products. 

After the VTMoS insertion, chelate complexes become the major resting states but are in rapid equilibrium with (α-diimine)Ni(R)(C2H4)^+^ complexes. Starting from the four-membered and five-membered chelate complexes, they undergo ring-opening via ethylene coordination followed by chain propagation to form the in-chain -Si(OR)_3_ structures with barriers of 27.0 and 23.3 kcal/mol. Additionally, β-silyl elimination from the five-membered chelate complex generates the Ni-Si(OR)_3_ species (the barrier was 30.2 kcal/mol), followed by butene displacement and ethylene insertion, leading to the formation of the chain-end copolymer. Moreover, after facial chain-walking from the five-membered chelate complex or 2,1-insertion of the Ni-Si(OR)_3_ species, the “new” five-membered chelate complex underwent ring-opening, followed by ethylene insertion to produce the chain-end product with a lower barrier of 13.7 kcal/mol, compared to ethylene insertion in in-chain silane copolymer formation. Notably, the pathway of chain-walking → ring-opening → ethylene insertion is considered the dominant one for chain-end -Si(OR)_3_ incorporation [28], while β-silyl elimination → olefin displacement → ethylene insertion is a minor pathway [28] due to the higher barrier (30.2 kcal/mol) of β-silyl elimination though this is the dominant pathway in the Pd system [20]. The higher barriers (27.0 and 23.3 kcal/mol) for in-chain product formation are consistent with lower ratios of in-chain silane groups observed in experimental results. We assumed the steric repulsion between the pre-inserted silane group and the incoming ethylene molecule contributes to the higher barriers for in-chain product formation, making the incorporation of the in-chain less likely. These calculations align with the difference caused by temperature and concentration [28]. Higher ethylene pressure enhances the ethylene trapping and subsequent insertion relative to the chain-walking and β-silyl elimination, increasing the ratio of in-chain -Si(OR)_3_ incorporation. Higher temperature led to the increase in the ratio of chain-end -Si(OR)_3_ incorporation, likely because the higher temperature caused the β-silyl elimination more facial, which accelerated the minor chain-end -Si(OR)_3_ incorporation pathway. 

## 4. Conclusions

In this manuscript, DFT calculations were performed to explore the mechanistic study of ethylene copolymerization with vinyltrimethoxysilane (VTMoS) catalyzed by α-diimine nickel catalysts. It was found that chain initiation is the dominant step in the ethylene homopolymerization catalyzed by the α-diimine Ni complex. After the insertion of the first molecule of ethylene, the subsequent insertion of vinylalkoxysilane in both 1,2-mode and 2,1-mode exhibits similar barriers, being consistent with the experimental results that five-membered and four-membered chelates were all isolated. After the VTMoS insertion, the barriers of ethylene reinsertion became higher (23.3 and 27.0 kcal/mol), which indicates that this step is the rate-determining step, similar to the previous studies [43,47]. This phenomenon could be attributed to the steric hindrance between the incoming ethylene and the bulky silane substrate. 

Furthermore, we considered the distribution of polar substrates. Experimental ratios of in-chain to chain-end incorporation of -Si(OR)_3_ groups ranging from 1.0:1.1 to 1.0:2.8 are aligned with the trend of calculated results: higher computational barriers for in-chain product formation result in lower experimental ratios of in-chain incorporation. Additionally, as ethylene pressure increases, the 1,2-insertion product, a five-membered chelate, shows a preference for coordinating with ethylene, meanwhile decreasing the chain-walking to form the chain-end product. Subsequently, it undergoes ethylene reinsertion to form the in-chain product, thereby increasing the ratio of in-chain products. Moreover, with increasing temperature, in-chain incorporation with higher barriers becomes more feasible, leading to a higher ratio. The experimental results mentioned above provide solid evidence for our computational findings.

The dominant pathway for chain-end -Si(OR)_3_ incorporation is suggested as chain-walking → ring-opening → ethylene insertion, with a lower barrier compared to other pathways. The preference of the chain-end -Si(OR)_3_ incorporation is primarily attributed to the steric repulsion between the pre-inserted silane group and the incoming ethylene molecule, reducing the likelihood of in-chain incorporation.

In summary, our DFT calculations provide a molecular understanding of Ni-catalyzed ethylene copolymerization, offering insights into the copolymerization details and inspiring the design of catalytic systems and modification of transition-metal catalysts. Future investigations will focus on the comparison of more transition-metal systems and the behavior of different polar monomers. In the forthcoming research, we intend to conduct a comparative analysis between this current nickel-based system and a palladium-based counterpart. Our objective is to deepen our understanding of catalytic mechanisms and optimize reactions by investigating the impact of diverse substrates. Through this investigation, we aim to build the internal relationship between catalyst structures and reactivity, thereby improving synthetic methodologies.

Furthermore, we plan to explore the behavior of the polar monomer utilized in this manuscript, vinyltrimethoxysilane, in comparison with other polar monomers such as methyl methacrylate and methyl acrylate. This comparative study is motivated by the observed differences in reactivity among these monomers. By elucidating the underlying factors contributing to these variations, we look forward to shedding light on the mechanistic intricacies governing polymerization processes, advancing the understanding of polymer chemistry and facilitating the development of synthetic catalytic systems.

## Data Availability

Data are contained within the article and Supplementary Materials.

## Supplementary Materials

The following supporting information can be downloaded at: https://www.mdpi.com/article/10.3390/polym16060762/s1, Figure S1: Calculated relative Gibbs free energies of optimization for the insertion of ethylene. Free energies (kcal/mol) were computed at the B3LYP-D3/BSI level of theory with the 6-31G(d) basis set for nonmetal atoms (C, N, H, Si, O) and the LANL2DZ basis set along with the associated pseudopotential for metal atoms (Ni), denoted as BSI.; Figure S2: Calculated relative Gibbs free energies of optimization for the (a) 1,2-insertion of vinyltrimethoxysilane (b) 2,1-insertion of vinyltrimethoxysilane, ethylene reinsertion, and β-H elimination. Free energies (kcal/mol) were computed at the B3LYP-D3/BSI level of theory with the 6-31G(d) basis set for nonmetal atoms (C, N, H, Si, O) and the LANL2DZ basis set along with the associated pseudopotential for metal atoms (Ni), denoted as BSI.; Figure S3: Calculated relative Gibbs free energies of optimization for the silane enchainment. Free energies (kcal/mol) were computed at the B3LYP-D3/BSI level of theory with the 6-31G(d) basis set for nonmetal atoms (C, N, H, Si, O) and the LANL2DZ basis set along with the associated pseudopotential for metal atoms (Ni), denoted as BSI; Figure S4: Calculated relative Gibbs free energies for the silane enchainment. Free energies (kcal/mol) were computed at the UB3LYP-D3/BSII(SMD)//B3LYP-D3/BSI level of theory with the 6-311G(d,p) basis set for nonmetal atoms (C, N, H, Si, O) and the SDD basis set along with the associated pseudopotential for metal atoms (Ni), denoted as BSII. And the Supplementary Materials include the optimized Cartesian coordinates (XYZ) with the relative energies.
