# Supplementary material for: Nickel-Catalyzed Ethylene Copolymerization with Vinylalkoxysilanes: A Computational Study
Source: Polymers (Basel). 2024 Mar 10;16(6):762. doi: 10.3390/polym16060762 (PMC10974285; doi:10.3390/polym16060762)
Supplement: Supplementary file 1 [file polymers-16-00762-s001.zip › polymers-2877706-supplementary/Supporting Information.pdf]

Supporting Information

Nickel-Catalyzed Ethylene Copolymerization with Vinylalkoxysilanes: A Computational Study

Zhihui Song [1,*], Rong Gao [1], Changjiang Wu [2], Qingqiang Gou [1], Gang Zheng [1], Junjie Liu [3], Shifang Yang [3], and Huasheng Feng [4],

[1] Department of Polyethylene, SINOPEC (Beijing) Research Institute of Chemical Industry Co., Ltd., Beijing 100013, China; songzhh.bjhy@sinopec.com (Z.S.); gaor.bjhy@sinopec.com (R.G.); gouqq.bjhy@sinopec.com (Q.G.); zhenggang.bjhy@sinopec.com (G.Z.)
[2] SINOPEC (Beijing) Research Institute of Chemical Industry Co., Ltd., Beijing 100013, China; wuchangjiang.bjhy@sinopec.com (C.W.)
[3] Department of Ethylene, SINOPEC (Beijing) Research Institute of Chemical Industry Co., Ltd., Beijing 100013, China; liujj.bjhy@sinopec.com (J.L.); yangshf.bjhy@sinopec.com (S.Y.)
[4] Department of Catalytic Science, SINOPEC (Beijing) Research Institute of Chemical Industry Co., Ltd., Beijing 100013, China; fenghs.bjhy@sinopec.com (H.F.)
* Correspondence: songzhh.bjhy@sinopec.com (Z.S.)

1. Computational Details

All density functional theory (DFT) calculations were performed with the Gaussian 16 program. Geometry optimizations employed the spin-unrestricted dispersion-corrected method (UB3LYP-D3), with the 6-31G(d) basis set for nonmetal atoms (C, N, H, Si, O) and the LANL2DZ basis set along with the associated pseudopotential for metal atoms (Ni), denoted as BSI. Frequency calculations were also conducted at the same level of theory to obtain vibrational frequencies to determine the identity of stationary points as intermediates (no imaginary frequencies) or transition states (only one imaginary frequency), as well as obtaining the thermal corrections to enthalpy (Hcorrection) and free energy (Gcorrection) at the temperature of 298K. Single-point energies based on BSI geometries were refined using a higher-level method denoted as BSII. For BSII, the 6-311G(d,p) basis set was applied to nonmetal atoms (C, N, H, Si, O), and the SDD basis set with its associated pseudopotential was used for metal atoms (Ni). To explore the effect of toluene solvent on the catalytic system, single point energies were performed in implicit toluene solvent through the SMD model, labeled as UB3LYP-D3/BSII(SMD)//B3LYP-D3/BSI. Energy profiles were constructed at the UB3LYP-D3/BSII(SMD)//B3LYP-D3/BSI level, including Gibbs free energy corrections taken from frequency calculations in the gas phase. All optimized geometrical figures were generated with CYLview, with distances presented in Å, and energies reported in kcal/mol.

2. Energetics with Different Methods from Optimizations

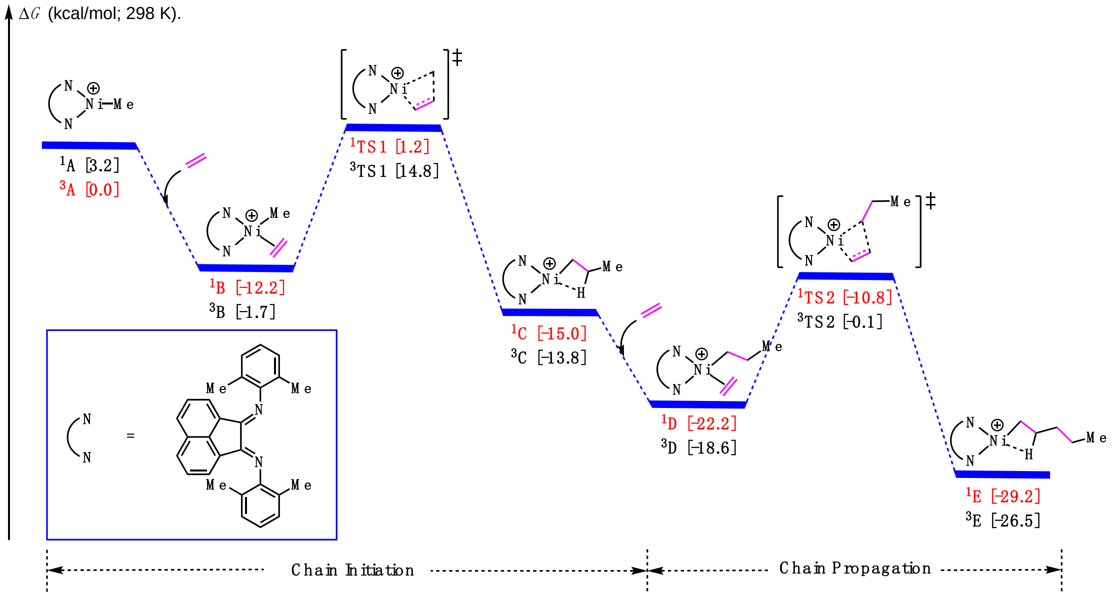

Figure S1. Calculated relative Gibbs free energies of optimization for the insertion of ethylene. Free energies (kcal/mol) were computed at the B3LYP-D3/BSI level of theory with the 6-31G(d) basis set for nonmetal atoms (C, N, H, Si, O) and the LANL2DZ basis set along with the associated pseudopotential for metal atoms (Ni), denoted as BSI.

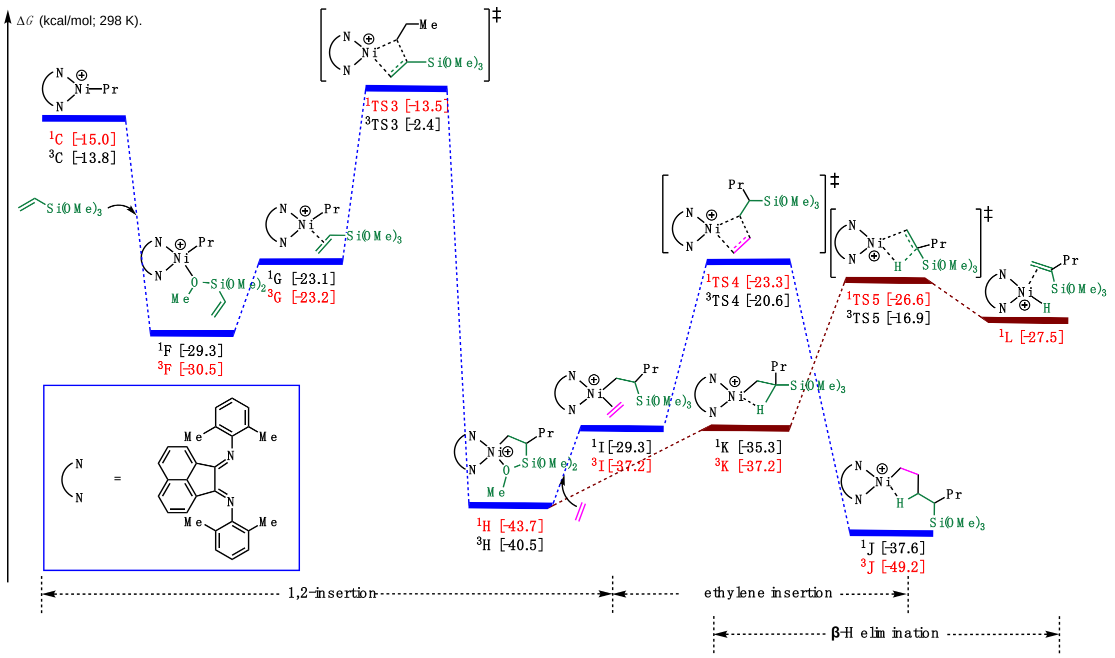

(a)

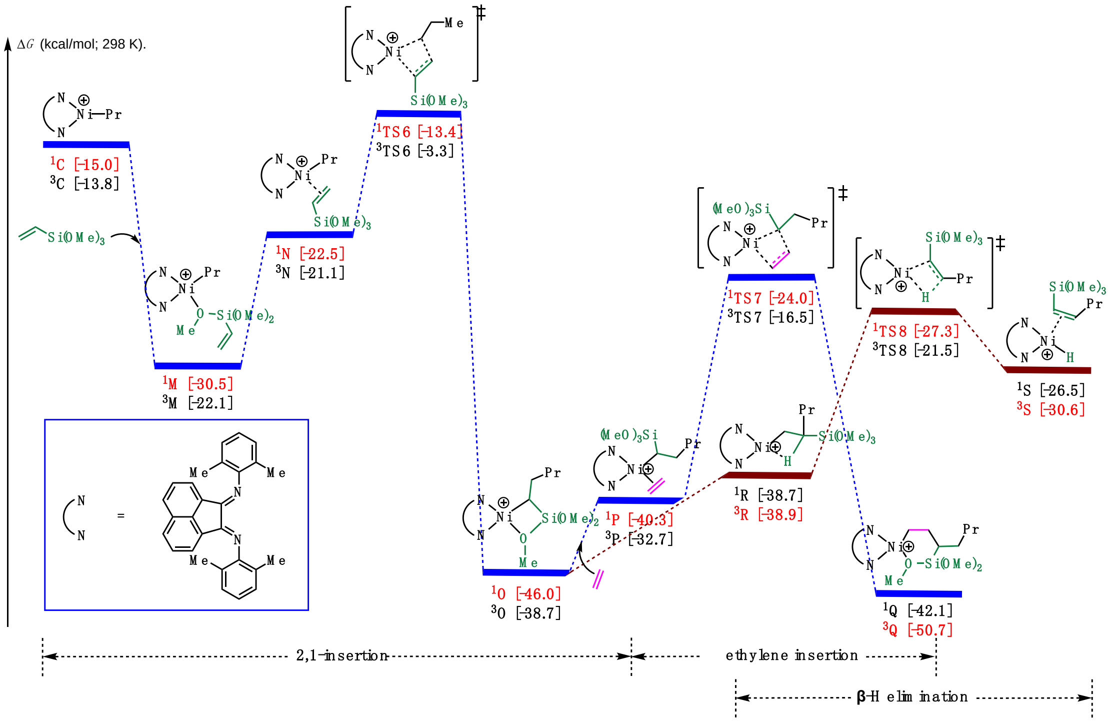

(b)

Figure S2. Calculated relative Gibbs free energies of optimization for the (a) 1,2-insertion of vinyltrimethoxysilane (b) 2,1-insertion of vinyltrimethoxysilane, ethylene reinsertion, and β-H elimination. Free energies (kcal/mol) were computed at the B3LYP-D3/BSI level of theory with the 6-31G(d) basis set for nonmetal atoms (C, N, H, Si, O) and the LANL2DZ basis set along with the associated pseudopotential for metal atoms (Ni), denoted as BSI.

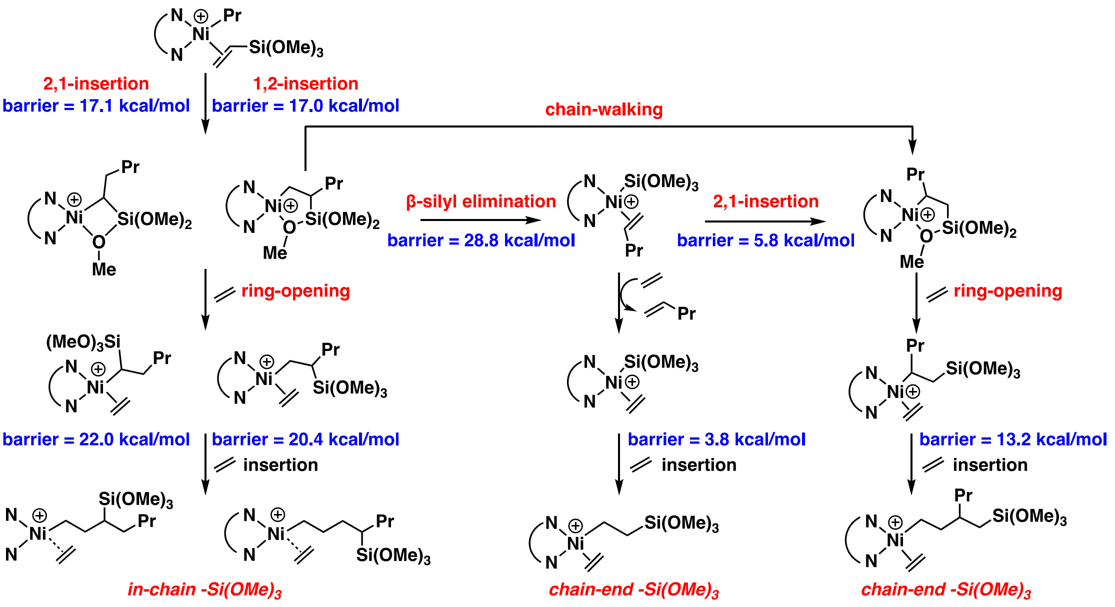

Figure S3. Calculated relative Gibbs free energies of optimization for the silane enchainment. Free energies (kcal/mol) were computed at the B3LYP-D3/BSI level of theory with the 6-31G(d) basis set for nonmetal atoms (C, N, H, Si, O) and the LANL2DZ basis set along with the associated pseudopotential for metal atoms (Ni), denoted as BSI.

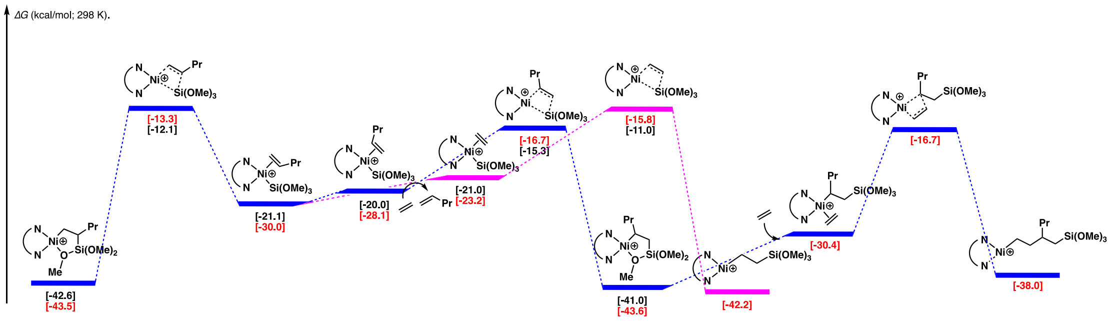

Figure S4. Calculated relative Gibbs free energies for the silane enchainment. Free energies (kcal/mol) were computed at the UB3LYP-D3/BSII(SMD)//B3LYP-D3/BSI level of theory with the 6-311G(d,p) basis set for nonmetal atoms (C, N, H, Si, O) and the SDD basis set along with the associated pseudopotential for metal atoms (Ni), denoted as BSII.

3. Coordinates and Energies of Optimized Structures in Computations

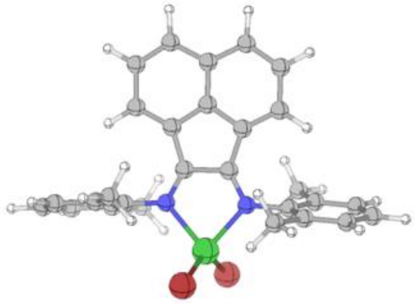

B3LYP-D3/BSI

Zero-point correction= 0.447859 (Hartree/Particle)

Thermal correction to Energy= 0.479246

Thermal correction to Enthalpy= 0.480191

Thermal correction to Gibbs Free Energy= 0.379854

Sum of electronic and zero-point Energies= -6502.838089

Sum of electronic and thermal Energies= -6502.806702

Sum of electronic and thermal Enthalpies= -6502.805758

Sum of electronic and thermal Free Energies= -6502.906095

Ni	-2.01261000	3.85067700	-1.36050700
Br	-1.90191100	4.01876200	-3.72352600
Br	-2.17416700	5.53661000	0.29933300
N	-0.61013900	2.41992600	-0.77975400
N	-3.33649900	2.33065200	-0.82542400
C	0.82149800	2.50834300	-0.79344800
C	1.51508000	2.06055800	-1.93285000
C	1.47191000	3.03056800	0.33891300
C	2.90957100	2.17715600	-1.92556100
C	2.86756900	3.12435800	0.29598100
C	3.58267000	2.70961800	-0.82658300
H	3.46727400	1.84419800	-2.79693400
H	3.39208800	3.52808800	1.15798400
H	4.66548500	2.79712900	-0.84351100
C	-4.77079300	2.33986400	-0.84837600
C	-5.42520700	1.95017500	-2.03074700
C	-5.46249100	2.72779000	0.31392900
C	-6.82423700	1.98473500	-2.03589200
C	-6.86056300	2.74637600	0.25592400
C	-7.53860100	2.38571000	-0.90788100
H	-7.35198600	1.69132600	-2.93952700
H	-7.41712900	3.04903600	1.13899400
H	-8.62442500	2.41276000	-0.93400700
C	0.69211200	3.42765400	1.56648000
H	-0.08646600	4.16025900	1.32914400
H	0.18872200	2.55877600	2.01189700

H	1.35330200	3.85522900	2.32567000
C	0.78334100	1.44442700	-3.09855400
H	0.28343500	0.51187300	-2.80366800
H	0.01132500	2.11771200	-3.48560200
H	1.47728700	1.20714100	-3.91001500
C	-4.72355100	3.07205000	1.58261000
H	-4.17660400	2.20198300	1.97040000
H	-3.98998900	3.86736000	1.41383800
H	-5.41994700	3.39990400	2.35970800
C	-4.64413000	1.46984300	-3.22732900
H	-3.91015700	2.21409200	-3.55377800
H	-4.08687900	0.55282900	-2.99238500
H	-5.31241800	1.24872100	-4.06450600
C	-2.69814500	1.29663500	-0.41153700
C	-1.19276500	1.34336200	-0.39303900
C	-3.10092100	-0.02553800	0.08328500
C	-4.31202700	-0.65797900	0.29544800
C	-1.89171900	-0.71615600	0.37128300
C	-4.29560400	-1.98559400	0.79864900
H	-5.25277900	-0.16112500	0.08750300
C	-1.85700800	-2.03177400	0.87153700
C	-0.72085400	0.04628500	0.10694900
C	-3.11696200	-2.65862100	1.08078500
H	-5.24411600	-2.48660800	0.96725900
C	-0.56604700	-2.58044100	1.10937200
C	0.52163300	-0.51077700	0.34716100
H	-3.15102800	-3.67448800	1.46566600
C	0.57520600	-1.83649900	0.85278100
H	-0.47848000	-3.59253300	1.49574300
H	1.43476100	0.04145400	0.15646500
H	1.54859600	-2.27861700	1.04294000

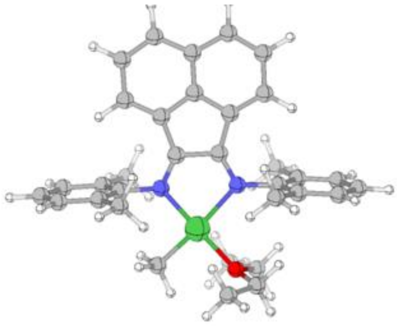

B3LYP-D3/BSI

Ni	-1.82272000	0.74040100	2.09963800
N	-0.37957600	-0.76269500	1.81385500
N	-3.04957100	-0.72238100	1.84579300
C	1.05213100	-0.71560800	1.80442300
C	1.71084100	-0.45693500	0.58953400
C	1.74494600	-0.91370700	3.01174300
C	3.10668000	-0.35574500	0.61324400
C	3.14014200	-0.80363500	2.98814400
C	3.81759700	-0.51955100	1.80222300
H	3.63663400	-0.15175400	-0.31325200
H	3.69628800	-0.94808300	3.91046800
H	4.89995000	-0.43319800	1.80328900
C	-4.48540300	-0.67093600	1.86773800
C	-5.16298500	-0.41967100	0.66516700
C	-5.14191300	-0.85956700	3.09326900
C	-6.56080700	-0.37985400	0.70871900
C	-6.54012800	-0.81120800	3.08997900
C	-7.24503000	-0.57766200	1.90860400
H	-7.11289300	-0.18669200	-0.20694400
H	-7.07613400	-0.95307200	4.02438600
H	-8.33014100	-0.54051700	1.92475600
C	1.00290000	-1.28199400	4.27407800
H	0.12268800	-0.64963900	4.42814300
H	0.64316500	-2.31826000	4.22890900
H	1.64837100	-1.19362400	5.15209100
C	0.93218600	-0.34530600	-0.69922100
H	0.57855000	-1.33076500	-1.02968400
H	0.04464300	0.28523900	-0.58484200
H	1.54984300	0.07029400	-1.49995900
C	-4.35180200	-1.11681100	4.35264300
H	-3.85853900	-2.09712700	4.32333600
H	-3.56264300	-0.36547700	4.48220600
H	-4.99701800	-1.09430300	5.23469300
C	-4.39503500	-0.21656000	-0.61758700
H	-3.60530400	0.53428200	-0.48863100

H	-3.90494700	-1.14354300	-0.94270400
H	-5.05487700	0.11258600	-1.42449800
C	-2.47643600	-1.86199300	1.63746600
C	-0.97778600	-1.88536000	1.61890600
C	-3.25883700	1.99530400	2.33996700
H	-3.86589200	1.67633000	3.19422500
H	-2.83504300	2.98564200	2.51816200
H	-3.87864100	1.99762000	1.43673000
C	-2.94797600	-3.22338700	1.39789600
C	-4.18749200	-3.83303900	1.30042100
C	-1.76845300	-4.00932200	1.24511800
C	-4.23019000	-5.22875600	1.05020400
H	-5.10691800	-3.26947600	1.41059000
C	-1.79438900	-5.39390100	0.99647200
C	-0.56100000	-3.26477000	1.36666300
C	-3.08189600	-5.99272900	0.90167600
H	-5.19941500	-5.71136900	0.97307000
C	-0.53004800	-6.03454900	0.86849000
C	0.65494100	-3.91310800	1.23755600
H	-3.16260700	-7.05931800	0.71090100
C	0.64668400	-5.30950600	0.98668900
H	-0.48977800	-7.10314000	0.67611500
H	1.59423100	-3.37814700	1.32404300
H	1.59699600	-5.82403300	0.88446900
O	-0.52689100	2.21504400	2.36418300
C	0.20816300	2.28390700	3.61156500
H	1.00516600	1.53315700	3.59360300
H	0.66844200	3.27720500	3.66245000
C	-0.74747300	2.08484300	4.77356700
H	-1.24540300	1.11026700	4.72247600
H	-0.19078100	2.13389400	5.71510200
H	-1.51909900	2.85922300	4.77726600
C	0.18805800	2.73765300	1.21654700
H	0.98094700	2.03324100	0.94399100
H	0.65329000	3.68045600	1.52602800
C	-0.78726400	2.97884500	0.07918800
H	-1.55226700	3.70290800	0.37170400
H	-0.24475600	3.36694800	-0.78906200
H	-1.29261800	2.05412200	-0.22022100

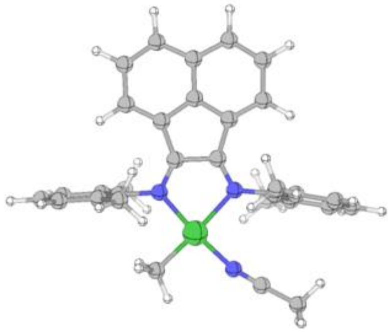

B3LYP-D3/BSI

Ni	-1.84908000	0.71488900	2.13264100
N	-0.43498100	-0.77378000	1.83993500
N	-3.09566500	-0.73661100	1.85287000
C	0.98293300	-0.62714100	1.86134600
C	1.68178400	-0.47178800	0.65053400
C	1.60800100	-0.51312500	3.11645000
C	3.05801000	-0.22449700	0.72284200
C	2.98604500	-0.27224900	3.14065400
C	3.70876200	-0.12965100	1.95425500
H	3.62047900	-0.10247200	-0.19889700
H	3.49277800	-0.19745300	4.09917600
H	4.77840900	0.05545100	1.98914000
C	-4.53055900	-0.68176600	1.89190700
C	-5.22613000	-0.42433400	0.70040900
C	-5.16891200	-0.86506600	3.12814000
C	-6.62268900	-0.37226300	0.76790200
C	-6.56654200	-0.80523800	3.14800900
C	-7.28875500	-0.56452700	1.97883600
H	-7.18817500	-0.17338800	-0.13831900
H	-7.08802400	-0.94406800	4.09103800
H	-8.37309700	-0.51774900	2.01286000
C	0.79792800	-0.65559700	4.38090800
H	-0.05764800	0.03223800	4.37841200
H	0.38760400	-1.66792000	4.48200000
H	1.40492800	-0.45000400	5.26674100
C	0.95977700	-0.54918600	-0.67306500
H	0.60158500	-1.56578200	-0.87738100
H	0.08193200	0.10875500	-0.68463700
H	1.61685700	-0.25742000	-1.49651600
C	-4.36037200	-1.13177700	4.37372700
H	-3.86906000	-2.11249000	4.33018000
H	-3.56737100	-0.38342100	4.49766400
H	-4.99234100	-1.11443200	5.26544400
C	-4.48080200	-0.22876900	-0.59702900

H	-3.68057100	0.51350900	-0.48617700
H	-4.00866900	-1.16137800	-0.93242300
H	-5.15351800	0.10745200	-1.39028400
C	-2.52130700	-1.86470600	1.58820200
C	-1.01943400	-1.88687200	1.57025300
C	-3.25522900	2.01505800	2.36600300
H	-3.86439600	1.67904700	3.21250600
H	-2.85950800	3.01563300	2.55265500
H	-3.85945100	2.00674500	1.45211200
C	-2.98875300	-3.21654800	1.29327800
C	-4.22643700	-3.82601900	1.17610500
C	-1.80731000	-3.99251400	1.10689600
C	-4.26549300	-5.21115400	0.87170600
H	-5.14688300	-3.26939700	1.31095200
C	-1.82919200	-5.36674200	0.80610300
C	-0.60118300	-3.25111200	1.25672900
C	-3.11527300	-5.96523800	0.69070300
H	-5.23339400	-5.69355700	0.77841000
C	-0.56291000	-5.99816600	0.65310000
C	0.61623000	-3.88963300	1.10270700
H	-3.19341100	-7.02385200	0.45886300
C	0.61239200	-5.27530400	0.79874000
H	-0.51969600	-7.05834700	0.41931700
H	1.55307900	-3.35298300	1.20803700
H	1.56384500	-5.78324700	0.67637100
N	-0.50747500	2.00800800	2.32497200
C	0.48159700	2.60422600	2.38817100
C	1.75582600	3.30354400	2.46174800
H	2.56333800	2.56683200	2.38281100
H	1.83733800	4.02507100	1.64275600
H	1.83938900	3.83457900	3.41522700

^{1}A

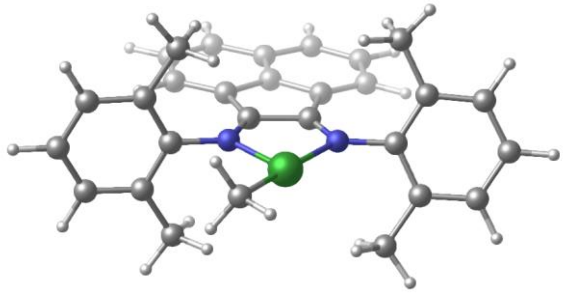

Ni	-1.99154500	3.72921100	-1.07778200
N	-0.56250000	2.35204400	-0.62505400
N	-3.23625400	2.30486900	-0.71881700
C	0.85110600	2.53337900	-0.67161800
C	1.62098600	1.89586900	-1.66673000
C	1.41056200	3.44088100	0.25142800
C	2.99662900	2.15582700	-1.68062400
C	2.78945700	3.65535800	0.20456200
C	3.58003700	3.01501300	-0.75076600
H	3.61179700	1.68471300	-2.44230600
H	3.24457100	4.33387000	0.92054600
H	4.64954500	3.19961000	-0.78081200
C	-4.66338500	2.38992900	-0.78057000
C	-5.30242400	2.06219200	-1.98758300
C	-5.35167000	2.83793300	0.35880400
C	-6.69681300	2.16372000	-2.02349200
C	-6.74494100	2.92364800	0.27555000
C	-7.41290400	2.58580500	-0.90228900
H	-7.22116200	1.91644000	-2.94226700
H	-7.30637100	3.26445100	1.14091800
H	-8.49498400	2.66183200	-0.95014300
C	0.54165400	4.12543700	1.27944100
H	-0.25291800	4.72419000	0.80806600
H	0.04294800	3.40385800	1.93734800
H	1.12938700	4.80329800	1.90371300
C	0.99923100	0.99558000	-2.70896600
H	0.81312500	-0.01317000	-2.32070800
H	0.03932100	1.38747500	-3.06406100
H	1.66082100	0.89632500	-3.57350200
C	-4.59707500	3.20259200	1.61257200
H	-4.10761900	2.32702300	2.05801500
H	-3.80772700	3.93497300	1.39706900
H	-5.26420500	3.63122200	2.36462800
C	-4.49848100	1.62107900	-3.18519900
H	-3.70699300	2.34591600	-3.41617900

H	-4.00804000	0.65509600	-3.00904000
H	-5.13305500	1.51633200	-4.06876300
C	-2.63501500	1.21130000	-0.38611000
C	-1.12752900	1.22859100	-0.33716800
C	-3.22774900	5.08040700	-1.56563800
H	-2.58648000	5.94815100	-1.80223700
H	-3.81074400	4.79128100	-2.44681200
H	-3.90635600	5.32503100	-0.74144600
C	-3.08032200	-0.11975000	0.00034200
C	-4.31188700	-0.73572400	0.14697100
C	-1.88881700	-0.84882300	0.28181300
C	-4.33537500	-2.08608900	0.57794600
H	-5.23665100	-0.20809300	-0.05859800
C	-1.89670300	-2.18557200	0.72009200
C	-0.69319600	-0.09795400	0.09316700
C	-3.17546600	-2.79492600	0.85871500
H	-5.29608700	-2.57751800	0.69402200
C	-0.62577000	-2.77122800	0.97903300
C	0.52872700	-0.69279900	0.35683800
H	-3.24119500	-3.82740600	1.19057800
C	0.53944700	-2.03882200	0.80357000
H	-0.57101900	-3.80166900	1.31886300
H	1.46017300	-0.15301300	0.22486000
H	1.49553900	-2.50876000	1.01112700

UB3LYP-D3/BSII(SMD)//B3LYP-D3/BSI

HF=-1402.0598939

^{3}A

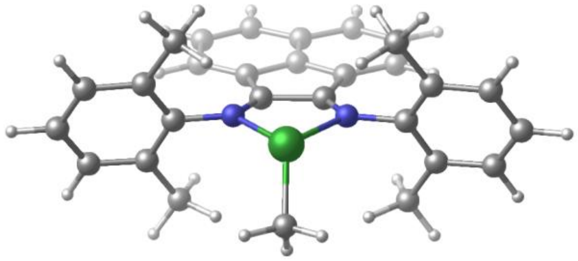

Zero-point correction= 0.481207 (Hartree/Particle)
Thermal correction to Energy= 0.511218
Thermal correction to Enthalpy= 0.512162
Thermal correction to Gibbs Free Energy= 0.418196
Sum of electronic and zero-point Energies= -1399.569944
Sum of electronic and thermal Energies= -1399.539933
Sum of electronic and thermal Enthalpies= -1399.538989
Sum of electronic and thermal Free Energies= -1399.632955

Ni	-2.04250100	3.74505500	-1.05138700
N	-0.65106000	2.38756900	-0.46541700
N	-3.36423000	2.31108600	-0.48647000
C	0.76180900	2.55960900	-0.55443200
C	1.48803300	1.91890800	-1.57786100
C	1.35749900	3.45805900	0.35151800
C	2.86475700	2.16294900	-1.63653900
C	2.73650100	3.66063100	0.25870300
C	3.48740600	3.01416000	-0.72360400
H	3.44966500	1.68713400	-2.41877800
H	3.22154200	4.33487900	0.95888800
H	4.55727300	3.18737500	-0.78868400
C	-4.78298400	2.40316100	-0.59717800
C	-5.45661700	1.72059700	-1.62952300
C	-5.44182600	3.26846200	0.29730400
C	-6.84390200	1.88652600	-1.70913800
C	-6.82849400	3.39273600	0.18363600
C	-7.52705600	2.70295600	-0.80781200
H	-7.38936500	1.37704000	-2.49863900
H	-7.36114600	4.03991100	0.87470700
H	-8.60390600	2.81539500	-0.88910500
C	0.52145800	4.15606500	1.39610900
H	-0.28041400	4.75309400	0.93415100
H	0.03532400	3.44432400	2.07412800
H	1.12797200	4.83732800	1.99823400
C	0.80979600	1.03271600	-2.59565900
H	0.54552900	0.05434700	-2.17590400
H	-0.11465500	1.48856900	-2.96881500

H	1.46677400	0.85610700	-3.45114500
C	-4.66215400	4.01496700	1.35193400
H	-4.14452200	3.33353500	2.03770800
H	-3.89040100	4.65751000	0.89978400
H	-5.31546900	4.66032500	1.94458400
C	-4.71473900	0.87198600	-2.63486600
H	-3.80901200	1.37585400	-2.99179700
H	-4.40702300	-0.09140400	-2.21010100
H	-5.34634700	0.66158500	-3.50174600
C	-2.73847700	1.24140000	-0.12852300
C	-1.22100300	1.28407600	-0.11718300
C	-2.04108900	4.30983000	-2.92084300
H	-2.95719100	4.88548900	-3.10577000
H	-1.15611600	4.93740800	-3.08776400
H	-2.00919200	3.43460200	-3.57722100
C	-3.13908800	-0.08184700	0.33832200
C	-4.35149900	-0.71020000	0.56868600
C	-1.92796300	-0.77421600	0.62143700
C	-4.33634100	-2.03172300	1.08174400
H	-5.29362100	-0.21448300	0.36233500
C	-1.89485200	-2.08439700	1.13382400
C	-0.75360900	-0.01498000	0.35541700
C	-3.15522400	-2.70605500	1.35692300
H	-5.28387600	-2.52903400	1.26296100
C	-0.60490700	-2.63470000	1.37462600
C	0.48872100	-0.57456500	0.60291100
H	-3.18950900	-3.71901800	1.74818700
C	0.54037500	-1.89523900	1.11580600
H	-0.51949400	-3.64437300	1.76657500
H	1.40437500	-0.02666900	0.40982700
H	1.51161900	-2.33890100	1.31029300

UB3LYP-D3/BSII(SMD)//B3LYP-D3/BSI

HF= -1402.0736328

^{1}B

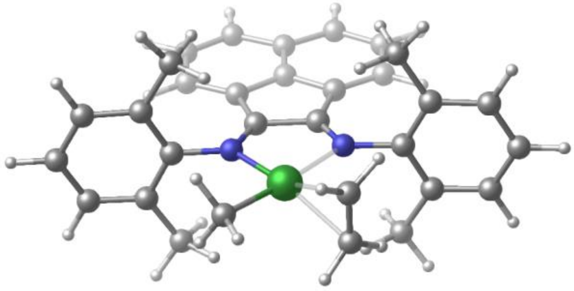

Zero-point correction= 0.539821 (Hartree/Particle)
Thermal correction to Energy= 0.572074
Thermal correction to Enthalpy= 0.573018
Thermal correction to Gibbs Free Energy= 0.476017
Sum of electronic and zero-point Energies= -1478.147931
Sum of electronic and thermal Energies= -1478.115678
Sum of electronic and thermal Enthalpies= -1478.114734
Sum of electronic and thermal Free Energies= -1478.211736

Ni	-1.77400900	0.76217400	2.11016100
N	-0.37826100	-0.79052100	1.81772700
N	-3.06311000	-0.72128800	1.82977800
C	1.05091400	-0.73359500	1.82846300
C	1.72181900	-0.45320100	0.62496700
C	1.72203900	-0.90940200	3.05150800
C	3.11596000	-0.33985500	0.67156200
C	3.11617800	-0.78703300	3.04895300
C	3.80870100	-0.50240400	1.87166500
H	3.65892400	-0.12275900	-0.24424900
H	3.65931500	-0.91750600	3.98095400
H	4.89004900	-0.40647900	1.88962000
C	-4.49602000	-0.64728600	1.84268600
C	-5.16195700	-0.36971900	0.63835200
C	-5.16385500	-0.82861700	3.06416700
C	-6.55849200	-0.29542900	0.67603400
C	-6.56036200	-0.74535400	3.05410600
C	-7.25318000	-0.48476500	1.87126500
H	-7.10127700	-0.08219600	-0.24068700
H	-7.10459300	-0.88158600	3.98454000
H	-8.33703600	-0.42029500	1.88260200
C	0.95501700	-1.25956900	4.30483300
H	0.08278400	-0.60896600	4.44471100
H	0.57426800	-2.28822800	4.26012900
H	1.58938700	-1.17890500	5.19142000
C	0.95471000	-0.32471300	-0.66998700
H	0.57894200	-1.30043100	-1.00453400

H	0.07918000	0.32762900	-0.56272800
H	1.58750900	0.07741200	-1.46550400
C	-4.38809000	-1.11951800	4.32521400
H	-3.92160100	-2.11255400	4.28838500
H	-3.57878400	-0.39252100	4.46990100
H	-5.03768200	-1.08898000	5.20372600
C	-4.38437600	-0.18003900	-0.64075000
H	-3.57469200	0.54893900	-0.50868300
H	-3.91814800	-1.11810500	-0.96900000
H	-5.03271400	0.17002800	-1.44809500
C	-2.49472300	-1.86101300	1.61456900
C	-0.99039000	-1.90146600	1.60769600
C	-3.19809000	2.03534200	2.35099400
H	-3.80243000	1.68196000	3.19428000
H	-2.84519000	3.05252700	2.54290800
H	-3.80350500	2.01378100	1.43767800
C	-0.25787500	2.42483100	1.72692900
H	0.46477600	1.93996000	1.07672600
H	-0.86886300	3.20934800	1.29077500
C	-0.25577200	2.17350300	3.06610200
H	-0.86502500	2.74614500	3.75883900
H	0.46869200	1.48644500	3.49404600
C	-2.97461400	-3.21587500	1.35804000
C	-4.21959200	-3.81198300	1.24437100
C	-1.80149000	-4.01189300	1.20840800
C	-4.27389600	-5.20452200	0.98106600
H	-5.13397400	-3.23966300	1.35184700
C	-1.83869700	-5.39380000	0.94715100
C	-0.58749400	-3.28149800	1.34717700
C	-3.13138100	-5.97837000	0.83567200
H	-5.24687200	-5.67710900	0.89096500
C	-0.57933500	-6.04558700	0.82478900
C	0.62350500	-3.93977600	1.22345500
H	-3.22122700	-7.04230300	0.63448500
C	0.60377500	-5.33360900	0.96007300
H	-0.54776200	-7.11273100	0.62315000
H	1.56657300	-3.41358900	1.32337100
H	1.54961200	-5.85694600	0.86176700

UB3LYP-D3/BSII(SMD)//B3LYP-D3/BSI

HF=-1480.7208426

^{3}B

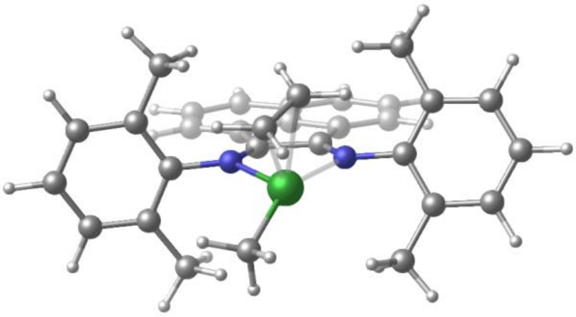

Zero-point correction= 0.536399 (Hartree/Particle)
Thermal correction to Energy= 0.569746
Thermal correction to Enthalpy= 0.570690
Thermal correction to Gibbs Free Energy= 0.470136
Sum of electronic and zero-point Energies= -1478.128599
Sum of electronic and thermal Energies= -1478.095252
Sum of electronic and thermal Enthalpies= -1478.094307
Sum of electronic and thermal Free Energies= -1478.194861

Ni	-2.11695100	3.70291400	-1.68136100
N	-0.71978500	2.43449000	-0.81424000
N	-3.43264900	2.26951200	-0.84272800
C	0.70607700	2.55599800	-0.90493100
C	1.33713500	2.08284100	-2.06941500
C	1.41443300	3.15300800	0.15195200
C	2.72423200	2.23489900	-2.16439700
C	2.80160900	3.27750500	0.01413400
C	3.45304900	2.82889900	-1.13438900
H	3.23345100	1.87926400	-3.05582600
H	3.37242100	3.73148000	0.81952900
H	4.52937300	2.93934100	-1.22501900
C	-4.85560400	2.35304300	-0.84938600
C	-5.48873600	2.41223400	-2.11016300
C	-5.57761300	2.47242200	0.35779000
C	-6.87771200	2.54546900	-2.14336600
C	-6.96785400	2.62472500	0.26829900
C	-7.61611400	2.65248700	-0.96369500
H	-7.38375000	2.56916400	-3.10438600
H	-7.54290400	2.72758800	1.18463700
H	-8.69490600	2.76714800	-1.00701000
C	0.71041000	3.62566200	1.40158000
H	0.06123400	2.84921600	1.82347200
H	1.43224400	3.90979400	2.17172200
H	0.08277000	4.50263200	1.20380400
C	0.54177900	1.39722400	-3.15318500
H	0.16871000	0.42150800	-2.81462500

H	-0.32870700	1.99256700	-3.44798400
H	1.15414600	1.22767700	-4.04248900
C	-4.91536500	2.45028400	1.71640300
H	-4.83351300	1.42872500	2.10744300
H	-3.90324900	2.86401200	1.69487100
H	-5.50018200	3.02891200	2.43700500
C	-4.68661600	2.30039600	-3.38548000
H	-4.02182200	3.16559900	-3.52748500
H	-4.05331700	1.40566700	-3.39381100
H	-5.34305200	2.25678800	-4.25852300
C	-2.77914700	1.27984100	-0.33489100
C	-1.26656300	1.37084500	-0.33653700
C	-1.28833700	4.63884800	-3.22110000
H	-1.85977600	5.52757000	-3.51454900
H	-0.25393600	4.92626200	-3.00722200
H	-1.29659900	3.92906200	-4.06075200
C	-2.63603300	5.01388900	0.27110800
H	-2.17865000	4.39006800	1.03266600
H	-3.72256700	5.02974900	0.23799400
C	-1.88947400	5.78636300	-0.55049100
H	-2.34763500	6.47046300	-1.25778000
H	-0.80601200	5.82155500	-0.48012200
C	-3.13204000	-0.02346400	0.22745100
C	-4.31701700	-0.70559900	0.44791700
C	-1.89666600	-0.65979400	0.53719100
C	-4.25145000	-2.01473200	0.98863000
H	-5.27807200	-0.25915400	0.21778600
C	-1.81275600	-1.95620400	1.07862300
C	-0.75145300	0.12655000	0.22999900
C	-3.04694400	-2.62728800	1.30274700
H	-5.17895200	-2.55084000	1.16322200
C	-0.50218100	-2.45054800	1.33029600
C	0.51246800	-0.38033700	0.48041800
H	-3.04245100	-3.63050600	1.71979900
C	0.61405400	-1.67999700	1.03922300
H	-0.37830500	-3.44429500	1.75173400
H	1.40642900	0.19070400	0.25657100
H	1.60175100	-2.08303400	1.23952500

UB3LYP-D3/BSII(SMD)//B3LYP-D3/BSI

HF=-1480.7114892

^{1}TS1

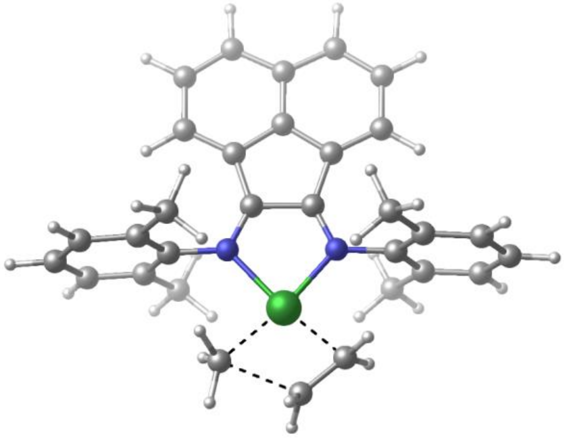

Zero-point correction= 0.538963 (Hartree/Particle)
Thermal correction to Energy= 0.570527
Thermal correction to Enthalpy= 0.571471
Thermal correction to Gibbs Free Energy= 0.476147
Sum of electronic and zero-point Energies= -1478.127425
Sum of electronic and thermal Energies= -1478.095861
Sum of electronic and thermal Enthalpies= -1478.094917
Sum of electronic and thermal Free Energies= -1478.190241

Ni	-1.64563100	0.76571900	2.03727700
N	-0.39841600	-0.74435100	1.77297500
N	-3.05387500	-0.71141300	1.79513300
C	1.03746600	-0.70901700	1.76994100
C	1.69922400	-0.46829200	0.55520200
C	1.71483800	-0.88695100	2.98688200
C	3.09765500	-0.42200000	0.57861000
C	3.11296900	-0.83249100	2.96283800
C	3.80024200	-0.60499100	1.77009000
H	3.63567800	-0.23779300	-0.34723000
H	3.66288900	-0.96729000	3.89019700
H	4.88524600	-0.56321100	1.77031000
C	-4.48290500	-0.62452900	1.81927300
C	-5.16045300	-0.35765500	0.61739700
C	-5.14392800	-0.76638700	3.05127100
C	-6.55395600	-0.24394100	0.67067100
C	-6.53774800	-0.64482200	3.05649700
C	-7.23843300	-0.38686100	1.87794200
H	-7.10276800	-0.03940800	-0.24450800
H	-7.07397100	-0.75185600	3.99542600
H	-8.31986000	-0.29201500	1.90122700
C	0.94718000	-1.14775200	4.25995500
H	0.14276000	-0.41421200	4.39975000
H	0.47227900	-2.13725800	4.24422500

H	1.60426200	-1.10758900	5.13255600
C	0.91522500	-0.28997900	-0.72214300
H	0.43889400	-1.22887300	-1.03276200
H	0.11025900	0.44587400	-0.59842800
H	1.56140300	0.04170400	-1.53900000
C	-4.36653000	-1.07360600	4.30859700
H	-3.95544700	-2.09117800	4.28297100
H	-3.51480100	-0.39329900	4.43471200
H	-5.00201700	-0.99369300	5.19436500
C	-4.40074800	-0.23620800	-0.68173300
H	-3.54575300	0.44530800	-0.58906100
H	-3.99680000	-1.20676800	-0.99793800
H	-5.04654800	0.13221300	-1.48300400
C	-2.49371300	-1.85357700	1.59793200
C	-0.99585200	-1.87221600	1.58500500
C	-3.16884200	2.13057500	2.27458500
H	-3.60691300	1.56234100	3.09978100
H	-3.37286000	3.18171500	2.45282400
H	-3.61227100	1.86473200	1.31094700
C	-0.14641900	2.00969100	2.24371900
H	0.41169800	1.67336100	3.11613200
H	0.40565000	1.98312100	1.30555600
C	-1.19997700	2.92686900	2.40415400
H	-1.42412900	3.61211600	1.59300600
H	-1.41821400	3.30355900	3.39827400
C	-2.95641900	-3.22074100	1.36921600
C	-4.19289500	-3.83489200	1.27337800
C	-1.77292300	-4.00087900	1.22890200
C	-4.22932900	-5.23327800	1.03647800
H	-5.11398900	-3.27170500	1.37507600
C	-1.79168000	-5.38772800	0.99369100
C	-0.56830800	-3.24829900	1.34841500
C	-3.07650500	-5.99289800	0.89978600
H	-5.19579500	-5.72147900	0.96025300
C	-0.52370700	-6.02340900	0.87707100
C	0.65130000	-3.89301500	1.23052000
H	-3.15163900	-7.06162200	0.71901700
C	0.64929300	-5.29150600	0.99314700
H	-0.47722700	-7.09357800	0.69517200
H	1.58803500	-3.35370000	1.31555300
H	1.60224800	-5.80273900	0.89977300

UB3LYP-D3/BSII(SMD)//B3LYP-D3/BSI

HF=-1480.6979817

^{3}TS1

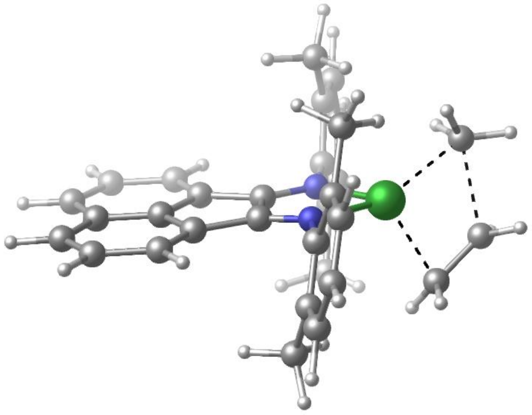

Zero-point correction= 0.536642 (Hartree/Particle)
Thermal correction to Energy= 0.568970
Thermal correction to Enthalpy= 0.569914
Thermal correction to Gibbs Free Energy= 0.471278
Sum of electronic and zero-point Energies= -1478.103349
Sum of electronic and thermal Energies= -1478.071021
Sum of electronic and thermal Enthalpies= -1478.070077
Sum of electronic and thermal Free Energies= -1478.168713

Ni	-2.19486800	3.75188200	-1.34254500
N	-0.74025000	2.40559200	-0.73565400
N	-3.43116800	2.21684200	-0.83469100
C	0.68074700	2.54238200	-0.84515600
C	1.28389400	2.18742900	-2.06536000
C	1.40917800	3.05853500	0.23967000
C	2.66645000	2.36767000	-2.18365400
C	2.78984700	3.21699800	0.07688800
C	3.41523500	2.87826000	-1.12342200
H	3.15567000	2.10146700	-3.11660600
H	3.37621900	3.61137100	0.90228600
H	4.48707400	3.01317600	-1.23254800
C	-4.85551500	2.29690100	-0.85016300
C	-5.49124700	2.24942800	-2.10570700
C	-5.56337000	2.51331600	0.34897000
C	-6.88300800	2.36842500	-2.13964300
C	-6.95484100	2.63864400	0.26229600
C	-7.61205500	2.55761400	-0.96512600
H	-7.39607900	2.31600900	-3.09588300
H	-7.52467500	2.81114000	1.17127900
H	-8.69256500	2.65580000	-1.00800600
C	0.71950500	3.39732300	1.53828600
H	0.29891300	2.50206000	2.01374100
H	1.41478800	3.85597100	2.24607100

H	-0.11246600	4.09215300	1.37851700
C	0.46127700	1.59885900	-3.18673300
H	0.09901400	0.59511600	-2.92949600
H	-0.42339000	2.20978300	-3.40309700
H	1.04907200	1.51554900	-4.10453400
C	-4.85313900	2.63160200	1.67698700
H	-4.54060600	1.65221900	2.05970000
H	-3.95152000	3.24948000	1.59523500
H	-5.50817700	3.08338900	2.42661300
C	-4.68204300	2.04783900	-3.36367400
H	-3.90744100	2.82117200	-3.47021900
H	-4.16108800	1.08277200	-3.36107600
H	-5.31659400	2.08816800	-4.25276000
C	-2.78477000	1.22516000	-0.31738600
C	-1.27904400	1.31295900	-0.31352500
C	-1.16844500	5.00343200	-2.86199700
H	-1.04899400	5.99849100	-3.28119000
H	-0.19369400	4.53624800	-2.70464800
H	-1.81418800	4.42471100	-3.54136200
C	-2.50223200	5.32889500	-0.02691000
H	-1.95906300	5.09833600	0.88508500
H	-3.58382400	5.41478500	0.07176800
C	-1.84515000	6.01158800	-1.09275800
H	-2.44779900	6.67305000	-1.70651900
H	-0.82099800	6.33731300	-0.93934400
C	-3.14815500	-0.07414400	0.24428200
C	-4.33839700	-0.73533600	0.49436800
C	-1.91528400	-0.73055700	0.52280000
C	-4.27972300	-2.04880500	1.02684700
H	-5.29714700	-0.26889500	0.29581000
C	-1.83843100	-2.03214800	1.05114700
C	-0.76635700	0.04780000	0.20723300
C	-3.07787000	-2.68488400	1.30167900
H	-5.21106500	-2.56961500	1.22584900
C	-0.52950000	-2.54625900	1.27081800
C	0.49504600	-0.47680500	0.42934900
H	-3.07941200	-3.69120700	1.71123000
C	0.59062900	-1.78615300	0.96718200
H	-0.40996400	-3.54575100	1.67976200
H	1.39178400	0.08832500	0.20015600
H	1.57672800	-2.20400100	1.14388500

UB3LYP-D3/BSII(SMD)//B3LYP-D3/BSI

HF=-1480.6797945

^{1}C

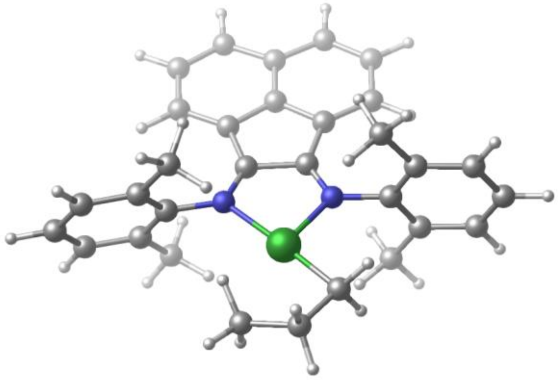

Zero-point correction= 0.540394 (Hartree/Particle)
Thermal correction to Energy= 0.572271
Thermal correction to Enthalpy= 0.573216
Thermal correction to Gibbs Free Energy= 0.476808
Sum of electronic and zero-point Energies= -1478.152552
Sum of electronic and thermal Energies= -1478.120675
Sum of electronic and thermal Enthalpies= -1478.119731
Sum of electronic and thermal Free Energies= -1478.216138

Ni	-1.67855900	0.61094000	1.90354700
N	-0.44454200	-0.81924900	1.70832400
N	-3.10710100	-0.83835000	1.70197500
C	0.98512700	-0.71782800	1.69784000
C	1.61861100	-0.42229700	0.48005500
C	1.68109300	-0.87813500	2.90683000
C	3.01320300	-0.31476300	0.48819600
C	3.07462600	-0.76079900	2.86679700
C	3.73636000	-0.48651900	1.66910900
H	3.53158200	-0.08976700	-0.43971900
H	3.64129200	-0.87966600	3.78614600
H	4.81841800	-0.39671000	1.65812300
C	-4.52410700	-0.67718900	1.73589700
C	-5.13161900	-0.07710400	0.61655200
C	-5.24358900	-1.02036100	2.89655100
C	-6.51173000	0.14069700	0.66180500
C	-6.62149700	-0.77545400	2.89707000
C	-7.25344000	-0.20725100	1.79084700
H	-7.00514300	0.58792000	-0.19671000
H	-7.19995000	-1.02578800	3.78216900
H	-8.32367400	-0.02546100	1.81330700
C	0.93928300	-1.17140600	4.18755200
H	0.11995500	-0.45779300	4.34186700
H	0.49226200	-2.17377900	4.17295600
H	1.60769900	-1.11792800	5.05065800

C	0.80528200	-0.24007100	-0.77680600
H	0.32452800	-1.17760100	-1.08419500
H	0.00137500	0.49168400	-0.62137700
H	1.42925200	0.10381900	-1.60567300
C	-4.55225400	-1.60764400	4.10453200
H	-4.25607600	-2.65061100	3.93694800
H	-3.64116300	-1.05109500	4.35571900
H	-5.21105400	-1.58840100	4.97657600
C	-4.31258300	0.27162600	-0.60282700
H	-3.43205400	0.87509400	-0.34239400
H	-3.93281100	-0.62911500	-1.10124800
H	-4.90524000	0.83348800	-1.32945100
C	-2.53048900	-1.98582900	1.60867500
C	-1.02619300	-1.96952600	1.60076900
C	-2.78668700	2.59140300	2.55695300
H	-2.84570700	2.20065900	3.57978600
H	-3.55605400	3.36309800	2.45396600
H	-3.16873600	1.81891900	1.84379700
C	-0.39409400	1.98262700	2.16680900
H	0.11228100	1.78365200	3.11905700
H	0.33814100	2.00556600	1.35482300
C	-1.37823800	3.14288200	2.20375900
H	-1.43115400	3.63201600	1.22585900
H	-1.10713100	3.90860300	2.94052900
C	-2.95316800	-3.37641500	1.45993100
C	-4.17204700	-4.02691500	1.38592700
C	-1.74969300	-4.13124100	1.36205100
C	-4.17184600	-5.43508400	1.21336500
H	-5.10775500	-3.48322500	1.45990600
C	-1.73104900	-5.52751500	1.19164600
C	-0.56459000	-3.34280200	1.44402100
C	-2.99899500	-6.17018000	1.11961900
H	-5.12503800	-5.95096300	1.15470300
C	-0.44625800	-6.13418100	1.10888600
C	0.67225000	-3.95956600	1.35862400
H	-3.04521700	-7.24785900	0.98979100
C	0.70745300	-5.36723900	1.19093200
H	-0.37121200	-7.21030200	0.97914000
H	1.59367700	-3.39102400	1.41765900
H	1.67348500	-5.85754400	1.12391600

UB3LYP-D3/BSII(SMD)//B3LYP-D3/BSI

HF=-1480.7262117

^{3}C

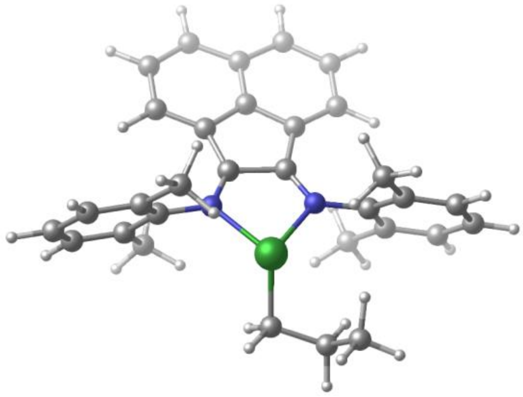

Zero-point correction= 0.539284 (Hartree/Particle)
Thermal correction to Energy= 0.571615
Thermal correction to Enthalpy= 0.572560
Thermal correction to Gibbs Free Energy= 0.473946
Sum of electronic and zero-point Energies= -1478.148816
Sum of electronic and thermal Energies= -1478.116485
Sum of electronic and thermal Enthalpies= -1478.115541
Sum of electronic and thermal Free Energies= -1478.214155

Ni	-2.30827100	3.48010000	-1.61908900
N	-0.84764600	2.21650700	-0.99659700
N	-3.55392400	2.04146100	-0.89946000
C	0.55011000	2.48592700	-1.06325500
C	1.15915300	2.42111000	-2.33100700
C	1.24032400	2.90771700	0.09054500
C	2.51507300	2.74511000	-2.42156100
C	2.59406100	3.23237200	-0.05370900
C	3.22973300	3.14561600	-1.29219200
H	3.01022300	2.68731900	-3.38677700
H	3.14957100	3.56790900	0.81767300
H	4.28095100	3.40286100	-1.37962700
C	-4.97820400	2.10752200	-0.87967100
C	-5.64410100	1.97699400	-2.11353400
C	-5.65660300	2.38995000	0.32252500
C	-7.03797300	2.06930800	-2.11691900
C	-7.05176100	2.48492600	0.26558300
C	-7.73921300	2.31538400	-0.93608300
H	-7.57448600	1.95282500	-3.05437500
H	-7.60067600	2.70758200	1.17643900
H	-8.82208200	2.39232800	-0.95528100
C	0.54653100	3.03611600	1.42621900
H	0.37974500	2.05820500	1.89420500
H	1.14731000	3.63305500	2.11726200
H	-0.43220800	3.51932000	1.32557400

C	0.36308800	1.98974300	-3.53802100
H	-0.00804800	0.96295500	-3.43430800
H	-0.51911400	2.63015700	-3.68721700
H	0.96542800	2.04207900	-4.44854400
C	-4.91411400	2.61704900	1.61821700
H	-4.56433400	1.67598400	2.06015200
H	-4.03401000	3.25331900	1.46890200
H	-5.56097600	3.10407200	2.35251900
C	-4.86243200	1.72500600	-3.37950600
H	-4.12695200	2.52409200	-3.56127100
H	-4.30070200	0.78426200	-3.33292900
H	-5.52179700	1.68340900	-4.25022400
C	-2.87269800	1.09954000	-0.34012500
C	-1.35987600	1.20111600	-0.38892700
C	-0.45274500	5.93820200	-2.39018600
H	0.51351700	6.44755800	-2.46432300
H	-0.30502300	4.92293500	-2.79032500
H	-1.16349700	6.45759700	-3.04373300
C	-2.34285700	5.27355200	-0.80014500
H	-2.67462400	5.17374500	0.23932600
H	-3.11235400	5.81295100	-1.37569400
C	-0.95946300	5.89747300	-0.94212200
H	-0.98465000	6.92415300	-0.54321800
H	-0.23893800	5.34905000	-0.32237000
C	-3.20116200	-0.14386700	0.35215500
C	-4.37669800	-0.79858400	0.67874800
C	-1.95330000	-0.74220300	0.68533000
C	-4.28795500	-2.05025500	1.33920600
H	-5.34564800	-0.37249000	0.44308700
C	-1.84676700	-1.98403100	1.33871900
C	-0.82226500	0.02223700	0.28291200
C	-3.07106600	-2.63168500	1.66491300
H	-5.20691800	-2.56671100	1.59775200
C	-0.52696400	-2.45104000	1.59337700
C	0.45050600	-0.45817300	0.54031800
H	-3.04904900	-3.59179300	2.17293200
C	0.57619100	-1.70581700	1.20209300
H	-0.38508700	-3.40232800	2.09866800
H	1.33459300	0.09873600	0.24982300
H	1.57158400	-2.08664900	1.40755100

UB3LYP-D3/BSII(SMD)//B3LYP-D3/BSI

HF=-1480.7310112

^{1}D

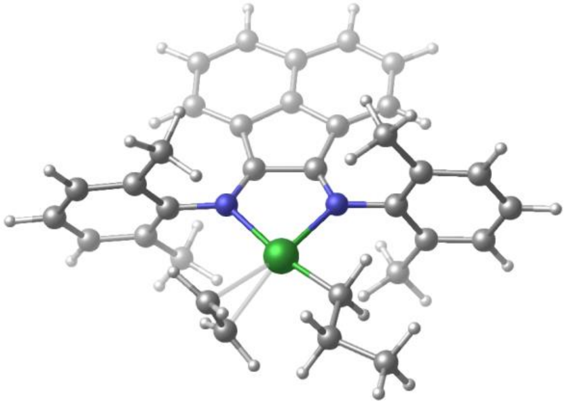

Zero-point correction= 0.597459 (Hartree/Particle)
Thermal correction to Energy= 0.632457
Thermal correction to Enthalpy= 0.633401
Thermal correction to Gibbs Free Energy= 0.530500
Sum of electronic and zero-point Energies= -1556.719924
Sum of electronic and thermal Energies= -1556.684926
Sum of electronic and thermal Enthalpies= -1556.683982
Sum of electronic and thermal Free Energies= -1556.786883

Ni	-2.21378700	3.49860000	-1.65330400
N	-0.83010900	2.21088400	-0.96711900
N	-3.50706100	2.11084700	-0.64586300
C	0.59332000	2.29053900	-1.12644000
C	1.18301300	1.64330000	-2.22450700
C	1.32919200	3.03887900	-0.19375600
C	2.57107100	1.74590600	-2.36607700
C	2.71424200	3.11459000	-0.37529300
C	3.33180100	2.47217000	-1.44912600
H	3.05445800	1.25484700	-3.20612000
H	3.30945700	3.68622600	0.33145500
H	4.40780200	2.54421000	-1.57601000
C	-4.93104300	2.12916800	-0.52810200
C	-5.68461800	1.65751200	-1.61896500
C	-5.52444700	2.66514900	0.62925500
C	-7.07840600	1.73384800	-1.53026400
C	-6.92260100	2.72544100	0.67007700
C	-7.69452900	2.26591600	-0.39707700
H	-7.68155800	1.37216000	-2.35852100
H	-7.40645500	3.13901200	1.55076900
H	-8.77751300	2.32448800	-0.34637300
C	0.63818300	3.70840400	0.96876700
H	0.26291100	2.96970700	1.68900300
H	1.32030300	4.37591800	1.50165900
H	-0.22699300	4.29430400	0.63406600

C	0.33503400	0.85870200	-3.19498500
H	-0.51004000	1.45747300	-3.55864500
H	0.92154900	0.53423100	-4.05837000
H	-0.08947400	-0.03655600	-2.72261800
C	-4.68342000	3.12015300	1.79927900
H	-4.28369200	2.26189000	2.35476500
H	-3.82091700	3.71706700	1.48036100
H	-5.27401400	3.71921700	2.49739600
C	-4.99079100	1.05631900	-2.81778400
H	-4.17421600	1.69748000	-3.17486000
H	-4.53982600	0.08689400	-2.56948700
H	-5.69067800	0.89757900	-3.64244200
C	-2.81350900	1.18341200	-0.08860900
C	-1.31993500	1.24610200	-0.26217300
C	-0.83433700	4.56255000	-2.51836300
H	-0.27994000	4.96702300	-1.65848900
H	-0.21218600	3.78646700	-2.98002900
C	-3.98343500	4.64023600	-2.52480600
H	-4.77360900	3.89626800	-2.53697400
H	-3.63068000	5.00299000	-3.48271500
C	-3.60634400	5.22425000	-1.35131000
H	-2.94090800	6.08129500	-1.32230500
H	-4.09344300	4.94814300	-0.41972700
C	-1.13656400	5.64879800	-3.54099900
H	-1.66371700	5.22040500	-4.40356600
H	-1.78615900	6.42872300	-3.12416700
C	0.16403000	6.30896900	-4.03590700
H	-0.05003600	7.08126600	-4.78318400
H	0.70465800	6.77898100	-3.20626500
H	0.83033400	5.56884300	-4.49364100
C	-3.11313000	0.00028300	0.71828500
C	-4.26997200	-0.59058400	1.19565400
C	-1.85255700	-0.59195000	1.00999800
C	-4.14945300	-1.77604500	1.96556300
H	-5.24743000	-0.16664400	0.99373600
C	-1.71495700	-1.76620500	1.77326900
C	-0.74365100	0.11093000	0.45582000
C	-2.92039500	-2.35197300	2.25164400
H	-5.05331900	-2.24431700	2.34234800
C	-0.38619300	-2.23226100	1.97760800
C	0.53863700	-0.36687400	0.67070200
H	-2.87374400	-3.25978800	2.84674900
C	0.69437700	-1.54769200	1.44067800
H	-0.21977600	-3.13403200	2.56040100

H	1.40786600	0.13884200	0.26638600
H	1.69738400	-1.92551900	1.61220900

UB3LYP-D3/BSII(SMD)//B3LYP-D3/BSI

HF=-1559.3733182

^{3}D

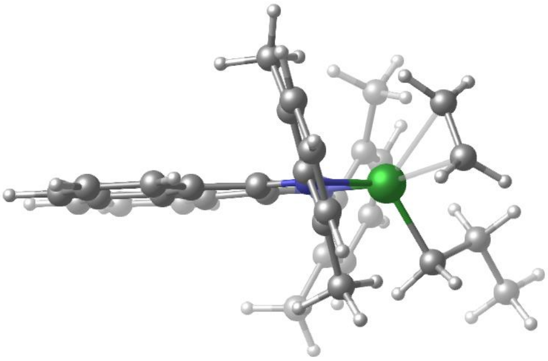

Zero-point correction=		0.594305 (Hartree/Particle)	
Thermal correction to Energy=		0.630127	
Thermal correction to Enthalpy=		0.631071	
Thermal correction to Gibbs Free Energy=		0.525639	
Sum of electronic and zero-point Energies=		-1556.712470	
Sum of electronic and thermal Energies=		-1556.676649	
Sum of electronic and thermal Enthalpies=		-1556.675705	
Sum of electronic and thermal Free Energies=		-1556.781137	
Ni	-1.83851000	3.70519300	-1.32615500
N	-0.46545000	2.28703800	-0.75438800
N	-3.16900400	2.19392000	-0.85136600
C	0.94160300	2.49095900	-0.62521300
C	1.70605000	2.57063500	-1.80531700
C	1.50028000	2.69671800	0.65212500
C	3.07070400	2.84919700	-1.68310100
C	2.86933900	2.98200700	0.72184700
C	3.65017700	3.05635900	-0.43086700
H	3.68093100	2.90392000	-2.58038400
H	3.32258500	3.15034600	1.69500900
H	4.70990700	3.27993400	-0.35383700
C	-4.57023200	2.24757000	-1.08734600
C	-5.15531600	1.52991900	-2.15252700
C	-5.31384700	3.14734400	-0.29332400
C	-6.52607000	1.71438300	-2.37891600
C	-6.67923900	3.28157900	-0.54723000
C	-7.28504100	2.56980400	-1.58418800
H	-6.99688800	1.17958300	-3.19945500
H	-7.26956200	3.95533600	0.06744600
H	-8.34605600	2.69270200	-1.77931900
C	0.66774300	2.59875400	1.90973400
H	1.17434700	3.08087500	2.75012400
H	-0.31697200	3.06548600	1.79199800
H	0.48898200	1.55262900	2.18832300
C	1.06261500	2.32438300	-3.14749500

H	0.72998300	1.28301000	-3.24355700
H	0.17254300	2.94902300	-3.28395000
H	1.76025400	2.53037300	-3.96356900
C	-4.63762200	3.93160500	0.80390800
H	-4.15833400	3.27642200	1.54047600
H	-3.84666400	4.57923500	0.39741300
H	-5.35133100	4.57150500	1.32937500
C	-4.36882200	0.60188400	-3.04896900
H	-3.32404000	0.90816700	-3.15298700
H	-4.36615000	-0.42398300	-2.66056700
H	-4.81134300	0.57162300	-4.04872000
C	-2.54496000	1.11610000	-0.50738800
C	-1.04091000	1.18302400	-0.41367800
C	-2.42439900	3.97265000	-3.24670300
H	-1.60514300	4.34648800	-3.86841100
H	-2.75783800	2.99555300	-3.60765400
C	-0.90225500	5.55334800	-0.20406300
H	-0.37747700	5.02963500	0.59035200
H	-1.77360000	6.13965000	0.07977500
C	-0.42070800	5.57037400	-1.47028900
H	-0.88127600	6.17659900	-2.24394900
H	0.50608100	5.06307500	-1.72675100
C	-3.56870700	4.96816000	-3.09467700
H	-4.24294100	4.67224000	-2.27744900
H	-3.17646700	5.96057200	-2.83381500
C	-4.41899100	5.07700800	-4.37796700
H	-5.22041100	5.81403800	-4.25282300
H	-3.80222900	5.37901100	-5.23126000
H	-4.87758900	4.11010200	-4.61259400
C	-0.57044100	-0.12190600	0.04750700
C	0.67026600	-0.67385000	0.31627500
C	-1.73913900	-0.91579700	0.21514200
C	0.72570100	-2.02103800	0.75645400
H	1.58257100	-0.09871700	0.20239300
C	-1.70391400	-2.24845600	0.66621100
C	-2.94607300	-0.23302300	-0.10602700
C	-0.41534500	-2.79000200	0.93274600
H	1.69640800	-2.45884300	0.96694900
C	-2.96068400	-2.90032500	0.80771100
C	-4.15411800	-0.89081300	0.04853100
H	-0.32818100	-3.81599900	1.27947800
C	-4.13905500	-2.23156300	0.51181900
H	-2.99380000	-3.93004200	1.15263400
H	-5.09627800	-0.40460500	-0.17931700

H	-5.08568300	-2.74891800	0.63241500

UB3LYP-D3/BSII(SMD)//B3LYP-D3/BSI

HF=-1559.3694633

[1]TS2

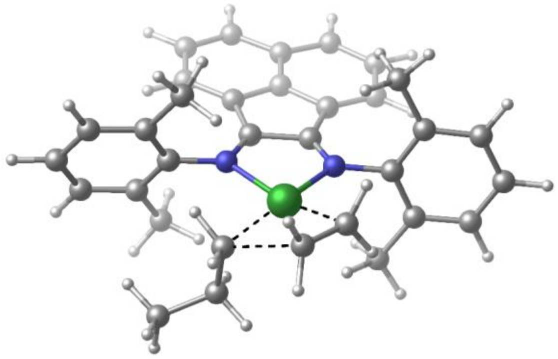

Zero-point correction= 0.597130 (Hartree/Particle)
Thermal correction to Energy= 0.631214
Thermal correction to Enthalpy= 0.632158
Thermal correction to Gibbs Free Energy= 0.531954
Sum of electronic and zero-point Energies= -1556.703600
Sum of electronic and thermal Energies= -1556.669515
Sum of electronic and thermal Enthalpies= -1556.668571
Sum of electronic and thermal Free Energies= -1556.768775

Ni	-2.37273300	3.80563000	-1.19304200
N	-0.84118700	2.42774800	-0.78523300
N	-3.48907800	2.33101300	-0.50759600
C	0.57329600	2.57379000	-0.93380500
C	1.24750500	1.92128800	-1.98368500
C	1.23087800	3.43956100	-0.03701500
C	2.62038300	2.16234800	-2.11861400
C	2.60321400	3.64391200	-0.20939600
C	3.29440300	3.01437100	-1.24419600
H	3.16223100	1.67452300	-2.92440100
H	3.12998900	4.30221800	0.47580400
H	4.35862500	3.18869100	-1.37068600
C	-4.92117700	2.31683600	-0.40556900
C	-5.66211500	2.02838200	-1.56324800
C	-5.52018900	2.62793800	0.82586800
C	-7.05718400	2.03513000	-1.45930400
C	-6.91879600	2.62519500	0.88253200
C	-7.68227600	2.32792700	-0.24670000
H	-7.65407000	1.81157000	-2.33927200
H	-7.40910400	2.86317200	1.82254300
H	-8.76624400	2.33231700	-0.18355800
C	0.47069500	4.08440500	1.09659200
H	0.11878000	3.33423600	1.81595300
H	1.09777100	4.79931300	1.63567600
H	-0.42198600	4.61280800	0.73614700
C	0.54179300	0.96704000	-2.91818500

H	-0.47068500	1.30055000	-3.16388300
H	1.09810400	0.85686900	-3.85291000
H	0.45073600	-0.02980900	-2.46816800
C	-4.67691700	2.93281200	2.04037600
H	-4.16663700	2.03324200	2.40803300
H	-3.89668800	3.67007300	1.81383500
H	-5.29025000	3.32342500	2.85653100
C	-4.95688400	1.71215900	-2.85926100
H	-4.22088800	2.48806900	-3.10977700
H	-4.40517300	0.76555700	-2.79532500
H	-5.66660100	1.63086200	-3.68667400
C	-2.82382500	1.28173900	-0.15956400
C	-1.33162400	1.34590600	-0.28713000
C	-1.05259800	5.04878600	-2.23865800
H	-0.86630300	6.11220900	-2.37513500
H	-0.25720800	4.70555800	-1.57480100
C	-3.90650200	5.01483900	-1.21113800
H	-4.23287100	5.04897900	-0.17256900
H	-4.65036100	4.64086100	-1.91258000
C	-2.91560000	5.90823700	-1.66123300
H	-2.97068300	6.26186500	-2.68521700
H	-2.47953900	6.61202500	-0.95785700
C	-1.07461800	4.34203700	-3.58992700
H	-1.25082600	3.26591600	-3.47271900
H	-1.90919000	4.72481600	-4.19137000
C	0.24876300	4.54933100	-4.34818000
H	0.22053000	4.04391000	-5.31974500
H	0.43526600	5.61440700	-4.52643200
H	1.09199200	4.14699300	-3.77731900
C	-3.17095700	-0.02923500	0.38432100
C	-4.35027900	-0.68018100	0.70456900
C	-1.92654700	-0.68691200	0.60525900
C	-4.26858600	-1.98874700	1.24587700
H	-5.31603200	-0.21185600	0.55218700
C	-1.82844800	-1.98088700	1.14993100
C	-0.79176300	0.08754700	0.23076900
C	-3.05650100	-2.62581300	1.46752100
H	-5.19030200	-2.50417000	1.49691800
C	-0.51271600	-2.49399300	1.32323700
C	0.47618900	-0.43670700	0.41260500
H	-3.04070900	-3.62713900	1.88895400
C	0.59367800	-1.73735800	0.96658600
H	-0.37714300	-3.48811300	1.74013800
H	1.36448300	0.12235500	0.14044800

H	1.58654800	-2.15141100	1.11163000

UB3LYP-D3/BSII(SMD)//B3LYP-D3/BSI

HF=-1559.3549407

^{3}TS2

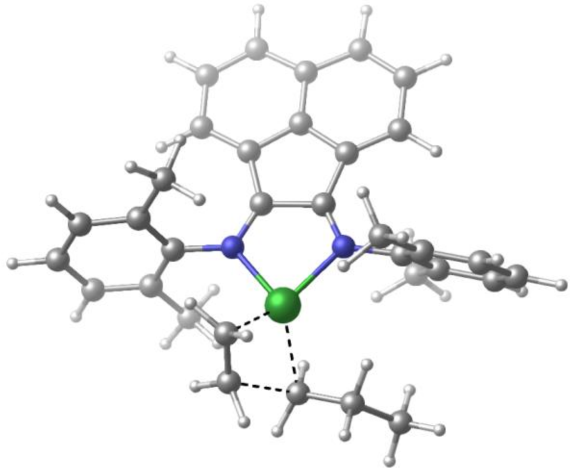

Zero-point correction= 0.594768 (Hartree/Particle)
Thermal correction to Energy= 0.629524
Thermal correction to Enthalpy= 0.630468
Thermal correction to Gibbs Free Energy= 0.526913
Sum of electronic and zero-point Energies= -1556.683727
Sum of electronic and thermal Energies= -1556.648971
Sum of electronic and thermal Enthalpies= -1556.648027
Sum of electronic and thermal Free Energies= -1556.751582

Ni	-1.86219000	3.71932300	-1.27154300
N	-0.45387200	2.24682600	-0.78801600
N	-3.15625200	2.19457300	-0.86034700
C	0.94896000	2.45526100	-0.63595600
C	1.73679600	2.51741200	-1.80027100
C	1.48071100	2.68598400	0.64878500
C	3.10150300	2.78191100	-1.65466200
C	2.85038200	2.96025400	0.74261300
C	3.65713400	3.00209600	-0.39395400
H	3.73062500	2.81749800	-2.53984800
H	3.28302300	3.14818600	1.72150300
H	4.71761300	3.21468900	-0.29816000
C	-4.56757700	2.26995300	-1.05995000
C	-5.14778100	1.65473800	-2.18729300
C	-5.31032400	3.07081700	-0.17128000
C	-6.52134300	1.83369800	-2.38714400
C	-6.68093300	3.21154800	-0.40738900
C	-7.28494300	2.59729900	-1.50470200
H	-6.99107800	1.37439600	-3.25265600
H	-7.27599200	3.81181700	0.27525600
H	-8.34939400	2.72279700	-1.67844900
C	0.60817400	2.65070300	1.88191000
H	1.12232400	3.10391300	2.73358400

H	-0.33347800	3.19021600	1.72571300
H	0.34747100	1.62258900	2.16224100
C	1.11612600	2.27483900	-3.15383500
H	0.69863900	1.26331800	-3.23110900
H	0.29093100	2.97250200	-3.33896100
H	1.85153300	2.39728600	-3.95340200
C	-4.63665400	3.73266800	1.00569500
H	-4.21377100	2.99339200	1.69677800
H	-3.80113800	4.36936700	0.68268800
H	-5.34058500	4.35504200	1.56433500
C	-4.31951000	0.85243400	-3.16332600
H	-3.38256800	1.36456900	-3.41317200
H	-4.04924700	-0.12981800	-2.75710600
H	-4.87032500	0.68458300	-4.09264000
C	-2.54591500	1.11037200	-0.51152000
C	-1.03996500	1.16016900	-0.41571800
C	-2.00747200	4.64505100	-3.31727000
H	-1.44286800	5.17836400	-4.07956900
H	-1.91649300	3.56757700	-3.52201900
C	-0.95413400	5.41209800	-0.47034700
H	-0.13679400	4.99013800	0.10861900
H	-1.67118000	6.01949700	0.07828700
C	-0.77839600	5.63729200	-1.86797300
H	-1.23239100	6.52905100	-2.28824600
H	0.17580000	5.36176500	-2.31010600
C	-3.44331600	5.12368800	-3.15784900
H	-3.89927900	4.75506400	-2.22223600
H	-3.45549800	6.21737900	-3.08077100
C	-4.33702100	4.66287400	-4.32186300
H	-5.36078400	5.02818800	-4.18870900
H	-3.95907300	5.03841200	-5.27879800
H	-4.37528400	3.56966500	-4.37333300
C	-0.59236700	-0.13866100	0.08779000
C	0.63652100	-0.69949900	0.38741500
C	-1.77205800	-0.91848600	0.24510800
C	0.66932000	-2.04169500	0.84668500
H	1.55652400	-0.13478400	0.28309800
C	-1.75918200	-2.24792400	0.70603800
C	-2.96719200	-0.22745800	-0.10371900
C	-0.48176600	-2.79873800	1.00643400
H	1.63123700	-2.48557100	1.08329800
C	-3.02520700	-2.88731700	0.82054400
C	-4.18504800	-0.87447400	0.01929500
H	-0.41157800	-3.82180000	1.36539500

C	-4.19128400	-2.21294700	0.48811900
H	-3.07571900	-3.91410700	1.17219000
H	-5.11610700	-0.38151900	-0.23826900
H	-5.14385500	-2.72417900	0.58568100

UB3LYP-D3/BSII(SMD)//B3LYP-D3/BSI

HF=-1559.3383153

^{1}E

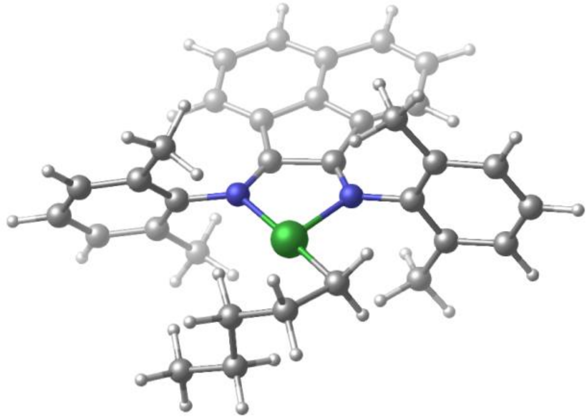

Zero-point correction= 0.598334 (Hartree/Particle)
Thermal correction to Energy= 0.632599
Thermal correction to Enthalpy= 0.633543
Thermal correction to Gibbs Free Energy= 0.532267
Sum of electronic and zero-point Energies= -1556.731923
Sum of electronic and thermal Energies= -1556.697659
Sum of electronic and thermal Enthalpies= -1556.696714
Sum of electronic and thermal Free Energies= -1556.797990

Ni	-2.27722700	3.63243800	-1.08386600
N	-0.75906800	2.31783800	-0.65720100
N	-3.41634900	2.24402900	-0.47321300
C	0.64014200	2.50134600	-0.85837700
C	1.29405900	1.83726600	-1.91553300
C	1.29220500	3.44153300	-0.03731700
C	2.65115600	2.11759000	-2.11240700
C	2.64989600	3.68032200	-0.26791700
C	3.32719100	3.02328600	-1.29527700
H	3.17624300	1.62651200	-2.92706900
H	3.17711200	4.38907700	0.36467000
H	4.37999900	3.22586900	-1.46693300
C	-4.84783700	2.27932600	-0.40608700
C	-5.57874200	1.85881200	-1.52832800
C	-5.44938800	2.76783500	0.76451400
C	-6.97417700	1.90989400	-1.44326000
C	-6.84727900	2.80093700	0.80438900
C	-7.60408700	2.37152400	-0.28671200
H	-7.56772900	1.58975100	-2.29511400
H	-7.34206100	3.17252900	1.69743600
H	-8.68833300	2.40649000	-0.23938400
C	0.54690000	4.12833800	1.08135700
H	0.22407200	3.41261500	1.84749600
H	1.17131300	4.88303900	1.56662900

H	-0.36319200	4.62522100	0.71780600
C	0.55400400	0.89341200	-2.83424600
H	-0.40815300	1.31454900	-3.14932200
H	1.14190400	0.68943800	-3.73290400
H	0.34409800	-0.06831000	-2.35090800
C	-4.59986500	3.23049900	1.92175300
H	-4.05737500	2.39471900	2.38210600
H	-3.84469000	3.95477600	1.58892200
H	-5.21014300	3.70060400	2.69710900
C	-4.86694600	1.36722300	-2.76427200
H	-4.10020800	2.08302200	-3.08767300
H	-4.35532000	0.41349000	-2.58103800
H	-5.56741900	1.21667000	-3.58968300
C	-2.76874100	1.18550900	-0.10903100
C	-1.26922100	1.22046100	-0.21539900
C	-1.35870700	5.56078000	-2.26419700
H	-0.61655600	6.35822000	-2.13346500
H	-0.89672500	4.77033500	-1.60733800
C	-3.65204900	4.87374400	-1.50101000
H	-4.35582500	4.98657000	-0.67204700
H	-4.17898500	4.50609600	-2.38910600
C	-2.73763500	6.06198200	-1.74569600
H	-3.14359900	6.77279400	-2.47860100
H	-2.58459500	6.61364800	-0.81185800
C	-1.35193000	5.07067400	-3.71810700
H	-2.07684600	4.25414500	-3.83872800
H	-1.70876900	5.89267300	-4.35236100
C	0.03294900	4.61012400	-4.18073200
H	0.00753700	4.26073300	-5.21791000
H	0.75956600	5.42874000	-4.12052700
H	0.40778400	3.79171200	-3.55593900
C	-3.15081200	-0.11589700	0.42392900
C	-4.34862000	-0.73381500	0.74145900
C	-1.92417500	-0.81089100	0.63470700
C	-4.30294200	-2.04870700	1.27061600
H	-5.29980200	-0.23457000	0.59414700
C	-1.86238700	-2.11272100	1.16444900
C	-0.76808100	-0.06493400	0.26760400
C	-3.10857600	-2.72356700	1.47990300
H	-5.23798700	-2.53974000	1.52101800
C	-0.56114000	-2.66613000	1.32565500
C	0.48472200	-0.62771700	0.43613500
H	-3.12140100	-3.72959600	1.89008400
C	0.56601300	-1.93850700	0.97252800

H	-0.45300100	-3.66876700	1.72988900
H	1.38667400	-0.08988200	0.16458500
H	1.54651500	-2.38424500	1.10706000

UB3LYP-D3/BSII(SMD)//B3LYP-D3/BSI

HF=-1559.3858533

^{3}E

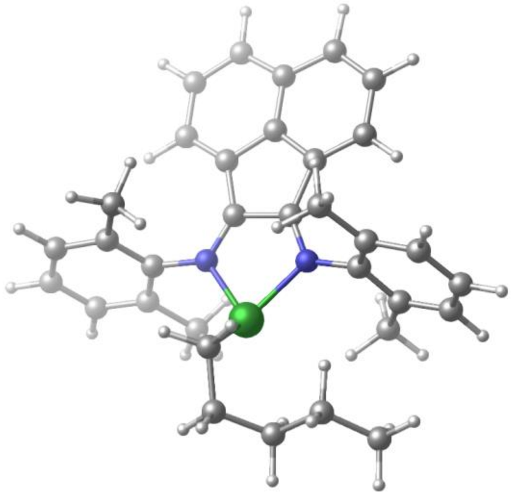

Zero-point correction= 0.596923 (Hartree/Particle)
Thermal correction to Energy= 0.631765
Thermal correction to Enthalpy= 0.632709
Thermal correction to Gibbs Free Energy= 0.527924
Sum of electronic and zero-point Energies= -1556.724707
Sum of electronic and thermal Energies= -1556.689865
Sum of electronic and thermal Enthalpies= -1556.688921
Sum of electronic and thermal Free Energies= -1556.793706

Ni	-1.46058100	3.28349300	-1.76721300
N	-0.19946100	1.94805500	-0.90164300
N	-2.89269800	1.94992400	-1.21983900
C	1.20899600	2.11840100	-0.76804500
C	1.98266700	2.00238600	-1.93910300
C	1.76285000	2.49104500	0.47302900
C	3.36139500	2.20038000	-1.83417100
C	3.14723400	2.69039700	0.52454300
C	3.94250800	2.53575400	-0.61073600
H	3.98079200	2.09645600	-2.72062300
H	3.60091100	2.98349900	1.46732300
H	5.01469500	2.69420500	-0.54576900
C	-4.29583800	2.10630200	-1.42220300
C	-4.77836900	1.94662100	-2.73452900
C	-5.11925600	2.51528900	-0.35515000
C	-6.14321400	2.14369800	-2.95735600
C	-6.47665300	2.71510600	-0.63119800
C	-6.98891500	2.52114800	-1.91410200
H	-6.54027900	2.01230700	-3.95981300
H	-7.13423900	3.03720400	0.17147700
H	-8.04593200	2.68059500	-2.10434700
C	0.90037300	2.70738700	1.69416300

H	1.44823200	3.26468900	2.45845200
H	-0.00596100	3.27304300	1.44830200
H	0.58073700	1.75935600	2.14378900
C	1.32846000	1.65669500	-3.25438300
H	0.82781500	0.68167900	-3.21896800
H	0.55959600	2.39708600	-3.52538100
H	2.05998200	1.63324600	-4.06616600
C	-4.55822200	2.76420500	1.02477400
H	-4.32409700	1.82772000	1.54574600
H	-3.63301600	3.35070800	0.97966000
H	-5.27576700	3.31263800	1.64047400
C	-3.83614700	1.57431700	-3.85196400
H	-3.03234900	2.31877000	-3.95471100
H	-3.35175300	0.60656100	-3.67532100
H	-4.35949300	1.52239800	-4.81011000
C	-2.37150200	0.98884200	-0.53574900
C	-0.86546300	0.98784400	-0.35604400
C	-2.49133500	5.75454400	-3.29095900
H	-2.41835800	6.52857100	-4.06615600
H	-2.15917700	4.81974100	-3.78753700
C	-1.51910700	5.10585800	-0.98154300
H	-0.65015700	5.21630600	-0.32315400
H	-2.43664600	5.13780200	-0.38317400
C	-1.52025200	6.09948200	-2.14371100
H	-1.78352000	7.09812800	-1.75723000
H	-0.50565300	6.18879500	-2.55219100
C	-3.95581400	5.59539000	-2.86331500
H	-4.04543400	4.77384200	-2.14004800
H	-4.27310000	6.50504900	-2.33502000
C	-4.89165400	5.32823800	-4.04539900
H	-5.92491400	5.19322300	-3.71043700
H	-4.87026300	6.15778900	-4.76164300
H	-4.59678500	4.41781700	-4.58218800
C	-0.52100700	-0.20382700	0.41465500
C	0.66044800	-0.76545300	0.86815700
C	-1.75265300	-0.87235100	0.66363600
C	0.59394800	-1.99542200	1.57067600
H	1.61711300	-0.28358300	0.69879100
C	-1.83680200	-2.09406900	1.35700400
C	-2.89000500	-0.20088200	0.13318700
C	-0.60734200	-2.64532800	1.81452200
H	1.51751400	-2.43894800	1.92899400
C	-3.14126800	-2.63974700	1.51628200
C	-4.14707500	-0.75681600	0.29998500

H	-0.61291500	-3.58560600	2.35879100
C	-4.25065600	-1.98538200	1.00027500
H	-3.26614300	-3.57954200	2.04694500
H	-5.03593100	-0.27204200	-0.08848600
H	-5.23393200	-2.42486300	1.13480800

UB3LYP-D3/BSII(SMD)//B3LYP-D3/BSI

HF=-1559.3874592

^{1}F

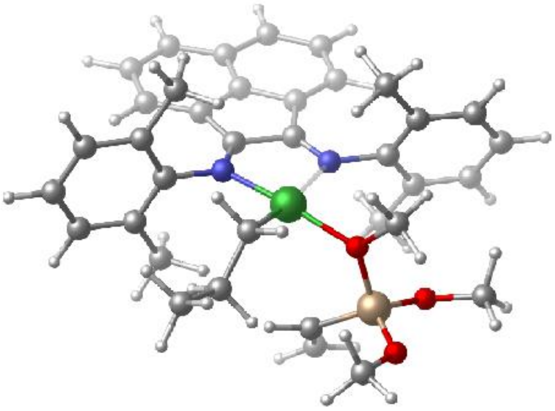

Zero-point correction= 0.717826 (Hartree/Particle)
Thermal correction to Energy= 0.763587
Thermal correction to Enthalpy= 0.764531
Thermal correction to Gibbs Free Energy= 0.638806
Sum of electronic and zero-point Energies= -2191.049821
Sum of electronic and thermal Energies= -2191.004060
Sum of electronic and thermal Enthalpies= -2191.003116
Sum of electronic and thermal Free Energies= -2191.128841

Ni	-2.56900100	3.78533000	-1.52736300
N	-0.99011000	2.39770800	-1.51135200
N	-3.45046800	2.42062000	-0.47629900
C	0.24385500	2.42901600	-2.23244300
C	0.17810600	2.21266300	-3.62215000
C	1.45135100	2.70118700	-1.56777800
C	1.36727800	2.28613700	-4.35460700
C	2.61678800	2.76141700	-2.34146900
C	2.58011600	2.56063700	-3.72082900
H	1.33944100	2.11549800	-5.42752600
H	3.56154300	2.97642200	-1.84936900
H	3.49584300	2.61572700	-4.30205200
C	-4.76697500	2.47811900	0.09283600
C	-5.84761200	2.01648800	-0.67629600
C	-4.91899100	3.00545100	1.38560300
C	-7.12671500	2.11145500	-0.11752600
C	-6.21678200	3.08299500	1.90303600
C	-7.31222300	2.64407100	1.15864700
H	-7.98126300	1.76401700	-0.69160300
H	-6.36497100	3.49328200	2.89823600
H	-8.31267600	2.71630500	1.57462800
C	1.49425100	2.89303100	-0.07302200
H	1.45294600	1.92882700	0.45048700
H	2.41649100	3.39720300	0.22711800

H	0.65205700	3.49445300	0.27679100
C	-1.13375900	1.85383300	-4.27874000
H	-1.94393900	2.52224800	-3.96224000
H	-1.05626900	1.89680700	-5.36872000
H	-1.44574200	0.83742800	-4.00557200
C	-3.71624500	3.43180300	2.19297500
H	-3.00039400	3.99500000	1.58479200
H	-4.01344600	4.05187900	3.04315500
H	-3.17899500	2.55990000	2.58929200
C	-5.61290900	1.40398000	-2.03530000
H	-4.96132100	2.03464900	-2.65192800
H	-5.11912000	0.42729700	-1.94678200
H	-6.55520100	1.25430400	-2.56887800
C	-2.70306000	1.39789500	-0.21451700
C	-1.33369900	1.36498500	-0.82696400
C	-1.05747300	5.93325700	0.41303700
H	-2.12118000	5.91500600	0.63819700
C	-0.18498000	5.80558900	1.42397400
H	0.88955700	5.82613800	1.25885000
C	-4.15538400	4.85391300	-1.79264800
H	-4.98537000	4.15139800	-1.92030200
H	-3.98652100	5.36583400	-2.75066400
C	-4.48142800	5.86257600	-0.69891000
H	-4.48094300	5.37041800	0.28046500
C	-5.85407200	6.52313900	-0.91079500
H	-6.64966800	5.76951300	-0.88656600
H	-6.06374800	7.26288800	-0.12939100
H	-5.90198000	7.03142700	-1.88129300
H	-3.70961800	6.64045100	-0.66448000
H	-0.51013900	5.67565300	2.45499400
Si	-0.49894000	6.22571000	-1.31961600
O	-0.68713400	7.73915300	-1.93009500
O	1.07986300	5.83585600	-1.43048600
O	-1.46558400	5.21623900	-2.31681300
C	-1.92243400	8.44510600	-2.05026000
H	-1.72770600	9.36417100	-2.60798200
H	-2.67222000	7.85194500	-2.59021200
H	-2.32060500	8.70832500	-1.06318800
C	1.98167200	6.19793100	-2.48087700
H	2.99419500	6.18093400	-2.07010400
H	1.92063600	5.46555100	-3.29294500
H	1.76496200	7.20116100	-2.86205500
C	-1.35614600	5.33408300	-3.75699700
H	-0.56442000	4.67275400	-4.11407400

H	-2.31372500	5.04326000	-4.19068800
H	-1.13318100	6.36993800	-4.02329200
C	-3.71863700	-1.68951700	1.83440100
C	-2.55631500	-2.41793000	1.62815800
C	-1.49426900	-1.88391300	0.84615000
C	-1.69736600	-0.59638300	0.31546500
C	-2.89233400	0.15208400	0.52492600
C	-3.91108200	-0.39530900	1.28661600
H	-0.04121600	-3.49938200	0.91960000
H	-4.51356400	-2.12143100	2.43430200
H	-2.45313600	-3.40741100	2.06504300
C	-0.25240700	-2.50533300	0.53518900
C	-0.73402000	0.07203000	-0.49138900
H	-4.83531500	0.14152800	1.46752400
C	0.46399300	-0.55674600	-0.78011100
C	0.68548400	-1.85547300	-0.25306400
H	1.22101000	-0.07806500	-1.39145200
H	1.62389800	-2.35306600	-0.47700800

UB3LYP-D3/BSII(SMD)//B3LYP-D3/BSI

HF=-2193.9537367

^{3}F

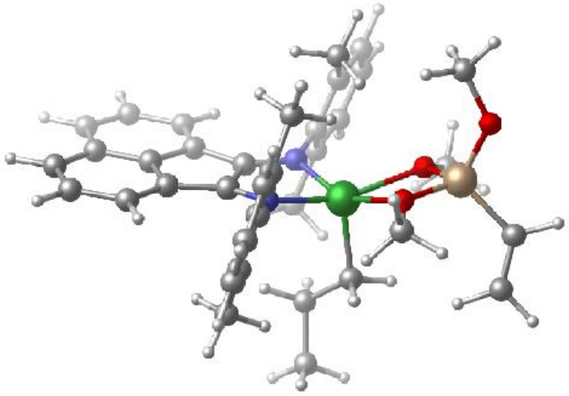

Ni	-3.02073300	3.92698600	-1.21070400
N	-1.19879500	2.93349400	-1.02377300
N	-3.74016300	2.03526300	-0.79346000
C	0.07395400	3.41939200	-1.45754600
C	0.26868700	3.44365700	-2.85593300
C	1.04571400	3.90755600	-0.56541000
C	1.46320900	3.97141800	-3.35123200
C	2.22642700	4.43242300	-1.11041700
C	2.43725700	4.47111100	-2.48624500
H	1.63088000	3.98421400	-4.42493000
H	2.98856800	4.81340200	-0.43571400
H	3.36097900	4.88064000	-2.88389800
C	-5.09795300	1.61029300	-0.91524300
C	-5.54585800	1.34574700	-2.22508200
C	-5.95576000	1.53721900	0.19762800
C	-6.88236300	0.97871000	-2.40515200
C	-7.28948100	1.17521000	-0.03342100
C	-7.75268500	0.89607200	-1.31859000
H	-7.24034400	0.75872700	-3.40743500
H	-7.96934900	1.11311000	0.81198500
H	-8.79011500	0.61464200	-1.47241300
C	0.84803400	3.91299500	0.93143300
H	1.81288400	3.88225100	1.44654500
H	0.33663700	4.82753100	1.25500200
H	0.25299700	3.06657800	1.28155000
C	-0.78564300	2.89232900	-3.78447700
H	-0.98729100	1.83263000	-3.58722500
H	-1.73492400	3.42608800	-3.65972400
H	-0.47641600	2.98678200	-4.82937600
C	-5.47383800	1.85687500	1.59142400
H	-5.20060800	2.91225900	1.68180100
H	-6.25355700	1.64729100	2.32868500

H	-4.58914400	1.27207400	1.86540000
C	-4.59673800	1.46436300	-3.39257500
H	-4.17955000	2.47779200	-3.45050700
H	-3.74749900	0.77682700	-3.29957200
H	-5.10461000	1.24672800	-4.33642200
C	-2.79436300	1.26429800	-0.37381000
C	-1.37416000	1.75819100	-0.51985900
C	-4.48006200	7.73372600	-2.39316000
H	-4.49938500	8.58288100	-3.07551400
C	-4.91076400	7.89976900	-1.13339000
H	-5.26927500	8.86062800	-0.76910700
C	-3.39081800	4.86390000	0.54532000
H	-4.49089600	4.86008800	0.60250000
H	-3.05751800	5.90165500	0.40351000
C	-2.76062800	4.24163500	1.78027800
H	-2.88770500	3.15003700	1.76445600
C	-3.32668600	4.79016900	3.10139400
H	-4.40455100	4.60085000	3.17844200
H	-2.84124500	4.32746100	3.96938100
H	-3.17851400	5.87446000	3.16799000
H	-1.68117200	4.41329900	1.75838600
H	-4.92233900	7.08607000	-0.41098600
Si	-3.88790400	6.11127700	-3.02372600
O	-3.94730200	6.14493100	-4.65391100
O	-4.68284300	4.79266400	-2.35267000
O	-2.39059400	5.61152600	-2.44133800
C	-3.55168100	5.09428100	-5.53966200
H	-3.83773200	5.38961500	-6.55128300
H	-2.46683300	4.94420700	-5.50151600
H	-4.05425200	4.15278100	-5.28622500
C	-6.10863000	4.65990800	-2.18711700
H	-6.58483600	5.64431600	-2.17413200
H	-6.51457500	4.05420300	-3.00016400
H	-6.28278800	4.15106600	-1.23814900
C	-1.25264900	6.46438000	-2.20215700
H	-0.78292300	6.13056100	-1.27616700
H	-0.54314100	6.35894200	-3.02559500
H	-1.56754000	7.50704400	-2.09838200
C	-3.20219700	-2.35332200	0.86343800
C	-1.85269200	-2.64728300	0.98465000
C	-0.86783100	-1.67072600	0.66660800
C	-1.34550800	-0.41590500	0.24506400
C	-2.72989300	-0.11230400	0.12125100
C	-3.66557300	-1.08522200	0.42672300

H	0.97040000	-2.76999300	1.04055900
H	-3.93425200	-3.11670500	1.10803200
H	-1.54034900	-3.63210300	1.32100700
C	0.54585100	-1.82353600	0.71718400
C	-0.48970800	0.65826600	-0.12753700
H	-4.72936900	-0.89618600	0.33728700
C	0.88161800	0.47721200	-0.07771200
C	1.38155500	-0.77885600	0.35282600
H	1.56799700	1.26708300	-0.36033100
H	2.45684300	-0.92179800	0.39557300

UB3LYP-D3/BSII(SMD)//B3LYP-D3/BSI

HF=-2193.954199

[1]G

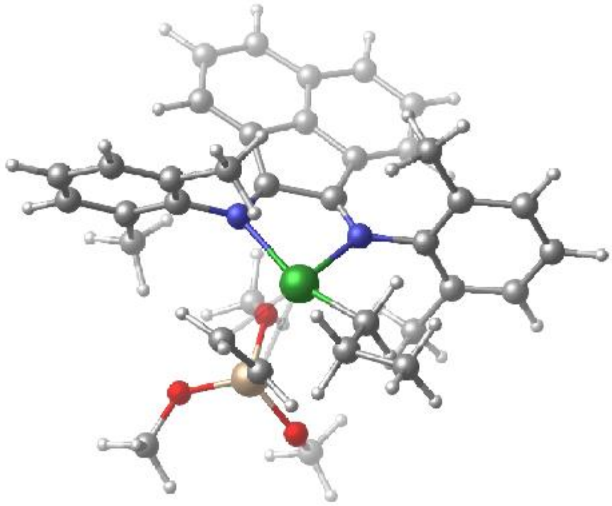

Zero-point correction= 0.717295 (Hartree/Particle)
Thermal correction to Energy= 0.763221
Thermal correction to Enthalpy= 0.764165
Thermal correction to Gibbs Free Energy= 0.637773
Sum of electronic and zero-point Energies= -2191.039540
Sum of electronic and thermal Energies= -2190.993614
Sum of electronic and thermal Enthalpies= -2190.992670
Sum of electronic and thermal Free Energies= -2191.119062

Ni	-2.21148300	4.10696100	-0.76323500
N	-0.82969900	2.51776600	-1.00559900
N	-3.43497300	2.61171100	-0.30575700
C	0.59305800	2.60369300	-1.10359800
C	1.29154800	2.28536700	-2.28530500
C	1.24778800	3.11655200	0.03681400
C	2.68381800	2.44918200	-2.27641600
C	2.63674800	3.25632200	-0.00273700
C	3.35473900	2.92183300	-1.15070100
H	3.24310900	2.20683700	-3.17598700
H	3.15604300	3.63206300	0.87461800
H	4.43424500	3.03725300	-1.17029600
C	-4.76616400	2.67135900	0.22740100
C	-5.85716900	2.92446600	-0.61970700
C	-4.90761100	2.50155900	1.61732700
C	-7.12874200	2.97707700	-0.03619500
C	-6.19640300	2.57088400	2.15586900
C	-7.30096700	2.80195900	1.33669700
H	-7.99081000	3.16228600	-0.67127800
H	-6.32952700	2.44239100	3.22653100
H	-8.29605700	2.85079500	1.76865600
C	0.45744100	3.49191000	1.26695700

H	-0.10965700	2.64235600	1.66553500
H	1.11311600	3.86407200	2.05883400
H	-0.27526200	4.28172500	1.04157700
C	0.59210500	1.83891700	-3.54699800
H	-0.14313600	1.05205500	-3.36351600
H	0.06479500	2.67143200	-4.02405500
H	1.31892700	1.45264100	-4.26738200
C	-3.70162900	2.23707300	2.48654700
H	-2.90113700	2.96239400	2.29334800
H	-3.96312000	2.28968500	3.54662200
H	-3.28106100	1.24112800	2.29583700
C	-5.66686000	3.13290100	-2.10035700
H	-5.17803200	4.09122600	-2.30986800
H	-5.02972900	2.36231700	-2.54346500
H	-6.63021000	3.12989200	-2.61843500
C	-2.92889400	1.46401700	-0.60910600
C	-1.46121900	1.39906200	-0.94707400
C	-2.08664300	5.38172400	-2.44767800
H	-2.78301000	6.20273600	-2.28486000
C	-0.87470500	5.43661900	-1.78817600
H	-0.04213400	4.79619300	-2.06493700
C	-3.39674700	5.38978800	0.07614800
H	-3.68333900	4.82000000	0.96791500
H	-4.28797000	5.52316300	-0.54780400
C	-2.75031400	6.70615500	0.47650000
H	-1.79790800	6.51553000	0.99104400
C	-3.67125600	7.51017000	1.41391900
H	-3.88970400	6.94721100	2.32862500
H	-3.20173600	8.45734200	1.70197400
H	-4.62460400	7.73724100	0.92356800
H	-2.51916900	7.31881600	-0.40331100
H	-0.62712100	6.26382600	-1.12808500
Si	-2.52866000	4.32181300	-3.92933800
O	-3.93198500	5.01598800	-4.43704300
O	-1.35975100	4.32080100	-5.09390900
O	-2.69421900	2.73903400	-3.49855000
C	-4.77247300	4.49280000	-5.46649300
H	-5.46163800	5.28350000	-5.77295400
H	-4.19304800	4.17218200	-6.34145100
H	-5.35466000	3.64304000	-5.08897700
C	-0.90043200	5.49367400	-5.77192800
H	-0.38753400	6.17055200	-5.07702200
H	-0.19451000	5.17720200	-6.54341000
H	-1.73114200	6.02985400	-6.24567000

C	-2.76393900	1.63963800	-4.40636400
H	-2.00834200	1.72267900	-5.19406100
H	-2.58502800	0.72361800	-3.83497700
H	-3.75906200	1.57687200	-4.86325400
C	-4.76833900	-1.89975900	-0.35911500
C	-3.66774400	-2.71226400	-0.58715900
C	-2.38352000	-2.14579400	-0.82021700
C	-2.31171000	-0.73977100	-0.81812800
C	-3.43925100	0.09426100	-0.57341000
C	-4.67514100	-0.48447500	-0.33960700
H	-1.15923300	-3.92626700	-1.06046100
H	-5.73626000	-2.35795000	-0.18144700
H	-3.78237000	-3.79276600	-0.58425200
C	-1.16228800	-2.83983800	-1.04580800
C	-1.10123200	-0.02069300	-1.02971100
H	-5.55500300	0.11756000	-0.14402800
C	0.07296600	-0.72419100	-1.23630800
C	0.01987200	-2.14181500	-1.24003800
H	1.01716600	-0.21529300	-1.39238500
H	0.94001400	-2.69419600	-1.40311400

UB3LYP-D3/BSII(SMD)//B3LYP-D3/BSI

HF=-2193.9416353

[3]G

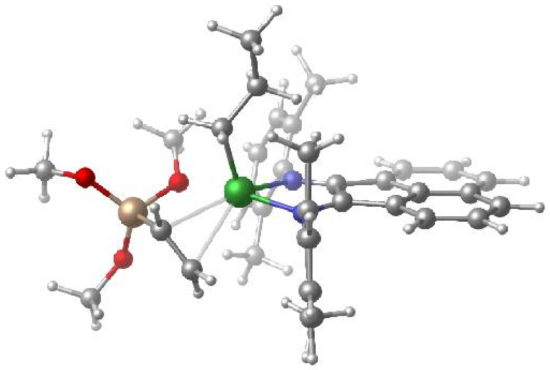

Zero-point correction= 0.715377 (Hartree/Particle)
Thermal correction to Energy= 0.762054
Thermal correction to Enthalpy= 0.762998
Thermal correction to Gibbs Free Energy= 0.632934
Sum of electronic and zero-point Energies= -2191.047720
Sum of electronic and thermal Energies= -2191.001043
Sum of electronic and thermal Enthalpies= -2191.000099
Sum of electronic and thermal Free Energies= -2191.130163

Ni	-3.02073300	3.92698600	-1.21070400
N	-1.19879500	2.93349400	-1.02377300
N	-3.74016300	2.03526300	-0.79346000
C	0.07395400	3.41939200	-1.45754600
C	0.26868700	3.44365700	-2.85593300
C	1.04571400	3.90755600	-0.56541000
C	1.46320900	3.97141800	-3.35123200
C	2.22642700	4.43242300	-1.11041700
C	2.43725700	4.47111100	-2.48624500
H	1.63088000	3.98421400	-4.42493000
H	2.98856800	4.81340200	-0.43571400
H	3.36097900	4.88064000	-2.88389800
C	-5.09795300	1.61029300	-0.91524300
C	-5.54585800	1.34574700	-2.22508200
C	-5.95576000	1.53721900	0.19762800
C	-6.88236300	0.97871000	-2.40515200
C	-7.28948100	1.17521000	-0.03342100
C	-7.75268500	0.89607200	-1.31859000
H	-7.24034400	0.75872700	-3.40743500
H	-7.96934900	1.11311000	0.81198500
H	-8.79011500	0.61464200	-1.47241300
C	0.84803400	3.91299500	0.93143300
H	1.81288400	3.88225100	1.44654500
H	0.33663700	4.82753100	1.25500200
H	0.25299700	3.06657800	1.28155000

C	-0.78564300	2.89232900	-3.78447700
H	-0.98729100	1.83263000	-3.58722500
H	-1.73492400	3.42608800	-3.65972400
H	-0.47641600	2.98678200	-4.82937600
C	-5.47383800	1.85687500	1.59142400
H	-5.20060800	2.91225900	1.68180100
H	-6.25355700	1.64729100	2.32868500
H	-4.58914400	1.27207400	1.86540000
C	-4.59673800	1.46436300	-3.39257500
H	-4.17955000	2.47779200	-3.45050700
H	-3.74749900	0.77682700	-3.29957200
H	-5.10461000	1.24672800	-4.33642200
C	-2.79436300	1.26429800	-0.37381000
C	-1.37416000	1.75819100	-0.51985900
C	-4.48006200	7.73372600	-2.39316000
H	-4.49938500	8.58288100	-3.07551400
C	-4.91076400	7.89976900	-1.13339000
H	-5.26927500	8.86062800	-0.76910700
C	-3.39081800	4.86390000	0.54532000
H	-4.49089600	4.86008800	0.60250000
H	-3.05751800	5.90165500	0.40351000
C	-2.76062800	4.24163500	1.78027800
H	-2.88770500	3.15003700	1.76445600
C	-3.32668600	4.79016900	3.10139400
H	-4.40455100	4.60085000	3.17844200
H	-2.84124500	4.32746100	3.96938100
H	-3.17851400	5.87446000	3.16799000
H	-1.68117200	4.41329900	1.75838600
H	-4.92233900	7.08607000	-0.41098600
Si	-3.88790400	6.11127700	-3.02372600
O	-3.94730200	6.14493100	-4.65391100
O	-4.68284300	4.79266400	-2.35267000
O	-2.39059400	5.61152600	-2.44133800
C	-3.55168100	5.09428100	-5.53966200
H	-3.83773200	5.38961500	-6.55128300
H	-2.46683300	4.94420700	-5.50151600
H	-4.05425200	4.15278100	-5.28622500
C	-6.10863000	4.65990800	-2.18711700
H	-6.58483600	5.64431600	-2.17413200
H	-6.51457500	4.05420300	-3.00016400
H	-6.28278800	4.15106600	-1.23814900
C	-1.25264900	6.46438000	-2.20215700
H	-0.78292300	6.13056100	-1.27616700
H	-0.54314100	6.35894200	-3.02559500

H	-1.56754000	7.50704400	-2.09838200
C	-3.20219700	-2.35332200	0.86343800
C	-1.85269200	-2.64728300	0.98465000
C	-0.86783100	-1.67072600	0.66660800
C	-1.34550800	-0.41590500	0.24506400
C	-2.72989300	-0.11230400	0.12125100
C	-3.66557300	-1.08522200	0.42672300
H	0.97040000	-2.76999300	1.04055900
H	-3.93425200	-3.11670500	1.10803200
H	-1.54034900	-3.63210300	1.32100700
C	0.54585100	-1.82353600	0.71718400
C	-0.48970800	0.65826600	-0.12753700
H	-4.72936900	-0.89618600	0.33728700
C	0.88161800	0.47721200	-0.07771200
C	1.38155500	-0.77885600	0.35282600
H	1.56799700	1.26708300	-0.36033100
H	2.45684300	-0.92179800	0.39557300

UB3LYP-D3/BSII(SMD)//B3LYP-D3/BSI

HF=-2193.940966

[1]TS3

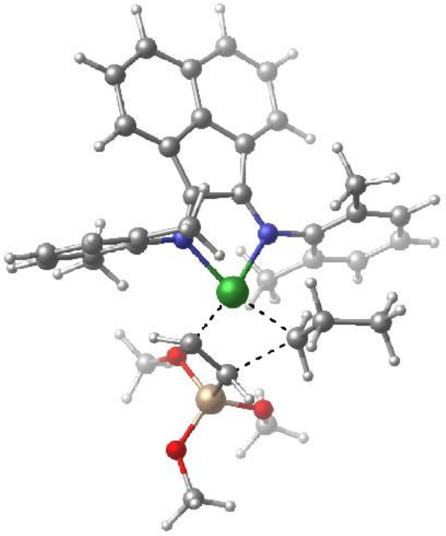

Zero-point correction= 0.716940 (Hartree/Particle)
Thermal correction to Energy= 0.762483
Thermal correction to Enthalpy= 0.763428
Thermal correction to Gibbs Free Energy= 0.635902
Sum of electronic and zero-point Energies= -2191.022626
Sum of electronic and thermal Energies= -2190.977083
Sum of electronic and thermal Enthalpies= -2190.976139
Sum of electronic and thermal Free Energies= -2191.103664

Ni	-1.96498600	3.74190700	-1.19265900
N	-0.74573700	2.22940000	-0.93392400
N	-3.35647000	2.36782800	-0.42084300
C	0.65947800	2.21165200	-1.22279600
C	1.08963600	1.70906700	-2.46187800
C	1.53859900	2.74422400	-0.26707100
C	2.46493500	1.72386600	-2.72068200
C	2.90492000	2.73597900	-0.56754100
C	3.36657400	2.22635100	-1.78156800
H	2.82682100	1.34257100	-3.67168800
H	3.60819700	3.13719000	0.15715000
H	4.43009200	2.23035800	-2.00085800
C	-4.75485400	2.52556600	-0.16384300
C	-5.62746000	2.54684400	-1.26823200
C	-5.20230400	2.72278900	1.15659500
C	-6.98719900	2.76520000	-1.02225100
C	-6.57069300	2.94250700	1.35154700
C	-7.45762300	2.96460000	0.27486500
H	-7.67911100	2.77801000	-1.85994600
H	-6.93956600	3.10051200	2.36138500
H	-8.51520600	3.13977100	0.44798400
C	1.00557700	3.29121600	1.03400400

H	0.57308800	2.49746000	1.65669500
H	1.79526000	3.77817300	1.61229000
H	0.20574900	4.02184600	0.85428700
C	0.09161500	1.18021300	-3.46396300
H	-0.36109600	0.24107200	-3.11995500
H	-0.72523900	1.89444400	-3.62284300
H	0.57280100	0.98325000	-4.42595200
C	-4.24858200	2.67915800	2.32688700
H	-3.33689300	3.25233000	2.13032400
H	-4.71760300	3.08624200	3.22647900
H	-3.93989700	1.64997800	2.55013900
C	-5.10179000	2.30514400	-2.66100000
H	-4.27729900	2.98056500	-2.91176300
H	-4.71000900	1.28519600	-2.76573100
H	-5.89323300	2.43705900	-3.40440900
C	-2.78066700	1.23943200	-0.19243500
C	-1.30955100	1.16647800	-0.46906900
C	-1.76733200	5.57553900	-2.41876300
H	-1.76103900	6.59778100	-2.04935600
C	-0.61177100	4.80463800	-2.09528700
H	-0.20412600	4.11985900	-2.83557200
C	-3.32223200	5.34372400	-1.00638900
H	-4.10865300	4.59031300	-1.05473300
H	-3.71817200	6.18574000	-1.56440000
C	-2.92567500	5.75696800	0.40613100
H	-2.49572900	4.91804300	0.97003700
C	-4.14133800	6.30209900	1.17726700
H	-4.92585300	5.54217200	1.25549600
H	-3.85409500	6.60596900	2.18987100
H	-4.56599000	7.17466300	0.66824700
H	-2.14063300	6.52250200	0.35712800
H	0.12700400	5.20686500	-1.40037200
Si	-2.57731700	5.28115300	-4.08403400
O	-4.15479100	5.73328000	-3.92097800
O	-1.85578300	6.08592500	-5.32489300
O	-2.37284000	3.67399200	-4.39385900
C	-5.14378900	5.57474200	-4.93849000
H	-6.00341100	6.19584100	-4.67392000
H	-4.77106800	5.88858400	-5.92172600
H	-5.46873000	4.52832100	-4.99719600
C	-1.94741000	7.49295100	-5.55494100
H	-1.25640100	8.03661600	-4.89903800
H	-1.66639600	7.68246800	-6.59394400
H	-2.96622000	7.86398500	-5.38754000

C	-2.50472900	3.07191500	-5.68462500
H	-2.06161900	3.70223900	-6.46244900
H	-1.98309700	2.11134100	-5.66167200
H	-3.56130300	2.89312000	-5.91885200
C	-4.41073600	-1.99559500	1.09585700
C	-3.25883600	-2.76777000	1.11979400
C	-2.00871000	-2.21820000	0.71992100
C	-2.02251800	-0.87232600	0.30886500
C	-3.20346800	-0.07727900	0.28925600
C	-4.40554700	-0.63808300	0.68315500
H	-0.67602300	-3.91382000	0.99449100
H	-5.35160300	-2.43995300	1.40522000
H	-3.30767300	-3.80286100	1.44654000
C	-0.74628400	-2.87412600	0.68678700
C	-0.85615300	-0.18068300	-0.12825100
H	-5.32507600	-0.06336200	0.68322000
C	0.35854300	-0.84409400	-0.15097500
C	0.39039400	-2.19985700	0.26590500
H	1.26707000	-0.35173400	-0.47884400
H	1.34075800	-2.72422200	0.25093400

UB3LYP-D3/BSII(SMD)//B3LYP-D3/BSI

HF=-2193.9225937

^{3}TS3

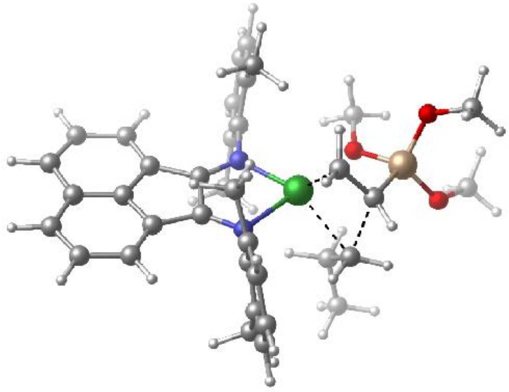

Zero-point correction= 0.715245 (Hartree/Particle)
Thermal correction to Energy= 0.762111
Thermal correction to Enthalpy= 0.763055
Thermal correction to Gibbs Free Energy= 0.631819
Sum of electronic and zero-point Energies= -2191.035778
Sum of electronic and thermal Energies= -2190.988913
Sum of electronic and thermal Enthalpies= -2190.987968
Sum of electronic and thermal Free Energies= -2191.119205

Ni	-2.73328600	4.03558600	-0.90395900
N	-1.08604900	2.79406600	-0.86909300
N	-3.66549800	2.21464700	-0.25586000
C	0.25717700	3.15060400	-1.20977400
C	0.59172600	3.23745200	-2.57148000
C	1.17199100	3.45274700	-0.18524900
C	1.89412900	3.62745800	-2.90088100
C	2.46201300	3.84505800	-0.56131300
C	2.82383900	3.93097000	-1.90662500
H	2.17552600	3.69707000	-3.94816200
H	3.18625900	4.08621700	0.21211500
H	3.82951900	4.23708100	-2.17877600
C	-5.06414000	1.93973400	-0.12460700
C	-5.76528700	1.47103400	-1.25587200
C	-5.70185700	2.17306000	1.10667900
C	-7.14990100	1.30851500	-1.14210100
C	-7.08865900	1.99046900	1.17247000
C	-7.81225100	1.57687200	0.05616400
H	-7.71078600	0.95869900	-2.00476800
H	-7.59827300	2.16816300	2.11596100
H	-8.88865500	1.44964000	0.12204300
C	0.76739400	3.36319700	1.26635900
H	0.51531700	2.33470300	1.55279300
H	1.57583700	3.70169200	1.91979900
H	-0.11571900	3.97975000	1.46992600

C	-0.43660400	2.92214700	-3.62856900
H	-0.75751800	1.87406500	-3.57894400
H	-1.33577600	3.53391600	-3.48964100
H	-0.04288900	3.10716600	-4.63152000
C	-4.91537500	2.50541200	2.35309100
H	-3.97026500	3.00221300	2.12713400
H	-5.49149800	3.14674900	3.02715500
H	-4.67045500	1.58853600	2.90561300
C	-5.04674000	1.07716700	-2.52654900
H	-4.21707000	1.74961400	-2.76180900
H	-4.62527600	0.06765300	-2.43131800
H	-5.73388800	1.06635200	-3.37739000
C	-2.80717500	1.25555700	-0.22145400
C	-1.37144900	1.58162200	-0.52942700
C	-4.21048700	5.65295000	-1.59410000
H	-5.23099200	5.89426000	-1.31280000
C	-4.03252000	4.51064000	-2.45067900
H	-4.86314900	3.82162100	-2.56952700
C	-3.99635800	5.46112700	0.54467100
H	-4.75894900	4.71094600	0.75315900
H	-4.42203700	6.46054500	0.58466700
C	-2.76069300	5.38792200	1.43681300
H	-2.35736900	4.36621200	1.49692800
C	-3.10019500	5.85526200	2.86797100
H	-3.88625700	5.23546500	3.31160200
H	-2.21478700	5.79691200	3.51150000
H	-3.44974900	6.89313200	2.85967800
H	-1.96368500	6.01967300	1.03137400
H	-3.35979100	4.57729200	-3.30544800
Si	-2.91094100	6.96088700	-1.81532700
O	-3.17630800	8.19037500	-0.76140400
O	-2.63502600	7.50866300	-3.34039500
O	-1.57098000	6.01695300	-1.45368100
C	-2.41944600	9.40094400	-0.67847100
H	-3.05874800	10.16921300	-0.23652000
H	-1.54359200	9.25519600	-0.03501900
H	-2.08621300	9.73980300	-1.66683200
C	-3.54114000	8.33184200	-4.08140000
H	-4.42576300	7.75825300	-4.38352200
H	-3.86120700	9.20443900	-3.49872400
H	-3.02049800	8.67671200	-4.97776300
C	-0.22995500	6.39952600	-1.81554700
H	-0.09361400	7.47942200	-1.69490700
H	0.46136500	5.87302900	-1.15856600

H	-0.02892700	6.12479500	-2.85354100
C	-3.56680900	-2.43284500	0.55622400
C	-2.26890100	-2.90531900	0.43330500
C	-1.20459500	-2.02073700	0.10197400
C	-1.55153300	-0.66971200	-0.08403700
C	-2.88276000	-0.18484100	0.03852700
C	-3.89891600	-1.06689900	0.36028200
H	0.49586100	-3.37519100	0.06439900
H	-4.36110800	-3.12844900	0.80867500
H	-2.05907800	-3.95999500	0.58879300
C	0.17002600	-2.34701000	-0.06714200
C	-0.60503500	0.33909500	-0.42132200
H	-4.92652300	-0.73503600	0.45899000
C	0.72546600	-0.00852700	-0.58296400
C	1.09305700	-1.36649200	-0.39908200
H	1.47718500	0.72872500	-0.84082100
H	2.13557600	-1.64236500	-0.52368000

UB3LYP-D3/BSII(SMD)//B3LYP-D3/BSI

HF=-2193.9066338

^{1}H

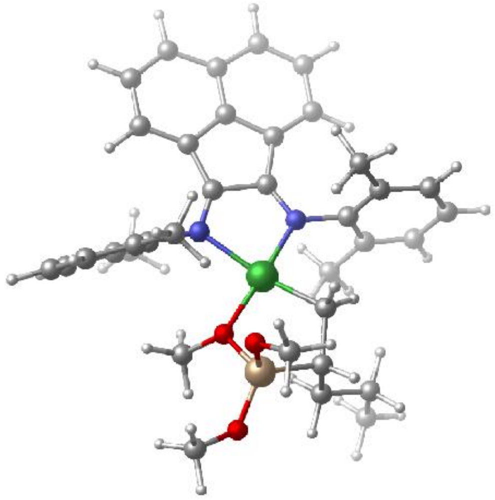

Zero-point correction= 0.720138 (Hartree/Particle)
Thermal correction to Energy= 0.765036
Thermal correction to Enthalpy= 0.765981
Thermal correction to Gibbs Free Energy= 0.639909
Sum of electronic and zero-point Energies= -2191.071589
Sum of electronic and thermal Energies= -2191.026691
Sum of electronic and thermal Enthalpies= -2191.025747
Sum of electronic and thermal Free Energies= -2191.151819

Ni	-2.60989400	3.37570100	-1.30732400
N	-1.11156600	2.20213200	-0.94181900
N	-3.68820000	1.87483600	-0.33705900
C	0.27047900	2.44647600	-1.24116800
C	0.83460200	1.86195900	-2.38625100
C	0.98206800	3.31552000	-0.39670300
C	2.17911900	2.14137000	-2.65654600
C	2.32283600	3.56592800	-0.70600000
C	2.91987600	2.97957000	-1.82265900
H	2.64272100	1.70213200	-3.53563800
H	2.89687500	4.23209100	-0.06789900
H	3.96117700	3.18651600	-2.05075400
C	-5.09632600	1.80159300	-0.11288200
C	-5.93044500	1.68803400	-1.24093300
C	-5.60260600	1.95243100	1.19211900
C	-7.31282200	1.70597100	-1.03029400
C	-6.99303500	1.97487900	1.35055500
C	-7.84354200	1.85145400	0.25215500
H	-7.97691200	1.60662500	-1.88463100
H	-7.40788500	2.09858300	2.34728800
H	-8.91987000	1.87094500	0.39510300
C	0.29682500	3.96155300	0.78079800
H	-0.02709300	3.21832900	1.52014600

H	0.95820600	4.67272700	1.28245600
H	-0.60359900	4.49942000	0.45411300
C	0.01217900	0.96951400	-3.28353300
H	-0.22125000	0.01435000	-2.79591400
H	-0.94323300	1.44168000	-3.54209200
H	0.54675700	0.74673900	-4.21064300
C	-4.68240100	2.11599900	2.37911400
H	-3.88404700	2.84078800	2.17688100
H	-5.23665300	2.45884500	3.25687700
H	-4.19259200	1.17138100	2.64553500
C	-5.34373100	1.54848400	-2.62431700
H	-4.81820500	2.46285700	-2.92391000
H	-4.62303400	0.72357100	-2.67534800
H	-6.12764900	1.35667100	-3.36228400
C	-2.89952700	0.90446500	-0.03142300
C	-1.44366700	1.11060900	-0.33179900
C	-2.11866400	5.93629800	-2.68474800
H	-1.70786600	6.39061000	-3.59989900
C	-1.56266000	4.51412000	-2.50709300
H	-1.59154900	3.93553800	-3.44334100
C	-1.78847900	6.84666700	-1.47743500
H	-2.13729000	6.34832900	-0.55898400
H	-2.34999700	7.78686900	-1.55158100
C	-0.29505600	7.17450300	-1.32846800
H	0.29335200	6.24969600	-1.27887400
C	-0.00783600	8.02050700	-0.08398900
H	-0.30189200	7.48760800	0.82953500
H	1.05763300	8.26140800	-0.00213700
H	-0.56327300	8.96559600	-0.10876300
H	0.04407000	7.70622700	-2.22805800
H	-0.53172400	4.52636400	-2.15189000
Si	-3.94594000	5.64723200	-2.81578400
O	-4.86217500	6.98534000	-2.58969100
O	-4.44702000	4.72466000	-4.08393300
O	-4.15926300	4.58409500	-1.48660600
C	-6.26242000	7.11080400	-2.86072700
H	-6.46159900	8.15022500	-3.13176600
H	-6.57314400	6.45591100	-3.68257300
H	-6.84124200	6.85880700	-1.96432600
C	-4.15578000	5.01086600	-5.45489800
H	-3.07478000	5.10194300	-5.62095200
H	-4.53478800	4.18007900	-6.05462000
H	-4.64742800	5.93519900	-5.78139300
C	-4.98440600	4.93403900	-0.34928200

H	-5.98177500	4.50981200	-0.48262200
H	-4.52564300	4.51069200	0.54339300
H	-5.03688100	6.02095500	-0.25474800
C	-3.82737100	-2.47443700	1.54818400
C	-2.53787800	-2.97616600	1.64383100
C	-1.42629000	-2.21114900	1.19212600
C	-1.71861500	-0.94371600	0.65464300
C	-3.04100800	-0.42514400	0.56358600
C	-4.10318100	-1.19072100	1.01067000
H	0.23119000	-3.54935900	1.62577700
H	-4.65766900	-3.07960600	1.89847000
H	-2.37089100	-3.96284100	2.06701400
C	-0.05297800	-2.58229700	1.22005900
C	-0.71845700	-0.06359000	0.14799800
H	-5.12331200	-0.82642200	0.95873800
C	0.60988700	-0.45248400	0.18710800
C	0.92190300	-1.72327900	0.73445500
H	1.39915000	0.18749000	-0.19045400
H	1.96228100	-2.03109200	0.76874500

UB3LYP-D3/BSII(SMD)//B3LYP-D3/BSI

HF=-2193.9768404

^{3}H

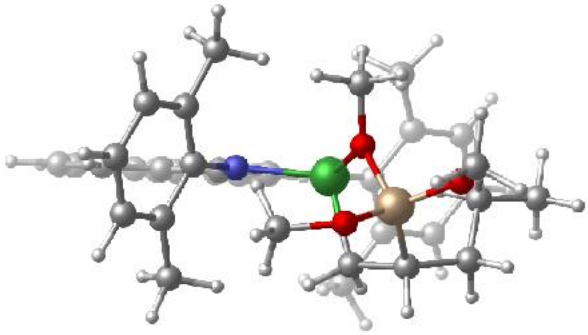

Zero-point correction= 0.718050 (Hartree/Particle)
Thermal correction to Energy= 0.763517
Thermal correction to Enthalpy= 0.764461
Thermal correction to Gibbs Free Energy= 0.636712
Sum of electronic and zero-point Energies= -2191.065410
Sum of electronic and thermal Energies= -2191.019943
Sum of electronic and thermal Enthalpies= -2191.018999
Sum of electronic and thermal Free Energies= -2191.146748

Ni	-2.63037700	3.72982400	0.33954500
N	-0.99106000	2.68242200	-0.33172400
N	-3.49684400	1.86317700	0.23787000
C	0.29937700	3.23676800	-0.58517800
C	0.65376900	3.62522400	-1.89064800
C	1.14344700	3.45066100	0.52055400
C	1.88447500	4.27016500	-2.06267800
C	2.36298800	4.09890100	0.30027000
C	2.72952400	4.51442700	-0.97998700
H	2.17686200	4.58691000	-3.06020300
H	3.03053500	4.27089700	1.14040500
H	3.67599800	5.02349300	-1.13520500
C	-4.86007200	1.58584500	0.54057500
C	-5.79787700	1.36085200	-0.48731800
C	-5.23677800	1.66659000	1.89723000
C	-7.13400800	1.17028500	-0.11144600
C	-6.57711900	1.45211800	2.22270600
C	-7.52207700	1.20266100	1.22645600
H	-7.87722700	1.00194300	-0.88618600
H	-6.88272500	1.49234100	3.26450200
H	-8.56353500	1.04973000	1.49278300
C	0.76669000	2.92074400	1.88256200
H	0.96030600	1.84191900	1.94665300
H	1.34296800	3.40770100	2.67436000
H	-0.29903500	3.05886500	2.09148300
C	-0.24894900	3.34200200	-3.06714500

H	-0.19914600	2.28433800	-3.35643500
H	-1.29596900	3.56135300	-2.83823700
H	0.04435600	3.93550800	-3.93731800
C	-4.20875600	1.98018900	2.95662600
H	-3.76198100	2.97203800	2.78918200
H	-4.65921800	1.98741100	3.95273800
H	-3.38596300	1.25587500	2.95888500
C	-5.40593900	1.34239000	-1.94610300
H	-4.61569400	2.06713900	-2.16481600
H	-5.03538500	0.35649000	-2.25302100
H	-6.26657100	1.57973800	-2.57757800
C	-2.69331100	0.99780400	-0.28566600
C	-1.29291500	1.45771200	-0.59975900
C	-3.90231700	6.17210000	-0.68770100
H	-3.96904600	6.87457400	-1.53604700
C	-3.41024500	4.80948000	-1.20690900
H	-4.22431700	4.22164200	-1.64838700
C	-5.30808500	6.10282700	-0.04151800
H	-6.00358400	5.70272200	-0.79402600
H	-5.65077700	7.11764700	0.19475300
C	-5.40899000	5.24161000	1.22425600
H	-5.02220300	4.23446100	1.00752600
C	-6.84425500	5.09956900	1.73659300
H	-7.48216900	4.62303800	0.98334800
H	-6.88405600	4.48356000	2.64116100
H	-7.27371600	6.07978500	1.97409100
H	-4.77432000	5.66487500	2.01339600
H	-2.60655400	4.91827200	-1.94248800
Si	-2.60868800	6.87949400	0.46438700
O	-3.21229700	7.94079100	1.54796200
O	-1.28188800	7.56121500	-0.22523000
O	-2.09475200	5.47533400	1.31743900
C	-2.64658500	9.17229100	2.00812700
H	-3.41947100	9.94414100	1.95903000
H	-2.32642000	9.05647600	3.04965800
H	-1.79063700	9.47449800	1.39709700
C	-0.25127300	6.86164800	-0.92289100
H	0.16966700	6.05631600	-0.31144600
H	-0.62751100	6.43251600	-1.85928700
H	0.53885300	7.57836700	-1.15914600
C	-1.34514100	5.60047100	2.54071800
H	-1.66618800	6.49621200	3.07893100
H	-1.54933000	4.72229600	3.15726900
H	-0.27535200	5.65608900	2.31943700

C	-3.42592600	-2.71631700	-0.96132900
C	-2.17244500	-3.05309800	-1.44978100
C	-1.14599500	-2.07234100	-1.54555300
C	-1.48501400	-0.77167600	-1.12881400
C	-2.77355200	-0.42148300	-0.63533900
C	-3.74971100	-1.39864800	-0.54652100
H	0.50193800	-3.24183200	-2.34749600
H	-4.19098600	-3.48345500	-0.89386400
H	-1.96801300	-4.07405700	-1.76008600
C	0.18671300	-2.25890000	-2.00837400
C	-0.56404400	0.31253200	-1.14308900
H	-4.74273100	-1.17403300	-0.17289400
C	0.72741600	0.10180400	-1.59330600
C	1.08369100	-1.20105200	-2.02737900
H	1.45632400	0.90452500	-1.61794000
H	2.09483200	-1.37121300	-2.38374000

UB3LYP-D3/BSII(SMD)//B3LYP-D3/BSI

HF=-2193.9751616

^{1}I

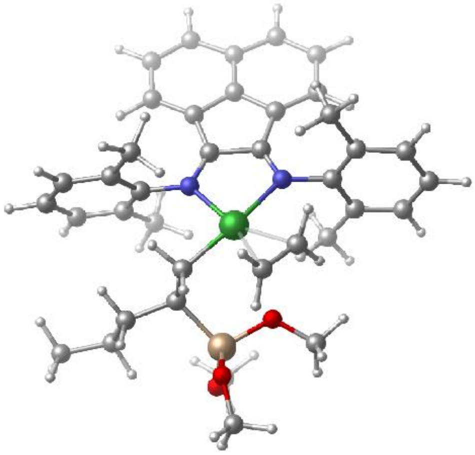

Zero-point correction= 0.776001 (Hartree/Particle)
Thermal correction to Energy= 0.824220
Thermal correction to Enthalpy= 0.825164
Thermal correction to Gibbs Free Energy= 0.693177
Sum of electronic and zero-point Energies= -2269.605266
Sum of electronic and thermal Energies= -2269.557047
Sum of electronic and thermal Enthalpies= -2269.556102
Sum of electronic and thermal Free Energies= -2269.688090

Ni	-0.02345600	1.05827700	-0.07128200
N	1.29537300	-0.49120100	-0.15705400
N	-1.38614100	-0.54778200	-0.21229900
C	2.73001500	-0.45517800	-0.17540200
C	3.41026200	-0.67962100	-1.38672100
C	3.40085200	-0.17090400	1.02881800
C	4.80583700	-0.56888700	-1.37351700
C	4.79570900	-0.07907400	0.99388400
C	5.49430600	-0.26568900	-0.19928800
H	5.35419400	-0.72351300	-2.29871300
H	5.33397100	0.14277300	1.91110700
H	6.57664200	-0.17944500	-0.21311400
C	-2.81380300	-0.54864000	-0.35693700
C	-3.33751100	-0.36718600	-1.64867700
C	-3.62627900	-0.70378000	0.77872800
C	-4.72930200	-0.32129200	-1.78373500
C	-5.01333900	-0.64891600	0.59533100
C	-5.56239300	-0.45482200	-0.67212000
H	-5.15950400	-0.18508000	-2.77237900
H	-5.66431800	-0.76096300	1.45814200
H	-6.64049700	-0.41142600	-0.79426500
C	2.62565400	-0.01623200	2.31429900
H	2.18249300	-0.97165600	2.62378100

H	3.27054600	0.33302200	3.12488300
H	1.79644000	0.69325700	2.19949200
C	2.68888700	-1.09292700	-2.64901300
H	2.53255400	-2.17934200	-2.66625000
H	1.70371800	-0.62610900	-2.74189300
H	3.27412400	-0.83014700	-3.53457400
C	-3.02081200	-0.96833700	2.13720500
H	-2.18230200	-0.29556900	2.35399500
H	-3.76538600	-0.85268100	2.92924100
H	-2.62569800	-1.99075000	2.19857600
C	-2.41259600	-0.27814700	-2.83835600
H	-1.62697800	0.47021300	-2.68577100
H	-1.91362900	-1.23996900	-3.01740000
H	-2.96544400	-0.02071100	-3.74632400
C	-0.77262500	-1.67921700	-0.21634800
C	0.72158000	-1.64798800	-0.14730400
C	1.85913800	2.39300200	-1.73136900
H	1.80776500	1.38348600	-2.15644200
C	1.48057900	2.33571100	-0.24311900
H	2.26249000	1.84327800	0.33817200
C	3.33639200	2.86518700	-1.85996900
H	3.41512800	3.91695000	-1.55323100
H	1.37300100	3.34107000	0.16323500
Si	0.64888900	3.45144800	-2.69180800
O	1.01064800	3.75643100	-4.27461500
O	-0.81448600	2.64449200	-2.61994300
O	0.53990900	4.91240900	-1.93731900
C	0.92454200	2.78520700	-5.31403200
H	1.81731400	2.14726300	-5.32246100
H	0.03511900	2.15151700	-5.20103100
H	0.86328600	3.31430800	-6.26887000
C	-2.01666500	3.27788900	-3.06091000
H	-1.94542700	3.58764200	-4.11091000
H	-2.83622200	2.55977200	-2.96332500
H	-2.24418800	4.15861700	-2.44682200
C	0.28853400	6.17947100	-2.54993700
H	0.73391100	6.23304200	-3.54826200
H	-0.79099800	6.35783900	-2.62980400
H	0.72797000	6.95351700	-1.91495100
C	-2.38188100	-5.16607300	-0.42331100
C	-1.20776800	-5.89916000	-0.34017800
C	0.05013700	-5.24339600	-0.23009700
C	0.02085400	-3.83616000	-0.21315600
C	-1.18463600	-3.08313100	-0.28959600

C	-2.39426200	-3.74754900	-0.39770700
H	1.41587600	-6.93235400	-0.14012400
H	-3.32824700	-5.69092100	-0.50858400
H	-1.24452900	-6.98490100	-0.35897600
C	1.33377200	-5.84895700	-0.13401800
C	1.19402500	-3.03385300	-0.11927400
H	-3.33338100	-3.21002800	-0.46116900
C	2.42988600	-3.65169800	-0.02653500
C	2.47623500	-5.06935100	-0.03214100
H	3.34692800	-3.07867500	0.04702400
H	3.44370200	-5.55594400	0.04258900
H	3.93240000	2.28844600	-1.13981500
C	-1.87521600	2.19783500	0.41280200
H	-2.27355000	1.59003800	1.21762300
H	-2.39941700	2.15372400	-0.53595600
C	-0.91565200	3.12580300	0.64652400
H	-0.64788400	3.86024500	-0.10354100
H	-0.48494900	3.25445400	1.63559000
C	3.95531600	2.67963300	-3.25308900
H	3.76367200	1.65114800	-3.59336800
H	3.46499700	3.34457900	-3.97254200
C	5.46511900	2.93856500	-3.24856200
H	5.89626400	2.80924000	-4.24744800
H	5.68890500	3.95991500	-2.91838100
H	5.97778700	2.24911300	-2.56633400

UB3LYP-D3/BSII(SMD)//B3LYP-D3/BSI

HF=-2193.9751616

^{3}I

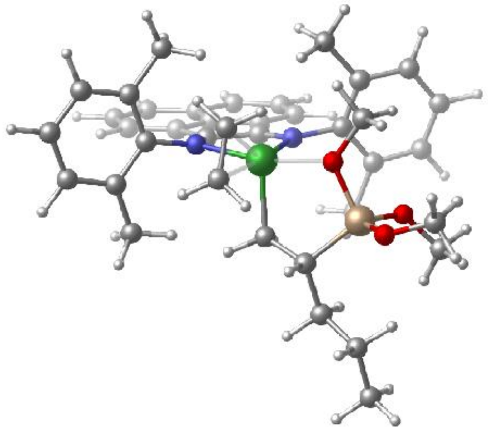

Zero-point correction= 0.772437 (Hartree/Particle)
Thermal correction to Energy= 0.820781
Thermal correction to Enthalpy= 0.821725
Thermal correction to Gibbs Free Energy= 0.688853
Sum of electronic and zero-point Energies= -2269.617155
Sum of electronic and thermal Energies= -2269.568811
Sum of electronic and thermal Enthalpies= -2269.567867
Sum of electronic and thermal Free Energies= -2269.700739

Ni	-2.69353500	3.87487300	-0.04985700
N	-1.03511900	2.58894400	-0.33832800
N	-3.69269500	2.05726100	-0.47275400
C	0.34265200	2.95223000	-0.47200300
C	0.74836500	3.40157600	-1.74193900
C	1.22737800	2.88112100	0.61666400
C	2.07798100	3.80340000	-1.90648900
C	2.55047100	3.28371200	0.40393100
C	2.97528100	3.74807000	-0.84061500
H	2.40973200	4.15022600	-2.88162000
H	3.25360800	3.23186500	1.23109300
H	4.00612100	4.05931800	-0.98133800
C	-5.08810700	1.86689700	-0.72700900
C	-5.50981500	1.98898700	-2.06808700
C	-5.98866300	1.61600400	0.32139300
C	-6.87817800	1.90312900	-2.33671400
C	-7.35226600	1.54125400	0.00304500
C	-7.79783400	1.69308500	-1.30770100
H	-7.22259000	1.99627800	-3.36318800
H	-8.06648800	1.35416900	0.80086200
H	-8.85900300	1.63717100	-1.53095800
C	0.77042200	2.39852400	1.96978300
H	0.25896400	1.43133000	1.91282700
H	1.61918600	2.28762700	2.65044900

H	0.07400100	3.11439100	2.41514200
C	-0.22288500	3.39539100	-2.89775600
H	-1.19537100	3.81418300	-2.61049900
H	0.16441800	3.96712100	-3.74573400
H	-0.41833000	2.37243100	-3.24504600
C	-5.52201200	1.35146300	1.73351900
H	-4.51536000	1.72923500	1.91555900
H	-6.19767400	1.80611500	2.46532700
H	-5.50746900	0.27213800	1.93443600
C	-4.49792000	2.13629600	-3.18119900
H	-3.96352500	1.19189600	-3.34885100
H	-4.98253900	2.41468100	-4.12103400
H	-3.73362600	2.88724100	-2.94872500
C	-2.87789100	1.06108600	-0.47666800
C	-1.40088200	1.35567100	-0.40987200
C	-2.33328400	5.16931000	2.67410100
H	-3.19666300	5.85126300	2.70333000
C	-2.77540400	3.85652400	2.01295100
H	-2.11977700	3.02105400	2.28170600
C	-1.86976800	4.95440000	4.13907500
H	-2.65049700	4.38457300	4.66240000
H	-3.80061000	3.60268800	2.29609100
Si	-1.09245400	6.08784600	1.62171000
O	-1.27517500	7.70774100	1.83127400
O	-1.51047300	5.66541600	0.03022200
O	0.49154700	5.64628700	1.71586100
C	-0.33357400	8.74121600	1.54713100
H	0.61227500	8.34397300	1.15886300
H	-0.76237500	9.42508700	0.80698200
H	-0.13202900	9.30053700	2.46654400
C	-0.86246200	6.32055900	-1.07467200
H	-0.94480000	7.40681700	-0.96596900
H	0.18721400	6.02158900	-1.12831100
H	-1.37328700	6.01777300	-1.99071600
C	1.41606500	5.87626900	2.77300500
H	1.43915700	6.93120000	3.07229800
H	1.17107100	5.26695200	3.65082200
H	2.40694300	5.59016600	2.41028800
C	-3.85087200	-2.62978400	-0.92599300
C	-2.56320800	-3.14361400	-0.96044800
C	-1.43605800	-2.28414500	-0.83585200
C	-1.71153100	-0.91387400	-0.67055300
C	-3.03180300	-0.38616100	-0.63632100
C	-4.10997400	-1.24378600	-0.76706000

H	0.20996400	-3.69670200	-0.98792900
H	-4.69341200	-3.30701000	-1.02591400
H	-2.40952700	-4.21184500	-1.08645400
C	-0.06187600	-2.65180500	-0.86585200
C	-0.69674600	0.07611500	-0.54392400
H	-5.13044400	-0.87758800	-0.75186500
C	0.63185400	-0.31045900	-0.58330900
C	0.92832200	-1.68891700	-0.74408700
H	1.43523000	0.41155000	-0.49339500
H	1.96960000	-1.99459700	-0.77279900
H	-0.97918300	4.30919300	4.14845100
C	-4.34956200	5.26150300	-1.22029700
H	-3.70479900	6.04477700	-1.60721500
H	-4.76603800	4.55682300	-1.93124800
C	-4.70209300	5.22430300	0.08068500
H	-5.40142800	4.48113100	0.45289700
H	-4.36038400	5.97987700	0.77969800
C	-1.58214500	6.24156100	4.92593700
H	-0.80238900	6.82892400	4.42202700
H	-2.47919300	6.87481200	4.91895400
C	-1.15169200	5.95771400	6.36830000
H	-0.95960400	6.88564300	6.91748100
H	-1.92709500	5.40262400	6.90945000
H	-0.23426400	5.35615600	6.39594400

UB3LYP-D3/BSII(SMD)//B3LYP-D3/BSI

HF=-2272.6038475

¹TS4

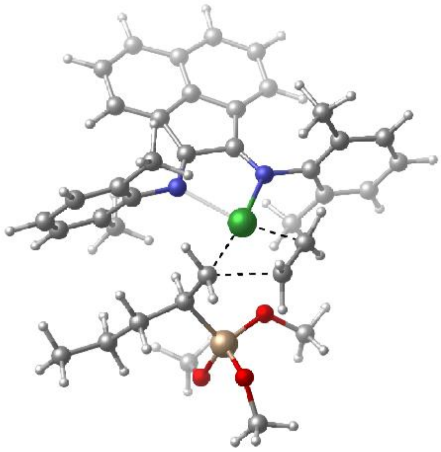

Zero-point correction= 0.776360 (Hartree/Particle)
Thermal correction to Energy= 0.823771
Thermal correction to Enthalpy= 0.824715
Thermal correction to Gibbs Free Energy= 0.694544
Sum of electronic and zero-point Energies= -2269.596820
Sum of electronic and thermal Energies= -2269.549408
Sum of electronic and thermal Enthalpies= -2269.548464
Sum of electronic and thermal Free Energies= -2269.678636

Ni	-0.20003900	1.02335800	-0.05634500
N	1.34016700	-0.57338100	-0.20245300
N	-1.36043400	-0.58367700	-0.19502300
C	2.76172400	-0.46944300	-0.19519900
C	3.48361200	-0.63777300	-1.39394300
C	3.38727800	-0.12540100	1.01878700
C	4.86682400	-0.43371000	-1.35155300
C	4.77300100	0.06792400	1.01053600
C	5.50868100	-0.07858200	-0.16467500
H	5.44355700	-0.54736400	-2.26524000
H	5.27500800	0.33129500	1.93746500
H	6.58255700	0.08188900	-0.15690400
C	-2.79479500	-0.55504200	-0.24738500
C	-3.40557500	-0.37410900	-1.49907600
C	-3.52131200	-0.67172500	0.94785500
C	-4.80287000	-0.31229400	-1.53334400
C	-4.91708900	-0.60339700	0.86555500
C	-5.55427000	-0.42492300	-0.36254100
H	-5.30220100	-0.17610300	-2.48894900
H	-5.50459000	-0.69018100	1.77554600
H	-6.63794900	-0.37169300	-0.40729900
C	2.58790700	-0.04708000	2.29802000
H	2.30501400	-1.05023000	2.64311000

H	3.16450500	0.42953900	3.09543800
H	1.65267100	0.51044200	2.16503800
C	2.80108600	-1.05017200	-2.67738200
H	2.54227500	-2.11625900	-2.66453900
H	1.86957000	-0.49852800	-2.84550300
H	3.45512200	-0.87985100	-3.53681100
C	-2.81048100	-0.89578300	2.26048600
H	-1.98125800	-0.19033000	2.39502100
H	-3.49659100	-0.78659400	3.10455200
H	-2.37801900	-1.90367900	2.30970600
C	-2.56507200	-0.27998700	-2.74907900
H	-1.77921500	0.47773800	-2.64718700
H	-2.06707700	-1.23509400	-2.96233900
H	-3.18054900	-0.02848000	-3.61751500
C	-0.75088000	-1.71793700	-0.27883700
C	0.75236800	-1.71536700	-0.25418500
C	1.73156600	2.48065600	-1.59927100
H	1.67857800	1.43832900	-1.93703800
C	1.28269600	2.56816300	-0.13341800
H	1.86144900	1.91892000	0.53195100
C	3.22730900	2.91156100	-1.65325700
H	3.30584000	3.97600700	-1.39295100
H	1.43828900	3.57958800	0.23322900
Si	0.61823800	3.46829200	-2.74453100
O	1.11980400	3.60847100	-4.31046800
O	-0.85377000	2.68793200	-2.71019800
O	0.50847400	4.99409800	-2.13563300
C	1.07978000	2.54894000	-5.26333200
H	1.96161600	1.90427400	-5.15871500
H	0.17479600	1.93836900	-5.14907600
H	1.08553100	2.99098000	-6.26323800
C	-2.03776700	3.30143400	-3.22178000
H	-1.95020800	3.50779400	-4.29579200
H	-2.87189800	2.61194400	-3.06372900
H	-2.25256200	4.24015700	-2.69567200
C	0.31251900	6.20226500	-2.87642700
H	0.82933600	6.16336500	-3.84039100
H	-0.75660600	6.37589100	-3.04924000
H	0.71319000	7.02830900	-2.28298900
C	-2.43425000	-5.16410900	-0.53182500
C	-1.27276500	-5.92188100	-0.52014500
C	0.00165000	-5.29420800	-0.43895900
C	0.00102300	-3.88798000	-0.37642100
C	-1.19133300	-3.10922100	-0.38226300

C	-2.41763900	-3.74715400	-0.46097100
H	1.33666000	-7.00988900	-0.45620500
H	-3.39360500	-5.66815500	-0.59485000
H	-1.33261100	-7.00551600	-0.57241400
C	1.27618300	-5.92598200	-0.41116500
C	1.19157400	-3.11343100	-0.29611800
H	-3.34746400	-3.19002500	-0.46838200
C	2.41757100	-3.75429000	-0.26974000
C	2.43707300	-5.17187800	-0.32640700
H	3.34604600	-3.19722200	-0.21014700
H	3.39672500	-5.67882800	-0.30476600
H	3.76380500	2.35933100	-0.86980200
C	-1.70634600	2.23089500	0.33092900
H	-2.17119800	1.82088900	1.22661500
H	-2.33009000	2.22876900	-0.56014800
C	-0.70638000	3.21739600	0.46652200
H	-0.61553600	3.99763400	-0.27943600
H	-0.37182000	3.48570600	1.46441400
C	3.93416800	2.65628900	-2.99263300
H	3.76392400	1.61340300	-3.29345300
H	3.49463100	3.28536900	-3.77363100
C	5.44027200	2.91940700	-2.89989300
H	5.93608900	2.73779100	-3.85986700
H	5.64175100	3.95814300	-2.61228800
H	5.90530600	2.26948200	-2.14911700

UB3LYP-D3/BSII(SMD)//B3LYP-D3/BSI

HF=-2272.5778781

^{3}TS4

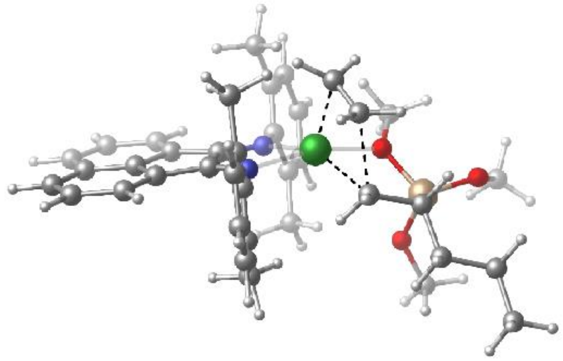

Zero-point correction=		0.773501 (Hartree/Particle)	
Thermal correction to Energy=		0.821523	
Thermal correction to Enthalpy=		0.822467	
Thermal correction to Gibbs Free Energy=		0.688719	
Sum of electronic and zero-point Energies=		-2269.589533	
Sum of electronic and thermal Energies=		-2269.541511	
Sum of electronic and thermal Enthalpies=		-2269.540567	
Sum of electronic and thermal Free Energies=		-2269.674315	
Ni	-2.20751600	3.78078900	-0.18183600
N	-0.72737200	2.42535100	-0.76910300
N	-3.36387000	1.96160300	-0.28916100
C	0.64680600	2.73278600	-1.01503400
C	1.04331900	3.10552700	-2.31095700
C	1.53180200	2.71891500	0.07615800
C	2.37987300	3.47365900	-2.50071300
C	2.86068400	3.08736300	-0.16212200
C	3.28346400	3.46534100	-1.43725600
H	2.71019000	3.77068000	-3.49248900
H	3.56681300	3.07717500	0.66390700
H	4.31756500	3.75277700	-1.60281700
C	-4.71542900	1.76573500	0.13625600
C	-5.77824100	1.98165100	-0.75826200
C	-4.92755900	1.40299200	1.48182900
C	-7.08244400	1.85550500	-0.26187000
C	-6.24651900	1.29878300	1.93295000
C	-7.31959700	1.53046400	1.07199900
H	-7.91849000	2.01050700	-0.93891800
H	-6.42995900	1.02353500	2.96813300
H	-8.33843100	1.44333900	1.43736300
C	1.04613200	2.32044300	1.44737800
H	0.61766900	1.31069300	1.44363200
H	1.86595300	2.33419300	2.17119600
H	0.27214000	3.00969900	1.80442400
C	0.04708900	3.11553700	-3.44531200

H	-0.85719000	3.67328300	-3.17311900
H	0.47668200	3.56860000	-4.34277000
H	-0.27311700	2.09851500	-3.70556100
C	-3.76468500	1.08050100	2.39095000
H	-2.97248200	1.83582900	2.33710900
H	-4.09065600	1.00216200	3.43159100
H	-3.30336300	0.12346600	2.11381400
C	-5.54642800	2.31624800	-2.21402800
H	-6.37574300	1.95224000	-2.82780400
H	-5.47845000	3.39866300	-2.37527000
H	-4.62390600	1.87097500	-2.59802000
C	-2.64401900	0.97942600	-0.70301300
C	-1.19100200	1.24586400	-1.00539100
C	-3.02400200	5.99540200	2.14233500
H	-3.42322000	6.91096200	1.67998000
C	-3.60494800	4.78142300	1.42646700
H	-3.18769500	3.83518700	1.80475000
C	-3.46113900	6.00339400	3.63320800
H	-4.55444300	5.89547100	3.67186000
H	-4.68819400	4.70240400	1.51270000
Si	-1.16470300	6.07448900	1.92576800
O	-0.63977200	7.62500700	2.00465600
O	-0.87742800	5.43269600	0.38973000
O	-0.30556900	5.07188700	2.91937500
C	0.69362600	8.08289400	2.23490000
H	1.39204800	7.25505900	2.40795800
H	1.03182300	8.65618500	1.36581600
H	0.69451100	8.73754300	3.11194200
C	0.19788000	5.90888000	-0.44381900
H	0.20886500	7.00247400	-0.44585600
H	1.15612500	5.51625700	-0.09333900
H	0.01635500	5.54390800	-1.45424500
C	0.00068700	5.25984800	4.30043800
H	0.35061900	6.27903600	4.50320800
H	-0.87379900	5.05679700	4.92842000
H	0.79567800	4.55682400	4.56320400
C	-3.84140400	-2.63403800	-1.22964300
C	-2.63502800	-3.15095400	-1.67815200
C	-1.48604500	-2.31884500	-1.79076300
C	-1.65584600	-0.97015300	-1.42700700
C	-2.89233100	-0.43990100	-0.96562200
C	-3.99390200	-1.27099400	-0.86511200
H	0.00447300	-3.72275800	-2.52269700
H	-4.70212900	-3.29110900	-1.15286700

H	-2.56264500	-4.20132300	-1.94670800
C	-0.18482500	-2.69430400	-2.22763700
C	-0.60596400	-0.01037500	-1.47946800
H	-4.95210800	-0.90072900	-0.51785200
C	0.65064300	-0.40462600	-1.90524900
C	0.83946000	-1.76071500	-2.27860000
H	1.47645700	0.29583100	-1.95792100
H	1.82314000	-2.07322900	-2.61507800
H	-3.05736200	5.11314200	4.13614500
C	-3.04089800	4.90204500	-1.73492300
H	-2.28075800	5.60583600	-2.06479700
H	-3.35942000	4.16694200	-2.46798600
C	-3.91718200	5.27348300	-0.67904200
H	-4.89352100	4.80177900	-0.65108700
H	-3.89484400	6.30216500	-0.33640000
C	-3.06440600	7.26681800	4.41225200
H	-1.97529800	7.40424600	4.38700100
H	-3.48485900	8.14589400	3.90624600
C	-3.53896100	7.22251500	5.86781800
H	-3.25844900	8.13425800	6.40573400
H	-4.62928600	7.12208700	5.92493200
H	-3.09962500	6.37022400	6.40150600

UB3LYP-D3/BSII(SMD)//B3LYP-D3/BSI

HF=-2272.5761181

^{1}J

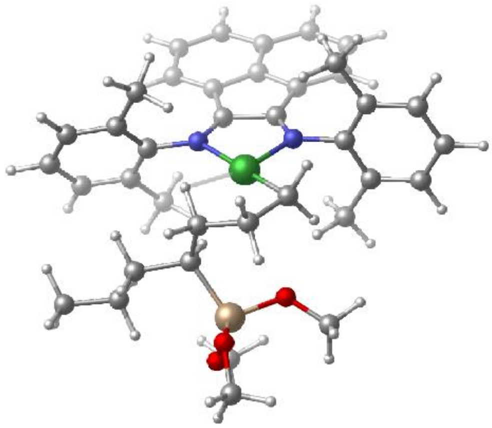

Zero-point correction=	0.776524 (Hartree/Particle)		
Thermal correction to Energy=	0.824233		
Thermal correction to Enthalpy=	0.825178		
Thermal correction to Gibbs Free Energy=	0.693459		
Sum of electronic and zero-point Energies=	-2269.618371		
Sum of electronic and thermal Energies=	-2269.570661		
Sum of electronic and thermal Enthalpies=	-2269.569717		
Sum of electronic and thermal Free Energies=	-2269.701436		

Ni	-0.05949800	0.78615000	0.04744800
N	1.39640400	-0.68211500	-0.07844600
N	-1.26989700	-0.65912400	-0.13958700
C	2.81456600	-0.53876400	-0.09713600
C	3.51869000	-0.71831800	-1.30438900
C	3.44721500	-0.13371300	1.09355300
C	4.89965100	-0.49607300	-1.28622600
C	4.82931400	0.07647800	1.06077400
C	5.55267800	-0.10367700	-0.11786200
H	5.46377900	-0.61683700	-2.20669100
H	5.33943200	0.37993400	1.97080400
H	6.62459600	0.06905600	-0.12877400
C	-2.69835100	-0.56282100	-0.21618100
C	-3.27956200	-0.34446300	-1.47601600
C	-3.44315700	-0.65151400	0.96972900
C	-4.67346900	-0.23872100	-1.52960900
C	-4.83405900	-0.53799600	0.86704700
C	-5.44536700	-0.33719000	-0.37093600
H	-5.15384500	-0.07463300	-2.49040700
H	-5.43767300	-0.60350400	1.76810000
H	-6.52606500	-0.25023200	-0.43225000
C	2.66118800	-0.00156400	2.37582300
H	2.34132500	-0.98407900	2.74617100
H	3.25949600	0.47279400	3.15826700

H	1.74678900	0.58944000	2.23935300
C	2.81425400	-1.11988500	-2.57924900
H	2.52193400	-2.17676400	-2.56640700
H	1.90118000	-0.53438900	-2.73789700
H	3.46558700	-0.96917200	-3.44412800
C	-2.75094800	-0.87020500	2.29215200
H	-1.93529600	-0.14963400	2.43314100
H	-3.45047700	-0.76723800	3.12572700
H	-2.30526700	-1.87174100	2.35050800
C	-2.41671000	-0.22787100	-2.70844800
H	-1.69400500	0.59230500	-2.61065400
H	-1.84518000	-1.14763800	-2.88684500
H	-3.02764300	-0.04055200	-3.59596100
C	-0.68616400	-1.80941100	-0.22497300
C	0.81541000	-1.82681500	-0.18470900
C	1.51872400	2.65998000	-1.54035700
H	1.33642400	1.61620600	-1.84867800
C	1.06500300	2.85924900	-0.05857900
H	1.40983100	1.98105900	0.55001900
C	3.05040500	2.89637700	-1.59409800
H	3.24857500	3.94876100	-1.34419600
H	1.70129000	3.63914400	0.37246300
Si	0.51315300	3.67054900	-2.76851500
O	1.09709800	3.71927500	-4.31361700
O	-0.96827700	2.91542400	-2.79159200
O	0.44918400	5.22342700	-2.23734100
C	0.97938000	2.65821500	-5.25640500
H	1.82663800	1.96693100	-5.16206800
H	0.04567500	2.09797800	-5.11988200
H	0.99107500	3.09046000	-6.26090900
C	-2.13940000	3.50723000	-3.34985900
H	-2.01195900	3.72083300	-4.41888100
H	-2.96451600	2.79898300	-3.22860200
H	-2.39634600	4.43816200	-2.82911300
C	0.26549500	6.39356100	-3.03860500
H	0.77587100	6.29901000	-4.00244800
H	-0.80238400	6.57379000	-3.21305200
H	0.68100100	7.24324100	-2.49046800
C	-2.42069900	-5.21576800	-0.56679000
C	-1.26920800	-5.98966100	-0.57686100
C	0.01493400	-5.38372000	-0.47946300
C	0.03481100	-3.98055200	-0.37778500
C	-1.14819300	-3.18589800	-0.36260100
C	-2.38424600	-3.80190200	-0.45818600

H	1.32623500	-7.11667300	-0.54681700
H	-3.38667100	-5.70506700	-0.64248900
H	-1.34497900	-7.07042500	-0.65900200
C	1.28086300	-6.03376600	-0.47124100
C	1.23718500	-3.22494300	-0.27663200
H	-3.30459700	-3.22871800	-0.44965200
C	2.45408600	-3.88333000	-0.27155600
C	2.45285900	-5.29880100	-0.36798600
H	3.39007300	-3.33987900	-0.20065300
H	3.40500500	-5.82002600	-0.36314700
H	3.51403600	2.29987000	-0.79427200
C	-1.37880400	2.12662700	0.19127500
H	-2.00470200	1.93803400	1.06913000
H	-1.97118800	2.11907600	-0.72409600
C	-0.40340400	3.27842900	0.31855800
H	-0.67794300	4.14153300	-0.29489900
H	-0.36027000	3.61741000	1.35913000
C	3.74987200	2.55719900	-2.91612400
H	3.52674600	1.51599000	-3.18537600
H	3.34979300	3.18302400	-3.71948700
C	5.26680500	2.75214700	-2.82656100
H	5.75781400	2.49204900	-3.77090100
H	5.51418500	3.79619700	-2.60038800
H	5.69908300	2.12868500	-2.03588000

UB3LYP-D3/BSII(SMD)//B3LYP-D3/BSI

HF=-2272.6022786

^{3}J

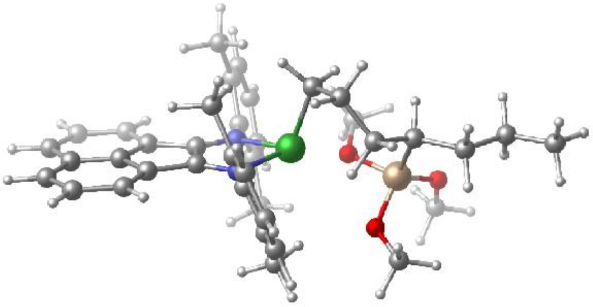

Zero-point correction= 0.776464 (Hartree/Particle)
Thermal correction to Energy= 0.824088
Thermal correction to Enthalpy= 0.825032
Thermal correction to Gibbs Free Energy= 0.693014
Sum of electronic and zero-point Energies= -2269.636393
Sum of electronic and thermal Energies= -2269.588769
Sum of electronic and thermal Enthalpies= -2269.587824
Sum of electronic and thermal Free Energies= -2269.719843

Ni	-1.82427600	3.36357200	0.29515300
N	-0.49653900	2.30641300	-0.84290900
N	-2.89752400	1.61550400	0.17904500
C	0.78727400	2.75047400	-1.28095500
C	0.93444300	3.29914500	-2.56708500
C	1.84102300	2.69872600	-0.35118900
C	2.19971300	3.78028500	-2.92365200
C	3.08983000	3.18154300	-0.75552400
C	3.27060600	3.71762100	-2.03127300
H	2.34049900	4.21152800	-3.91106500
H	3.92349400	3.14033400	-0.05959000
H	4.24513000	4.09373400	-2.32805800
C	-4.18456800	1.41300500	0.75405400
C	-5.34775200	1.48623100	-0.03821800
C	-4.23325300	1.24294400	2.15223600
C	-6.58140200	1.36953700	0.61447700
C	-5.48726800	1.11904500	2.75491200
C	-6.65533300	1.18476100	1.99417400
H	-7.49347400	1.43310400	0.02715400
H	-5.54557900	0.96877200	3.82956300
H	-7.62368000	1.09634900	2.47762700
C	1.60403100	2.15458000	1.03673600
H	1.27674300	1.10803300	1.01217400
H	2.51410800	2.20713900	1.64067300
H	0.81818800	2.72527800	1.54911400
C	-0.23861300	3.37384000	-3.51464000
H	-1.11194600	3.82469000	-3.02906300

H	0.00876800	3.96882600	-4.39772500
H	-0.54085600	2.37668600	-3.85895400
C	-2.95619200	1.15794400	2.95145400
H	-2.33480300	2.05292100	2.82143200
H	-3.16974600	1.04993300	4.01861000
H	-2.34745300	0.29929400	2.64253500
C	-5.28604500	1.70106700	-1.53208200
H	-6.23557900	2.09510700	-1.90451200
H	-4.49529500	2.40539600	-1.80704500
H	-5.08626200	0.76382500	-2.06606500
C	-2.33875400	0.78640100	-0.63736700
C	-0.98826600	1.16502800	-1.18909300
C	-3.05698500	6.35040400	1.93958700
H	-2.82849800	6.95066200	1.04556500
C	-4.02742000	5.19697700	1.51086800
H	-3.73126100	4.23078000	1.95243400
C	-3.72882800	7.30326800	2.96451400
H	-4.74629800	7.51005400	2.60186700
H	-5.01529100	5.39903400	1.94344800
Si	-1.43507400	5.60403700	2.50953200
O	-0.42080600	6.72171100	3.14915800
O	-0.64912100	4.85132700	1.19483700
O	-1.63188800	4.27387200	3.47307900
C	0.85332300	6.42043900	3.73045300
H	0.80667400	5.51696700	4.34909400
H	1.60719000	6.28033900	2.94642100
H	1.14317900	7.26914700	4.35389100
C	0.25202700	5.61822700	0.35792000
H	0.19074400	6.68288100	0.59826200
H	1.26897900	5.25807500	0.52235600
H	-0.02221700	5.45641700	-0.68505400
C	-2.45651400	4.25425700	4.64231300
H	-2.24354300	5.10605000	5.29913600
H	-3.51896500	4.26705300	4.37083100
H	-2.24108600	3.33028200	5.18391300
C	-3.65854400	-2.67728300	-1.65685700
C	-2.57944400	-3.04705400	-2.44558200
C	-1.47576200	-2.16622000	-2.62119500
C	-1.55714000	-0.92511300	-1.96303700
C	-2.66707500	-0.54085000	-1.15932300
C	-3.72278600	-1.42119800	-1.00001300
H	-0.18135100	-3.33949000	-3.91528400
H	-4.48651600	-3.36873900	-1.53538000
H	-2.57202100	-4.01803700	-2.93303500

C	-0.29603000	-2.39981900	-3.38195300
C	-0.52800700	0.05581000	-2.02397000
H	-4.58391700	-1.16711400	-0.39165500
C	0.61074100	-0.19974900	-2.76778900
C	0.70558300	-1.44247300	-3.44573000
H	1.41559500	0.52358300	-2.83779300
H	1.59605500	-1.64699100	-4.03191200
H	-3.86169000	6.79198800	3.92950300
C	-2.93089300	4.66832400	-0.78714000
H	-2.27924300	5.54147400	-0.91791700
H	-3.15565500	4.25619100	-1.77656800
C	-4.20342600	5.00842600	-0.00996900
H	-4.94154100	4.21149500	-0.15354500
H	-4.65670300	5.92654900	-0.41910900
C	-3.01740800	8.64477300	3.18677100
H	-2.00912800	8.47728500	3.57955200
H	-2.89070100	9.14269600	2.21463300
C	-3.79920000	9.56035600	4.13488600
H	-3.28470900	10.51680800	4.27637900
H	-4.80202000	9.77433100	3.74558100
H	-3.91868600	9.09721900	5.12230300

UB3LYP-D3/BSII(SMD)//B3LYP-D3/BSI

HF=-2272.6282077

[1]K

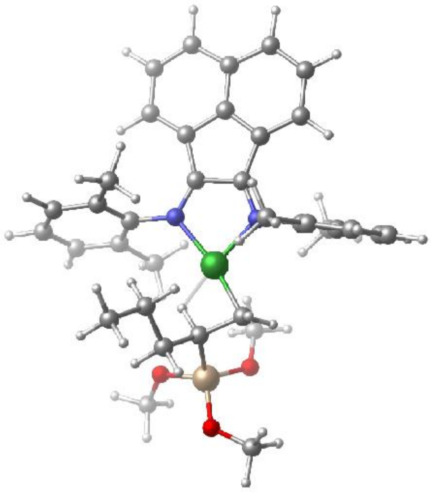

Zero-point correction= 0.717518 (Hartree/Particle)
Thermal correction to Energy= 0.763262
Thermal correction to Enthalpy= 0.764206
Thermal correction to Gibbs Free Energy= 0.635033
Sum of electronic and zero-point Energies= -2191.055971
Sum of electronic and thermal Energies= -2191.010227
Sum of electronic and thermal Enthalpies= -2191.009282
Sum of electronic and thermal Free Energies= -2191.138456

Ni	-1.83117800	3.68651200	-1.20016900
N	-0.64057500	2.21295600	-0.94264000
N	-3.26806800	2.42575800	-0.63261400
C	0.77806100	2.20995400	-1.14763600
C	1.25370600	1.99734600	-2.45188600
C	1.62676200	2.45957000	-0.05676000
C	2.63828500	2.02154100	-2.64871000
C	3.00437100	2.47743700	-0.30154800
C	3.50817900	2.25774200	-1.58403700
H	3.03290300	1.85498300	-3.64731000
H	3.68432200	2.67044900	0.52376100
H	4.58036900	2.27690300	-1.75473100
C	-4.67873000	2.64692400	-0.68201500
C	-5.43051000	2.05527100	-1.72013600
C	-5.25000900	3.53403100	0.24920300
C	-6.79798100	2.34530400	-1.77647500
C	-6.62448800	3.77561100	0.16185500
C	-7.39578900	3.18731400	-0.83931300
H	-7.39568900	1.90788200	-2.57151300
H	-7.08916900	4.43887400	0.88643400
H	-8.46038300	3.39338200	-0.89655100
C	1.06100900	2.69608400	1.32217400

H	0.59713500	1.78832800	1.72913400
H	1.84262100	3.01065500	2.01871800
H	0.28605800	3.47133600	1.30316300
C	0.28932000	1.73216600	-3.58083300
H	-0.21696800	0.76597900	-3.45682500
H	-0.49632600	2.49706900	-3.61511200
H	0.80275200	1.71939600	-4.54584100
C	-4.41238300	4.22760700	1.29651300
H	-4.00270800	5.16481700	0.89786800
H	-5.01900200	4.48807200	2.16929900
H	-3.57463000	3.61183500	1.63697600
C	-4.78948000	1.16957100	-2.76392200
H	-3.83776500	1.58504800	-3.11467300
H	-4.58318300	0.16400700	-2.37754000
H	-5.44766200	1.05953200	-3.62989100
C	-2.75224500	1.27543400	-0.35525400
C	-1.25919800	1.16678600	-0.50423400
C	-2.03563700	5.75870900	-1.84029500
H	-2.78577900	5.10985300	-1.20042200
C	-0.71669300	5.06040500	-1.87090600
H	-0.28733200	4.85103900	-2.85304000
C	-2.75912000	5.95630500	-3.19229800
H	-2.09757000	6.53877900	-3.84502300
H	-3.65728400	6.56510800	-3.03266400
C	-3.16696000	4.64632000	-3.88098200
H	-2.27885700	4.01643900	-4.03083600
C	-3.87058600	4.87166600	-5.22181400
H	-3.21701700	5.39778900	-5.92722300
H	-4.16358900	3.92109900	-5.68056000
H	-4.77632000	5.47526700	-5.09156100
H	-3.83362000	4.09034800	-3.20531900
H	0.03789400	5.35921300	-1.14012500
Si	-2.05787300	7.29511500	-0.72756500
O	-3.62748400	7.40741100	-0.25625900
O	-1.00933200	7.08011700	0.52972100
O	-1.60688200	8.63806000	-1.53960200
C	-4.18870900	8.54459200	0.41154000
H	-3.76643000	8.65440700	1.41790200
H	-4.00364700	9.46125600	-0.15796700
H	-5.26614600	8.38193700	0.49276900
C	-1.19340600	6.12055300	1.56307900
H	-0.28512500	6.09729100	2.17135200
H	-2.04453200	6.38298800	2.20356200
H	-1.36545300	5.11382100	1.15319900

C	-0.25968900	9.04956200	-1.78596600
H	0.32811300	9.04875300	-0.86186000
H	0.21965400	8.38832000	-2.51942100
H	-0.29272000	10.06261500	-2.19375000
C	-4.55152000	-1.89826400	0.84636100
C	-3.42384600	-2.69783000	0.96417500
C	-2.13440000	-2.18551300	0.64761900
C	-2.08435000	-0.84603300	0.22011800
C	-3.24221200	-0.02546100	0.09459300
C	-4.48340600	-0.55090400	0.40787100
H	-0.86813600	-3.90934300	1.03663700
H	-5.52283100	-2.31477400	1.09403800
H	-3.52270200	-3.72634800	1.30008700
C	-0.88938600	-2.87217900	0.71355700
C	-0.86942900	-0.18715800	-0.12787700
H	-5.38664300	0.04277500	0.31731500
C	0.32697600	-0.87926400	-0.05506600
C	0.29283700	-2.23189000	0.37082000
H	1.26945400	-0.40781100	-0.31115300
H	1.22742600	-2.78070800	0.43067200

UB3LYP-D3/BSII(SMD)//B3LYP-D3/BSI

HF=-2193.9621976

^{3}K

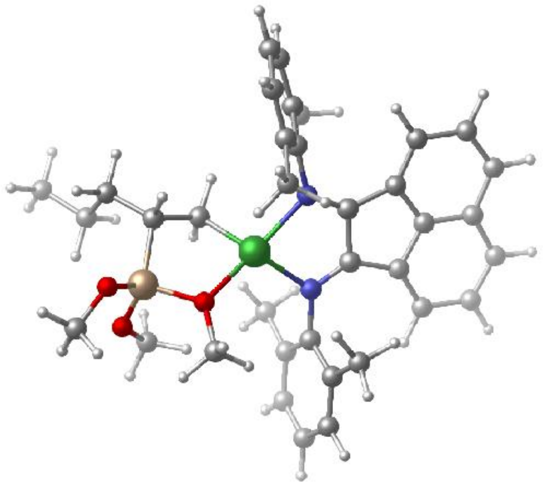

Zero-point correction= 0.717863 (Hartree/Particle)
Thermal correction to Energy= 0.763611
Thermal correction to Enthalpy= 0.764555
Thermal correction to Gibbs Free Energy= 0.635009
Sum of electronic and zero-point Energies= -2191.058637
Sum of electronic and thermal Energies= -2191.012889
Sum of electronic and thermal Enthalpies= -2191.011945
Sum of electronic and thermal Free Energies= -2191.141491

Ni	-2.37603700	3.63105900	-0.92606100
N	-0.85932600	2.42641900	-0.27610000
N	-3.48282000	1.92965000	-0.69049400
C	0.50612500	2.81321900	-0.16377200
C	1.44736500	2.40395900	-1.13116900
C	0.83364400	3.70712100	0.87458300
C	2.75956200	2.87163000	-0.99224900
C	2.15879600	4.13571800	0.97902200
C	3.11874500	3.71687800	0.05685000
H	3.50235000	2.57679700	-1.72847700
H	2.43664200	4.80811100	1.78594900
H	4.14353800	4.06471100	0.14543700
C	-4.89218300	1.81109700	-0.88564100
C	-5.39453700	1.29319500	-2.09223600
C	-5.72532400	2.30062700	0.13603100
C	-6.78474100	1.26381400	-2.25014800
C	-7.10852800	2.25230100	-0.06703800
C	-7.63656400	1.73782900	-1.25160600
H	-7.19932600	0.87067900	-3.17437000
H	-7.77184600	2.61579100	0.71324200
H	-8.71228300	1.70655400	-1.39712200
C	-0.22637400	4.16839600	1.84473800
H	-1.05017600	4.67805300	1.32147800

H	-0.67373500	3.32990100	2.39162500
H	0.18446500	4.87072200	2.57477200
C	1.05578800	1.54034400	-2.30686100
H	0.08551000	1.84195600	-2.71619300
H	1.79841500	1.62027000	-3.10518900
H	0.97933900	0.48159300	-2.03152200
C	-5.12702400	2.82706400	1.41772700
H	-5.88932300	3.28842500	2.05140700
H	-4.64973800	2.02519900	1.99505100
H	-4.34875700	3.57550800	1.21854900
C	-4.46573500	0.80633400	-3.17578400
H	-3.87360200	-0.05574500	-2.84567900
H	-5.02848200	0.50466200	-4.06297300
H	-3.77426100	1.60083800	-3.47157200
C	-2.73365500	0.93499500	-0.35901900
C	-1.26400800	1.20575200	-0.14674600
C	-0.64831900	4.84381200	-2.97114600
H	0.08114800	4.40667900	-2.27538200
H	-0.79463100	5.87881100	-2.62959200
C	-1.96663000	4.06616400	-2.91780800
C	0.00821800	4.85901800	-4.36949000
H	0.23343000	3.82185100	-4.65941800
H	0.97602600	5.37422300	-4.29215200
C	-0.83297900	5.51817200	-5.46803200
H	-1.78283500	4.97919700	-5.57906700
C	-0.10863400	5.55767700	-6.81721700
H	0.14137100	4.54591900	-7.15960200
H	-0.72768600	6.02790100	-7.58887500
H	0.82772200	6.12450800	-6.74718400
H	-1.09696600	6.53963800	-5.16109800
H	-1.92463700	3.13589900	-3.49266500
Si	-3.56492400	4.95940600	-3.01694200
O	-4.73708600	4.01290900	-3.65258200
O	-3.73455600	6.47113500	-3.63655600
O	-3.76793800	5.12823300	-1.30671700
C	-6.08497600	4.38791800	-3.94428600
H	-6.73027100	4.13207900	-3.09631800
H	-6.16580900	5.45739500	-4.16596300
H	-6.41191800	3.81837200	-4.81842700
C	-3.15812800	7.67219100	-3.12888200
H	-3.83749900	8.49857700	-3.35619900
H	-3.00739500	7.62363400	-2.04258800
H	-2.19458400	7.86418300	-3.61317100
C	-4.95363600	5.65447500	-0.68571600

H	-5.74408900	4.89832000	-0.68545300
H	-4.70904200	5.93131300	0.34213300
H	-5.28906000	6.54349000	-1.22870800
C	-3.89351500	-2.67627000	0.24061600
C	-2.65621300	-3.19663000	0.59023700
C	-1.49972100	-2.36752500	0.61266200
C	-1.69213300	-1.01967100	0.25711100
C	-2.96206600	-0.48441800	-0.09578200
C	-4.07084700	-1.31227400	-0.10688700
H	0.03550800	-3.77416800	1.23897200
H	-4.75938400	-3.33085900	0.23225100
H	-2.56528700	-4.24737500	0.85137300
C	-0.17228500	-2.74528800	0.95846800
C	-0.63919600	-0.06148600	0.23281000
H	-5.05306800	-0.93852800	-0.37420600
C	0.64124300	-0.45729900	0.57995600
C	0.85381000	-1.81217400	0.94225100
H	1.47042600	0.24155500	0.57465500
H	1.85741200	-2.12482800	1.21308500

UB3LYP-D3/BSII(SMD)//B3LYP-D3/BSI

HF=-2193.9704071

[1]TS5

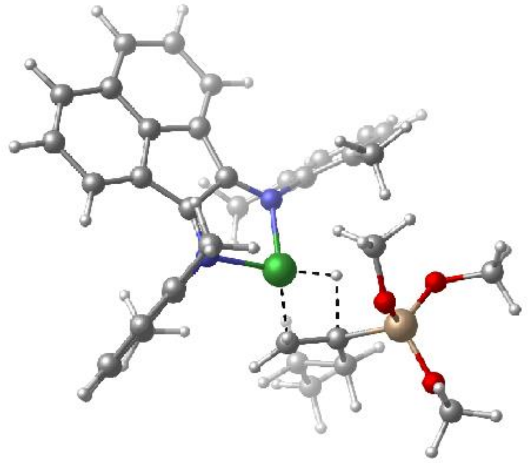

Zero-point correction= 0.713911 (Hartree/Particle)
Thermal correction to Energy= 0.758537
Thermal correction to Enthalpy= 0.759482
Thermal correction to Gibbs Free Energy= 0.633534
Sum of electronic and zero-point Energies= -2191.044158
Sum of electronic and thermal Energies= -2190.999532
Sum of electronic and thermal Enthalpies= -2190.998588
Sum of electronic and thermal Free Energies= -2191.124536

Ni	-1.94445500	3.71394400	-1.25300500
N	-0.69662800	2.22560900	-0.97743200
N	-3.31061300	2.47012500	-0.61257500
C	0.70306000	2.17318600	-1.28032500
C	1.07434700	1.83738000	-2.59301100
C	1.63639200	2.50368500	-0.28512800
C	2.43985500	1.81999700	-2.89639400
C	2.99139000	2.47370500	-0.63394900
C	3.39216900	2.13304800	-1.92622500
H	2.75410200	1.55996800	-3.90353400
H	3.73510600	2.72560200	0.11712800
H	4.44806500	2.11783400	-2.17900700
C	-4.73381200	2.64458100	-0.63430300
C	-5.47209200	2.01889400	-1.65783900
C	-5.32949600	3.47754100	0.32765300
C	-6.85619100	2.22237000	-1.67495200
C	-6.71808200	3.63686700	0.27880100
C	-7.47854500	3.01435200	-0.71036000
H	-7.44719200	1.75612000	-2.45858700
H	-7.20247700	4.26233100	1.02370700
H	-8.55497500	3.15508700	-0.73658800
C	1.17896700	2.87243500	1.10468000
H	0.70410500	2.02283300	1.61204600

H	2.01949200	3.20161200	1.72124800
H	0.43960100	3.68101500	1.07434600
C	0.02247600	1.48929000	-3.61784700
H	-0.47059500	0.53832300	-3.37720100
H	-0.76608000	2.25132600	-3.65496400
H	0.45873300	1.39552100	-4.61585800
C	-4.49377100	4.19548200	1.35587800
H	-3.95792100	5.02822500	0.88483500
H	-5.12090300	4.60800600	2.15157000
H	-3.74812600	3.53816100	1.81674000
C	-4.79853500	1.17654900	-2.71655000
H	-3.89876300	1.66681500	-3.10701700
H	-4.49101600	0.19869000	-2.32499800
H	-5.47609300	0.99595400	-3.55524200
C	-2.78537100	1.34117100	-0.26815500
C	-1.30123500	1.21067600	-0.45932900
C	-1.75942700	5.78440700	-2.08127800
H	-2.95857400	4.85371600	-1.35251700
C	-0.60228700	4.97007300	-1.99795300
H	-0.18446400	4.50510200	-2.89002800
C	-2.49370800	5.99075600	-3.40462800
H	-1.94924100	6.78494900	-3.93564700
H	-3.49268400	6.39147800	-3.19608000
C	-2.62404800	4.75653500	-4.30585500
H	-1.63041000	4.38542600	-4.58662500
C	-3.43849900	5.04330100	-5.57049900
H	-2.96725500	5.82807400	-6.17353400
H	-3.52615600	4.14852400	-6.19595300
H	-4.45130300	5.37881900	-5.31826700
H	-3.10596000	3.95355800	-3.72651300
H	0.11657600	5.14360600	-1.19716700
Si	-1.94603200	7.15020700	-0.78933200
O	-3.54036100	7.14759000	-0.37962100
O	-0.95586300	6.84528400	0.49556800
O	-1.49046200	8.56420600	-1.46482800
C	-4.20242400	8.23328600	0.27529300
H	-3.94015600	8.26367800	1.34038000
H	-3.93977800	9.19163100	-0.18667300
H	-5.27925900	8.07356700	0.17991300
C	-1.27784800	5.99146800	1.58669600
H	-0.41371900	5.95854300	2.25585800
H	-2.14601800	6.36304100	2.14459100
H	-1.49881200	4.97068200	1.24374700
C	-0.48069100	9.48205700	-1.03616900

H	0.02465300	9.13033000	-0.13119600
H	0.25317100	9.59844700	-1.83954400
H	-0.94798400	10.45172900	-0.83777800
C	-4.57823600	-1.75104700	1.13663100
C	-3.45555300	-2.55764300	1.25474100
C	-2.17171500	-2.07532800	0.87455700
C	-2.12163900	-0.75664000	0.38687400
C	-3.27498300	0.07132000	0.26191800
C	-4.51130200	-0.42530300	0.63640200
H	-0.91131100	-3.79534900	1.29777200
H	-5.54527200	-2.14505700	1.43306200
H	-3.55420300	-3.56894800	1.63948200
C	-0.93253900	-2.77346500	0.92917300
C	-0.91293900	-0.12990000	-0.03360800
H	-5.41094000	0.17366400	0.54770500
C	0.27752600	-0.83288600	0.02813400
C	0.24390100	-2.16411200	0.51712300
H	1.21487300	-0.38593900	-0.28457100
H	1.17389700	-2.72144800	0.56934700

UB3LYP-D3/BSII(SMD)//B3LYP-D3/BSI

HF=-2193.9466587

^{3}TS5

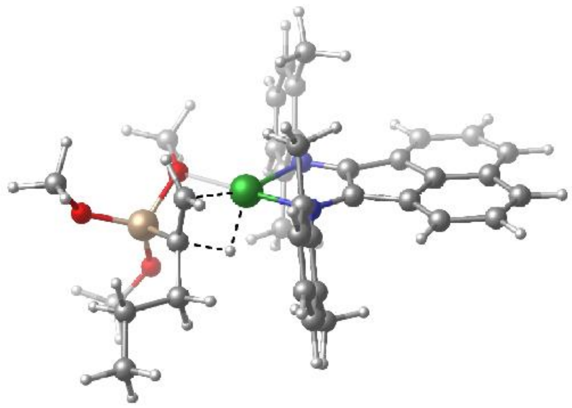

Zero-point correction= 0.711745 (Hartree/Particle)
Thermal correction to Energy= 0.757811
Thermal correction to Enthalpy= 0.758755
Thermal correction to Gibbs Free Energy= 0.627739
Sum of electronic and zero-point Energies= -2191.025155
Sum of electronic and thermal Energies= -2190.979090
Sum of electronic and thermal Enthalpies= -2190.978146
Sum of electronic and thermal Free Energies= -2191.109161

Ni	-2.60740800	3.44319000	-1.58428600
N	-1.03195300	2.08806900	-1.27138000
N	-3.62585700	1.99837300	-0.52396000
C	0.29664100	2.22457800	-1.77085300
C	0.52022300	1.89017100	-3.11892900
C	1.29752500	2.76305300	-0.94291500
C	1.80198600	2.10674600	-3.63598600
C	2.56371100	2.96257700	-1.50559300
C	2.81590800	2.64060700	-2.83967500
H	2.00275100	1.85154300	-4.67294000
H	3.35327600	3.38447000	-0.88964500
H	3.80357800	2.80680800	-3.25929200
C	-5.02935300	1.99765200	-0.26022500
C	-5.87792800	1.46267800	-1.24428400
C	-5.50625900	2.60067600	0.91488600
C	-7.25524500	1.50893300	-1.00515300
C	-6.89259300	2.63385700	1.10659900
C	-7.76065900	2.08756400	0.15989000
H	-7.93472600	1.09557100	-1.74579700
H	-7.29007900	3.09400500	2.00719400
H	-8.83313800	2.12011300	0.32709400
C	0.99479200	3.14191200	0.48644300
H	0.10048300	3.77448300	0.54261300
H	0.80523200	2.25704700	1.10715100

H	1.83096400	3.68722400	0.93202100
C	-0.58478600	1.28445200	-3.95081200
H	-1.49232200	1.90051700	-3.94347700
H	-0.27041300	1.16158500	-4.99080800
H	-0.86968500	0.29567500	-3.56864900
C	-4.54186500	3.18796000	1.91631800
H	-5.07219700	3.73312300	2.70158500
H	-3.94104900	2.40621700	2.39886300
H	-3.83811300	3.87572900	1.43031400
C	-5.30222900	0.89537700	-2.51933400
H	-4.65104300	0.03441300	-2.32410600
H	-6.09588400	0.56494300	-3.19531400
H	-4.69220000	1.64255100	-3.04495300
C	-2.85147100	1.05818400	-0.10831200
C	-1.39834700	1.11092300	-0.52140800
C	-1.56089100	5.53385700	-2.21123100
H	-1.95550800	4.71735300	-0.74837300
H	-2.32563800	6.25262800	-1.91703400
C	-1.82416100	4.72793600	-3.34165300
C	-0.14506100	5.90668800	-1.83192200
H	0.49557500	5.02397700	-1.91451400
H	-0.10743500	6.24201200	-0.78926900
C	0.40304900	7.01590300	-2.75230000
H	0.34573200	6.67593300	-3.79415800
C	1.84963700	7.37482500	-2.39831900
H	2.50112400	6.49709400	-2.48895800
H	2.23770200	8.15354500	-3.06311900
H	1.92557700	7.74399900	-1.36860900
H	-0.23472100	7.90744600	-2.67718000
H	-0.97127400	4.22223300	-3.79022700
Si	-3.50919000	4.61848200	-4.06489400
O	-3.63259400	3.27471400	-4.99172200
O	-4.15183300	5.87878300	-4.89669800
O	-4.34350200	4.43066200	-2.59678200
C	-4.57676600	3.03342400	-6.03958100
H	-5.50385500	2.62006100	-5.62405900
H	-4.80467600	3.95196600	-6.58939500
H	-4.13791200	2.30174000	-6.72251800
C	-4.04112800	7.24980500	-4.51099800
H	-4.45276900	7.85803300	-5.31958100
H	-4.60859200	7.44981700	-3.59274200
H	-2.99281400	7.53220300	-4.35235600
C	-5.76083500	4.57169200	-2.40676400
H	-6.29661100	3.75371500	-2.89722500

H	-5.96152500	4.53194100	-1.33531600
H	-6.09789200	5.52882900	-2.81666500
C	-3.89548300	-1.96805500	2.01055300
C	-2.65056600	-2.57865800	2.06059100
C	-1.52694400	-1.99306200	1.41263400
C	-1.76038600	-0.78069200	0.73770900
C	-3.03769900	-0.15440300	0.68507000
C	-4.11394500	-0.74701800	1.32224900
H	0.04381800	-3.42474000	1.87193800
H	-4.73489500	-2.43776200	2.51362100
H	-2.52896600	-3.51464700	2.59887200
C	-0.19517100	-2.49294300	1.36680800
C	-0.74370300	-0.07198100	0.03840400
H	-5.10073900	-0.29765500	1.30196500
C	0.54209400	-0.58132300	0.00853600
C	0.79610500	-1.80284200	0.68490900
H	1.34178100	-0.06702800	-0.51319700
H	1.80348900	-2.20674900	0.66552400

UB3LYP-D3/BSII(SMD)//B3LYP-D3/BSI

HF=-2193.9286758

^{3}L

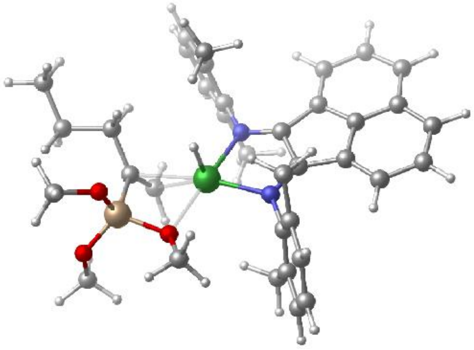

Zero-point correction= 0.713345 (Hartree/Particle)
Thermal correction to Energy= 0.759783
Thermal correction to Enthalpy= 0.760727
Thermal correction to Gibbs Free Energy= 0.628756
Sum of electronic and zero-point Energies= -2191.041455
Sum of electronic and thermal Energies= -2190.995017
Sum of electronic and thermal Enthalpies= -2190.994073
Sum of electronic and thermal Free Energies= -2191.126043

Ni	-2.61367300	3.47787200	-1.55188900
N	-1.06262700	2.11680300	-1.28332200
N	-3.67194600	1.95248700	-0.62030900
C	0.28581100	2.29380700	-1.71348700
C	0.59253600	1.94854500	-3.04185500
C	1.22209400	2.87798800	-0.84236300
C	1.88779400	2.21314300	-3.49973500
C	2.50596600	3.12009300	-1.34368500
C	2.83756600	2.79719000	-2.66009100
H	2.15112500	1.95336500	-4.52154500
H	3.24652900	3.57983200	-0.69491900
H	3.83741600	3.00035900	-3.03203900
C	-5.07560100	1.93519400	-0.36192200
C	-5.91652400	1.43377600	-1.37052700
C	-5.56453300	2.47974200	0.83816300
C	-7.29620500	1.46715600	-1.14245000
C	-6.95296100	2.50166500	1.01730800
C	-7.81278000	1.99858300	0.04018400
H	-7.96777200	1.07597300	-1.90216800
H	-7.35883300	2.91939100	1.93469300
H	-8.88670600	2.02289400	0.19959400
C	0.83677100	3.25085600	0.56842600
H	-0.10954200	3.80480600	0.58508500
H	0.69860500	2.35947600	1.19391100
H	1.61069600	3.86600000	1.03548900
C	-0.43688500	1.26760400	-3.91218600

H	-1.41499600	1.76031900	-3.85817800
H	-0.12040400	1.24955300	-4.95885200
H	-0.59284500	0.22849200	-3.59403400
C	-4.61618700	3.01598200	1.88262300
H	-5.15878000	3.54609400	2.66996000
H	-4.04899200	2.20448300	2.35723700
H	-3.88408500	3.69987400	1.43692300
C	-5.32894300	0.88933100	-2.64957700
H	-4.69955300	0.01032800	-2.46240600
H	-6.11628300	0.59050400	-3.34749200
H	-4.69443900	1.63416700	-3.14548400
C	-2.88499700	1.04140400	-0.16902400
C	-1.42525100	1.13439100	-0.53681500
C	-1.35735600	5.47117300	-2.69817800
H	-2.34598000	4.39411800	-0.29306900
H	-2.05429200	6.12214400	-2.16757300
C	-1.83112600	4.62029600	-3.64789200
C	0.08483400	5.66595100	-2.34805800
H	0.69923200	4.89926900	-2.83066700
H	0.19949700	5.54142300	-1.26352500
C	0.59295700	7.06887200	-2.74183600
H	0.47649800	7.20231100	-3.82533800
C	2.05957400	7.26397000	-2.34481400
H	2.69739100	6.50949500	-2.82041900
H	2.42271500	8.25219600	-2.64515400
H	2.18771300	7.17310600	-1.25946700
H	-0.03032000	7.83524400	-2.26134700
H	-1.10414600	4.01745800	-4.18905000
Si	-3.62703500	4.56884000	-4.10066000
O	-3.91025800	3.24490700	-5.01278700
O	-4.29168000	5.87542300	-4.82854400
O	-4.25626400	4.40570200	-2.53899600
C	-5.05077800	3.00640500	-5.84639100
H	-5.89178200	2.64509500	-5.24267800
H	-5.34770900	3.91549600	-6.37860700
H	-4.77621000	2.23559600	-6.57006500
C	-4.05741000	7.24077100	-4.47451000
H	-4.52502200	7.86605200	-5.23799600
H	-4.50108800	7.47773400	-3.49929500
H	-2.98336500	7.46041000	-4.44160000
C	-5.58657000	4.74064300	-2.09665100
H	-6.28982200	3.95886100	-2.39249500
H	-5.56032900	4.80591400	-1.00883900
H	-5.88891100	5.69968900	-2.52601000

C	-3.89257200	-1.97764500	1.98224500
C	-2.63095100	-2.54564300	2.08104300
C	-1.50863400	-1.93478300	1.45450700
C	-1.76165800	-0.74400900	0.74861800
C	-3.05590900	-0.16139200	0.64615800
C	-4.12992500	-0.77711800	1.26385800
H	0.09443100	-3.30221300	1.99081400
H	-4.73109500	-2.46468300	2.47015100
H	-2.49451000	-3.46616300	2.64194600
C	-0.16005700	-2.38910900	1.45973600
C	-0.74808700	-0.01371300	0.06695300
H	-5.12971600	-0.36079300	1.20799100
C	0.55470500	-0.47906700	0.08748400
C	0.82842300	-1.67801400	0.79542300
H	1.35316500	0.05249500	-0.41844300
H	1.84913800	-2.04695400	0.81569700

UB3LYP-D3/BSII(SMD)//B3LYP-D3/BSI

HF=-2193.9469254

[1]M

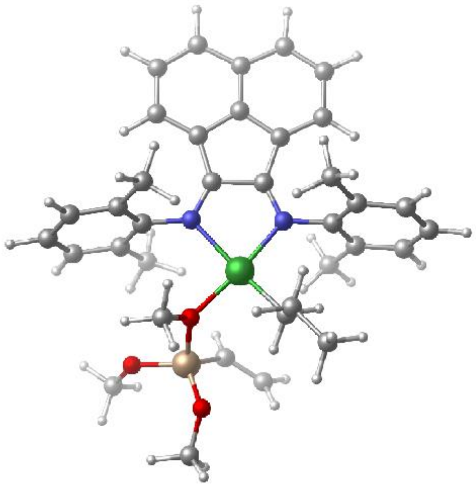

Zero-point correction= 0.716551 (Hartree/Particle)
Thermal correction to Energy= 0.763195
Thermal correction to Enthalpy= 0.764139
Thermal correction to Gibbs Free Energy= 0.633092
Sum of electronic and zero-point Energies= -2191.047403
Sum of electronic and thermal Energies= -2191.000760
Sum of electronic and thermal Enthalpies= -2190.999815
Sum of electronic and thermal Free Energies= -2191.130862

Ni	-1.91424100	3.50186500	-1.52730600
N	-0.53733500	1.93120400	-1.55102700
N	-3.03443000	2.18063500	-0.64668200
C	0.81797000	1.97271000	-1.99308600
C	1.07022500	1.93907600	-3.37456100
C	1.84227800	2.15904500	-1.04395800
C	2.39056500	2.10069900	-3.80463000
C	3.14807000	2.31628600	-1.51987500
C	3.42345900	2.29118500	-2.88797900
H	2.60510500	2.07794300	-4.86977100
H	3.95415300	2.46847700	-0.80712100
H	4.44306400	2.42274300	-3.23766700
C	-4.37513300	2.35910900	-0.16857200
C	-5.41197100	2.38989500	-1.11830900
C	-4.59953300	2.52853200	1.20875300
C	-6.71442000	2.59329300	-0.65368700
C	-5.91972300	2.74015600	1.62504700
C	-6.96837900	2.77142600	0.70676000
H	-7.53298100	2.61594000	-1.36784100
H	-6.12110100	2.88146700	2.68350000
H	-7.98477900	2.93745400	1.05113500
C	1.53684600	2.20205300	0.43505700
H	2.40206300	2.55424500	1.00296200

H	0.69077300	2.86499100	0.65623200
H	1.26893500	1.20993800	0.81959300
C	-0.05050600	1.67877200	-4.34765100
H	-0.24281900	0.60238900	-4.44839500
H	-0.98332700	2.13514700	-4.00410700
H	0.18824100	2.06962900	-5.34044700
C	-3.47602600	2.44036300	2.21510800
H	-3.24358800	1.39410400	2.45303600
H	-2.55431800	2.89933000	1.84387500
H	-3.75114000	2.93729800	3.14942300
C	-5.10759800	2.19344100	-2.58215700
H	-4.36299300	2.92059400	-2.93011400
H	-4.69163100	1.19609700	-2.77410600
H	-6.01003500	2.30385900	-3.18976900
C	-2.46379100	1.02647500	-0.51018600
C	-1.04773400	0.89436200	-0.98909200
C	-3.64631000	5.06287800	-4.89592400
H	-4.51702900	4.60079600	-5.35782200
H	-3.79265700	6.06519300	-4.49913700
C	-2.46397900	4.43501100	-4.80609400
H	-2.37708700	3.43304700	-5.22527500
C	-3.11452500	4.98415700	-1.23811400
H	-4.15155300	4.63679200	-1.27074400
H	-2.95849500	5.70897300	-2.04370000
C	-2.77350700	5.59922000	0.11383900
H	-2.90506000	4.85609600	0.90854300
C	-3.65174700	6.82326500	0.42958800
H	-4.71039500	6.53960800	0.45693600
H	-3.39128600	7.26050600	1.40059700
H	-3.53056300	7.59828100	-0.33624100
H	-1.71650000	5.90005600	0.13533300
Si	-0.99003200	5.27324400	-4.07105600
O	-1.28033800	6.86567300	-3.79180600
O	0.39151900	4.99643300	-4.91129100
O	-0.66267200	4.65044900	-2.52668300
C	0.43506300	5.24284200	-1.77841700
H	0.52962600	4.69285400	-0.84350700
H	1.35493800	5.13851000	-2.35781200
H	0.21124000	6.29291800	-1.57863300
C	0.54734800	4.90973100	-6.32560800
H	-0.36524000	4.54882600	-6.81705300
H	0.80999000	5.88912300	-6.74088700
H	1.36147800	4.20959700	-6.53375100
C	-1.16534000	7.90411800	-4.76754100

H	-1.38314000	8.85215300	-4.27039200
H	-0.15049100	7.94520100	-5.18105500
H	-1.88283100	7.76085900	-5.58550500
C	-2.87194000	-0.27197400	0.02601300
C	-4.03314600	-0.79158100	0.57274300
C	-1.73081200	-1.11527900	-0.11161500
C	-4.03672800	-2.15105500	0.97798900
H	-4.92279000	-0.18445400	0.69556400
C	-1.71676500	-2.46481200	0.28751000
C	-0.60704000	-0.46960800	-0.69842000
C	-2.92368400	-2.96844100	0.84782800
H	-4.94596500	-2.56072700	1.40677900
C	-0.49824700	-3.17064800	0.08094000
C	0.56468400	-1.17886200	-0.88948700
H	-2.97083100	-4.00361800	1.17456200
C	0.59804900	-2.53953100	-0.48796900
H	-0.43004100	-4.21497800	0.37294500
H	1.43998000	-0.71454200	-1.33097200
H	1.51511500	-3.10183600	-0.63358800

UB3LYP-D3/BSII(SMD)//B3LYP-D3/BSI

HF=-2193.9513608

^{3}M

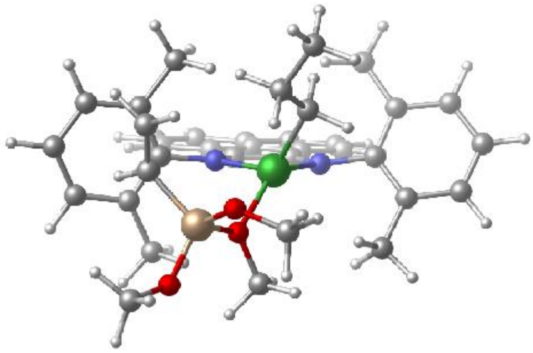

Zero-point correction= 0.714605 (Hartree/Particle)
Thermal correction to Energy= 0.761644
Thermal correction to Enthalpy= 0.762588
Thermal correction to Gibbs Free Energy= 0.630962
Sum of electronic and zero-point Energies= -2191.033796
Sum of electronic and thermal Energies= -2190.986757
Sum of electronic and thermal Enthalpies= -2190.985813
Sum of electronic and thermal Free Energies= -2191.117440

Ni	-2.59626700	3.49258000	-1.55671900
N	-1.09041500	2.23152500	-0.96654600
N	-3.78095000	2.04484200	-0.59521300
C	0.31913100	2.41792600	-1.13242700
C	0.96710500	1.84906000	-2.24169800
C	0.98952800	3.20942900	-0.18499900
C	2.33947300	2.08535200	-2.38319200
C	2.36115400	3.41742100	-0.36451700
C	3.03376700	2.86130000	-1.45318700
H	2.86342800	1.65778400	-3.23367100
H	2.90182000	4.02418300	0.35678700
H	4.09800900	3.03578900	-1.58025900
C	-5.15173800	2.19662200	-0.25213000
C	-6.08105700	2.26382200	-1.31151700
C	-5.54170200	2.39550100	1.09157200
C	-7.42936100	2.44637400	-1.00429300
C	-6.90452000	2.59572600	1.34700900
C	-7.84377900	2.60518500	0.31895200
H	-8.15810900	2.47374000	-1.80969100
H	-7.22522300	2.75744400	2.37266900
H	-8.89505600	2.75601900	0.54501000
C	0.23897500	3.79687700	0.98359900
H	-0.66709900	4.31375600	0.64897400
H	-0.08005300	3.01470500	1.68502300
H	0.86067100	4.50672300	1.53564100
C	0.20071800	1.01303400	-3.23616500

H	-0.19242800	0.09792400	-2.77562400
H	-0.65261400	1.57123800	-3.63349100
H	0.83625000	0.71855400	-4.07539600
C	-4.55770600	2.43776000	2.23771900
H	-4.93271200	3.08744700	3.03358200
H	-4.39820300	1.44382100	2.67303000
H	-3.57952800	2.81822900	1.92853700
C	-5.61450700	2.14858500	-2.74005000
H	-5.02500600	1.24091600	-2.91330200
H	-6.45916900	2.14446500	-3.43438000
H	-4.97565600	3.00342200	-3.00309600
C	-3.04045500	1.09265700	-0.13368300
C	-1.55186400	1.18715500	-0.36651700
C	0.69450400	6.50840700	-2.07636000
H	0.01252000	7.35366000	-2.11831900
H	1.54702500	6.60860700	-1.40633100
C	0.49946300	5.40103900	-2.80592900
C	-3.24078400	5.02753800	-0.45175100
H	-3.29311200	4.68219500	0.58797800
H	-4.27784900	5.10669200	-0.82502500
C	-2.48853100	6.34096500	-0.58388200
H	-1.45067600	6.21066200	-0.25219400
C	-3.13633600	7.47768500	0.22807000
H	-3.17290200	7.22546800	1.29460300
H	-2.57409900	8.41314800	0.11946700
H	-4.16480300	7.65997100	-0.10579600
H	-2.42937300	6.63894700	-1.63642300
H	1.22367500	4.59255200	-2.72982900
Si	-0.88121500	5.21832100	-4.00312400
O	-0.39523100	4.73663700	-5.50035900
O	-1.74301100	6.61728400	-4.01342500
O	-1.91302800	3.93551700	-3.54844300
C	0.48178700	5.50638000	-6.33067300
H	0.69959500	4.91325800	-7.22195600
H	0.00796900	6.44725100	-6.63339700
H	1.42196700	5.72957900	-5.81178400
C	-3.08973900	6.74366700	-4.46492500
H	-3.77080100	6.17927300	-3.81392300
H	-3.36078400	7.80114100	-4.42263600
H	-3.20268800	6.39243100	-5.49854000
C	-2.62693300	3.17407000	-4.55559100
H	-1.97615300	2.98235600	-5.40945600
H	-2.93157300	2.22827700	-4.10217500
H	-3.51586100	3.72338100	-4.88118200

C	-4.24350000	-2.04682800	1.73474800
C	-2.99703700	-2.62489900	1.92328500
C	-1.82182700	-1.97956700	1.44772600
C	-2.00715800	-0.74811400	0.79164600
C	-3.28321900	-0.14624200	0.61015200
C	-4.40938000	-0.79920500	1.08040500
H	-0.27700000	-3.38142800	2.05924100
H	-5.12603900	-2.55968700	2.10420300
H	-2.91359200	-3.57835500	2.43747300
C	-0.47954200	-2.43851700	1.55873400
C	-0.93477100	0.00910300	0.24363400
H	-5.39962600	-0.37233400	0.96485400
C	0.36162600	-0.46145700	0.36594400
C	0.56774800	-1.69541800	1.03478100
H	1.20477600	0.08738000	-0.03770400
H	1.58238900	-2.06828900	1.13392500

UB3LYP-D3/BSII(SMD)//B3LYP-D3/BSI

HF=-2193.9431886

[1]N

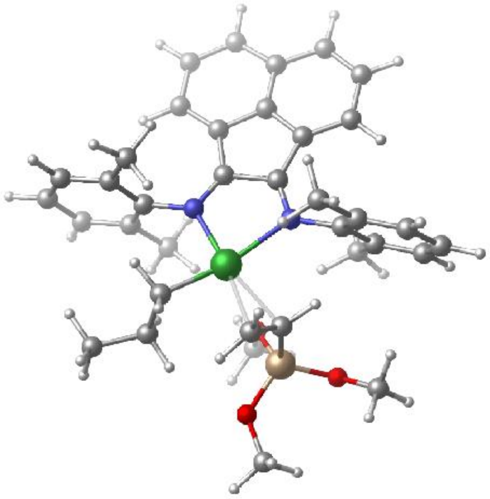

Zero-point correction= 0.717932 (Hartree/Particle)
Thermal correction to Energy= 0.764148
Thermal correction to Enthalpy= 0.765093
Thermal correction to Gibbs Free Energy= 0.635948
Sum of electronic and zero-point Energies= -2191.036132
Sum of electronic and thermal Energies= -2190.989915
Sum of electronic and thermal Enthalpies= -2190.988971
Sum of electronic and thermal Free Energies= -2191.118115

Ni	-1.86638900	3.61766800	-1.47361800
N	-0.50744600	1.95088600	-1.52083400
N	-3.00285600	2.24569000	-0.57062400
C	0.81160300	1.84788100	-2.06644300
C	0.93653800	1.79801400	-3.46705500
C	1.92507800	1.89239300	-1.20602900
C	2.22545100	1.76791300	-4.00870200
C	3.19533700	1.86546300	-1.79542100
C	3.34826900	1.80059800	-3.18089900
H	2.34529900	1.72086800	-5.08764400
H	4.07163400	1.90075900	-1.15369900
H	4.34306600	1.78229500	-3.61595500
C	-4.30239600	2.42502600	0.01393000
C	-5.43608600	2.19965500	-0.78530300
C	-4.38100700	2.83581400	1.35420800
C	-6.69064800	2.40778800	-0.20306300
C	-5.65692100	3.03716900	1.89257700
C	-6.80191500	2.82734500	1.12328300
H	-7.58404300	2.24427600	-0.79931500
H	-5.74824400	3.36118800	2.92572100
H	-7.78330300	2.99255800	1.55776700
C	1.75532500	1.96064100	0.29271900

H	2.68659800	2.27059400	0.77379000
H	0.97370000	2.67007900	0.58034700
H	1.47709300	0.98043300	0.70142100
C	-0.29649900	1.75833100	-4.33657700
H	-0.82084200	0.79878600	-4.24090000
H	-1.01587100	2.53447000	-4.04484600
H	-0.04361100	1.89810900	-5.39100000
C	-3.13228200	2.99985200	2.18637000
H	-2.75978800	2.02344100	2.52545900
H	-2.32588800	3.47295100	1.61797700
H	-3.33290600	3.60307100	3.07639000
C	-5.28178800	1.73465300	-2.21214100
H	-4.58538900	2.37740400	-2.76557200
H	-4.87794700	0.71487600	-2.25663900
H	-6.24205600	1.73760000	-2.73433800
C	-2.43659500	1.09432700	-0.42979500
C	-1.05767900	0.91780100	-0.98934800
C	-0.91631500	5.37024800	-2.71648100
H	-0.97336000	4.98948600	-3.73518600
H	-1.49825500	6.25996300	-2.50799600
C	-0.03672500	4.86874400	-1.80405200
H	0.63420400	4.09291700	-2.16395300
C	-3.36124400	4.88758700	-1.48538300
H	-4.24390400	4.25047600	-1.42182500
H	-3.40950300	5.41507400	-2.44466300
C	-3.30608800	5.84840700	-0.30549200
H	-3.07311600	5.30001600	0.61300600
C	-4.64625800	6.58219600	-0.11873600
H	-5.45236800	5.86883400	0.08910200
H	-4.59147700	7.28714300	0.71888400
H	-4.91826400	7.14675100	-1.01845900
H	-2.49926400	6.57831600	-0.43269600
Si	0.43107300	5.63109700	-0.15546100
O	-0.10044900	7.18319900	-0.05098400
O	2.06300900	5.51556200	0.02412800
O	-0.28159400	4.74434400	1.03491400
C	-0.04029800	5.02771900	2.42106200
H	-0.60917200	4.30507700	3.01070300
H	1.02468300	4.92553600	2.65705100
H	-0.37645100	6.03950600	2.67288600
C	3.05416700	5.64389200	-0.99349700
H	3.04423400	4.77142900	-1.65850200
H	2.91505200	6.55384800	-1.59134900
H	4.02980400	5.69938700	-0.50375900

C	0.64054600	8.34751400	-0.41790800
H	0.11326200	9.21789200	-0.01987800
H	1.65348900	8.32281800	0.00023500
H	0.70388600	8.44420900	-1.50986100
C	-2.84267600	-0.17952600	0.16445300
C	-3.96680500	-0.64219600	0.82798400
C	-1.75904900	-1.07253300	-0.07690500
C	-3.99124800	-1.99828900	1.24348300
H	-4.81260300	0.00418600	1.03119700
C	-1.76986900	-2.42158800	0.32452600
C	-0.66662500	-0.47636400	-0.76701700
C	-2.93691300	-2.86725200	1.00542100
H	-4.87029500	-2.36392300	1.76494400
C	-0.61399200	-3.18528100	0.00053400
C	0.44459800	-1.24328200	-1.07034900
H	-2.99958200	-3.89921900	1.33961700
C	0.44925800	-2.60593800	-0.67563500
H	-0.56790900	-4.23246900	0.28653700
H	1.29643400	-0.82426500	-1.59396100
H	1.31861300	-3.21140800	-0.91220600

UB3LYP-D3/BSII(SMD)//B3LYP-D3/BSI

HF=-2193.9374693

³N

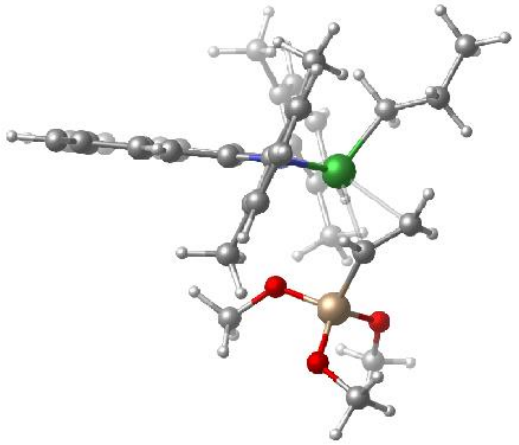

Zero-point correction= 0.714941 (Hartree/Particle)
Thermal correction to Energy= 0.761966
Thermal correction to Enthalpy= 0.762910
Thermal correction to Gibbs Free Energy= 0.630600
Sum of electronic and zero-point Energies= -2191.031407
Sum of electronic and thermal Energies= -2190.984382
Sum of electronic and thermal Enthalpies= -2190.983438
Sum of electronic and thermal Free Energies= -2191.115749

Ni	-2.23360100	3.86584200	-1.10794900
N	-0.80684900	2.44679800	-0.54807700
N	-3.53641400	2.37807600	-0.50083900
C	0.61560800	2.59642700	-0.55667700
C	1.40577000	2.03300400	-1.58015300
C	1.17444900	3.39321700	0.46539700
C	2.78262400	2.29735300	-1.55814500
C	2.55277000	3.61500200	0.45041300
C	3.35507900	3.07721700	-0.55726500
H	3.40607600	1.88019500	-2.34478700
H	2.99924400	4.21488500	1.23864300
H	4.42411700	3.26781200	-0.56083600
C	-4.91727500	2.54828000	-0.20332700
C	-5.77710100	2.94198700	-1.24961200
C	-5.36817200	2.45027000	1.13326700
C	-7.12595600	3.14975900	-0.95540100
C	-6.72623300	2.68601800	1.37874600
C	-7.60365700	3.01577400	0.34831900
H	-7.80431600	3.42927900	-1.75680000
H	-7.09205800	2.62097900	2.39991600
H	-8.65377800	3.18921100	0.56351200
C	0.30360100	3.94053300	1.56812600
H	-0.54375600	4.50164000	1.16439700
H	-0.11834000	3.13168200	2.17865300

H	0.87253800	4.59926600	2.22960500
C	0.83287100	1.15971900	-2.67159200
H	-0.23978200	1.30319800	-2.79896600
H	1.31495500	1.36924100	-3.63175000
H	0.99678700	0.09773900	-2.44844700
C	-4.44373000	2.14671300	2.28985500
H	-4.85209700	2.55636200	3.21788500
H	-4.31302000	1.06787200	2.43668300
H	-3.44846600	2.57872900	2.14297200
C	-5.24689100	3.13377600	-2.64547200
H	-4.68863300	2.26378600	-3.00077800
H	-6.05857700	3.34425700	-3.34740800
H	-4.54954000	3.98210100	-2.69538500
C	-2.89444500	1.28733300	-0.25280700
C	-1.38330700	1.31931300	-0.30092600
C	-1.38577700	5.44572600	-2.68476100
H	-2.32684700	5.88370900	-3.01398300
H	-0.76693000	6.06152200	-2.03638300
C	-0.95734200	4.24689900	-3.15816900
C	-2.91585200	5.23611400	0.21329400
H	-2.88394800	4.71181300	1.17637700
H	-3.96801300	5.29380800	-0.10861900
C	-2.25964800	6.60460300	0.27586900
H	-1.17257900	6.50511200	0.40755300
C	-2.81891000	7.46791200	1.42249700
H	-2.64480600	6.98692700	2.39225700
H	-2.34265900	8.45517300	1.44202700
H	-3.89946700	7.61287200	1.30990300
H	-2.40827400	7.13428200	-0.67377600
H	0.02721200	3.91141200	-2.83300100
Si	-1.84392500	3.20040100	-4.42808100
O	-0.84797200	2.69797900	-5.63559900
O	-3.07707200	4.15258300	-4.95588800
O	-2.35241000	1.82959500	-3.66203800
C	-0.26014800	3.56173900	-6.61359800
H	0.20292800	2.93278400	-7.37750200
H	-1.01394600	4.20216600	-7.08687500
H	0.51122500	4.19578700	-6.15966700
C	-4.05875900	3.75223300	-5.91466000
H	-4.68471200	2.94345200	-5.51872400
H	-4.69185500	4.61714500	-6.12641300
H	-3.59066100	3.41846500	-6.84862100
C	-2.51795800	0.53388800	-4.24364100
H	-1.76158200	0.34416700	-5.01136200

H	-2.41635300	-0.20602200	-3.44401600
H	-3.51666100	0.44062800	-4.68654900
C	-4.48166200	-2.10339600	0.61826600
C	-3.30005800	-2.80781100	0.79485000
C	-2.04225400	-2.16399300	0.63036200
C	-2.07854400	-0.80173400	0.27551300
C	-3.28910500	-0.07423200	0.11391600
C	-4.49847300	-0.72526900	0.28324800
H	-0.66301900	-3.78665800	1.06809200
H	-5.42890400	-2.61696300	0.75025000
H	-3.33113400	-3.85972600	1.06506600
C	-0.75143100	-2.73952900	0.79193300
C	-0.90913900	-0.02017400	0.05996900
H	-5.44173200	-0.20083800	0.17720400
C	0.33308900	-0.60581600	0.23522700
C	0.38953300	-1.97402800	0.60590100
H	1.24955100	-0.04446400	0.09535600
H	1.36343400	-2.43364300	0.74272100

UB3LYP-D3/BSII(SMD)//B3LYP-D3/BSI

HF=-2193.9383111

[1]TS6

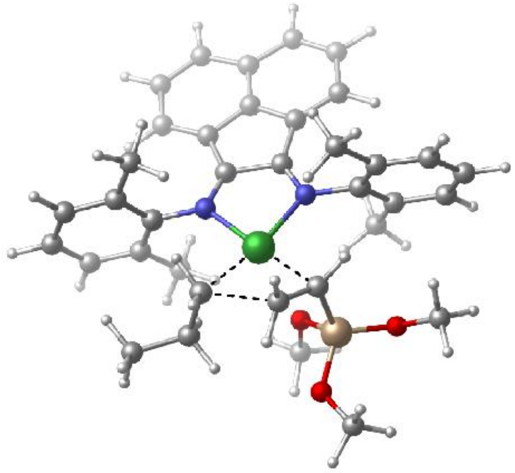

Zero-point correction= 0.718177 (Hartree/Particle)
Thermal correction to Energy= 0.763248
Thermal correction to Enthalpy= 0.764192
Thermal correction to Gibbs Free Energy= 0.639196
Sum of electronic and zero-point Energies= -2191.024481
Sum of electronic and thermal Energies= -2190.979410
Sum of electronic and thermal Enthalpies= -2190.978466
Sum of electronic and thermal Free Energies= -2191.103462

Ni	-1.72648500	3.65649600	-1.40382400
N	-0.47908900	2.06739000	-1.37391400
N	-3.00231800	2.28370200	-0.53428000
C	0.85837900	1.97969400	-1.88968300
C	1.01480100	1.93586600	-3.28837800
C	1.95075500	1.96091700	-1.00387800
C	2.31471600	1.87127000	-3.80050600
C	3.23218600	1.89148700	-1.56511200
C	3.41762300	1.84819900	-2.94618100
H	2.45776000	1.83099600	-4.87686400
H	4.09267500	1.87737400	-0.90165500
H	4.42139700	1.79834200	-3.35770400
C	-4.31756100	2.47238800	-0.00057300
C	-5.42090600	2.34136000	-0.86163700
C	-4.44909100	2.80637200	1.35976400
C	-6.69390600	2.57184000	-0.32691300
C	-5.74028900	3.02779100	1.84912000
C	-6.85404800	2.91512700	1.01528300
H	-7.56317300	2.47652500	-0.97174700
H	-5.86985500	3.29372900	2.89467700
H	-7.84811900	3.09644400	1.41285300
C	1.76128600	1.97017100	0.49348200
H	2.67373400	2.30631500	0.99255700

H	0.94782900	2.63477000	0.79243400
H	1.52834100	0.96278400	0.86290100
C	-0.19303600	1.91408400	-4.19357600
H	-0.76293800	0.98408300	-4.07201600
H	-0.88591800	2.73394800	-3.96344300
H	0.09901200	1.99370100	-5.24409900
C	-3.23956700	2.87738900	2.26240100
H	-2.91986500	1.87168200	2.56654600
H	-2.38518100	3.34713100	1.76417400
H	-3.46492800	3.44043900	3.17250300
C	-5.23036200	1.90031500	-2.29341800
H	-4.43085800	2.46112100	-2.79259800
H	-4.94754100	0.84055900	-2.34184300
H	-6.15017800	2.02672800	-2.87055800
C	-2.42858700	1.14177200	-0.37586100
C	-1.02854100	1.00663700	-0.88712400
C	-1.40757200	5.58873800	-2.47960900
H	-1.67946800	5.45795000	-3.52572700
H	-1.63879700	6.56802000	-2.07639800
C	-0.29562900	4.88844400	-1.95537100
H	0.29985800	4.30728900	-2.65651400
C	-3.26430800	5.09962300	-1.55352600
H	-3.91520100	4.23106000	-1.65676400
H	-3.58381400	5.77814200	-2.34401200
C	-3.36200500	5.76261900	-0.18536900
H	-3.03133900	5.07555100	0.59749300
C	-4.80050700	6.21895700	0.10591700
H	-5.49212000	5.37035000	0.09383700
H	-4.86310800	6.69145000	1.09254300
H	-5.14109600	6.94798000	-0.63893600
H	-2.67906500	6.61938800	-0.13549400
Si	0.49060700	5.49704200	-0.37200200
O	0.12563400	7.09220700	-0.14212000
O	2.10933800	5.20161800	-0.34494900
O	-0.21776400	4.69654200	0.88922600
C	0.02430800	5.06757200	2.25296600
H	-0.69212500	4.52745100	2.87713100
H	1.04118400	4.78827800	2.55152600
H	-0.11538300	6.14435500	2.39418200
C	3.02099400	5.33810400	-1.43237500
H	2.69228200	4.76215000	-2.30564900
H	3.14830500	6.38980700	-1.71752000
H	3.98748300	4.94464800	-1.10864000
C	0.84057500	8.18271300	-0.71859500

H	0.36308500	9.10928400	-0.38952500
H	1.88648000	8.18912900	-0.38891500
H	0.81286500	8.14732900	-1.81704600
C	-2.82876500	-0.15237200	0.17684400
C	-3.96709900	-0.64907300	0.78766800
C	-1.72334900	-1.01952000	-0.05386700
C	-3.98401500	-2.01663100	1.16501900
H	-4.82842300	-0.01912100	0.97930200
C	-1.72690800	-2.37980500	0.30760300
C	-0.61920400	-0.38594600	-0.69365400
C	-2.90830100	-2.86188200	0.93748500
H	-4.87342100	-2.41059600	1.64679100
C	-0.55101100	-3.11787600	-0.00299000
C	0.51052400	-1.13077500	-0.98616100
H	-2.96505900	-3.90384900	1.24021600
C	0.52243700	-2.50409200	-0.63087800
H	-0.49777100	-4.17221900	0.25403400
H	1.37227900	-0.68741700	-1.47164500
H	1.40707300	-3.09024600	-0.85936900

UB3LYP-D3/BSII(SMD)//B3LYP-D3/BSI

HF=-2193.9240474

^{3}TS6

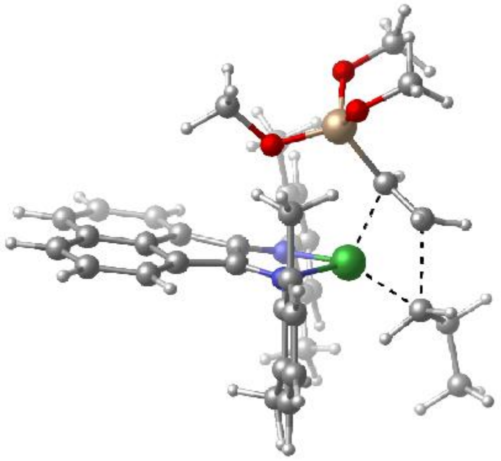

Zero-point correction=		0.714441 (Hartree/Particle)	
Thermal correction to Energy=		0.760599	
Thermal correction to Enthalpy=		0.761543	
Thermal correction to Gibbs Free Energy=		0.631182	
Sum of electronic and zero-point Energies=		-2191.004193	
Sum of electronic and thermal Energies=		-2190.958035	
Sum of electronic and thermal Enthalpies=		-2190.957091	
Sum of electronic and thermal Free Energies=		-2191.087452	
Ni	-2.11413100	3.87478800	-1.00875700
N	-0.78464000	2.43605100	-0.49253900
N	-3.48645300	2.42888300	-0.51027600
C	0.62368500	2.63167600	-0.35558200
C	1.53812400	2.24228900	-1.35281800
C	1.03279500	3.30483000	0.81669300
C	2.89279600	2.52930000	-1.13306100
C	2.39348200	3.56633800	0.98879800
C	3.32122100	3.18238400	0.01992100
H	3.61695300	2.23505000	-1.88823400
H	2.72647300	4.06828100	1.89310700
H	4.37681700	3.39157600	0.16528400
C	-4.86752100	2.59720100	-0.16358700
C	-5.88556900	2.42219100	-1.11522400
C	-5.13658900	2.97934700	1.16661400
C	-7.20429400	2.65622100	-0.70452200
C	-6.46762100	3.21086100	1.52670800
C	-7.49666400	3.05535600	0.59847800
H	-8.00813300	2.51981900	-1.42303400
H	-6.69539200	3.50487800	2.54772500
H	-8.52600700	3.23631700	0.89326800
C	0.02433500	3.68467900	1.87446200
H	-0.77439000	4.31863000	1.46720700

H	-0.46818000	2.80089400	2.29806100
H	0.49960300	4.23231500	2.69267200
C	1.11111300	1.58857500	-2.64460000
H	0.21040700	0.98243600	-2.54460700
H	0.89141000	2.33749000	-3.41304400
H	1.91129000	0.95157400	-3.03424800
C	-4.02540800	3.07565700	2.18614000
H	-4.38688200	3.51276400	3.12072600
H	-3.61495600	2.08411900	2.41828900
H	-3.18435600	3.68216400	1.83008600
C	-5.57516800	1.98938500	-2.52387300
H	-4.83185000	1.18755800	-2.55271900
H	-6.47874100	1.64012700	-3.03179900
H	-5.15754000	2.80629900	-3.11968700
C	-2.88440700	1.29878400	-0.38233900
C	-1.37281200	1.29110500	-0.42147800
C	-2.75188000	5.17409800	-2.67073900
H	-3.80412400	4.97715100	-2.86557600
H	-2.47097100	6.21734400	-2.78342300
C	-1.79836400	4.17004600	-3.04190100
C	-2.97399900	5.78475200	-0.59278100
H	-3.36000900	5.15371400	0.22608200
H	-3.81381700	6.38443600	-0.93877300
C	-1.75428400	6.61027000	-0.19925300
H	-0.84725700	5.98630400	-0.12744700
C	-1.95354700	7.32895900	1.14528800
H	-2.11217000	6.60677200	1.95517800
H	-1.07688200	7.93498500	1.39872700
H	-2.82668400	7.98941500	1.10887500
H	-1.54103100	7.34371100	-0.98661700
H	-0.75911000	4.51509600	-3.07829100
Si	-2.15261500	2.69509600	-4.10567500
O	-0.89558600	2.48092500	-5.15613500
O	-3.60569900	2.87764500	-4.87472200
O	-2.25789400	1.29855500	-3.23050200
C	-0.39092800	3.43977700	-6.07926300
H	0.65261500	3.19124000	-6.29318500
H	-0.95673500	3.41099100	-7.01782300
H	-0.43021800	4.46109000	-5.67505600
C	-3.88282200	3.83585900	-5.89099000
H	-4.96869200	3.93695400	-5.97459800
H	-3.45874800	4.82173800	-5.65298500
H	-3.48645900	3.50469500	-6.85800900
C	-2.42387700	0.02041600	-3.85346900

H	-1.59289500	-0.19151000	-4.53511800
H	-2.43920200	-0.73238000	-3.05979200
H	-3.36867300	-0.02356800	-4.40706600
C	-4.57347700	-2.08013600	0.29941200
C	-3.41466000	-2.83904700	0.37304500
C	-2.13743200	-2.23280500	0.21029400
C	-2.13231900	-0.84668900	-0.03302200
C	-3.32055300	-0.06792000	-0.10583600
C	-4.54939700	-0.68181500	0.06124400
H	-0.80827400	-3.93664600	0.45370500
H	-5.53433200	-2.56819000	0.42954200
H	-3.47869900	-3.90761100	0.55941800
C	-0.86499900	-2.86708200	0.27106400
C	-0.93932600	-0.09040100	-0.21263300
H	-5.47433800	-0.11801800	0.01349300
C	0.28380800	-0.73315500	-0.13971900
C	0.29814400	-2.13116600	0.10319700
H	1.21502100	-0.19308200	-0.26569100
H	1.25676700	-2.63768400	0.15713600

UB3LYP-D3/BSII(SMD)//B3LYP-D3/BSI

HF=-2193.9117833

[1]O

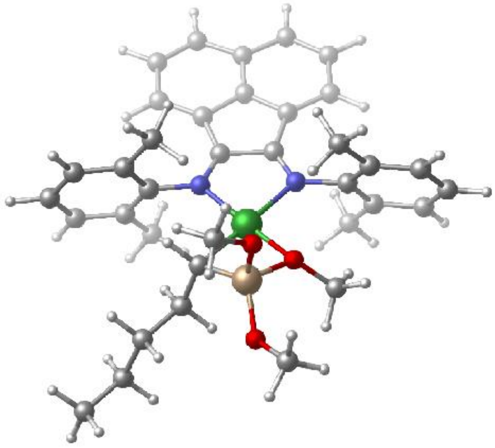

Zero-point correction=	0.719722 (Hartree/Particle)
Thermal correction to Energy=	0.765485
Thermal correction to Enthalpy=	0.766429
Thermal correction to Gibbs Free Energy=	0.635687
Sum of electronic and zero-point Energies=	-2191.071529
Sum of electronic and thermal Energies=	-2191.025766
Sum of electronic and thermal Enthalpies=	-2191.024822
Sum of electronic and thermal Free Energies=	-2191.155564

Ni	-2.04870100	3.79241000	-1.26824700
N	-1.04629700	2.14441700	-1.25364900
N	-3.42232500	2.70935800	-0.22040100
C	0.25157200	1.91718200	-1.81847000
C	0.35323700	1.28116600	-3.06743600
C	1.37035200	2.37555700	-1.10082600
C	1.63512400	1.12243800	-3.60723000
C	2.63049900	2.18963800	-1.67785400
C	2.76361300	1.57329700	-2.92269200
H	1.74311300	0.64235800	-4.57596100
H	3.51233500	2.53258400	-1.14363300
H	3.74911600	1.44359200	-3.35984000
C	-4.66805600	3.15711200	0.31396700
C	-5.86766100	2.78566900	-0.32244500
C	-4.63009800	4.03652400	1.41179500
C	-7.06079100	3.31096500	0.18744000
C	-5.84908500	4.52691200	1.89062300
C	-7.05494700	4.17091900	1.28579800
H	-8.00024400	3.04482300	-0.28938400
H	-5.84773300	5.19764500	2.74549700
H	-7.99086600	4.56794400	1.66742500
C	1.19989800	3.01388400	0.25614300
H	2.14813100	3.41370900	0.62545400
H	0.46587400	3.82934800	0.22777100

H	0.83324600	2.28772200	0.99305400
C	-0.87206900	0.76303700	-3.78307600
H	-1.24703100	-0.15463700	-3.31141700
H	-1.69010800	1.49181600	-3.76655800
H	-0.64513300	0.52975600	-4.82669300
C	-3.31522000	4.42017500	2.04531800
H	-2.76299800	3.53801100	2.39213700
H	-2.67371500	4.94500200	1.32778000
H	-3.47228900	5.07699300	2.90520300
C	-5.86194200	1.87541300	-1.52820700
H	-5.13226800	2.20743300	-2.27721400
H	-5.59749900	0.84554400	-1.25783300
H	-6.84677300	1.84994300	-2.00194800
C	-2.99795400	1.50707200	-0.04258400
C	-1.65580500	1.18798000	-0.63204100
C	-0.83459100	4.60008900	-3.76207900
H	-1.84452900	4.84294300	-4.12110500
H	-0.68096300	3.53315000	-3.96100800
C	-0.71584300	4.85683200	-2.25941400
H	0.29230600	4.62860000	-1.90364000
C	0.19813700	5.39036700	-4.58713900
H	1.20402900	5.16418200	-4.20374100
H	0.03491100	6.46567300	-4.43479400
C	0.13904100	5.06333700	-6.08401300
H	0.29900200	3.98421700	-6.22274500
C	1.17001400	5.84656500	-6.90288600
H	2.19037800	5.62805000	-6.56460100
H	1.10956900	5.59593800	-7.96763400
H	1.01212100	6.92737500	-6.80407200
H	-0.87134200	5.27611600	-6.46101200
Si	-1.49707600	6.37139800	-1.59144400
O	-1.87920900	7.53493100	-2.67994300
O	-0.92541300	6.96816700	-0.16920200
O	-2.93095200	5.53910200	-1.13047900
C	-4.26939200	5.91945200	-1.51320500
H	-4.89890800	5.03200900	-1.47982300
H	-4.64442700	6.65257000	-0.79399300
H	-4.26557600	6.34422500	-2.52085900
C	0.39793500	7.48047300	0.01061800
H	1.15398200	6.74555200	-0.29487500
H	0.54782600	8.40426200	-0.56132100
H	0.52761600	7.69909400	1.07306600
C	-2.33661900	8.86088600	-2.39347900
H	-3.43251500	8.88608100	-2.39517100

H	-1.97287400	9.21131800	-1.42088400
H	-1.96505500	9.52457000	-3.17795100
C	-3.46889600	0.31187600	0.65520700
C	-4.59409900	-0.01375900	1.39131500
C	-2.44447100	-0.66053000	0.47482900
C	-4.68191200	-1.31866200	1.94140800
H	-5.39379100	0.70203700	1.54744100
C	-2.51447900	-1.95788500	1.01508500
C	-1.33565900	-0.19514000	-0.29089900
C	-3.68413100	-2.26594700	1.76512300
H	-5.56276200	-1.57994200	2.51942600
C	-1.40493400	-2.80969000	0.75387400
C	-0.26833800	-1.04523200	-0.52576800
H	-3.79244400	-3.25392500	2.20404000
C	-0.32537600	-2.35771700	0.00894400
H	-1.40566700	-3.82336900	1.14501200
H	0.59263800	-0.72742600	-1.10306400
H	0.50838800	-3.02834600	-0.17372300

UB3LYP-D3/BSII(SMD)//B3LYP-D3/BSI

HF=-2193.9771875

^{3}O

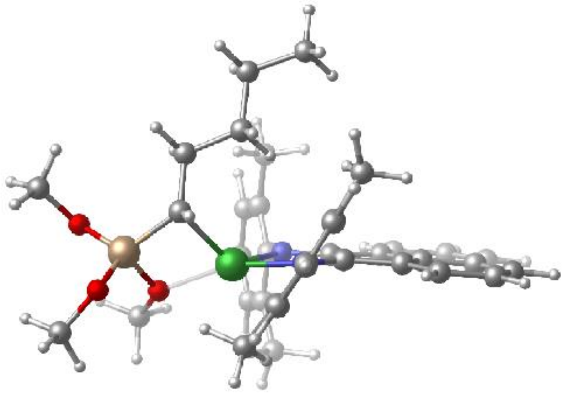

Zero-point correction= 0.718182 (Hartree/Particle)
Thermal correction to Energy= 0.764052
Thermal correction to Enthalpy= 0.764996
Thermal correction to Gibbs Free Energy= 0.634811
Sum of electronic and zero-point Energies= -2191.060442
Sum of electronic and thermal Energies= -2191.014571
Sum of electronic and thermal Enthalpies= -2191.013627
Sum of electronic and thermal Free Energies= -2191.143813

Ni	-1.97719300	3.02668000	-1.35027000
N	-0.57042800	2.11543700	-0.19746300
N	-3.24031100	2.41943000	0.17859000
C	0.79927800	1.87762400	-0.54222500
C	1.08453200	0.77630200	-1.36992400
C	1.79103800	2.75864500	-0.08383800
C	2.41838800	0.56417200	-1.73481800
C	3.11239200	2.50395300	-0.46901800
C	3.42656800	1.41836000	-1.28671600
H	2.66386400	-0.27930500	-2.37448700
H	3.89903400	3.16912400	-0.12362100
H	4.45724900	1.23969900	-1.57794300
C	-4.59667700	2.81053300	0.28608500
C	-5.40121000	2.61283000	-0.86316100
C	-5.09398900	3.48121900	1.42989000
C	-6.73514300	3.01353700	-0.81805100
C	-6.43612500	3.88503000	1.41167200
C	-7.25616000	3.64241300	0.31439700
H	-7.36973800	2.84005200	-1.68262400
H	-6.83316500	4.40967000	2.27671900
H	-8.29485500	3.95841800	0.33123900
C	1.42996400	3.92740400	0.79766000
H	0.72588200	4.59542300	0.29160600
H	0.94579600	3.60124900	1.72651600
H	2.31646000	4.50767100	1.06664400

C	-0.02197600	-0.13935400	-1.83664600
H	-0.45242300	-0.70818700	-1.00276400
H	-0.84410800	0.42748700	-2.29377300
H	0.34591400	-0.85713900	-2.57457500
C	-4.25213900	3.83632800	2.63372000
H	-4.53066000	4.82758300	3.00405700
H	-4.40212500	3.12825300	3.45742000
H	-3.18363400	3.85642100	2.41415300
C	-4.83472200	1.99427200	-2.12044100
H	-4.16978100	1.14913000	-1.91318500
H	-5.63518900	1.63578700	-2.77415300
H	-4.27143200	2.73933300	-2.70455500
C	-2.59914800	1.69871700	1.03882100
C	-1.11512000	1.51656900	0.80797900
C	-1.60323900	5.99436600	-0.76686700
H	-1.87421000	7.04000200	-0.98926800
H	-0.52927900	5.91509900	-0.98757300
C	-2.39867400	5.04519600	-1.65700900
C	-1.83875800	5.74248400	0.72817400
H	-1.55435200	4.70473800	0.95416800
H	-2.91590600	5.81250300	0.93339300
C	-1.07294800	6.69276000	1.65354300
H	-0.00155700	6.65373200	1.41118800
C	-1.27465500	6.35823600	3.13500800
H	-0.93055300	5.33945100	3.35813100
H	-0.72071700	7.04563400	3.78307900
H	-2.33413200	6.41758500	3.41281300
H	-1.39154300	7.72572900	1.45884600
H	-3.47836200	5.13016400	-1.48387700
Si	-1.89711800	4.84205900	-3.40839600
O	-0.66046700	5.73878300	-4.02152600
O	-3.21685000	4.79626800	-4.39372200
O	-1.21441000	3.27779900	-3.29307500
C	-0.75944300	7.13279400	-4.32877800
H	0.14845900	7.42111000	-4.86395500
H	-1.62840900	7.34311800	-4.96419300
H	-0.83417400	7.72814200	-3.41081400
C	-3.18107600	4.41508400	-5.77089400
H	-2.98066200	3.34023600	-5.87096100
H	-4.15968100	4.63293600	-6.20537100
H	-2.41547100	4.97338100	-6.32361500
C	-0.20914700	2.69774600	-4.14699100
H	0.55628700	2.24180900	-3.51640000
H	-0.67043000	1.93548000	-4.78235100

H	0.24562500	3.47639200	-4.76361500
C	-4.09300900	-0.35777600	3.92682600
C	-2.90889600	-0.93351200	4.36108300
C	-1.68053100	-0.63794700	3.70696600
C	-1.74705600	0.26519100	2.62955600
C	-2.95653100	0.88182000	2.20067700
C	-4.13876000	0.55705400	2.84350100
H	-0.28296100	-1.86069900	4.83629600
H	-5.02030300	-0.60861500	4.43235600
H	-2.91651300	-1.62309300	5.20058600
C	-0.39394700	-1.16571500	4.00845900
C	-0.61435400	0.61733500	1.84408500
H	-5.08601100	0.98472400	2.53432600
C	0.62469300	0.08155800	2.15345100
C	0.71397600	-0.80820800	3.25392400
H	1.50801600	0.32488300	1.57392600
H	1.68285300	-1.22847400	3.50473300

UB3LYP-D3/BSII(SMD)//B3LYP-D3/BSI

HF=-2193.9695596

[1]P

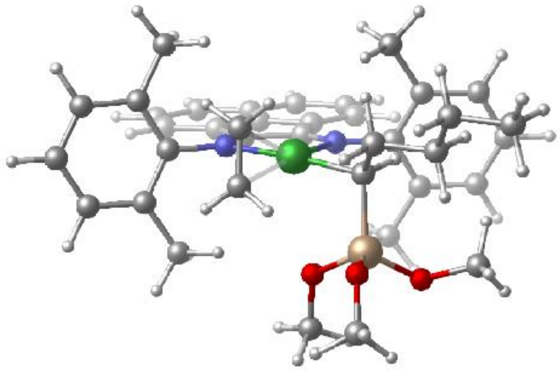

Zero-point correction= 0.776535 (Hartree/Particle)
Thermal correction to Energy= 0.824987
Thermal correction to Enthalpy= 0.825932
Thermal correction to Gibbs Free Energy= 0.693109
Sum of electronic and zero-point Energies= -2269.622258
Sum of electronic and thermal Energies= -2269.573805
Sum of electronic and thermal Enthalpies= -2269.572861
Sum of electronic and thermal Free Energies= -2269.705683

Ni	-0.40050300	-0.83562000	-0.50129600
N	0.18159800	1.09531100	-0.32017800
N	1.68369300	-1.13757800	-0.34590700
C	-0.65456600	2.26014900	-0.31077800
C	-1.15829700	2.71302700	-1.54372500
C	-1.00069900	2.85040900	0.91721000
C	-2.01758300	3.81551700	-1.53094200
C	-1.86844700	3.94844800	0.88005400
C	-2.37018200	4.43088800	-0.32880400
H	-2.41614700	4.18676200	-2.47101800
H	-2.15657200	4.42104900	1.81506300
H	-3.04411600	5.28244400	-0.33394900
C	2.41478000	-2.36705800	-0.34477200
C	3.01823500	-2.80820100	-1.53639100
C	2.45806600	-3.10822700	0.84958000
C	3.67381600	-4.04467500	-1.51085900
C	3.12773000	-4.33647900	0.82715200
C	3.72777200	-4.80465500	-0.34214100
H	4.14485200	-4.41076000	-2.41909000
H	3.17739900	-4.92709400	1.73792600
H	4.23812900	-5.76307700	-0.34295500
C	-0.44922400	2.32647400	2.22010400
H	-1.04846500	2.67883100	3.06318000
H	-0.44953100	1.23390600	2.23732400
H	0.58398200	2.66420700	2.37532600

C	-0.78053000	2.00543200	-2.82013200
H	-1.00318200	0.93239100	-2.74787800
H	-1.32346900	2.41464900	-3.67609100
H	0.29360500	2.09218300	-3.02674100
C	1.83720400	-2.55713200	2.11094900
H	2.42579900	-1.71544600	2.50035400
H	0.82443500	-2.17766100	1.93497500
H	1.79369800	-3.32038700	2.89300700
C	2.98707900	-1.95524500	-2.78309300
H	1.98272300	-1.56521500	-2.98780700
H	3.64865700	-1.08501400	-2.68247800
H	3.31801600	-2.52372700	-3.65622100
C	2.29614300	-0.01834300	-0.18675600
C	1.45875000	1.22974300	-0.18934200
C	-3.23754100	-0.94626600	-1.50320400
H	-3.37893000	-2.02602900	-1.38134100
H	-2.78636300	-0.78995800	-2.49422300
C	-2.31037100	-0.37317100	-0.42620200
H	-2.29991900	0.71322700	-0.54955300
C	-4.62524100	-0.27662800	-1.50863100
H	-5.12103600	-0.46942200	-0.54816300
Si	-2.74943900	-0.72012300	1.35690000
O	-3.79656700	-2.00119800	1.34033300
O	-3.35502300	0.57344700	2.19379900
O	-1.34839700	-1.09082200	2.18500700
C	-1.23066500	-1.05175900	3.60953100
H	-0.16621800	-1.08015700	3.86032000
H	-1.67292000	-0.13663500	4.01648800
H	-1.71989100	-1.92173900	4.06449500
C	-4.43268900	1.40023300	1.76246500
H	-4.17608900	1.92907100	0.83628900
H	-5.34893000	0.81900400	1.60121400
H	-4.62054700	2.13927600	2.54626000
C	-4.40476000	-2.57372900	2.49779900
H	-3.69787400	-3.22894400	3.02271000
H	-4.75743200	-1.80102100	3.19226800
H	-5.26033700	-3.17118100	2.17140600
C	3.68999500	0.38473700	0.00180000
C	4.89685800	-0.28478400	0.10246600
C	3.66863900	1.80555900	0.07782500
C	6.07707900	0.48279100	0.27850300
H	4.95091700	-1.36638900	0.04783600
C	4.83026500	2.58223400	0.24615000
C	2.36453900	2.36824000	-0.03441400

C	6.05687200	1.86790100	0.34747500
H	7.02670100	-0.03684000	0.35919000
C	4.64407000	3.99186900	0.29452900
C	2.21444000	3.74372500	0.01587700
H	6.98522600	2.41673900	0.47960800
C	3.37568700	4.54150500	0.17982100
H	5.50586100	4.64105500	0.42265200
H	1.23950100	4.21069800	-0.06381900
H	3.26157900	5.62028300	0.21881100
H	-4.50146800	0.81364600	-1.58807200
C	-0.92477300	-3.11197300	-0.38746100
H	-1.94548800	-3.12871600	-0.01871500
H	-0.15299500	-3.46551100	0.28894400
C	-0.62760400	-2.81036100	-1.68024900
H	0.38180100	-2.93770300	-2.05809400
H	-1.39959400	-2.57745400	-2.40488100
C	-5.52429600	-0.77362700	-2.64729200
H	-5.02886200	-0.58325400	-3.61004600
H	-5.63590500	-1.86397600	-2.56820800
C	-6.90609700	-0.11208300	-2.63838400
H	-7.53109700	-0.48062500	-3.45895500
H	-7.43395000	-0.31562900	-1.69864000
H	-6.82320300	0.97674500	-2.74383300

UB3LYP-D3/BSII(SMD)//B3LYP-D3/BSI

HF=-2272.6060627

^{3}P

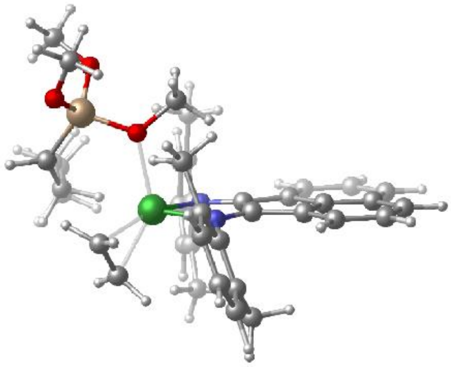

Zero-point correction= 0.772440 (Hartree/Particle)
Thermal correction to Energy= 0.821704
Thermal correction to Enthalpy= 0.822648
Thermal correction to Gibbs Free Energy= 0.686589
Sum of electronic and zero-point Energies= -2269.607721
Sum of electronic and thermal Energies= -2269.558457
Sum of electronic and thermal Enthalpies= -2269.557513
Sum of electronic and thermal Free Energies= -2269.693572

Ni	-1.52095000	3.80846800	-1.01059500
N	-0.41394200	2.10425000	-0.55620800
N	-3.11000700	2.50246300	-0.64255200
C	0.96813300	2.08742800	-0.20941000
C	1.92000700	2.36562300	-1.21129800
C	1.33831000	1.92643100	1.14500800
C	3.26737900	2.42440100	-0.84631200
C	2.69964500	2.00868900	1.46172900
C	3.65961400	2.24449000	0.47991200
H	4.01327300	2.61991300	-1.61182000
H	3.00384600	1.89435300	2.49871400
H	4.70986600	2.30242200	0.74939800
C	-4.50917400	2.77907500	-0.47232600
C	-5.40304900	2.70561600	-1.55457200
C	-4.93089500	3.12264500	0.82668600
C	-6.75735000	2.95307400	-1.29804800
C	-6.29244000	3.36893500	1.03328500
C	-7.20423000	3.27609700	-0.01746500
H	-7.46616700	2.89054000	-2.11917800
H	-6.63544500	3.63042200	2.03062900
H	-8.25857900	3.46721400	0.15753400
C	0.32346400	1.69759500	2.24231500
H	0.03789800	0.64117500	2.31554800
H	0.73512600	1.99338400	3.21124600
C	1.49017700	2.56786700	-2.64164200

H	1.04877900	1.65441100	-3.05728000
H	0.73131700	3.35505000	-2.72024200
H	2.34108400	2.84576400	-3.27010100
C	-3.93798000	3.18392300	1.96180800
H	-3.05889200	3.77749800	1.68559400
H	-4.38885300	3.62125500	2.85663400
H	-3.57292500	2.18275200	2.22679100
C	-4.92947300	2.36294900	-2.94407400
H	-4.29066100	1.47312500	-2.94610900
H	-5.77729200	2.16807900	-3.60669900
H	-4.34946800	3.18284200	-3.37978600
C	-2.67690600	1.29931800	-0.47736600
C	-1.18162300	1.07873700	-0.40793500
C	-3.57627200	6.33079100	-1.82967600
H	-3.42215300	7.06004200	-1.02305100
H	-3.55029000	5.33369900	-1.36080900
C	-2.51594000	6.48357900	-2.87805900
C	-5.01059100	6.50914000	-2.39467700
H	-5.06520400	7.44559900	-2.96553400
H	-2.12573300	7.48950600	-3.02947500
Si	-1.99569100	5.18913700	-4.06137900
O	-0.62759800	5.72711400	-4.79313300
O	-3.14053900	4.70217200	-5.14971300
O	-1.72701700	3.75593800	-3.20211200
C	0.09217800	5.03977300	-5.81675600
H	0.78943000	4.31897600	-5.37141000
H	-0.58150300	4.51121900	-6.50292600
H	0.66513100	5.77859000	-6.38275200
C	-3.77247700	5.57858300	-6.08685100
H	-3.03510900	6.07518900	-6.72917200
H	-4.43591400	4.97523700	-6.71129600
H	-4.36740400	6.34085000	-5.56912900
C	-1.66237200	2.48864400	-3.88647800
H	-0.74488100	2.42606500	-4.47829700
H	-1.65037200	1.70640600	-3.12662100
H	-2.52802500	2.36262900	-4.53806500
C	-4.85973800	-1.80648900	0.14924200
C	-3.82542100	-2.71100000	0.33431400
C	-2.46967500	-2.28577500	0.25705300
C	-2.25907100	-0.92044900	-0.01434900
C	-3.31927900	0.00769100	-0.21137700
C	-4.62864900	-0.43336600	-0.12599700
H	-1.40654900	-4.14372400	0.63583700
H	-5.88554900	-2.15552000	0.21568300

H	-4.04792200	-3.75402300	0.54216400
C	-1.30346300	-3.08257500	0.42625700
C	-0.96522400	-0.33799400	-0.10603500
H	-5.46542900	0.24145600	-0.26510700
C	0.15209900	-1.13620100	0.06634500
C	-0.04057400	-2.51663700	0.32894300
H	1.15420100	-0.72453000	0.01310000
H	0.83324300	-3.14661900	0.46319100
H	-5.21067100	5.69454900	-3.10418900
C	-0.89309200	5.14203500	0.50957000
H	-0.51455900	4.51519300	1.31308900
H	-1.75320100	5.76458300	0.75063200
C	-0.13888000	5.38225400	-0.61990600
H	-0.38524600	6.19589000	-1.29762100
H	0.84433000	4.93309200	-0.74094900
H	-0.59713200	2.26803400	2.07794800
C	-6.06558300	6.50986600	-1.28268500
H	-5.90599700	7.38584300	-0.63892500
H	-5.92089600	5.62666300	-0.64951800
C	-7.49604200	6.51868300	-1.82703700
H	-7.68546800	5.62804700	-2.43834100
H	-8.22916200	6.52590500	-1.01296100
H	-7.68064800	7.40055900	-2.45204800

UB3LYP-D3/BSII(SMD)//B3LYP-D3/BSI

HF=-2272.5906992

[1]TS7

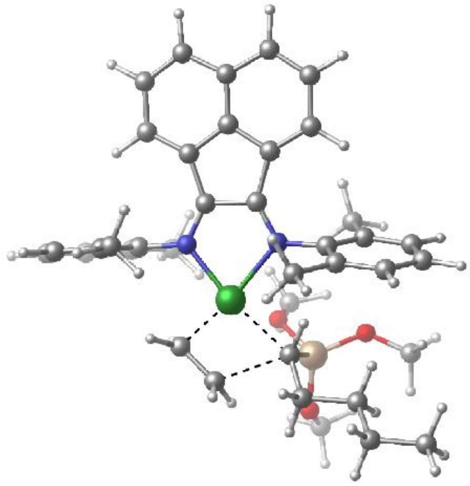

Zero-point correction= 0.776152 (Hartree/Particle)
Thermal correction to Energy= 0.823917
Thermal correction to Enthalpy= 0.824862
Thermal correction to Gibbs Free Energy= 0.693306
Sum of electronic and zero-point Energies= -2269.596861
Sum of electronic and thermal Energies= -2269.549095
Sum of electronic and thermal Enthalpies= -2269.548151
Sum of electronic and thermal Free Energies= -2269.679706

Ni	-0.10019400	-1.06816900	-0.61201400
N	0.12423300	1.03606400	-0.30897700
N	1.87442100	-0.98824200	-0.41392500
C	-0.89393900	2.03540200	-0.27301800
C	-1.47229500	2.41841100	-1.49882000
C	-1.36759700	2.50291300	0.96726600
C	-2.53176400	3.33034500	-1.46575900
C	-2.43472500	3.40804000	0.95009400
C	-3.01022400	3.82354800	-0.25127000
H	-2.98670400	3.64719100	-2.40020900
H	-2.82050600	3.78181900	1.89461100
H	-3.83857200	4.52580100	-0.24058000
C	2.78037000	-2.09832700	-0.49046100
C	3.37987200	-2.39417400	-1.72510400
C	2.99349900	-2.86572900	0.66535400
C	4.24516600	-3.49278300	-1.77694400
C	3.86733800	-3.95396900	0.56794200
C	4.49209500	-4.26413600	-0.64064200
H	4.72383800	-3.74413400	-2.71952400
H	4.05283500	-4.56310300	1.44847900
H	5.16534600	-5.11409000	-0.69956200
C	-0.74708900	2.03781600	2.26129100
H	-1.40080400	2.26344300	3.10688600

H	-0.57674600	0.95788400	2.24620200
H	0.21935300	2.52734700	2.43753300
C	-0.95068200	1.84670800	-2.79358000
H	-0.93544000	0.74845300	-2.76085800
H	-1.56825200	2.15756800	-3.64037100
H	0.08012300	2.16713700	-2.98914200
C	2.29974400	-2.50224800	1.95472000
H	2.68754300	-1.55984200	2.36396300
H	1.22454900	-2.35965500	1.79490800
H	2.44212800	-3.27831300	2.71174200
C	3.09777200	-1.54012200	-2.93705200
H	2.01871600	-1.40088400	-3.08215600
H	3.53480100	-0.53864900	-2.83182100
H	3.51200200	-1.99122200	-3.84254600
C	2.34008800	0.18864900	-0.16176400
C	1.36076500	1.32505000	-0.10524200
C	-3.49389900	-1.05424800	-1.60016600
H	-3.93390200	-2.05688700	-1.56490400
H	-3.09164700	-0.91176300	-2.61347100
C	-2.35628000	-0.89504000	-0.58495900
H	-2.00272900	0.12686200	-0.71039500
C	-4.61193000	-0.01836600	-1.37647700
H	-5.14614100	-0.25823000	-0.44855600
Si	-2.72251800	-1.21716500	1.21769900
O	-3.79339600	-2.47372600	1.22037200
O	-3.27825100	0.05655700	2.10977400
O	-1.26807200	-1.57765200	1.93965000
C	-1.07126100	-1.59242300	3.35699100
H	-0.00280400	-1.46575400	3.55185000
H	-1.62229800	-0.78038700	3.84212100
H	-1.39263300	-2.55259500	3.77816300
C	-4.57283000	0.64744400	2.10614400
H	-4.63933000	1.40998900	1.32254100
H	-5.36234600	-0.09803500	1.95103100
H	-4.72760000	1.12401700	3.07885900
C	-4.30235600	-3.11370100	2.39084500
H	-3.55526700	-3.79676500	2.81376900
H	-4.59190100	-2.38414100	3.15788700
H	-5.18498500	-3.69144800	2.10430000
C	3.67285300	0.73697900	0.08506900
C	4.95145300	0.21233200	0.16375600
C	3.47954300	2.13676100	0.26605300
C	6.02648200	1.10122300	0.42346800
H	5.13865900	-0.84725300	0.03110800

C	4.53513300	3.03213200	0.52002000
C	2.12056900	2.54708800	0.16300200
C	5.83786500	2.46444100	0.59618400
H	7.03099800	0.69460300	0.48667900
C	4.17760700	4.40123900	0.67020300
C	1.80146000	3.88471100	0.31359500
H	6.69098300	3.10845000	0.79117100
C	2.85372100	4.80209100	0.56701800
H	4.95202800	5.13725300	0.86796700
H	0.77662400	4.23263800	0.24462600
H	2.60877400	5.85297400	0.68543100
H	-4.17226400	0.97934500	-1.23930500
C	-0.07741300	-3.01256600	-0.99119500
H	0.25440200	-3.50550800	-0.07885200
H	0.59912300	-3.10570500	-1.84010900
C	-1.45339800	-2.85243300	-1.22943000
H	-1.79176000	-2.72677900	-2.25132800
H	-2.18221900	-3.26790000	-0.54310200
C	-5.61955900	0.02874500	-2.53213700
H	-5.08909900	0.27367400	-3.46319800
H	-6.05458700	-0.96928000	-2.67885700
C	-6.73456100	1.05093500	-2.28982600
H	-7.43907800	1.08123900	-3.12780500
H	-7.30316600	0.80749300	-1.38396400
H	-6.32174700	2.05957400	-2.16171300

UB3LYP-D3/BSII(SMD)//B3LYP-D3/BSI

HF=-2272.5779061

[3]TS7

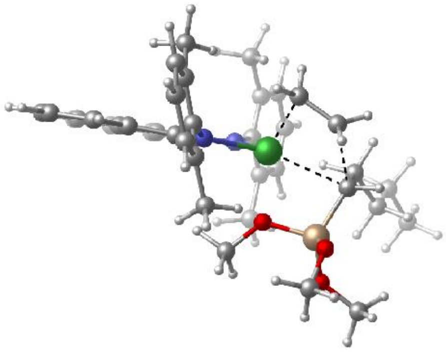

Zero-point correction= 0.773454 (Hartree/Particle)
Thermal correction to Energy= 0.821553
Thermal correction to Enthalpy= 0.822497
Thermal correction to Gibbs Free Energy= 0.689802
Sum of electronic and zero-point Energies= -2269.584138
Sum of electronic and thermal Energies= -2269.536040
Sum of electronic and thermal Enthalpies= -2269.535095
Sum of electronic and thermal Free Energies= -2269.667791

Ni	-1.63911800	3.76536500	-1.07092600
N	-0.44999100	2.07487100	-0.69342500
N	-3.15165000	2.44265600	-0.52698200
C	0.95361500	2.07656200	-0.43590100
C	1.84394600	2.27983300	-1.50751200
C	1.40253300	1.99552100	0.90210700
C	3.21203100	2.33504800	-1.22892000
C	2.78131700	2.07297200	1.13126800
C	3.68230200	2.22833100	0.07965100
H	3.91228700	2.47045100	-2.04859500
H	3.14637700	2.01944100	2.15334800
H	4.74799700	2.28258600	0.28070100
C	-4.53971200	2.69768400	-0.25649900
C	-5.47708000	2.69030400	-1.30340900
C	-4.90184300	2.97036400	1.07608300
C	-6.81366300	2.95668200	-0.98359900
C	-6.24694300	3.24313300	1.34589000
C	-7.19917200	3.23452400	0.32737300
H	-7.55464200	2.95211600	-1.77834200
H	-6.54599900	3.45893300	2.36796500
H	-8.23975800	3.44619400	0.55411100
C	0.44703300	1.86172000	2.06588600
H	0.11823400	0.82465100	2.20466400
H	0.92904700	2.17981700	2.99430500
C	1.32880300	2.41062400	-2.91722400
H	0.81223300	1.49923300	-3.23979200

H	0.61008800	3.23309100	-3.00012300
H	2.14828700	2.59882300	-3.61655100
C	-3.87181600	2.93389400	2.17868800
H	-2.97963700	3.50840200	1.90944200
H	-4.27765500	3.33858800	3.10957000
H	-3.54086600	1.90611300	2.37843100
C	-5.05719600	2.41080200	-2.72349400
H	-4.50435800	1.46783600	-2.80305100
H	-5.92672500	2.34863400	-3.38320800
H	-4.40148300	3.20057300	-3.10277300
C	-2.68405800	1.24968800	-0.37783800
C	-1.18880900	1.05057900	-0.43212900
C	-3.78696400	5.98804300	-1.66520700
H	-3.80980500	6.62131400	-0.76678600
H	-4.05993900	4.98428200	-1.31956400
C	-2.38114200	6.01111500	-2.25589000
C	-4.88844000	6.46777000	-2.63161200
H	-4.59494900	7.43274800	-3.07007200
H	-2.01676900	7.03212500	-2.38924500
Si	-1.94057300	5.04127200	-3.75789300
O	-0.46102300	5.56816300	-4.25161900
O	-2.98821200	4.91054500	-5.02616500
O	-1.87856300	3.45500300	-3.18480200
C	0.21499000	5.15811500	-5.44132700
H	0.89074000	4.32369400	-5.21963000
H	-0.48724400	4.84915700	-6.22526700
H	0.80591900	6.00195300	-5.80730500
C	-3.30955500	5.96319100	-5.93978600
H	-2.41828000	6.53180600	-6.23204100
H	-3.74758500	5.50910900	-6.83209600
H	-4.03941300	6.64751200	-5.49321900
C	-1.90728900	2.26810500	-3.98791100
H	-1.01937300	2.21505900	-4.62458200
H	-1.91397500	1.41301700	-3.30882300
H	-2.80451000	2.25861900	-4.61033600
C	-4.76577900	-1.86165400	0.50288300
C	-3.70555800	-2.74454200	0.64302400
C	-2.36588500	-2.30405000	0.45307600
C	-2.19795500	-0.94644800	0.12150900
C	-3.28459600	-0.04027600	-0.02587600
C	-4.57755600	-0.49555400	0.16780400
H	-1.24651100	-4.13199600	0.81749200
H	-5.77819800	-2.22293900	0.65427500
H	-3.89565500	-3.78261600	0.90162100

C	-1.17712000	-3.07778300	0.56384200
C	-0.92493700	-0.34896700	-0.08788700
H	-5.43244600	0.16341400	0.06796100
C	0.21510000	-1.12469100	0.02812600
C	0.06515400	-2.49725000	0.35392300
H	1.20352100	-0.70192500	-0.11517700
H	0.95714300	-3.10912400	0.44561100
H	-4.96890600	5.75742600	-3.46510500
C	-0.85657300	4.95708900	0.41121300
H	0.12293700	4.49639200	0.53384700
H	-1.48320800	4.96414000	1.29997700
C	-0.98176700	6.03367600	-0.50464700
H	-1.62374600	6.86298400	-0.23207100
H	-0.12761500	6.29442200	-1.12334100
H	-0.45107500	2.47254100	1.92448100
C	-6.25369000	6.60831300	-1.94623500
H	-6.18392200	7.36911400	-1.15651800
H	-6.50191900	5.66446800	-1.44340300
C	-7.36953700	6.97982900	-2.92738700
H	-7.48356700	6.21161400	-3.70266900
H	-8.33279900	7.08439700	-2.41619400
H	-7.15511200	7.93003300	-3.43150200

UB3LYP-D3/BSII(SMD)//B3LYP-D3/BSI

HF=-2272.5707502

^{1}Q

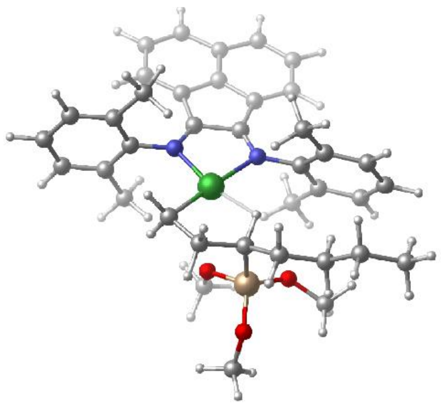

Zero-point correction= 0.777072 (Hartree/Particle)
Thermal correction to Energy= 0.824852
Thermal correction to Enthalpy= 0.825796
Thermal correction to Gibbs Free Energy= 0.693977
Sum of electronic and zero-point Energies= -2269.625432
Sum of electronic and thermal Energies= -2269.577652
Sum of electronic and thermal Enthalpies= -2269.576707
Sum of electronic and thermal Free Energies= -2269.708526

Ni	0.00738700	-0.94620800	0.64859100
N	-0.15127400	1.07318800	0.32294600
N	-1.87891500	-0.95394700	0.43159500
C	0.89995600	2.03659300	0.30217300
C	1.48322900	2.38032200	1.53675700
C	1.39004100	2.51640900	-0.92668800
C	2.55206400	3.28105800	1.52657500
C	2.47134100	3.40386600	-0.88705400
C	3.04286900	3.79256000	0.32477700
H	3.00895500	3.57148500	2.46846200
H	2.87133600	3.78566500	-1.82246900
H	3.87952600	4.48483400	0.33247000
C	-2.74594200	-2.08983100	0.53850200
C	-3.25852100	-2.41744500	1.80339600
C	-3.00457200	-2.84696000	-0.61445600
C	-4.09484800	-3.53543900	1.89002500
C	-3.84589300	-3.95644700	-0.48092600
C	-4.39188900	-4.29539300	0.75796300
H	-4.50910300	-3.81222300	2.85561100
H	-4.06577400	-4.56130100	-1.35639400
H	-5.04117100	-5.16158100	0.84366200
C	0.79125700	2.06334300	-2.23532900
H	1.43817200	2.34054800	-3.07152200
H	0.67256000	0.97580400	-2.25059700
H	-0.19369000	2.51378900	-2.40976200

C	0.94917000	1.79981800	2.82325000
H	0.92028200	0.70209100	2.78433800
H	1.56916200	2.09210000	3.67488100
H	-0.07788500	2.13042100	3.02128800
C	-2.38268100	-2.45933800	-1.93271700
H	-2.79957200	-1.51710500	-2.31231100
H	-1.30211600	-2.31274500	-1.82023900
H	-2.55366900	-3.22896100	-2.69019600
C	-2.90503900	-1.57996600	3.00673600
H	-1.81709400	-1.45532500	3.08836300
H	-3.33552600	-0.57260300	2.93702400
H	-3.27144500	-2.03790000	3.92916100
C	-2.35201700	0.21091900	0.12963800
C	-1.37885600	1.35583500	0.05796100
C	3.62169100	-1.12832700	1.66160700
H	4.24949300	-2.02795200	1.69502200
H	3.25026300	-0.97410500	2.68491200
C	2.39573700	-1.44115700	0.76339100
H	1.77018000	-0.52286800	0.93623100
C	4.45962900	0.08427900	1.23534700
H	5.11773900	-0.20241800	0.40590000
Si	2.79075600	-1.48755600	-1.08317500
O	4.02941400	-2.55830200	-1.23563500
O	3.15455600	-0.02310400	-1.74528900
O	1.38709600	-1.94328100	-1.84588500
C	1.20078400	-1.81564200	-3.25833800
H	0.12664300	-1.81608300	-3.46133800
H	1.63462700	-0.88212800	-3.63356700
H	1.65497800	-2.66318200	-3.78522700
C	4.41420400	0.52661100	-2.11107300
H	4.63319600	1.38714300	-1.47135200
H	5.22412400	-0.20567600	-2.01566200
H	4.35989100	0.86132900	-3.15272800
C	4.44535600	-3.19983100	-2.44065100
H	3.69414200	-3.92639200	-2.77320500
H	4.62320500	-2.47516500	-3.24620800
H	5.37948200	-3.72845400	-2.23483100
C	-3.68221300	0.73591900	-0.15713200
C	-4.95452100	0.19607700	-0.23443000
C	-3.49685000	2.13101500	-0.38303000
C	-6.03300400	1.06584800	-0.53875100
H	-5.13186100	-0.86075200	-0.06842700
C	-4.55652100	3.00716600	-0.68109100
C	-2.14310300	2.55791100	-0.27184700

C	-5.85286600	2.42436000	-0.75577200
H	-7.03330300	0.64903100	-0.60151000
C	-4.20877400	4.37415500	-0.87101700
C	-1.83360900	3.89274000	-0.46124300
H	-6.70886100	3.05326900	-0.98449100
C	-2.89023100	4.79101500	-0.76102400
H	-4.98657400	5.09597000	-1.10446200
H	-0.81249700	4.25117600	-0.38747100
H	-2.65332300	5.83978100	-0.91024100
H	3.80437400	0.87749000	0.85469100
C	0.23114600	-2.80638700	0.91904700
H	0.01113700	-3.28888500	-0.03714500
H	-0.43011300	-3.18033700	1.70606300
C	1.70488900	-2.75775500	1.28576700
H	1.81025300	-2.76847200	2.37667900
H	2.27344300	-3.61465000	0.90177200
C	5.31434100	0.65360700	2.37335100
H	4.65093100	0.98476400	3.18556600
H	5.94441400	-0.14067500	2.79641800
C	6.18996300	1.82340400	1.91398200
H	6.77606200	2.23694000	2.74166200
H	6.89225300	1.50674800	1.13318600
H	5.57383900	2.63050400	1.49848000

UB3LYP-D3/BSII(SMD)//B3LYP-D3/BSI

HF=-2272.6090285

^{3}Q

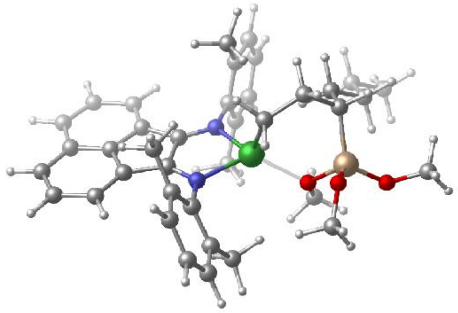

Zero-point correction= 0.775629 (Hartree/Particle)
Thermal correction to Energy= 0.823757
Thermal correction to Enthalpy= 0.824701
Thermal correction to Gibbs Free Energy= 0.690983
Sum of electronic and zero-point Energies= -2269.637589
Sum of electronic and thermal Energies= -2269.589462
Sum of electronic and thermal Enthalpies= -2269.588518
Sum of electronic and thermal Free Energies= -2269.722235

Ni	-1.80472200	3.28373300	-1.42717500
N	-0.59637200	1.71412000	-0.90146800
N	-3.25042100	2.10394600	-0.58631800
C	0.81615200	1.72073600	-1.06729700
C	1.30323200	1.66470500	-2.39051800
C	1.67193200	1.89681900	0.04010800
C	2.68407400	1.72821700	-2.58734200
C	3.04804600	1.97114300	-0.21176300
C	3.55420800	1.87864800	-1.50632900
H	3.07841200	1.66161500	-3.59769200
H	3.72626700	2.11399900	0.62512300
H	4.62533000	1.93558400	-1.67538400
C	-4.66099500	2.34741400	-0.54737200
C	-5.47278700	1.77026500	-1.54096600
C	-5.17212400	3.19634000	0.44899200
C	-6.84189400	2.05886000	-1.51056700
C	-6.54731800	3.45183100	0.44281200
C	-7.37784500	2.88936700	-0.52624100
H	-7.48933400	1.62628600	-2.26856400
H	-6.96561000	4.10214200	1.20613000
H	-8.44205200	3.10480800	-0.51978200
C	1.14913200	2.03724000	1.45024400
H	0.95161900	1.06152600	1.91066700
H	1.87907400	2.55458600	2.07869500
C	0.35058400	1.53149300	-3.55430000
H	-0.32416600	0.67579300	-3.43511400
H	-0.27428600	2.42904800	-3.65046600

H	0.89555600	1.40417900	-4.49376200
C	-4.25568000	3.78402500	1.49170900
H	-3.40358300	4.28682600	1.02373900
H	-4.78416300	4.50627300	2.11959400
H	-3.84544500	3.00486600	2.14720400
C	-4.88474800	0.85022500	-2.58406200
H	-4.59707700	-0.11514600	-2.14801000
H	-5.60400900	0.65174100	-3.38319600
H	-3.98104900	1.27622500	-3.03489000
C	-2.73928100	1.02327500	-0.10012200
C	-1.25178000	0.81527600	-0.24715100
C	-3.87938700	6.01202200	-1.51756100
H	-4.30918700	6.32944600	-0.55702600
H	-3.86365000	4.91294900	-1.46778500
C	-2.43240700	6.55501600	-1.60124000
C	-4.82934200	6.43848000	-2.64377000
H	-4.93336300	7.53329500	-2.63409600
H	-2.49047800	7.65258000	-1.64671500
Si	-1.56581000	5.94088000	-3.14279700
O	0.05288400	6.06990000	-2.92469000
O	-2.07924700	6.52393600	-4.59361900
O	-1.92830100	4.27132600	-3.27947700
C	1.05362000	5.47955300	-3.75720400
H	1.28497400	4.46857200	-3.40092600
H	0.73836900	5.43230400	-4.80671600
H	1.95484800	6.09372600	-3.68735600
C	-2.18728400	7.91190500	-4.92288900
H	-1.24042100	8.43799300	-4.75276900
H	-2.44417000	7.98248600	-5.98233600
H	-2.97976900	8.38898800	-4.33388400
C	-2.85679800	3.71299800	-4.23675300
H	-2.84430800	4.30075300	-5.15532000
H	-2.53976100	2.68798400	-4.44450500
H	-3.86475900	3.71003400	-3.81003000
C	-4.66585400	-1.76255400	1.71000900
C	-3.58008100	-2.59155700	1.95110000
C	-2.27373000	-2.21971900	1.52615100
C	-2.16197300	-0.98068200	0.86860500
C	-3.27689700	-0.13418700	0.61255000
C	-4.53701400	-0.52190600	1.03402400
H	-1.09526400	-3.92302100	2.18822700
H	-5.65127600	-2.07124500	2.04467100
H	-3.72503500	-3.53625900	2.46792000
C	-1.06894400	-2.95967700	1.68650300

C	-0.92681500	-0.46311100	0.38617000
H	-5.40943100	0.09653400	0.85425200
C	0.22780700	-1.20806200	0.55059400
C	0.13362500	-2.46256500	1.20657000
H	1.18738500	-0.84921400	0.19427200
H	1.03819900	-3.04804200	1.33816000
H	-4.40332500	6.18632900	-3.62404200
C	-1.02838400	4.75258200	-0.26225300
H	-0.00013300	4.72443500	-0.64874200
H	-1.02484800	4.36608800	0.76192900
C	-1.61478100	6.17275700	-0.32907300
H	-2.27101200	6.33801500	0.53556100
H	-0.78863300	6.88950700	-0.22750900
H	0.21561700	2.60704000	1.48041200
C	-6.21142500	5.78613300	-2.51913100
H	-6.64472100	6.03571900	-1.54112100
H	-6.09399600	4.69328400	-2.52016200
C	-7.17270400	6.20271300	-3.63558300
H	-6.77258200	5.93507100	-4.62171800
H	-8.14694400	5.71350500	-3.52658600
H	-7.34073600	7.28632400	-3.63029700

UB3LYP-D3/BSII(SMD)//B3LYP-D3/BSI

HF=-2272.6270402

^{1}**R**

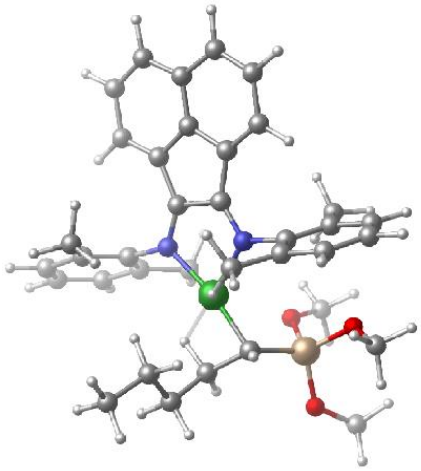

Ni	-1.52023200	3.44826000	-1.36032800
N	-0.32165900	1.93387400	-1.38575700
N	-2.86215200	2.17569700	-0.65640800
C	1.05593200	1.87123600	-1.78589500
C	1.34214100	1.79321600	-3.15902200
C	2.05391100	1.87607100	-0.79710100
C	2.68574900	1.69830000	-3.53877500
C	3.38208400	1.77381400	-1.22381700
C	3.69848000	1.68107300	-2.57946500
H	2.93294100	1.62843700	-4.59460100
H	4.17408400	1.77782600	-0.47998900
H	4.73658300	1.60473600	-2.88935800
C	-4.21774800	2.47419000	-0.31754900
C	-5.25869600	2.06569500	-1.17236500
C	-4.43913500	3.25372300	0.83363000
C	-6.56213300	2.43983500	-0.82528300
C	-5.75953200	3.59386200	1.14227600
C	-6.81414200	3.19101000	0.32246500
H	-7.38432100	2.14310100	-1.47085100
H	-5.95835600	4.18214500	2.03391500
H	-7.83298200	3.47047100	0.57347300
C	1.69729900	1.97539400	0.66481800
H	2.58132400	2.21133900	1.26260400
H	0.95212800	2.75845700	0.83118200
H	1.28182700	1.03058200	1.03946600
C	0.22593200	1.75346600	-4.17229100
H	-0.52633700	2.52227800	-3.96594500
H	0.60601500	1.90010500	-5.18702300
H	-0.29686500	0.78804500	-4.14812400
C	-3.28465000	3.66993000	1.71323700
H	-2.79076800	2.79756000	2.16033200

H	-2.51009500	4.20982700	1.15305100
H	-3.62764900	4.31446000	2.52731700
C	-4.98194000	1.27291000	-2.42822400
H	-4.14668600	1.69833200	-2.99711800
H	-4.71989600	0.23177200	-2.20211200
H	-5.86045500	1.25773400	-3.07864800
C	-2.35449000	0.99536700	-0.55821800
C	-0.91639300	0.86191700	-0.97338800
C	-1.89895100	5.51270400	-2.00512400
H	-2.00951800	6.34690600	-1.30323900
H	-2.61198300	4.74449100	-1.45257700
C	-0.51611600	4.93769000	-2.01061700
H	-0.07130700	4.76592700	-2.99324200
C	-2.60215400	5.80136800	-3.33852000
H	-2.06262400	6.62069400	-3.82997200
H	-3.61252400	6.17440300	-3.12675700
C	-2.69467100	4.59479300	-4.27862400
H	-1.68752600	4.28546500	-4.58288500
C	-3.53941200	4.87832700	-5.52394300
H	-3.12062300	5.71080900	-6.10119400
H	-3.58296400	4.00382200	-6.18180100
H	-4.56678400	5.14626100	-5.25067200
H	-3.12322300	3.74487800	-3.72385400
Si	0.59542900	5.55662400	-0.66043400
O	0.62533500	7.20169700	-0.78424000
O	2.12669300	4.94603200	-0.64508600
O	-0.13747500	5.03020200	0.73376000
C	0.33823200	5.30025900	2.05229700
H	0.30114700	6.37402100	2.27318900
H	-0.31418000	4.77684500	2.75703700
H	1.36573300	4.94063000	2.18189600
C	3.04454100	5.13588000	-1.72292500
H	2.76700600	4.50972300	-2.57901700
H	3.08738200	6.18663400	-2.03671600
H	4.03504100	4.82867100	-1.37937300
C	1.49862900	8.07887200	-0.07288200
H	1.68227600	8.95858600	-0.69618900
H	1.03046900	8.40416700	0.86406200
H	2.45901800	7.60041700	0.15667400
C	-2.81156700	-0.31846000	-0.11526500
C	-3.99761200	-0.82610100	0.38478300
C	-1.69210900	-1.18367700	-0.27890600
C	-4.05008100	-2.20391000	0.71807500
H	-4.87004500	-0.19537300	0.51764100

C	-1.72943800	-2.55352700	0.04043600
C	-0.53133700	-0.53554700	-0.79388300
C	-2.96140000	-3.04789500	0.55362700
H	-4.97743100	-2.60782400	1.11192900
C	-0.53067300	-3.28593300	-0.18673400
C	0.62103000	-1.27362800	-1.00396100
H	-3.04678500	-4.09836100	0.81767000
C	0.59747800	-2.65747000	-0.69337700
H	-0.50213300	-4.34801300	0.04067500
H	1.52438100	-0.81559500	-1.39049700
H	1.49836000	-3.24045000	-0.85716000
Ni	-2.37603700	3.63105900	-0.92606100
N	-0.85932600	2.42641900	-0.27610000
N	-3.48282000	1.92965000	-0.69049400
C	0.50612500	2.81321900	-0.16377200
C	1.44736500	2.40395900	-1.13116900
C	0.83364400	3.70712100	0.87458300
C	2.75956200	2.87163000	-0.99224900
C	2.15879600	4.13571800	0.97902200
C	3.11874500	3.71687800	0.05685000
H	3.50235000	2.57679700	-1.72847700
H	2.43664200	4.80811100	1.78594900
H	4.14353800	4.06471100	0.14543700
C	-4.89218300	1.81109700	-0.88564100
C	-5.39453700	1.29319500	-2.09223600
C	-5.72532400	2.30062700	0.13603100
C	-6.78474100	1.26381400	-2.25014800
C	-7.10852800	2.25230100	-0.06703800
C	-7.63656400	1.73782900	-1.25160600
H	-7.19932600	0.87067900	-3.17437000
H	-7.77184600	2.61579100	0.71324200
H	-8.71228300	1.70655400	-1.39712200
C	-0.22637400	4.16839600	1.84473800
H	-1.05017600	4.67805300	1.32147800
H	-0.67373500	3.32990100	2.39162500
H	0.18446500	4.87072200	2.57477200
C	1.05578800	1.54034400	-2.30686100
H	0.08551000	1.84195600	-2.71619300
H	1.79841500	1.62027000	-3.10518900
H	0.97933900	0.48159300	-2.03152200
C	-5.12702400	2.82706400	1.41772700
H	-5.88932300	3.28842500	2.05140700
H	-4.64973800	2.02519900	1.99505100
H	-4.34875700	3.57550800	1.21854900

C	-4.46573500	0.80633400	-3.17578400
H	-3.87360200	-0.05574500	-2.84567900
H	-5.02848200	0.50466200	-4.06297300
H	-3.77426100	1.60083800	-3.47157200
C	-2.73365500	0.93499500	-0.35901900
C	-1.26400800	1.20575200	-0.14674600
C	-0.64831900	4.84381200	-2.97114600
H	0.08114800	4.40667900	-2.27538200
H	-0.79463100	5.87881100	-2.62959200
C	-1.96663000	4.06616400	-2.91780800
C	0.00821800	4.85901800	-4.36949000
H	0.23343000	3.82185100	-4.65941800
H	0.97602600	5.37422300	-4.29215200
C	-0.83297900	5.51817200	-5.46803200
H	-1.78283500	4.97919700	-5.57906700
C	-0.10863400	5.55767700	-6.81721700
H	0.14137100	4.54591900	-7.15960200
H	-0.72768600	6.02790100	-7.58887500
H	0.82772200	6.12450800	-6.74718400
H	-1.09696600	6.53963800	-5.16109800
H	-1.92463700	3.13589900	-3.49266500
Si	-3.56492400	4.95940600	-3.01694200
O	-4.73708600	4.01290900	-3.65258200
O	-3.73455600	6.47113500	-3.63655600
O	-3.76793800	5.12823300	-1.30671700
C	-6.08497600	4.38791800	-3.94428600
H	-6.73027100	4.13207900	-3.09631800
H	-6.16580900	5.45739500	-4.16596300
H	-6.41191800	3.81837200	-4.81842700
C	-3.15812800	7.67219100	-3.12888200
H	-3.83749900	8.49857700	-3.35619900
H	-3.00739500	7.62363400	-2.04258800
H	-2.19458400	7.86418300	-3.61317100
C	-4.95363600	5.65447500	-0.68571600
H	-5.74408900	4.89832000	-0.68545300
H	-4.70904200	5.93131300	0.34213300
H	-5.28906000	6.54349000	-1.22870800
C	-3.89351500	-2.67627000	0.24061600
C	-2.65621300	-3.19663000	0.59023700
C	-1.49972100	-2.36752500	0.61266200
C	-1.69213300	-1.01967100	0.25711100
C	-2.96206600	-0.48441800	-0.09578200
C	-4.07084700	-1.31227400	-0.10688700
H	0.03550800	-3.77416800	1.23897200

H	-4.75938400	-3.33085900	0.23225100
H	-2.56528700	-4.24737500	0.85137300
C	-0.17228500	-2.74528800	0.95846800
C	-0.63919600	-0.06148600	0.23281000
H	-5.05306800	-0.93852800	-0.37420600
C	0.64124300	-0.45729900	0.57995600
C	0.85381000	-1.81217400	0.94225100
H	1.47042600	0.24155500	0.57465500
H	1.85741200	-2.12482800	1.21308500

UB3LYP-D3/BSII(SMD)//B3LYP-D3/BSI

HF=-2193.9691734

^{3}R

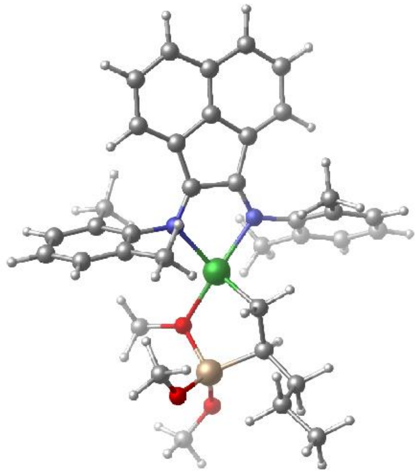

Zero-point correction= 0.718794 (Hartree/Particle)
Thermal correction to Energy= 0.764299
Thermal correction to Enthalpy= 0.765244
Thermal correction to Gibbs Free Energy= 0.637614
Sum of electronic and zero-point Energies= -2191.063000
Sum of electronic and thermal Energies= -2191.017495
Sum of electronic and thermal Enthalpies= -2191.016550
Sum of electronic and thermal Free Energies= -2191.144179

Ni	-2.37603700	3.63105900	-0.92606100
N	-0.85932600	2.42641900	-0.27610000
N	-3.48282000	1.92965000	-0.69049400
C	0.50612500	2.81321900	-0.16377200
C	1.44736500	2.40395900	-1.13116900
C	0.83364400	3.70712100	0.87458300
C	2.75956200	2.87163000	-0.99224900
C	2.15879600	4.13571800	0.97902200
C	3.11874500	3.71687800	0.05685000
H	3.50235000	2.57679700	-1.72847700
H	2.43664200	4.80811100	1.78594900
H	4.14353800	4.06471100	0.14543700
C	-4.89218300	1.81109700	-0.88564100
C	-5.39453700	1.29319500	-2.09223600
C	-5.72532400	2.30062700	0.13603100
C	-6.78474100	1.26381400	-2.25014800
C	-7.10852800	2.25230100	-0.06703800
C	-7.63656400	1.73782900	-1.25160600
H	-7.19932600	0.87067900	-3.17437000
H	-7.77184600	2.61579100	0.71324200
H	-8.71228300	1.70655400	-1.39712200
C	-0.22637400	4.16839600	1.84473800
H	-1.05017600	4.67805300	1.32147800

H	-0.67373500	3.32990100	2.39162500
H	0.18446500	4.87072200	2.57477200
C	1.05578800	1.54034400	-2.30686100
H	0.08551000	1.84195600	-2.71619300
H	1.79841500	1.62027000	-3.10518900
H	0.97933900	0.48159300	-2.03152200
C	-5.12702400	2.82706400	1.41772700
H	-5.88932300	3.28842500	2.05140700
H	-4.64973800	2.02519900	1.99505100
H	-4.34875700	3.57550800	1.21854900
C	-4.46573500	0.80633400	-3.17578400
H	-3.87360200	-0.05574500	-2.84567900
H	-5.02848200	0.50466200	-4.06297300
H	-3.77426100	1.60083800	-3.47157200
C	-2.73365500	0.93499500	-0.35901900
C	-1.26400800	1.20575200	-0.14674600
C	-0.64831900	4.84381200	-2.97114600
H	0.08114800	4.40667900	-2.27538200
H	-0.79463100	5.87881100	-2.62959200
C	-1.96663000	4.06616400	-2.91780800
C	0.00821800	4.85901800	-4.36949000
H	0.23343000	3.82185100	-4.65941800
H	0.97602600	5.37422300	-4.29215200
C	-0.83297900	5.51817200	-5.46803200
H	-1.78283500	4.97919700	-5.57906700
C	-0.10863400	5.55767700	-6.81721700
H	0.14137100	4.54591900	-7.15960200
H	-0.72768600	6.02790100	-7.58887500
H	0.82772200	6.12450800	-6.74718400
H	-1.09696600	6.53963800	-5.16109800
H	-1.92463700	3.13589900	-3.49266500
Si	-3.56492400	4.95940600	-3.01694200
O	-4.73708600	4.01290900	-3.65258200
O	-3.73455600	6.47113500	-3.63655600
O	-3.76793800	5.12823300	-1.30671700
C	-6.08497600	4.38791800	-3.94428600
H	-6.73027100	4.13207900	-3.09631800
H	-6.16580900	5.45739500	-4.16596300
H	-6.41191800	3.81837200	-4.81842700
C	-3.15812800	7.67219100	-3.12888200
H	-3.83749900	8.49857700	-3.35619900
H	-3.00739500	7.62363400	-2.04258800
H	-2.19458400	7.86418300	-3.61317100
C	-4.95363600	5.65447500	-0.68571600

H	-5.74408900	4.89832000	-0.68545300
H	-4.70904200	5.93131300	0.34213300
H	-5.28906000	6.54349000	-1.22870800
C	-3.89351500	-2.67627000	0.24061600
C	-2.65621300	-3.19663000	0.59023700
C	-1.49972100	-2.36752500	0.61266200
C	-1.69213300	-1.01967100	0.25711100
C	-2.96206600	-0.48441800	-0.09578200
C	-4.07084700	-1.31227400	-0.10688700
H	0.03550800	-3.77416800	1.23897200
H	-4.75938400	-3.33085900	0.23225100
H	-2.56528700	-4.24737500	0.85137300
C	-0.17228500	-2.74528800	0.95846800
C	-0.63919600	-0.06148600	0.23281000
H	-5.05306800	-0.93852800	-0.37420600
C	0.64124300	-0.45729900	0.57995600
C	0.85381000	-1.81217400	0.94225100
H	1.47042600	0.24155500	0.57465500
H	1.85741200	-2.12482800	1.21308500

UB3LYP-D3/BSII(SMD)//B3LYP-D3/BSI

HF=-2193.9745854

[1]TS8

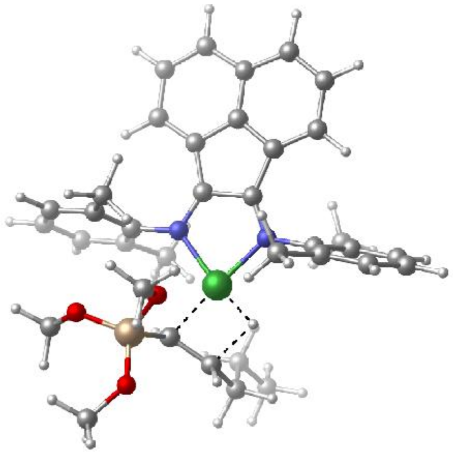

Ni	-1.63824500	3.51936400	-1.30871100
N	-0.35211600	1.98675400	-1.35381800
N	-2.89817500	2.21154000	-0.62970400
C	1.01592800	1.91476400	-1.78652300
C	1.26396300	1.85144000	-3.16937400
C	2.04683200	1.88652500	-0.83025100
C	2.59467100	1.76023800	-3.59316700
C	3.36097900	1.78938900	-1.30173700
C	3.63707700	1.72576300	-2.66736300
H	2.80742200	1.70229600	-4.65726000
H	4.17474000	1.76978100	-0.58196500
H	4.66505200	1.65305100	-3.01015300
C	-4.26313300	2.45139500	-0.26036200
C	-5.28830900	2.07309500	-1.14314500
C	-4.50542400	3.10838600	0.95729800
C	-6.60507800	2.34790700	-0.75686500
C	-5.83761500	3.35725800	1.30303200
C	-6.88022800	2.97811600	0.45711100
H	-7.41858000	2.06941700	-1.42133800
H	-6.05428000	3.85665500	2.24345500
H	-7.90858400	3.18305900	0.73941300
C	1.74921800	1.91954600	0.64814100
H	2.64152200	2.20020000	1.21372900
H	0.95817400	2.63726600	0.87668000
H	1.42344800	0.93356800	1.00528300
C	0.12100000	1.79527800	-4.15216700
H	-0.67459700	2.49725900	-3.88737400
H	0.45930600	2.01751800	-5.16822100
H	-0.33415900	0.79579200	-4.16536400
C	-3.35649300	3.51155000	1.84820100
H	-2.82227800	2.63366600	2.23460300

H	-2.61682300	4.10902700	1.30001600
H	-3.70944300	4.09325200	2.70424200
C	-4.97168400	1.40948900	-2.46199600
H	-4.20957100	1.97161800	-3.01626500
H	-4.58315900	0.39273100	-2.32167900
H	-5.86490800	1.33891800	-3.08829500
C	-2.36622500	1.04356100	-0.50438800
C	-0.93267800	0.91459000	-0.92776500
C	-1.69294400	5.64007300	-2.09465300
H	-1.96380000	6.36301100	-1.32475700
H	-2.76817900	4.56370900	-1.32280500
C	-0.43958900	4.99437800	-1.94090100
H	0.04525100	4.60678100	-2.83823200
C	-2.42340000	5.85416500	-3.40484600
H	-1.99545900	6.76727300	-3.84675200
H	-3.47261900	6.08712900	-3.18748000
C	-2.34858600	4.70630300	-4.41409100
H	-1.30515700	4.53201600	-4.70332500
C	-3.18932600	4.97193100	-5.66599600
H	-2.85066000	5.87632100	-6.18474000
H	-3.12142600	4.13706600	-6.37126600
H	-4.24615200	5.11075100	-5.40933800
H	-2.69401500	3.78640900	-3.91823400
Si	0.58135900	5.48925400	-0.45466100
O	0.45483700	7.10997600	-0.17944000
O	2.15218000	5.01663600	-0.57636000
O	-0.13786200	4.73887000	0.83115600
C	0.13726500	5.07130300	2.19631400
H	0.19018300	6.15667100	2.33181400
H	-0.67719200	4.67188600	2.80653200
H	1.08181400	4.61675500	2.51691300
C	2.95485300	5.12572100	-1.75113200
H	2.56674500	4.48501300	-2.55108200
H	3.01318400	6.16221100	-2.10761300
H	3.96162600	4.78589000	-1.49981300
C	1.44342300	8.10319700	-0.44051700
H	1.45132000	8.37750100	-1.50365000
H	1.19233700	8.99118500	0.14577700
H	2.44376100	7.75926500	-0.15222700
C	-2.81745500	-0.26553600	-0.04324400
C	-3.99956300	-0.77181400	0.46804100
C	-1.69910500	-1.13105200	-0.21557300
C	-4.04852000	-2.14899900	0.80442300
H	-4.87085300	-0.14148300	0.60759600

C	-1.73441200	-2.50058400	0.10594100
C	-0.54514600	-0.48449500	-0.74646000
C	-2.96153600	-2.99341400	0.63196300
H	-4.97226000	-2.55200100	1.20762200
C	-0.53945800	-3.23484300	-0.13376300
C	0.60281700	-1.22525500	-0.97025000
H	-3.04450200	-4.04330900	0.89913000
C	0.58236800	-2.60847000	-0.65625600
H	-0.50920200	-4.29643400	0.09571300
H	1.50159600	-0.77040000	-1.37080200
H	1.48066500	-3.19240800	-0.83063100

UB3LYP-D3/BSII(SMD)//B3LYP-D3/BSI

HF=-2193.9505661

^{3}TS8

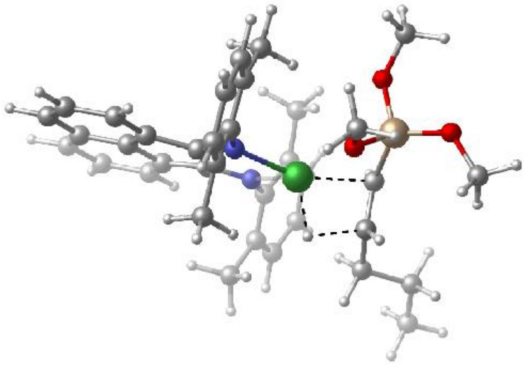

Zero-point correction= 0.715387 (Hartree/Particle)
Thermal correction to Energy= 0.761274
Thermal correction to Enthalpy= 0.762218
Thermal correction to Gibbs Free Energy= 0.631328
Sum of electronic and zero-point Energies= -2191.032363
Sum of electronic and thermal Energies= -2190.986477
Sum of electronic and thermal Enthalpies= -2190.985533
Sum of electronic and thermal Free Energies= -2191.116423

Ni	-2.60740800	3.44319000	-1.58428600
N	-1.03195300	2.08806900	-1.27138000
N	-3.62585700	1.99837300	-0.52396000
C	0.29664100	2.22457800	-1.77085300
C	0.52022300	1.89017100	-3.11892900
C	1.29752500	2.76305300	-0.94291500
C	1.80198600	2.10674600	-3.63598600
C	2.56371100	2.96257700	-1.50559300
C	2.81590800	2.64060700	-2.83967500
H	2.00275100	1.85154300	-4.67294000
H	3.35327600	3.38447000	-0.88964500
H	3.80357800	2.80680800	-3.25929200
C	-5.02935300	1.99765200	-0.26022500
C	-5.87792800	1.46267800	-1.24428400
C	-5.50625900	2.60067600	0.91488600
C	-7.25524500	1.50893300	-1.00515300
C	-6.89259300	2.63385700	1.10659900
C	-7.76065900	2.08756400	0.15989000
H	-7.93472600	1.09557100	-1.74579700
H	-7.29007900	3.09400500	2.00719400
H	-8.83313800	2.12011300	0.32709400
C	0.99479200	3.14191200	0.48644300
H	0.10048300	3.77448300	0.54261300
H	0.80523200	2.25704700	1.10715100
H	1.83096400	3.68722400	0.93202100
C	-0.58478600	1.28445200	-3.95081200

H	-1.49232200	1.90051700	-3.94347700
H	-0.27041300	1.16158500	-4.99080800
H	-0.86968500	0.29567500	-3.56864900
C	-4.54186500	3.18796000	1.91631800
H	-5.07219700	3.73312300	2.70158500
H	-3.94104900	2.40621700	2.39886300
H	-3.83811300	3.87572900	1.43031400
C	-5.30222900	0.89537700	-2.51933400
H	-4.65104300	0.03441300	-2.32410600
H	-6.09588400	0.56494300	-3.19531400
H	-4.69220000	1.64255100	-3.04495300
C	-2.85147100	1.05818400	-0.10831200
C	-1.39834700	1.11092300	-0.52140800
C	-1.56089100	5.53385700	-2.21123100
H	-1.95550800	4.71735300	-0.74837300
H	-2.32563800	6.25262800	-1.91703400
C	-1.82416100	4.72793600	-3.34165300
C	-0.14506100	5.90668800	-1.83192200
H	0.49557500	5.02397700	-1.91451400
H	-0.10743500	6.24201200	-0.78926900
C	0.40304900	7.01590300	-2.75230000
H	0.34573200	6.67593300	-3.79415800
C	1.84963700	7.37482500	-2.39831900
H	2.50112400	6.49709400	-2.48895800
H	2.23770200	8.15354500	-3.06311900
H	1.92557700	7.74399900	-1.36860900
H	-0.23472100	7.90744600	-2.67718000
H	-0.97127400	4.22223300	-3.79022700
Si	-3.50919000	4.61848200	-4.06489400
O	-3.63259400	3.27471400	-4.99172200
O	-4.15183300	5.87878300	-4.89669800
O	-4.34350200	4.43066200	-2.59678200
C	-4.57676600	3.03342400	-6.03958100
H	-5.50385500	2.62006100	-5.62405900
H	-4.80467600	3.95196600	-6.58939500
H	-4.13791200	2.30174000	-6.72251800
C	-4.04112800	7.24980500	-4.51099800
H	-4.45276900	7.85803300	-5.31958100
H	-4.60859200	7.44981700	-3.59274200
H	-2.99281400	7.53220300	-4.35235600
C	-5.76083500	4.57169200	-2.40676400
H	-6.29661100	3.75371500	-2.89722500
H	-5.96152500	4.53194100	-1.33531600
H	-6.09789200	5.52882900	-2.81666500

C	-3.89548300	-1.96805500	2.01055300
C	-2.65056600	-2.57865800	2.06059100
C	-1.52694400	-1.99306200	1.41263400
C	-1.76038600	-0.78069200	0.73770900
C	-3.03769900	-0.15440300	0.68507000
C	-4.11394500	-0.74701800	1.32224900
H	0.04381800	-3.42474000	1.87193800
H	-4.73489500	-2.43776200	2.51362100
H	-2.52896600	-3.51464700	2.59887200
C	-0.19517100	-2.49294300	1.36680800
C	-0.74370300	-0.07198100	0.03840400
H	-5.10073900	-0.29765500	1.30196500
C	0.54209400	-0.58132300	0.00853600
C	0.79610500	-1.80284200	0.68490900
H	1.34178100	-0.06702800	-0.51319700
H	1.80348900	-2.20674900	0.66552400

UB3LYP-D3/BSII(SMD)//B3LYP-D3/BSI

HF=-2193.9390128

[1]S

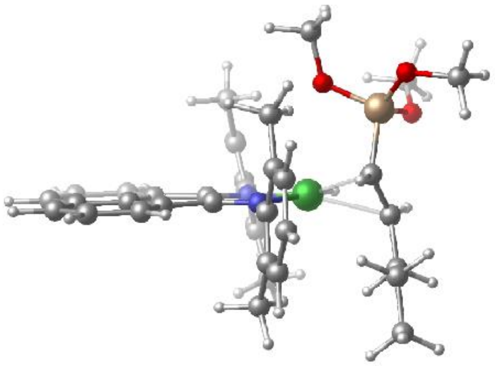

Ni	-1.73997800	3.50841900	-1.38395400
N	-0.59178200	1.81579800	-1.64570800
N	-2.92268600	2.31463100	-0.44450200
C	0.59862600	1.57317800	-2.40729100
C	0.45686200	1.11398600	-3.73008000
C	1.85075600	1.82503500	-1.82275800
C	1.62107400	0.91382300	-4.47849100
C	2.98849100	1.61514700	-2.61222600
C	2.87833900	1.16486400	-3.92714100
H	1.53589900	0.56679900	-5.50462000
H	3.96845900	1.80470300	-2.18273100
H	3.77217100	1.01083400	-4.52419700
C	-4.14607400	2.69086000	0.20284700
C	-5.28584200	2.85758500	-0.59999500
C	-4.14442600	2.92452900	1.58864900
C	-6.47592700	3.22913000	0.03301200
C	-5.35710800	3.30134400	2.17666800
C	-6.51490300	3.44402100	1.41082700
H	-7.37522500	3.35497700	-0.56365900
H	-5.38658900	3.49264700	3.24600900
H	-7.44605800	3.73605700	1.88721200
C	1.95827000	2.24859300	-0.37873300
H	2.94460600	2.66939300	-0.16445500
H	1.19927800	2.98819900	-0.11053200
H	1.81219300	1.38922400	0.28988800
C	-0.91196800	0.85498900	-4.31180400
H	-1.38245000	-0.02571800	-3.85605400
H	-1.58548900	1.70259400	-4.13674600
H	-0.85357500	0.68153200	-5.38933200
C	-2.87426800	2.80279500	2.39623800
H	-2.55956200	1.75640200	2.50209200
H	-2.05321700	3.34690700	1.91578600
H	-3.01587000	3.20568300	3.40299300
C	-5.20238300	2.64226000	-2.08954900

H	-4.41895300	3.27850600	-2.52463800
H	-4.94223900	1.60517400	-2.33661300
H	-6.15136400	2.87827700	-2.57827600
C	-2.45737400	1.11511400	-0.33999800
C	-1.15927800	0.83089500	-1.03879700
C	-1.23626800	5.26157000	-2.80013900
H	-1.95098200	6.00779400	-2.46096500
H	-2.70760400	4.56259600	-1.11691200
C	-0.24808500	4.87847300	-1.91724100
H	0.57187300	4.27334300	-2.30507000
C	-1.34877800	4.88145000	-4.25643200
H	-1.72854600	5.75934300	-4.79739000
H	-2.14528600	4.12686700	-4.35295100
C	-0.06282300	4.37669600	-4.92116200
H	0.70247000	5.16129600	-4.85483000
C	-0.28345100	3.98131800	-6.38355100
H	-0.64224100	4.82875900	-6.97921100
H	0.64752100	3.62390000	-6.83567200
H	-1.02642200	3.17840400	-6.46609900
H	0.32910100	3.51665500	-4.37269600
Si	0.00114500	5.72683900	-0.26617500
O	-1.12950100	6.91846600	-0.20039200
O	1.54460200	6.28344200	-0.12910400
O	-0.18179000	4.61767200	0.94545800
C	0.52426800	4.63402300	2.19056700
H	0.40394100	3.65006400	2.65214900
H	1.58792400	4.83905200	2.03666300
H	0.10482900	5.39244400	2.86238100
C	2.08167900	7.36411000	-0.89532600
H	3.08756800	7.57034900	-0.52172700
H	2.14771200	7.09709400	-1.95821300
H	1.46864000	8.26684200	-0.79105900
C	-1.76599500	7.36172500	0.99917600
H	-2.16853500	6.51252800	1.56489800
H	-1.06686800	7.92022800	1.63385500
H	-2.58872800	8.02321900	0.71711000
C	-2.91313700	-0.12637900	0.27815800
C	-4.00887400	-0.50242100	1.03587000
C	-1.93022600	-1.10058800	-0.06263400
C	-4.10749900	-1.85654400	1.44640200
H	-4.77374500	0.21256900	1.31751800
C	-2.01276600	-2.44684400	0.33825200
C	-0.86049400	-0.58854500	-0.85121800
C	-3.14925700	-2.80395000	1.11669500

H	-4.96450000	-2.15655200	2.04122400
C	-0.95089100	-3.29539600	-0.08316600
C	0.15858300	-1.43693500	-1.24800200
H	-3.26442700	-3.83016000	1.45457400
C	0.09311100	-2.79659300	-0.84833600
H	-0.96187000	-4.34475900	0.19839500
H	0.98766600	-1.08297800	-1.85038000
H	0.88963100	-3.46662400	-1.15641000

UB3LYP-D3/BSII(SMD)//B3LYP-D3/BSI

HF=-2193.9475446

³S

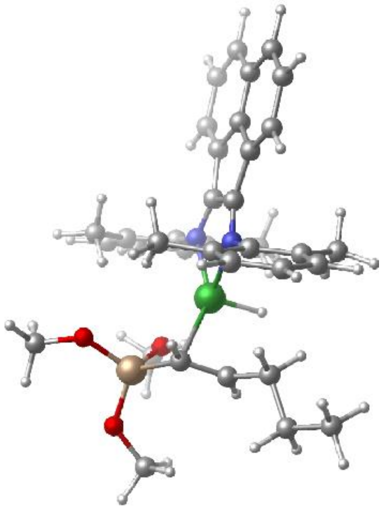

Zero-point correction= 0.713716 (Hartree/Particle)
Thermal correction to Energy= 0.760073
Thermal correction to Enthalpy= 0.761017
Thermal correction to Gibbs Free Energy= 0.630074
Sum of electronic and zero-point Energies= -2191.047376
Sum of electronic and thermal Energies= -2191.001019
Sum of electronic and thermal Enthalpies= -2191.000075
Sum of electronic and thermal Free Energies= -2191.131019

Ni	-2.61367300	3.47787200	-1.55188900
N	-1.06262700	2.11680300	-1.28332200
N	-3.67194600	1.95248700	-0.62030900
C	0.28581100	2.29380700	-1.71348700
C	0.59253600	1.94854500	-3.04185500
C	1.22209400	2.87798800	-0.84236300
C	1.88779400	2.21314300	-3.49973500
C	2.50596600	3.12009300	-1.34368500
C	2.83756600	2.79719000	-2.66009100
H	2.15112500	1.95336500	-4.52154500
H	3.24652900	3.57983200	-0.69491900
H	3.83741600	3.00035900	-3.03203900
C	-5.07560100	1.93519400	-0.36192200
C	-5.91652400	1.43377600	-1.37052700
C	-5.56453300	2.47974200	0.83816300
C	-7.29620500	1.46715600	-1.14245000
C	-6.95296100	2.50166500	1.01730800
C	-7.81278000	1.99858300	0.04018400
H	-7.96777200	1.07597300	-1.90216800
H	-7.35883300	2.91939100	1.93469300
H	-8.88670600	2.02289400	0.19959400
C	0.83677100	3.25085600	0.56842600

H	-0.10954200	3.80480600	0.58508500
H	0.69860500	2.35947600	1.19391100
H	1.61069600	3.86600000	1.03548900
C	-0.43688500	1.26760400	-3.91218600
H	-1.41499600	1.76031900	-3.85817800
H	-0.12040400	1.24955300	-4.95885200
H	-0.59284500	0.22849200	-3.59403400
C	-4.61618700	3.01598200	1.88262300
H	-5.15878000	3.54609400	2.66996000
H	-4.04899200	2.20448300	2.35723700
H	-3.88408500	3.69987400	1.43692300
C	-5.32894300	0.88933100	-2.64957700
H	-4.69955300	0.01032800	-2.46240600
H	-6.11628300	0.59050400	-3.34749200
H	-4.69443900	1.63416700	-3.14548400
C	-2.88499700	1.04140400	-0.16902400
C	-1.42525100	1.13439100	-0.53681500
C	-1.35735600	5.47117300	-2.69817800
H	-2.34598000	4.39411800	-0.29306900
H	-2.05429200	6.12214400	-2.16757300
C	-1.83112600	4.62029600	-3.64789200
C	0.08483400	5.66595100	-2.34805800
H	0.69923200	4.89926900	-2.83066700
H	0.19949700	5.54142300	-1.26352500
C	0.59295700	7.06887200	-2.74183600
H	0.47649800	7.20231100	-3.82533800
C	2.05957400	7.26397000	-2.34481400
H	2.69739100	6.50949500	-2.82041900
H	2.42271500	8.25219600	-2.64515400
H	2.18771300	7.17310600	-1.25946700
H	-0.03032000	7.83524400	-2.26134700
H	-1.10414600	4.01745800	-4.18905000
Si	-3.62703500	4.56884000	-4.10066000
O	-3.91025800	3.24490700	-5.01278700
O	-4.29168000	5.87542300	-4.82854400
O	-4.25626400	4.40570200	-2.53899600
C	-5.05077800	3.00640500	-5.84639100
H	-5.89178200	2.64509500	-5.24267800
H	-5.34770900	3.91549600	-6.37860700
H	-4.77621000	2.23559600	-6.57006500
C	-4.05741000	7.24077100	-4.47451000
H	-4.52502200	7.86605200	-5.23799600
H	-4.50108800	7.47773400	-3.49929500
H	-2.98336500	7.46041000	-4.44160000

C	-5.58657000	4.74064300	-2.09665100
H	-6.28982200	3.95886100	-2.39249500
H	-5.56032900	4.80591400	-1.00883900
H	-5.88891100	5.69968900	-2.52601000
C	-3.89257200	-1.97764500	1.98224500
C	-2.63095100	-2.54564300	2.08104300
C	-1.50863400	-1.93478300	1.45450700
C	-1.76165800	-0.74400900	0.74861800
C	-3.05590900	-0.16139200	0.64615800
C	-4.12992500	-0.77711800	1.26385800
H	0.09443100	-3.30221300	1.99081400
H	-4.73109500	-2.46468300	2.47015100
H	-2.49451000	-3.46616300	2.64194600
C	-0.16005700	-2.38910900	1.45973600
C	-0.74808700	-0.01371300	0.06695300
H	-5.12971600	-0.36079300	1.20799100
C	0.55470500	-0.47906700	0.08748400
C	0.82842300	-1.67801400	0.79542300
H	1.35316500	0.05249500	-0.41844300
H	1.84913800	-2.04695400	0.81569700

UB3LYP-D3/BSII(SMD)//B3LYP-D3/BSI

HF=-2193.954489

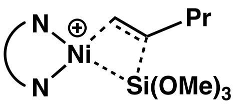

Singlet

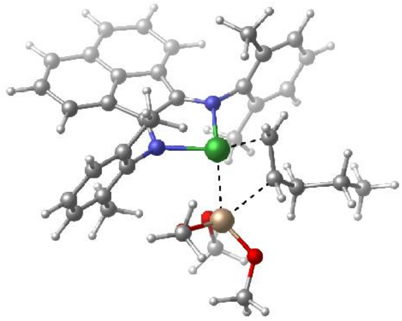

Zero-point correction= 0.718642 (Hartree/Particle)
Thermal correction to Energy= 0.763563
Thermal correction to Enthalpy= 0.764507
Thermal correction to Gibbs Free Energy= 0.639414
Sum of electronic and zero-point Energies= -2191.026725
Sum of electronic and thermal Energies= -2190.981805
Sum of electronic and thermal Enthalpies= -2190.980861
Sum of electronic and thermal Free Energies= -2191.105953

Ni	-2.58691800	3.33231000	-1.66972600
N	-1.09147600	2.14584600	-1.10668600
N	-3.74357600	1.84310500	-0.83404200
C	0.29811000	2.48606300	-1.12626100
C	1.07512000	2.07801200	-2.22301700
C	0.81231400	3.26454500	-0.07282400
C	2.42050900	2.46194600	-2.24457500
C	2.16085200	3.63124500	-0.14214600
C	2.96015300	3.23323000	-1.21501200
H	3.04497300	2.15349400	-3.07853900
H	2.58501700	4.23500200	0.65563400
H	4.00458700	3.52830500	-1.24999900
C	-5.14832700	1.55746600	-0.95169900
C	-5.60713000	1.11063300	-2.20100000
C	-6.00006900	1.73247300	0.15031300
C	-6.96726400	0.80698800	-2.32964700
C	-7.35358200	1.42613100	-0.02546000
C	-7.83626100	0.96010100	-1.25011800
H	-7.34243700	0.45341000	-3.28631400
H	-8.03477100	1.55760600	0.81099800
H	-8.89024100	0.72408100	-1.36343500
C	-0.06129000	3.66903100	1.09234100
H	-0.27542900	2.81013000	1.74193100
H	0.43420400	4.42839100	1.70377300

H	-1.02975400	4.06463700	0.76364900
C	0.46448200	1.23177300	-3.31219700
H	0.21131400	0.23001800	-2.94184700
H	-0.46750300	1.67519400	-3.68356200
H	1.15153400	1.11388000	-4.15428700
C	-5.46453400	2.22627900	1.47014600
H	-4.81844200	3.09726900	1.32651200
H	-6.28116500	2.50090000	2.14366300
H	-4.86748100	1.45319500	1.97162500
C	-4.65217800	0.97074700	-3.36174400
H	-4.11141400	1.91049500	-3.54436100
H	-3.88941900	0.20496500	-3.17267900
H	-5.18110200	0.69895100	-4.27924600
C	-2.98313000	0.92331600	-0.34075000
C	-1.50409000	1.15733000	-0.38865500
C	-2.24918500	5.35646400	-2.44137700
H	-2.97113500	5.88623900	-3.06293900
C	-1.61222600	4.22961200	-3.04977900
H	-2.01922800	3.82926500	-3.98215500
C	-1.34746300	6.26103900	-1.59903700
H	-0.67560300	5.66333500	-0.97815800
H	-1.89984700	6.95648500	-0.96764700
H	-0.53662000	4.11397700	-2.93140000
Si	-4.02752600	5.14737000	-1.00092200
O	-4.27644000	6.75936200	-1.29268300
O	-5.49252000	4.40923800	-1.15639600
O	-3.44420300	4.90845300	0.52698100
C	-5.43503700	7.47806000	-0.85551800
H	-5.70903300	8.19041500	-1.63852300
H	-6.28287000	6.81099300	-0.66384800
H	-5.20366400	8.03163700	0.06153000
C	-6.27059800	4.36763700	-2.35223300
H	-5.70830100	3.90759600	-3.17475200
H	-7.14677600	3.75070300	-2.14678500
H	-6.58834200	5.37299000	-2.65396500
C	-3.47924900	5.86782000	1.59027700
H	-2.71660700	5.58210800	2.31906000
H	-3.26381400	6.87708800	1.22325800
H	-4.46100200	5.86012600	2.07703300
C	-4.08241800	-2.38925700	1.28335800
C	-2.82371500	-2.80539900	1.68972300
C	-1.67534300	-2.01241400	1.41300700
C	-1.90172500	-0.80722300	0.72239600
C	-3.19516800	-0.37390900	0.30990000

C	-4.29305300	-1.16972700	0.58933300
H	-0.09209300	-3.21769300	2.28784500
H	-4.94228500	-3.01464100	1.50228100
H	-2.70905400	-3.74576400	2.22182100
C	-0.32359300	-2.30211800	1.75051800
C	-0.85275600	0.08400800	0.35726400
H	-5.29380200	-0.88019500	0.29097600
C	0.45395100	-0.22557900	0.69156600
C	0.69697300	-1.43104700	1.39871900
H	1.27647600	0.42985100	0.42750100
H	1.71910500	-1.67743600	1.66867500
H	-0.73617300	6.85673300	-2.28807800

UB3LYP-D3/BSII(SMD)//B3LYP-D3/BSI

HF=-2193.9296826

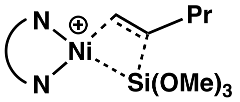

Triplet

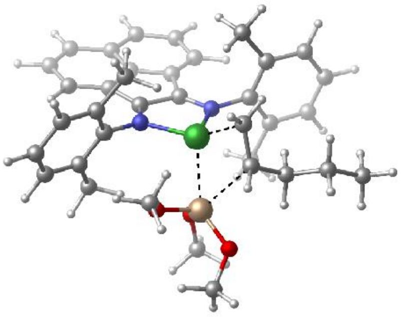

Zero-point correction=		0.716509 (Hartree/Particle)	
Thermal correction to Energy=		0.762307	
Thermal correction to Enthalpy=		0.763251	
Thermal correction to Gibbs Free Energy=		0.634770	
Sum of electronic and zero-point Energies=		-2191.017098	
Sum of electronic and thermal Energies=		-2190.971300	
Sum of electronic and thermal Enthalpies=		-2190.970356	
Sum of electronic and thermal Free Energies=		-2191.098837	
Ni	-2.86239700	3.25282400	-1.72829600
N	-1.23499200	2.39228100	-0.78954900
N	-3.81116000	1.64752200	-1.00479700
C	0.14657700	2.75012500	-0.89667100
C	0.86680900	2.27618700	-2.00938500
C	0.71745700	3.59704600	0.06874500
C	2.19112000	2.70349200	-2.15673500
C	2.04717900	3.99184200	-0.11623200
C	2.77765100	3.55796400	-1.22295900
H	2.76559400	2.35626100	-3.01129500
H	2.50817700	4.64949700	0.61569500
H	3.80563100	3.88191700	-1.35516500
C	-5.20209900	1.37755200	-1.18520100
C	-5.62472500	1.01961200	-2.47984200
C	-6.10509200	1.56471700	-0.12089700
C	-6.99119200	0.81488900	-2.69221900
C	-7.46445400	1.36102100	-0.38618400
C	-7.90765000	0.98555200	-1.65449000
H	-7.33553000	0.52164300	-3.68020700
H	-8.18231600	1.50704700	0.41639300
H	-8.96732600	0.83083500	-1.83496600
C	-0.08645900	4.04374200	1.26362900
H	-0.24492800	3.21323100	1.96452100
H	0.42655100	4.84354800	1.80509500

H	-1.07830300	4.39519100	0.96584100
C	0.24153600	1.30074700	-2.97871800
H	0.16449100	0.30121600	-2.53052700
H	-0.77437900	1.59941400	-3.26067600
H	0.84142700	1.20998900	-3.88835000
C	-5.63780200	1.99036900	1.24908200
H	-4.90497500	2.79953100	1.18623800
H	-6.48241100	2.33823100	1.85036600
H	-5.16916900	1.15902700	1.79062700
C	-4.62271900	0.84412300	-3.59528400
H	-4.04165500	1.76142000	-3.76316700
H	-3.89567600	0.05564900	-3.36576900
H	-5.11975700	0.58366100	-4.53361600
C	-3.03247700	0.88871200	-0.30405400
C	-1.58685000	1.28734800	-0.22336500
C	-2.38237700	5.26140200	-2.50795700
H	-3.17756800	5.93909700	-2.82584400
C	-2.14595000	4.17547700	-3.41721600
H	-2.83123200	4.00547700	-4.24644100
C	-1.17408800	5.95582700	-1.88415600
H	-0.49678800	5.19688700	-1.48504000
H	-1.47671500	6.59471400	-1.04825500
C	-0.41775400	6.81294200	-2.91483900
H	-0.14141800	6.18561900	-3.77199200
C	0.83853700	7.44299600	-2.30510400
H	1.52382100	6.66835800	-1.93957300
H	1.37494900	8.04906500	-3.04267100
H	0.58442100	8.09298000	-1.45898700
H	-1.08556200	7.59503100	-3.30015300
H	-1.13022700	3.81325200	-3.55513200
Si	-3.84954400	5.18032500	-0.62655400
O	-3.94395100	6.82629100	-0.72751100
O	-5.32125300	4.49805000	-0.97230000
O	-3.42094100	4.72094100	0.89891500
C	-5.06225200	7.59380600	-0.26212800
H	-4.92269500	7.87026400	0.78899300
H	-5.11694000	8.50427900	-0.86397900
H	-6.00213900	7.03851300	-0.36227500
C	-6.00684200	4.54085000	-2.22598700
H	-5.35255000	4.22599200	-3.05059800
H	-6.84207000	3.84080300	-2.16401100
H	-6.38108000	5.54984400	-2.43461400
C	-3.40187700	5.57454000	2.05137800
H	-2.72933900	5.12388600	2.78528900

H	-3.03864300	6.57634800	1.79926100
H	-4.40681000	5.64745900	2.48192000
C	-3.95347400	-2.45564700	1.35009600
C	-2.67944100	-2.78644600	1.78745100
C	-1.57950600	-1.91920800	1.53609500
C	-1.86849800	-0.73313300	0.83724600
C	-3.17705100	-0.38827800	0.39238600
C	-4.22641900	-1.25463000	0.64580600
H	0.06188100	-3.01606500	2.44760800
H	-4.77501900	-3.13580700	1.55226100
H	-2.51573900	-3.71649300	2.32480700
C	-0.21895800	-2.11821300	1.90395500
C	-0.87670100	0.22717400	0.49231900
H	-5.23651500	-1.02932100	0.32202200
C	0.43919100	0.00694500	0.85855400
C	0.74758300	-1.18075800	1.57179600
H	1.22213900	0.71495900	0.61016900
H	1.77830000	-1.35830200	1.86255500

UB3LYP-D3/BSII(SMD)//B3LYP-D3/BSI

HF=-2193.923159

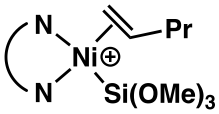

Singlet

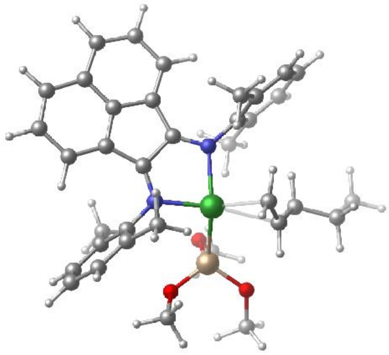

Zero-point correction= 0.718913 (Hartree/Particle)
Thermal correction to Energy= 0.765083
Thermal correction to Enthalpy= 0.766027
Thermal correction to Gibbs Free Energy= 0.636742
Sum of electronic and zero-point Energies= -2191.036594
Sum of electronic and thermal Energies= -2190.990424
Sum of electronic and thermal Enthalpies= -2190.989479
Sum of electronic and thermal Free Energies= -2191.118765

Ni	-2.59626900	3.75197000	-1.01764100
N	-0.91328600	2.32528900	-0.84344000
N	-3.59513400	2.17611200	-0.35014800
C	0.46129600	2.48073400	-1.18453100
C	0.76654900	2.61420700	-2.55497300
C	1.44548300	2.57662100	-0.18097200
C	2.09797600	2.84038400	-2.91280700
C	2.76391400	2.81664300	-0.59014600
C	3.09170200	2.94605800	-1.93852300
H	2.35442500	2.93547000	-3.96437100
H	3.53978400	2.90271600	0.16599500
H	4.12066500	3.13228300	-2.23087300
C	-4.99211300	2.06845400	-0.00756400
C	-5.85739900	1.37221300	-0.86585500
C	-5.41241000	2.63360200	1.20733800
C	-7.18955200	1.22768600	-0.45861500
C	-6.74720400	2.45094300	1.58023700
C	-7.62957700	1.74821100	0.75823300
H	-7.88137300	0.69264900	-1.10350600
H	-7.09487500	2.87010900	2.52047900
H	-8.66440100	1.61770200	1.06084100
C	1.12141800	2.39777400	1.28463900
H	1.14688500	1.33750300	1.56621200

H	1.85102800	2.92017000	1.90999600
H	0.12547000	2.77175700	1.54045700
C	-0.32233300	2.47212100	-3.59081800
H	-0.72323400	1.45067100	-3.60960300
H	-1.17075900	3.13569600	-3.37593600
H	0.05092900	2.70588600	-4.59151100
C	-4.44279900	3.40768200	2.06223300
H	-4.02255800	4.24018600	1.48435000
H	-4.93641100	3.81948200	2.94635400
H	-3.60659500	2.78277300	2.40013300
C	-5.35776200	0.74872100	-2.14446500
H	-4.64368100	1.40341300	-2.64845000
H	-4.85615000	-0.20775800	-1.94608000
H	-6.18571800	0.55263300	-2.83107800
C	-2.84816600	1.15342000	-0.07516800
C	-1.37887200	1.21050400	-0.40784600
C	-1.70648700	5.76352200	-0.49389300
H	-2.55280400	6.44438900	-0.44623500
C	-1.26580500	5.35969800	-1.72965000
H	-1.73634500	5.74704400	-2.62730000
H	-0.31219700	4.84929000	-1.83260000
Si	-4.46355100	4.83779500	-1.74266100
O	-3.99847100	6.26151600	-2.47009800
O	-5.21901800	3.87705400	-2.87225200
O	-5.55364100	5.15163700	-0.55044500
C	-4.95557400	7.27358500	-2.80659300
H	-4.40813100	8.12724800	-3.21436700
H	-5.66396200	6.91313600	-3.56345900
H	-5.51244600	7.59576800	-1.91893400
C	-4.70711100	3.71672400	-4.19389100
H	-4.49289000	4.68458200	-4.66222900
H	-3.78515300	3.11747500	-4.18989500
H	-5.46158000	3.19043300	-4.78428200
C	-6.97531700	5.02174900	-0.66623100
H	-7.39566300	5.09935100	0.33855600
H	-7.38573200	5.82470600	-1.29041400
H	-7.24718500	4.05319200	-1.09400100
C	-4.07513100	-2.13471400	1.50647000
C	-2.87845600	-2.82833600	1.40928500
C	-1.72538200	-2.20619000	0.85624000
C	-1.87929100	-0.87365700	0.42763500
C	-3.11356000	-0.16508400	0.50654400
C	-4.21810400	-0.79786100	1.05597700
H	-0.26112800	-3.80570500	1.00080400

H	-4.93984200	-2.62931600	1.93770900
H	-2.81644800	-3.85500600	1.75954000
C	-0.43414000	-2.77926300	0.68941700
C	-0.81415900	-0.11133400	-0.12437000
H	-5.17524800	-0.29925100	1.14626600
C	0.43202800	-0.69384000	-0.27583800
C	0.60033100	-2.04087400	0.13499100
H	1.26729000	-0.14339200	-0.69408100
H	1.57552900	-2.50230900	0.01513900
C	-0.97721900	5.50032500	0.79033900
H	-0.61924600	6.45222600	1.20503700
H	-1.64237000	5.05803800	1.54157300
H	-0.11809900	4.84429200	0.63695500

UB3LYP-D3/BSII(SMD)//B3LYP-D3/BSI

HF=-2193.9394361

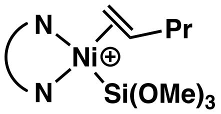

Triplet

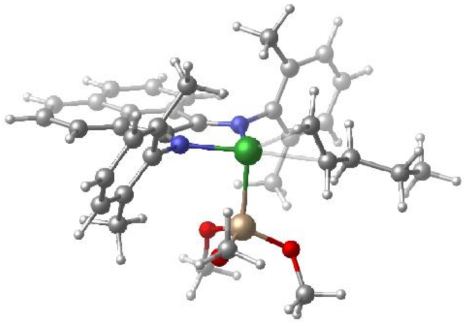

Zero-point correction= 0.717167 (Hartree/Particle)
Thermal correction to Energy= 0.764015
Thermal correction to Enthalpy= 0.764959
Thermal correction to Gibbs Free Energy= 0.633251
Sum of electronic and zero-point Energies= -2191.039948
Sum of electronic and thermal Energies= -2190.993100
Sum of electronic and thermal Enthalpies= -2190.992155
Sum of electronic and thermal Free Energies= -2191.123864

Ni	-2.89529800	3.22147900	-1.93293100
N	-1.30713600	2.42292800	-0.94962000
N	-3.85584700	1.58341900	-1.19149600
C	0.05347900	2.85399200	-1.04008900
C	0.77813300	2.39399900	-2.15791400
C	0.59660600	3.75732800	-0.10999900
C	2.07607700	2.87706000	-2.34538300
C	1.90296300	4.20932200	-0.33776900
C	2.63510100	3.78519800	-1.44545700
H	2.65119100	2.53337700	-3.20091400
H	2.34414300	4.91036800	0.36558200
H	3.64374400	4.15522500	-1.60330700
C	-5.21931100	1.26690800	-1.46497800
C	-5.50778100	0.91470700	-2.80516800
C	-6.23382100	1.39030900	-0.49313300
C	-6.83505500	0.67947900	-3.16075600
C	-7.55415300	1.15609100	-0.90791600
C	-7.85866000	0.80690400	-2.21899500
H	-7.06800500	0.38985900	-4.18172500
H	-8.35133300	1.25669000	-0.17581300
H	-8.88999400	0.63114200	-2.51011300
C	-0.19440900	4.26140100	1.07170000
H	-0.82135200	3.48379600	1.51376600
H	0.47743900	4.64780800	1.84381100

H	-0.86837300	5.07523800	0.77810900
C	0.17050700	1.38163300	-3.09910400
H	0.10540400	0.39263900	-2.62696600
H	-0.85295400	1.65512400	-3.38266200
H	0.76766100	1.27784700	-4.00914500
C	-5.98596100	1.76246200	0.94928300
H	-4.98108100	2.13724600	1.13394200
H	-6.68494400	2.54597700	1.25716500
H	-6.15156400	0.89563500	1.60155300
C	-4.39462600	0.74086000	-3.81094700
H	-3.80649900	1.65955400	-3.93706600
H	-3.68772300	-0.03685700	-3.49771400
H	-4.79107100	0.46459900	-4.79166300
C	-3.10208400	0.95257400	-0.34935700
C	-1.67987000	1.42505100	-0.21990600
C	-2.25115900	5.21779900	-3.15991500
H	-3.09305900	5.86872400	-2.92640200
C	-2.45621000	4.16396100	-3.99584400
H	-3.42523100	4.00013400	-4.46598100
C	-0.92374900	5.65197600	-2.61518700
H	-0.14959300	4.92221000	-2.87379100
H	-0.99554500	5.68856300	-1.52349000
C	-0.52071500	7.05276900	-3.11787800
H	-0.45198600	7.04385100	-4.21324900
C	0.81384900	7.49372800	-2.50850100
H	1.61224900	6.78444600	-2.75646600
H	1.10968800	8.48109600	-2.87750800
H	0.74559300	7.54650500	-1.41522100
H	-1.30771600	7.77295000	-2.85644100
H	-1.62631500	3.55564900	-4.34945600
Si	-3.74064200	4.57610700	-0.17581300
O	-3.06387200	6.09800500	-0.14575200
O	-5.40094700	4.70432600	-0.28068100
O	-3.40136100	3.79898000	1.25414900
C	-3.72681000	7.30991700	0.22501600
H	-3.56172600	7.51907500	1.28873100
H	-3.30289600	8.12811500	-0.36496100
H	-4.80540500	7.25198200	0.04080400
C	-6.10751800	4.76797100	-1.51420700
H	-5.69047300	4.06909200	-2.25594200
H	-7.14475500	4.47736500	-1.32967000
H	-6.08984500	5.78263600	-1.93322200
C	-3.45287900	4.44577400	2.52762200
H	-3.07342800	3.74422200	3.27580600

H	-2.82622400	5.34600300	2.53832300
H	-4.48269300	4.71892000	2.78835600
C	-4.00976400	-2.19593000	1.66093500
C	-2.77586300	-2.37368900	2.26832300
C	-1.70386400	-1.47629700	2.00338900
C	-1.98029800	-0.41849000	1.11819400
C	-3.25165400	-0.22297000	0.50701900
C	-4.27008200	-1.12195400	0.77115700
H	-0.11256100	-2.32587200	3.21699800
H	-4.80921100	-2.89949100	1.87150900
H	-2.62079800	-3.20779900	2.94707800
C	-0.38013300	-1.53372100	2.52308000
C	-1.00488500	0.54003800	0.72602700
H	-5.24701000	-1.02110000	0.31259400
C	0.27696000	0.45635700	1.23996900
C	0.56845600	-0.59369000	2.14858800
H	1.04572000	1.16870300	0.96200100
H	1.57142200	-0.66225300	2.55818500

UB3LYP-D3/BSII(SMD)//B3LYP-D3/BSI

HF=-2193.9501853

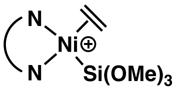

Singlet

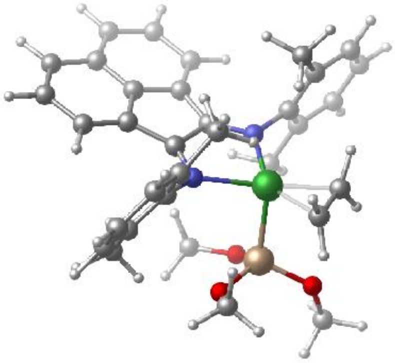

Zero-point correction= 0.632777 (Hartree/Particle)
Thermal correction to Energy= 0.674359
Thermal correction to Enthalpy= 0.675303
Thermal correction to Gibbs Free Energy= 0.558346
Sum of electronic and zero-point Energies= -2073.170529
Sum of electronic and thermal Energies= -2073.128948
Sum of electronic and thermal Enthalpies= -2073.128004
Sum of electronic and thermal Free Energies= -2073.244961

Ni	-0.44993500	-1.37432100	0.75065500
N	1.38401800	-0.51561000	0.34043400
N	-1.11333100	0.53212300	0.30955700
C	2.66345300	-1.12513100	0.53744500
C	3.13350600	-1.18569300	1.86291500
C	3.37616600	-1.67303500	-0.54268300
C	4.35548200	-1.82425000	2.09604900
C	4.59389900	-2.30316200	-0.25968300
C	5.08098400	-2.38343200	1.04486300
H	4.73902400	-1.87697500	3.11142500
H	5.16190200	-2.73769900	-1.07783000
H	6.02603400	-2.88069800	1.24125400
C	-2.41728300	1.05701800	0.61178400
C	-2.75979700	1.10536800	1.97987200
C	-3.30647200	1.48145500	-0.39281500
C	-4.04051900	1.54194700	2.32812100
C	-4.57924500	1.91223300	0.00819700
C	-4.95364400	1.93187900	1.34910100
H	-4.31564500	1.58755400	3.37825500
H	-5.28237800	2.23796100	-0.75368900
H	-5.94714800	2.26591600	1.63241200
C	2.84940000	-1.57638100	-1.95299800
H	2.78324600	-0.53146200	-2.28401300

H	3.50695800	-2.10206300	-2.65065800
H	1.84479900	-2.00176100	-2.03522100
C	2.36478300	-0.52115400	2.97978300
H	2.41886700	0.57290400	2.90200600
H	1.29876200	-0.78001300	2.95358500
H	2.76150500	-0.80558500	3.95806100
C	-2.95014700	1.47369800	-1.85897300
H	-3.02110000	0.45931100	-2.26022900
H	-3.63602400	2.11335900	-2.42166500
H	-1.93534500	1.83704100	-2.04320000
C	-1.74505000	0.76589700	3.04577200
H	-1.35278100	-0.25198500	2.93412200
H	-0.88122400	1.44112800	3.00356000
H	-2.18258100	0.84354300	4.04459000
C	-0.12970300	1.31135800	0.00964800
C	1.26145500	0.70611500	-0.03647400
C	-1.42887600	-2.85270300	1.70945000
H	-1.84288900	-2.37877600	2.59944300
C	-0.04608500	-3.10608000	1.60089200
Si	-1.38352000	-2.24212200	-1.09623800
O	-1.30923000	-3.88100300	-1.04334500
O	-2.89837300	-1.63828500	-1.29526900
O	-0.45066400	-1.78218600	-2.36673800
C	-1.18708700	-4.72366600	-2.20101700
H	-1.01610200	-5.74108800	-1.84263500
H	-2.10861700	-4.70152400	-2.79356400
H	-0.34599400	-4.40855100	-2.82603800
C	-3.99959700	-1.81809900	-0.39914500
H	-3.74527200	-1.45890200	0.60385500
H	-4.83120100	-1.22357200	-0.78220200
H	-4.29496700	-2.87218000	-0.35372800
C	-0.38177600	-0.58065300	-3.11922500
H	-0.08273300	0.26537900	-2.49011800
H	0.37486800	-0.72313600	-3.89481000
H	-1.34356500	-0.35417800	-3.58826500
C	-0.30129100	5.11778600	-0.51415100
C	1.05076100	5.32081100	-0.74835100
C	1.96277500	4.22955300	-0.72130400
C	1.41092800	2.96154100	-0.45799800
C	0.02151100	2.74908200	-0.22365000
C	-0.84018000	3.83380300	-0.24766800
H	3.84913100	5.22954500	-1.12994000
H	-0.97533600	5.96836800	-0.53471500
H	1.42097400	6.32230200	-0.94968800

C	3.37143900	4.27689200	-0.91840500
C	2.19689900	1.77946900	-0.37038300
H	-1.90249300	3.71732200	-0.06852900
C	3.56613600	1.85477800	-0.55571900
C	4.13642400	3.12313400	-0.83686300
H	4.19503500	0.97400000	-0.48926900
H	5.20935100	3.18779000	-0.98816300
H	0.31259300	-3.85636600	0.90148900
H	-2.13599700	-3.47244300	1.16632200
H	0.63964500	-2.85985800	2.41131600

UB3LYP-D3/BSII(SMD)//B3LYP-D3/BSI

HF=-2075.957666

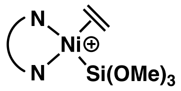

Triplet

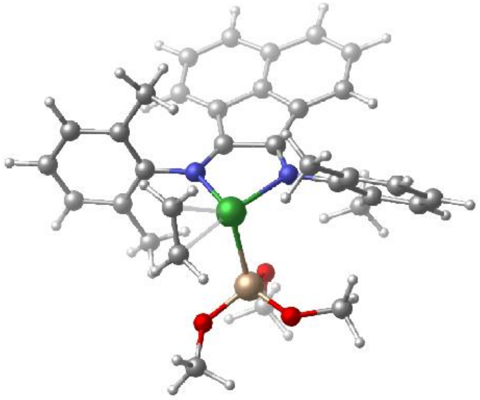

Zero-point correction= 0.631719 (Hartree/Particle)
Thermal correction to Energy= 0.674155
Thermal correction to Enthalpy= 0.675099
Thermal correction to Gibbs Free Energy= 0.553646
Sum of electronic and zero-point Energies= -2073.165057
Sum of electronic and thermal Energies= -2073.122622
Sum of electronic and thermal Enthalpies= -2073.121678
Sum of electronic and thermal Free Energies= -2073.243131

Ni	0.60573800	-1.12841500	0.92238700
N	0.93646900	0.86447600	0.59170700
N	-1.27474300	-0.66837300	0.31716000
C	2.19276300	1.52267700	0.69868600
C	2.86144700	1.40375900	1.93861100
C	2.79622800	2.16074600	-0.40997400
C	4.10979700	2.00840100	2.08467400
C	4.05721900	2.74101600	-0.21358300
C	4.70490400	2.68289600	1.01695300
H	4.62240300	1.94406200	3.04040800
H	4.54053100	3.23115400	-1.05451800
H	5.68008600	3.14405800	1.14104600
C	-2.42947900	-1.51400400	0.35230100
C	-3.26273800	-1.44253300	1.48946100
C	-2.67237600	-2.40591700	-0.70620500
C	-4.35715200	-2.31051000	1.55217600
C	-3.79098700	-3.24296600	-0.60291300
C	-4.62244000	-3.20489000	0.51462100
H	-5.00939400	-2.27606400	2.42063900
H	-4.00671800	-3.93127900	-1.41578900
H	-5.48008000	-3.86808300	0.57632900
C	2.18494600	2.17805600	-1.79161300

H	1.55452700	3.06050300	-1.95025800
H	2.97703100	2.20878300	-2.54625900
H	1.57834200	1.28910700	-1.98074900
C	2.25171300	0.61845500	3.07522900
H	1.19640600	0.86212600	3.23566500
H	2.31327300	-0.46315400	2.87324900
H	2.79011100	0.79453900	4.01052100
C	-1.77320900	-2.46857100	-1.91660900
H	-1.31033600	-1.50418000	-2.14150000
H	-0.95547700	-3.18163400	-1.76109700
H	-2.33609700	-2.79677600	-2.79595400
C	-3.00613800	-0.43714300	2.58892500
H	-1.94385100	-0.37976900	2.85408700
H	-3.31019400	0.57147600	2.28136500
H	-3.57074500	-0.69133200	3.49007300
C	-1.37284300	0.58703300	0.03227800
C	-0.14814200	1.44661900	0.18219100
C	0.72560700	-3.25669800	1.73221100
H	1.79814700	-3.40802200	1.83932400
C	0.00934100	-2.57196800	2.66540900
H	-1.07486500	-2.54243400	2.62197800
Si	1.77449000	-1.96127000	-0.97335200
O	1.37908600	-3.52411600	-1.36394200
O	3.41318800	-1.98490100	-0.70833400
O	1.33439100	-0.98422900	-2.24031100
C	2.19781800	-4.68408900	-1.20305400
H	3.25742100	-4.44926900	-1.34025300
H	2.05589800	-5.11757400	-0.20469700
H	1.88459000	-5.41870600	-1.95004300
C	4.31819500	-0.88395600	-0.77615700
H	5.31807900	-1.28776700	-0.95765000
H	4.05517700	-0.19833400	-1.58912100
H	4.32480600	-0.32745700	0.16712200
C	1.45810200	-1.38147000	-3.61394800
H	1.07388900	-0.56309400	-4.22839800
H	2.50636400	-1.56376300	-3.87984600
H	0.87625900	-2.28797400	-3.80990300
C	-4.59518800	2.37937200	-1.04054700
C	-4.07424400	3.66443300	-1.06404900
C	-2.70679900	3.89921100	-0.74747300
C	-1.93471500	2.76470300	-0.43553900
C	-2.47418700	1.45018000	-0.39021000
C	-3.80928700	1.25005400	-0.69455400
H	-2.58338400	6.06273900	-0.92829700

H	-5.64197900	2.22809700	-1.28484100
H	-4.71445600	4.50377900	-1.32129600
C	-2.03678500	5.15289000	-0.69628000
C	-0.55123500	2.82595500	-0.10182700
H	-4.25457400	0.26187800	-0.66704600
C	0.06683000	4.06403800	-0.04246600
C	-0.69756200	5.22025000	-0.34315100
H	1.11310500	4.16224500	0.22424700
H	-0.20972100	6.18899000	-0.29893000
H	0.23434900	-3.81029100	0.93736600
H	0.48575000	-2.11611900	3.53031300

UB3LYP-D3/BSII(SMD)//B3LYP-D3/BSI

HF=-2075.9564871

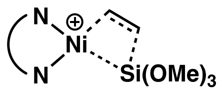

Singlet

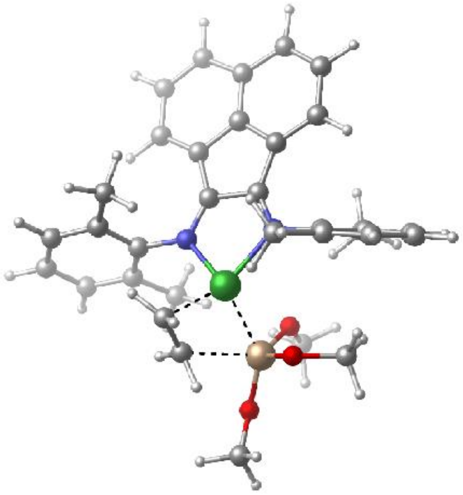

Zero-point correction= 0.632681 (Hartree/Particle)
Thermal correction to Energy= 0.673272
Thermal correction to Enthalpy= 0.674216
Thermal correction to Gibbs Free Energy= 0.559482
Sum of electronic and zero-point Energies= -2073.165636
Sum of electronic and thermal Energies= -2073.125045
Sum of electronic and thermal Enthalpies= -2073.124100
Sum of electronic and thermal Free Energies= -2073.238835

Ni	-0.74681200	-1.09767700	0.57988300
N	1.17672100	-1.00393000	0.24387400
N	-0.69259100	0.92693500	0.16742400
C	2.12531500	-2.06537900	0.42061000
C	2.81351300	-2.13210300	1.64444000
C	2.29299400	-3.01287800	-0.60070700
C	3.71656200	-3.18555700	1.82283200
C	3.21027400	-4.04617300	-0.37930000
C	3.91880300	-4.13309800	0.81931300
H	4.26096400	-3.26098200	2.76024700
H	3.36287500	-4.79031200	-1.15626900
H	4.62326200	-4.94470500	0.97459700
C	-1.66229100	1.93027500	0.51887800
C	-1.84155600	2.16340800	1.89838000
C	-2.39618100	2.62592600	-0.45598400
C	-2.81342000	3.08946300	2.29009800
C	-3.35143500	3.55151600	-0.01440400
C	-3.57105500	3.77669000	1.34252400
H	-2.96352800	3.28030200	3.34922500
H	-3.92904600	4.09880700	-0.75461800

H	-4.32082700	4.49453500	1.66117200
C	1.50723900	-2.90153800	-1.88257300
H	1.76106900	-1.98375300	-2.42889700
H	1.70954600	-3.74914200	-2.54292400
H	0.42993200	-2.86489900	-1.68440200
C	2.58363700	-1.09261800	2.71501300
H	3.00013300	-0.11879800	2.42578100
H	1.51279200	-0.93786900	2.89746700
H	3.05329600	-1.38765100	3.65697900
C	-2.19980900	2.38788000	-1.93125100
H	-2.72797800	1.47879000	-2.23279200
H	-2.59910300	3.22248900	-2.51450100
H	-1.14470400	2.26972100	-2.19687500
C	-0.96755400	1.48103800	2.92474500
H	-0.96812900	0.39080900	2.81104500
H	0.07814800	1.80112200	2.83037300
H	-1.29735700	1.71648600	3.94006200
C	0.52111700	1.26728700	-0.10739300
C	1.55530800	0.16731200	-0.13879800
C	-2.23604600	-2.00614100	1.57439000
H	-2.46795600	-1.25243000	2.32940300
C	-0.95317300	-2.68324200	1.62901200
Si	-2.68195100	-1.62395300	-0.62831000
O	-3.49798400	-3.05500600	-0.61578800
O	-3.63320900	-0.29584400	-0.74641400
O	-1.72344900	-1.75016500	-1.96365900
C	-3.98013400	-3.69992100	-1.80522900
H	-4.38189700	-4.66981300	-1.50447900
H	-4.77824300	-3.11013200	-2.27041500
H	-3.16796600	-3.84525000	-2.52360900
C	-4.58978800	0.13924000	0.22700900
H	-4.08335300	0.47672900	1.13766600
H	-5.12912100	0.98506100	-0.20239000
H	-5.29983600	-0.66043700	0.46646200
C	-1.16082000	-0.81085100	-2.87005600
H	-0.50405900	-0.10527100	-2.35274900
H	-0.57370200	-1.37475400	-3.59879200
H	-1.94660700	-0.25669000	-3.39125100
C	1.86242300	4.82541800	-0.67044500
C	3.18450600	4.47498100	-0.90215500
C	3.59249200	3.11250000	-0.85767900
C	2.58605000	2.17024400	-0.57600200
C	1.22669100	2.52784700	-0.34076900
C	0.86051300	3.86253600	-0.38605800

H	5.71837200	3.27675700	-1.28120500
H	1.57917700	5.87267100	-0.70676300
H	3.91903200	5.24626700	-1.11696400
C	4.90295200	2.59442000	-1.05753300
C	2.83969800	0.77278500	-0.47752500
H	-0.16216300	4.17516500	-0.20915100
C	4.12491800	0.29605000	-0.66762000
C	5.14846800	1.23209100	-0.96500800
H	4.35221600	-0.76162100	-0.59238600
H	6.15817300	0.86523900	-1.12036200
H	-0.85520800	-3.64315400	1.12235600
H	-3.10970300	-2.62335400	1.36466300
H	-0.30349800	-2.56900400	2.49718100

UB3LYP-D3/BSII(SMD)//B3LYP-D3/BSI

HF=-2075.9505901

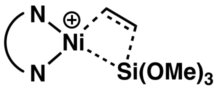

Triplet

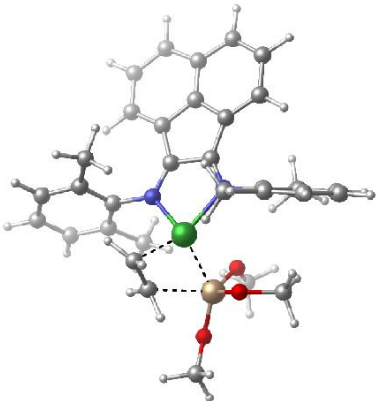

Zero-point correction= 0.631491 (Hartree/Particle)
Thermal correction to Energy= 0.672696
Thermal correction to Enthalpy= 0.673640
Thermal correction to Gibbs Free Energy= 0.556584
Sum of electronic and zero-point Energies= -2073.153249
Sum of electronic and thermal Energies= -2073.112044
Sum of electronic and thermal Enthalpies= -2073.111100
Sum of electronic and thermal Free Energies= -2073.228155

Ni	1.00797300	-0.63615500	0.81287100
N	0.33395500	1.21612100	0.47379100
N	-0.89560200	-1.16504300	0.23841800
C	1.11941700	2.40526900	0.55179000
C	1.62968600	2.76165000	1.81673900
C	1.43957000	3.12846100	-0.61654400
C	2.44156500	3.89560200	1.90486400
C	2.26486100	4.25156800	-0.47700600
C	2.75766000	4.63966600	0.76736700
H	2.82831600	4.19498500	2.87505900
H	2.52858100	4.82054600	-1.36453500
H	3.39430200	5.51545500	0.85067800
C	-1.46730100	-2.47125900	0.29671000
C	-2.20194300	-2.84316200	1.44244600
C	-1.20499100	-3.37157100	-0.75190800
C	-2.67026600	-4.15945900	1.51564100
C	-1.69853600	-4.67570800	-0.63496600
C	-2.42209800	-5.07090700	0.48907200
H	-3.23280700	-4.46995700	2.39197600
H	-1.51239500	-5.38368200	-1.43823200
H	-2.79139500	-6.08906600	0.56707000
C	0.95426500	2.70442900	-1.98273900

H	-0.08088400	3.01873300	-2.16186900
H	1.57130000	3.15960200	-2.76313200
H	0.99972900	1.61795500	-2.09933300
C	1.29494600	1.94593400	3.04165900
H	0.21250000	1.84810000	3.18334400
H	1.69686500	0.92658900	2.96220900
H	1.71727400	2.39907300	3.94256200
C	-0.47593000	-2.92263700	-1.99442800
H	0.29622200	-2.18354900	-1.76777600
H	-0.01495600	-3.77013400	-2.51044100
H	-1.16799900	-2.44316000	-2.69995700
C	-2.48845900	-1.85423100	2.54877300
H	-1.60220600	-1.26358600	2.80838600
H	-3.27015400	-1.14318900	2.25330300
H	-2.83297400	-2.36780200	3.45036200
C	-1.60126900	-0.10871700	0.00940000
C	-0.91182500	1.22107900	0.11226400
C	2.00809300	-2.51062500	1.12384700
H	3.09048100	-2.55889000	1.22899000
C	1.30776700	-2.08749800	2.29037700
H	0.32458600	-2.48815900	2.51190100
Si	2.73146500	-1.16683000	-0.71543500
O	3.67495400	-2.37331200	-1.30957000
O	3.52892800	-0.07260900	0.24681500
O	2.07605800	-0.45479100	-2.04914300
C	4.78984400	-3.02017200	-0.69445700
H	5.28127400	-2.36932600	0.03883800
H	4.46518000	-3.94431200	-0.20343200
H	5.50647600	-3.27192900	-1.48062200
C	4.20836700	1.10921700	-0.22280600
H	5.27303200	0.88533800	-0.34330100
H	3.78993600	1.45014500	-1.17363900
H	4.07589600	1.89091400	0.52654300
C	2.26735900	-0.81299700	-3.42545800
H	1.46026900	-1.47803900	-3.74951700
H	2.22871200	0.10970800	-4.01077300
H	3.22763300	-1.31197000	-3.57884700
C	-5.33754300	-0.01829600	-0.88209800
C	-5.46013900	1.36076100	-0.96165400
C	-4.33699600	2.20331200	-0.72818900
C	-3.12400800	1.55675500	-0.42830400
C	-3.00022300	0.14296000	-0.33627900
C	-4.10859300	-0.65274400	-0.56362200
H	-5.21102800	4.17836600	-0.98182200

H	-6.21012700	-0.63825200	-1.06290000
H	-6.42189100	1.80616700	-1.20074400
C	-4.30341700	3.62547500	-0.75585100
C	-1.90819100	2.25378600	-0.17199500
H	-4.05233400	-1.73383300	-0.49643000
C	-1.90920800	3.63819700	-0.19942800
C	-3.12476600	4.30776800	-0.49351600
H	-1.00728800	4.20866600	-0.00744500
H	-3.12565700	5.39309300	-0.51622800
H	1.57419400	-3.31401900	0.53141900
H	1.85343400	-1.63592400	3.11554000

UB3LYP-D3/BSII(SMD)//B3LYP-D3/BSI

HF=-2075.940064

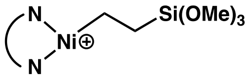

Triplet

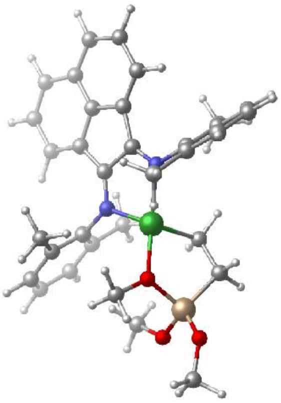

Zero-point correction= 0.631789 (Hartree/Particle)
Thermal correction to Energy= 0.673374
Thermal correction to Enthalpy= 0.674318
Thermal correction to Gibbs Free Energy= 0.553785
Sum of electronic and zero-point Energies= -2073.195402
Sum of electronic and thermal Energies= -2073.153817
Sum of electronic and thermal Enthalpies= -2073.152873
Sum of electronic and thermal Free Energies= -2073.273406

Ni	0.88549000	0.61374100	0.07564700
N	-0.10218900	-1.17959300	0.12559500
N	-1.02659600	1.34848500	0.16679400
C	0.51906700	-2.46493200	0.11218800
C	0.68198600	-3.14736500	-1.10727200
C	1.03056100	-2.95625700	1.32723900
C	1.40570800	-4.34529600	-1.08715700
C	1.74739800	-4.15720500	1.29863700
C	1.94199800	-4.84424600	0.10037100
H	1.55124400	-4.88757400	-2.01750000
H	2.14933500	-4.55599900	2.22619900
H	2.50693400	-5.77150100	0.09224900
C	-1.33823000	2.73317900	0.09738900
C	-1.92563500	3.28976900	-1.05877000
C	-0.92358200	3.53053900	1.18601200
C	-2.14357100	4.67360200	-1.06929200
C	-1.17817600	4.90147900	1.13385800
C	-1.78929900	5.47199600	0.01591300
H	-2.58837400	5.12672800	-1.95120300
H	-0.88839600	5.52722400	1.97343200
H	-1.97263600	6.54178400	-0.01521300
C	0.74723800	-2.23562800	2.62283800
H	-0.27999600	-2.42779200	2.95919600

H	1.42273500	-2.56361200	3.41794400
H	0.84081200	-1.15001500	2.51313000
C	0.08526300	-2.61117100	-2.38586000
H	-1.00650200	-2.72443000	-2.39034800
H	0.29567800	-1.54467400	-2.51335600
H	0.47642700	-3.14563400	-3.25565400
C	-0.23638900	2.90545800	2.37691900
H	0.73455900	2.46968400	2.09426400
H	-0.04217000	3.65003100	3.15357100
H	-0.83242600	2.09882600	2.81912900
C	-2.28130100	2.45966600	-2.27035100
H	-1.58314700	1.62952500	-2.41671300
H	-3.28612600	2.02776400	-2.18860500
H	-2.26366600	3.07697400	-3.17287900
C	-1.90303600	0.40054300	0.11447000
C	-1.38062900	-1.01390100	0.10019000
C	2.87253400	1.76696000	-1.70245600
H	3.38292400	1.83477600	-2.67549800
C	1.50802300	1.06964400	-1.81004300
H	0.74449900	1.70704000	-2.26798200
Si	3.96559400	0.90330200	-0.46925800
O	4.62565800	-0.53896400	-0.89692500
O	2.82234000	0.59774900	0.78510300
O	5.15164700	1.84327100	0.13489500
C	3.90358100	-1.74778600	-1.13404400
H	3.29418800	-2.02687900	-0.26716700
H	3.24851800	-1.65450600	-2.00830700
H	4.63095300	-2.54032200	-1.32483400
C	3.27041400	0.17361200	2.08737400
H	4.21934000	0.66154700	2.32532700
H	2.51975900	0.47650700	2.82062300
H	3.38949500	-0.91373500	2.10629700
C	6.50806800	1.49750700	0.43546400
H	7.16260700	2.24058800	-0.02798600
H	6.65344400	1.52351700	1.52095300
H	6.76192600	0.50226000	0.05802400
C	-5.72997500	0.76603000	0.20077600
C	-6.03305100	-0.58702900	0.17332500
C	-4.99555300	-1.55965500	0.12427600
C	-3.67816200	-1.06577500	0.09857300
C	-3.36461000	0.32291300	0.11360800
C	-4.39486300	1.24529600	0.17048200
H	-6.13292000	-3.41235800	0.11823000
H	-6.53898100	1.48853400	0.24374800

H	-7.07000500	-0.91066900	0.19286300
C	-5.13814500	-2.97554600	0.10567300
C	-2.53383200	-1.91071800	0.07553900
H	-4.19825700	2.31178200	0.18902500
C	-2.70091100	-3.28434100	0.06257200
C	-4.02259800	-3.79963400	0.07455900
H	-1.85072900	-3.95763000	0.04453200
H	-4.16060200	-4.87625200	0.06213700
H	2.74916100	2.79922700	-1.34791800
H	1.57094900	0.12934500	-2.36749900

UB3LYP-D3/BSII(SMD)//B3LYP-D3/BSI

HF=-2075.9869062

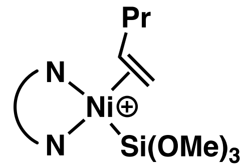

Singlet

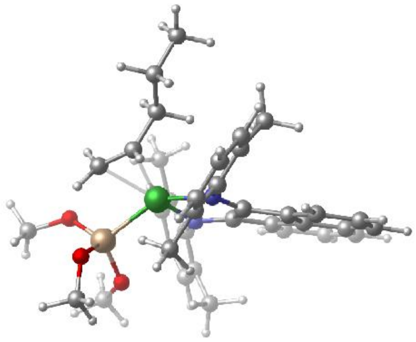

Zero-point correction= 0.719843 (Hartree/Particle)
Thermal correction to Energy= 0.765347
Thermal correction to Enthalpy= 0.766291
Thermal correction to Gibbs Free Energy= 0.640701
Sum of electronic and zero-point Energies= -2191.036710
Sum of electronic and thermal Energies= -2190.991207
Sum of electronic and thermal Enthalpies= -2190.990262
Sum of electronic and thermal Free Energies= -2191.115852

Ni	-2.63171900	3.44826700	-1.56175900
N	-1.02601500	2.24813100	-0.88589000
N	-3.71114500	1.92924300	-0.93911300
C	0.36051600	2.57661700	-0.82819900
C	1.31238700	1.88733900	-1.60796700
C	0.71035800	3.69693800	-0.04018900
C	2.63717000	2.34726300	-1.57074000
C	2.04587700	4.10185500	-0.02429700
C	3.00656800	3.43696000	-0.78850900
H	3.38369200	1.83409900	-2.17153400
H	2.33299300	4.95037700	0.59055100
H	4.03940700	3.77166800	-0.77507400
C	-5.13291500	1.74029100	-1.05882700
C	-5.69720500	1.79173900	-2.34595000
C	-5.90770500	1.52172600	0.09756500
C	-7.07473300	1.57882500	-2.46643400
C	-7.28026000	1.31329300	-0.07592100
C	-7.86253400	1.33376600	-1.34304300
H	-7.52709500	1.60358100	-3.45391400
H	-7.89932400	1.14641100	0.80156200
H	-8.93027300	1.16925100	-1.45327800
C	-0.33487300	4.42251700	0.77247300

H	-0.88911800	3.73823400	1.42550500
H	0.12602400	5.19015600	1.40044100
H	-1.07917100	4.91636700	0.13365700
C	0.98622100	0.68663400	-2.46745200
H	1.30659200	-0.24204900	-1.97986000
H	-0.08083500	0.58404100	-2.67619800
H	1.51056100	0.74982600	-3.42587800
C	-5.30415700	1.54307800	1.48129800
H	-4.63074400	2.39787800	1.59162800
H	-6.08933800	1.62664500	2.23810400
H	-4.73967300	0.62942200	1.69972200
C	-4.84552100	2.06865800	-3.56019100
H	-4.48763900	3.10612500	-3.54544300
H	-3.96217300	1.42106500	-3.60086400
H	-5.41567300	1.92397900	-4.48185300
C	-2.97669400	0.91542300	-0.59940000
C	-1.49613100	1.13260400	-0.44866300
C	-2.34873100	5.19691600	-2.88548300
H	-3.12930000	5.14859600	-3.63838000
C	-1.28130800	4.32926500	-2.91699900
Si	-4.15548700	5.02914400	-0.92798000
O	-5.13345900	5.59078400	-2.14005300
O	-5.08822400	4.43850700	0.31025500
O	-3.24124100	6.25939600	-0.27548800
C	-5.78093900	6.86610900	-2.14270600
H	-5.15652600	7.63152400	-1.66634100
H	-5.96091900	7.15142100	-3.18279200
H	-6.74371900	6.81127000	-1.62076900
C	-6.49866400	4.64831500	0.40519600
H	-7.00669000	4.36430200	-0.52200800
H	-6.87294400	4.02126400	1.21747600
H	-6.72222900	5.69724600	0.63803100
C	-3.30970100	6.75743600	1.06188900
H	-2.30699300	6.72506200	1.50080700
H	-3.65145900	7.79786500	1.03708800
H	-3.99157700	6.16528100	1.67977100
C	-4.20407700	-2.66392300	0.11642200
C	-3.00561100	-3.15998100	0.60685700
C	-1.85106900	-2.33180500	0.66509100
C	-2.00470000	-1.01149200	0.19977100
C	-3.24332500	-0.48925500	-0.27313700
C	-4.34828200	-1.32353000	-0.32274500
H	-0.38546100	-3.69330700	1.51501300
H	-5.06989200	-3.31734100	0.07474100

H	-2.94356600	-4.18965300	0.94809300
C	-0.55797300	-2.68880000	1.13862200
C	-0.93346900	-0.07735800	0.15481400
H	-5.30963500	-0.97154500	-0.67703200
C	0.31364400	-0.45151300	0.62257100
C	0.47870700	-1.76798400	1.12400400
H	1.15245300	0.23540700	0.60766300
H	1.45446400	-2.06289500	1.49720200
C	-1.10861000	3.27773100	-3.98560000
H	-1.99646100	3.25898100	-4.62955100
H	-1.02493200	2.28171600	-3.53597300
H	-0.40321800	4.55468700	-2.31319500
H	-2.28642500	6.11674700	-2.31417100
C	0.15816000	3.53333200	-4.82753600
H	1.03081000	3.51923200	-4.16120700
H	0.10792400	4.54073600	-5.25978400
C	0.32824200	2.49425500	-5.93944400
H	-0.51862000	2.51662800	-6.63540400
H	1.23973900	2.68113000	-6.51653200
H	0.39318000	1.47995400	-5.52685300

UB3LYP-D3/BSII(SMD)//B3LYP-D3/BSI

HF=-2193.9416719

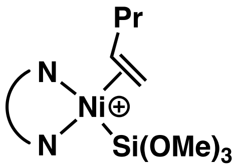

Triplet

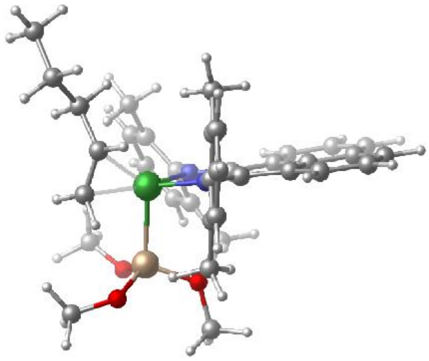

Zero-point correction= 0.717644 (Hartree/Particle)
Thermal correction to Energy= 0.764062
Thermal correction to Enthalpy= 0.765007
Thermal correction to Gibbs Free Energy= 0.635110
Sum of electronic and zero-point Energies= -2191.038324
Sum of electronic and thermal Energies= -2190.991906
Sum of electronic and thermal Enthalpies= -2190.990961
Sum of electronic and thermal Free Energies= -2191.120858

Ni	-2.93895600	3.31196100	-1.83739100
N	-1.33273000	2.45520200	-0.95216600
N	-3.91599200	1.65486700	-1.11855500
C	0.04503300	2.82103200	-1.09260600
C	0.79021700	2.15568600	-2.08927300
C	0.59343400	3.84489800	-0.30137500
C	2.12159200	2.53780400	-2.27846600
C	1.93545800	4.18284100	-0.51997700
C	2.69436400	3.54220300	-1.49769700
H	2.71150700	2.03875200	-3.04256100
H	2.38460700	4.96220400	0.08967400
H	3.73132500	3.82505900	-1.65226300
C	-5.31042800	1.39350600	-1.24946200
C	-5.78898000	1.24009300	-2.57130900
C	-6.18695200	1.39530300	-0.14182100
C	-7.15161300	1.02686500	-2.77137300
C	-7.55176100	1.19423000	-0.39881600
C	-8.03394900	1.00119500	-1.68937100
H	-7.52481200	0.88503400	-3.78184300
H	-8.24240500	1.19995200	0.44036800
H	-9.09520500	0.84247000	-1.85611100
C	-0.22362700	4.55679600	0.74690600

H	-0.87774900	3.87281300	1.29286500
H	0.42785800	5.06603200	1.46310000
H	-0.87637100	5.31544400	0.29819800
C	0.16665400	1.05813900	-2.91916500
H	0.02552300	0.14241200	-2.33104300
H	-0.82274800	1.34915700	-3.29221900
H	0.79760900	0.80788600	-3.77626800
C	-5.74912500	1.63234800	1.28354400
H	-4.77121300	2.10677300	1.35665900
H	-6.46960200	2.28580600	1.78501000
H	-5.71216800	0.69095800	1.84565000
C	-4.84091900	1.31866200	-3.74149800
H	-4.45502400	2.34167100	-3.86114200
H	-3.97216500	0.66265800	-3.62005100
H	-5.34252100	1.05515500	-4.67672500
C	-3.11312100	0.94496100	-0.38663900
C	-1.67934500	1.38709200	-0.31221200
C	-2.35023700	5.15664500	-3.00791100
H	-3.30691700	5.55046000	-3.35108700
C	-1.70086800	4.18962900	-3.71632700
H	-0.69348200	3.91527000	-3.40566900
Si	-3.83091100	4.65039500	-0.09592000
O	-3.23739100	6.20562900	-0.02795200
O	-5.49055400	4.68398900	-0.19741000
O	-3.38604200	3.92423800	1.32126900
C	-3.89888700	7.35045100	-0.56188700
H	-4.97585800	7.32212200	-0.36064500
H	-3.47049800	8.23736900	-0.08727700
H	-3.74003700	7.42658900	-1.64635900
C	-6.24647900	4.64676500	-1.40207200
H	-5.67115300	4.21170400	-2.23438300
H	-7.12748200	4.01996000	-1.24264900
H	-6.56297900	5.65719700	-1.68644000
C	-3.51199400	4.54629800	2.60694000
H	-3.20200400	5.59586800	2.56842200
H	-4.54852400	4.48468100	2.95841200
H	-2.86528400	4.00548300	3.30325200
C	-3.91472900	-2.39965500	1.34050900
C	-2.64606300	-2.64311800	1.84391100
C	-1.58681300	-1.72418800	1.60405600
C	-1.91160000	-0.57846100	0.85484200
C	-3.21984200	-0.31089300	0.35878700
C	-4.22416600	-1.23502100	0.59175500
H	0.07262500	-2.70068900	2.61324100

H	-4.70494200	-3.12059700	1.52570000
H	-2.45385400	-3.54476200	2.41884200
C	-0.23209700	-1.83988300	2.02436500
C	-0.95299100	0.40700300	0.49200800
H	-5.22937600	-1.08608400	0.21473000
C	0.35986300	0.26648100	0.90657400
C	0.69941300	-0.86999000	1.68495300
H	1.11748500	0.99708300	0.64604400
H	1.72663700	-0.98369800	2.01703800
H	-1.85977000	5.70435800	-2.20836400
C	-2.19700500	3.55497600	-4.98387900
H	-2.21301900	2.46116600	-4.87638100
H	-3.22662900	3.87381800	-5.19182800
C	-1.28786400	3.90896400	-6.18153800
H	-0.25925300	3.59974700	-5.95252000
H	-1.26698300	4.99908300	-6.30389000
C	-1.75940700	3.24011400	-7.47584200
H	-1.10435700	3.50221800	-8.31283100
H	-1.75979100	2.14758100	-7.37903100
H	-2.77695200	3.55485100	-7.73609200

UB3LYP-D3/BSII(SMD)//B3LYP-D3/BSI

HF= -2193.9490144

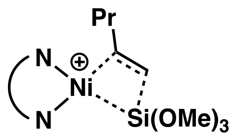

Singlet

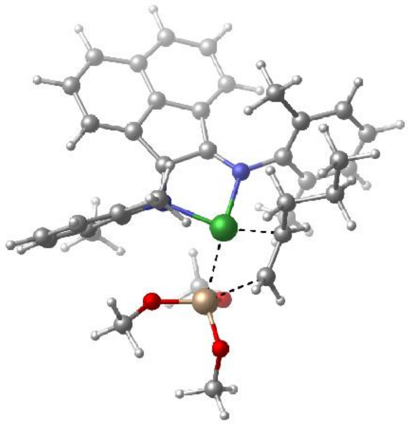

Zero-point correction= 0.719662 (Hartree/Particle)
Thermal correction to Energy= 0.764359
Thermal correction to Enthalpy= 0.765303
Thermal correction to Gibbs Free Energy= 0.641461
Sum of electronic and zero-point Energies= -2191.033427
Sum of electronic and thermal Energies= -2190.988729
Sum of electronic and thermal Enthalpies= -2190.987785
Sum of electronic and thermal Free Energies= -2191.111627

Ni	-2.65408600	3.49193300	-1.61086600
N	-1.11573900	2.33286300	-0.92506600
N	-3.78402000	1.95741400	-0.87153100
C	0.29088500	2.57659400	-1.03991500
C	1.07018500	1.78941800	-1.91108600
C	0.83009400	3.66766900	-0.32864200
C	2.41895900	2.13799400	-2.06669200
C	2.18352000	3.96486800	-0.50690100
C	2.97429100	3.21233500	-1.37625800
H	3.03596000	1.55051800	-2.74157200
H	2.61728300	4.79708600	0.04055300
H	4.02172700	3.46341200	-1.51353200
C	-5.17244600	1.65074800	-1.09089200
C	-5.57198000	1.41521300	-2.41784600
C	-6.07233300	1.58219100	-0.01186000
C	-6.90676200	1.06280600	-2.65151700
C	-7.39727200	1.23163800	-0.29396100
C	-7.81421500	0.96334100	-1.59823500
H	-7.22985000	0.86506300	-3.66993800
H	-8.11027100	1.17670900	0.52426800

H	-8.84676800	0.68991500	-1.79359200
C	-0.03445700	4.46633200	0.61355300
H	-0.42723200	3.83702800	1.42203900
H	0.53201800	5.28263700	1.06987500
H	-0.90528000	4.89814300	0.10645500
C	0.52840800	0.58121100	-2.64146700
H	0.72408000	-0.33652400	-2.07279100
H	-0.55140300	0.63143000	-2.80494700
H	1.01110400	0.47236900	-3.61730300
C	-5.63752100	1.89449600	1.39769800
H	-5.12551500	2.85934800	1.42921800
H	-6.50096400	1.94323100	2.06686300
H	-4.95471600	1.13491800	1.79615700
C	-4.58466800	1.53172900	-3.55297400
H	-4.16673800	2.54652900	-3.60596100
H	-3.73497500	0.84948000	-3.42940000
H	-5.05819500	1.31164000	-4.51354600
C	-3.03222100	1.02807300	-0.37613600
C	-1.55438500	1.29166500	-0.30616200
C	-2.53817200	5.30369200	-2.74526000
H	-3.25072900	5.38657100	-3.56338000
C	-1.44878400	4.41263700	-2.85925800
Si	-4.13079000	5.25817100	-1.02954800
O	-4.62413400	6.63600700	-1.80188300
O	-5.49456700	4.48447600	-0.51536700
O	-3.23226000	5.66755200	0.30019700
C	-5.22457900	7.72156100	-1.08423100
H	-4.50904500	8.16045300	-0.37993800
H	-5.51864500	8.47579400	-1.81795500
H	-6.11549800	7.39944200	-0.53053400
C	-6.78610500	4.57476800	-1.12692800
H	-6.94024500	3.72690300	-1.79900800
H	-7.53312600	4.53412100	-0.32996000
H	-6.90074100	5.50684400	-1.68829100
C	-3.36669500	5.10958400	1.60608100
H	-3.00790600	4.07285000	1.62423400
H	-2.75278200	5.70692700	2.28477100
H	-4.40846700	5.13378900	1.94234200
C	-4.13296800	-2.46210900	0.83613200
C	-2.89903800	-2.87526100	1.31394300
C	-1.76364000	-2.02544400	1.20861900
C	-1.97606600	-0.76980100	0.60733600
C	-3.24834400	-0.33145500	0.13700900
C	-4.33183600	-1.18837000	0.24488700

H	-0.21841900	-3.25837700	2.11129100
H	-4.98386500	-3.13101100	0.91935200
H	-2.79331800	-3.85646500	1.76845900
C	-0.43590200	-2.30630500	1.63508900
C	-0.93018800	0.16951800	0.39335000
H	-5.31682500	-0.90555800	-0.10567000
C	0.35400000	-0.13185700	0.81152800
C	0.57909600	-1.38046300	1.44498900
H	1.17545500	0.55908200	0.65854800
H	1.58334100	-1.61897800	1.78100700
C	-1.25962700	3.50072500	-4.04856200
H	-2.16290300	3.50588600	-4.67155100
H	-1.11492000	2.46647800	-3.71548600
H	-0.53766300	4.66616400	-2.31841300
H	-2.34401800	6.24643500	-2.23979300
C	-0.03141000	3.90429000	-4.88976700
H	0.85424600	3.89400000	-4.24089800
H	-0.15541500	4.93665200	-5.24112800
C	0.18478000	2.96411600	-6.07866500
H	-0.68059000	2.96821800	-6.75205700
H	1.06306400	3.26054100	-6.66140500
H	0.33933300	1.93184100	-5.74003400

UB3LYP-D3/BSII(SMD)//B3LYP-D3/BSI

HF=-2193.9372067

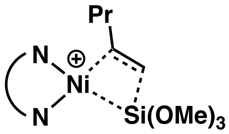

Triplet

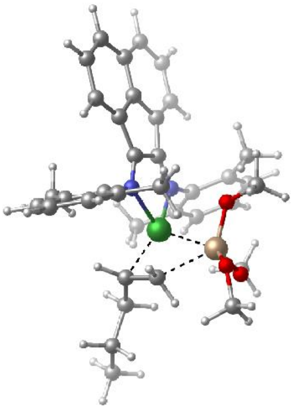

Zero-point correction= 0.717272 (Hartree/Particle)

Thermal correction to Energy= 0.762672

Thermal correction to Enthalpy= 0.763616

Thermal correction to Gibbs Free Energy= 0.635923

Sum of electronic and zero-point Energies= -2191.021177

Sum of electronic and thermal Energies= -2190.975778

Sum of electronic and thermal Enthalpies= -2190.974834

Sum of electronic and thermal Free Energies= -2191.102526

Ni	-2.85123500	3.01985500	-1.85361600
N	-1.28561300	2.31053800	-0.76880700
N	-3.81912400	1.45805800	-1.10370800
C	0.08403000	2.70463100	-0.87689700
C	0.87611300	2.04659300	-1.84313200
C	0.58219700	3.75598800	-0.08820800
C	2.19479600	2.47775000	-2.01243100
C	1.91447700	4.14245200	-0.28571300
C	2.71372700	3.51849200	-1.24094400
H	2.82009400	1.98646100	-2.75305800
H	2.32379300	4.94333400	0.32457400
H	3.74214400	3.83761300	-1.38140600
C	-5.18612100	1.10728800	-1.33525200
C	-5.52442000	0.64552200	-2.62289900
C	-6.15452700	1.29501400	-0.32750500
C	-6.86616200	0.36014200	-2.89065500
C	-7.48644100	1.00139800	-0.64667100
C	-7.84390700	0.53865900	-1.91209800
H	-7.14225300	-0.00873600	-3.87468200
H	-8.25048800	1.14586900	0.11248900
H	-8.88348600	0.31895400	-2.13613900

C	-0.26940000	4.44985300	0.94818000
H	-1.06744800	3.80486600	1.32101900
H	0.34433700	4.77145400	1.79550200
H	-0.74812700	5.34730400	0.53669400
C	0.32098300	0.89671000	-2.65168300
H	0.22078700	-0.00755300	-2.03774900
H	-0.67609500	1.12358300	-3.04803600
H	0.97912200	0.65520100	-3.49072700
C	-5.78943700	1.80694400	1.04535300
H	-5.03852400	2.59897500	0.98846800
H	-6.67335000	2.19933000	1.55653500
H	-5.37680100	1.00706800	1.67305900
C	-4.45510500	0.43049200	-3.66528400
H	-3.88291400	1.34747100	-3.84996900
H	-3.73054600	-0.32839500	-3.34535900
H	-4.88951300	0.10633500	-4.61479900
C	-3.05873900	0.78105600	-0.29508200
C	-1.64055100	1.23680100	-0.14279600
C	-2.11198800	4.88945700	-2.52464900
H	-2.71991700	5.71531000	-2.90394300
C	-1.90254400	3.83937300	-3.49908200
H	-0.91356900	3.38761100	-3.53784800
Si	-3.50706600	5.13829900	-0.76976300
O	-2.83017700	6.58110000	-0.37723900
O	-4.96234000	5.45596400	-1.47462800
O	-3.56209800	4.29316100	0.64575900
C	-2.91095200	7.80361300	-1.11191200
H	-3.88850300	7.91550600	-1.59294800
H	-2.76147000	8.62531000	-0.40717700
H	-2.12055100	7.84405500	-1.87182500
C	-5.91036500	4.50686200	-1.96238900
H	-5.44940900	3.79495200	-2.66349200
H	-6.36009400	3.93843400	-1.14302900
H	-6.68956200	5.05827900	-2.49306300
C	-3.67360400	4.91291800	1.94115900
H	-2.83593600	5.59325500	2.11606300
H	-4.61468300	5.46623800	2.02872700
H	-3.65791800	4.10990500	2.68210600
C	-3.96900100	-2.49620500	1.50157400
C	-2.72078200	-2.74158500	2.05392300
C	-1.64117000	-1.84262500	1.82747300
C	-1.92383400	-0.71731100	1.03278700
C	-3.20757900	-0.45581900	0.47173100
C	-4.23573800	-1.35423300	0.70276600

H	-0.03081900	-2.80575800	2.92648200
H	-4.77572800	-3.19911400	1.68494300
H	-2.56156800	-3.62804300	2.66167800
C	-0.30511200	-1.95759600	2.30524200
C	-0.94681900	0.25936100	0.69433400
H	-5.22687100	-1.19932300	0.29133400
C	0.34543600	0.12199600	1.16793000
C	0.64602800	-1.00167400	1.98144000
H	1.11613800	0.84605500	0.92698800
H	1.65819400	-1.11431800	2.35736300
H	-1.22526500	5.21767100	-1.98363000
C	-2.74411100	3.76239800	-4.74430800
H	-2.70969300	2.74685700	-5.15832200
H	-3.79789500	3.97281700	-4.50456000
C	-2.28023000	4.75705900	-5.83615000
H	-1.22879300	4.55354400	-6.07673900
H	-2.31502500	5.77547300	-5.42700900
C	-3.13828900	4.66831600	-7.10113800
H	-2.79399300	5.37831100	-7.86028700
H	-3.09516000	3.66364600	-7.53836800
H	-4.18920900	4.89419300	-6.88283000

UB3LYP-D3/BSII(SMD)//B3LYP-D3/BSI

HF=-2193.9294142

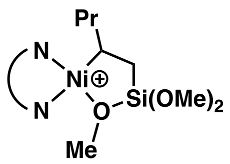

Singlet

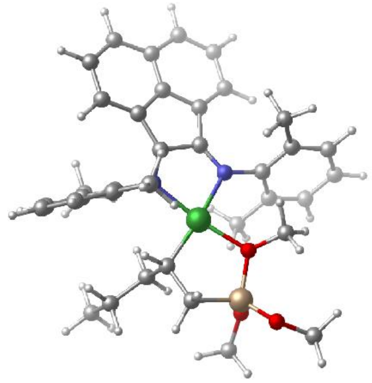

Zero-point correction= 0.720868 (Hartree/Particle)
Thermal correction to Energy= 0.765464
Thermal correction to Enthalpy= 0.766409
Thermal correction to Gibbs Free Energy= 0.642660
Sum of electronic and zero-point Energies= -2191.072122
Sum of electronic and thermal Energies= -2191.027526
Sum of electronic and thermal Enthalpies= -2191.026582
Sum of electronic and thermal Free Energies= -2191.150330

Ni	-2.61891100	3.36274200	-1.31607900
N	-1.09890100	2.17157200	-0.92729100
N	-3.69603400	1.83569800	-0.37117900
C	0.28131800	2.40628500	-1.23622700
C	0.91061500	1.68120900	-2.26521400
C	0.94288600	3.41990600	-0.51343300
C	2.23852600	2.01177800	-2.56889300
C	2.27006800	3.70226900	-0.84396800
C	2.91420500	3.00922500	-1.86997700
H	2.74262700	1.47222600	-3.36635500
H	2.79893800	4.47555000	-0.29381500
H	3.94278600	3.24766800	-2.12354600
C	-5.10964600	1.73537300	-0.20023500
C	-5.89868000	1.66535300	-1.36470800
C	-5.67157500	1.82092100	1.08869400
C	-7.28816800	1.65178400	-1.21010000
C	-7.06767300	1.81608200	1.19053000
C	-7.87210900	1.72906200	0.05491800
H	-7.91573700	1.58310200	-2.09451000
H	-7.52384100	1.89000200	2.17411000
H	-8.95342000	1.72551400	0.15509400

C	0.23000800	4.15782700	0.59200300
H	-0.08018600	3.47673700	1.39419400
H	0.87146100	4.92686800	1.03033500
H	-0.68345400	4.64011700	0.21854800
C	0.23676200	0.54949300	-3.00675800
H	0.48974300	-0.41638200	-2.55129000
H	-0.85298200	0.63096900	-3.00514800
H	0.56994700	0.51921000	-4.04837600
C	-4.80980700	1.94917200	2.32327500
H	-3.98039600	2.65029200	2.17093500
H	-5.40009500	2.30270800	3.17301500
H	-4.36375700	0.98874100	2.60857400
C	-5.25977600	1.61015800	-2.73118300
H	-4.79266900	2.56959900	-2.98356400
H	-4.48121900	0.84073800	-2.78559900
H	-6.00527300	1.39231400	-3.50108400
C	-2.90404700	0.87448400	-0.04404300
C	-1.44056900	1.09362800	-0.29767700
C	-2.18169500	5.88277700	-2.77501800
H	-1.84186300	6.33678700	-3.71563500
C	-1.60104200	4.47120900	-2.58211500
H	-0.58890000	4.53700100	-2.17387300
Si	-4.02287800	5.75514600	-2.65356100
O	-4.75219400	7.15752000	-2.22650200
O	-4.80384500	4.99336400	-3.88928600
O	-4.13349900	4.63522700	-1.36793800
C	-6.13510200	7.49064300	-2.38631800
H	-6.20116700	8.54767800	-2.65534200
H	-6.60884300	6.88680300	-3.16852300
H	-6.66687600	7.32962500	-1.44164100
C	-4.77141200	5.42909300	-5.25149400
H	-3.74181400	5.47310300	-5.62893600
H	-5.33228000	4.70358900	-5.84514300
H	-5.23686700	6.41551400	-5.36609300
C	-5.00528300	4.85762800	-0.23432700
H	-5.98897900	4.42821400	-0.43546900
H	-4.56128600	4.36561000	0.62928700
H	-5.08237200	5.93048500	-0.04362800
C	-3.85284100	-2.52425100	1.48495000
C	-2.56155500	-3.00068900	1.65393100
C	-1.44216000	-2.21579900	1.26000000
C	-1.72846800	-0.95689800	0.69844600
C	-3.05262300	-0.46064100	0.53975800
C	-4.12238200	-1.24614600	0.93137400

H	0.21290500	-3.51843500	1.79831100
H	-4.68988900	-3.14409500	1.79074900
H	-2.39891300	-3.98215200	2.09072100
C	-0.06626300	-2.55997600	1.36941000
C	-0.72033200	-0.06355900	0.23451900
H	-5.14503500	-0.90129700	0.82625100
C	0.61046000	-0.42633900	0.35503000
C	0.91707600	-1.68399100	0.93458600
H	1.40804800	0.22435200	0.01532400
H	1.96005600	-1.96855100	1.03259200
H	-1.85662700	6.54977800	-1.96675700
C	-1.59470400	3.63353700	-3.86525700
H	-2.57703900	3.68662100	-4.35314200
H	-1.43993800	2.57569900	-3.62671300
C	-0.49428400	4.04344900	-4.86482600
H	-0.60989000	5.10046700	-5.13830400
H	0.47925600	3.95619200	-4.36451400
C	-0.50923300	3.18097900	-6.13053400
H	-0.36109500	2.12150900	-5.88638800
H	-1.46771000	3.26659900	-6.65734900
H	0.28281600	3.47778100	-6.82650700

UB3LYP-D3/BSII(SMD)//B3LYP-D3/BSI

HF=-2193.9771189

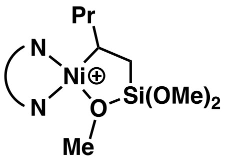

Triplet

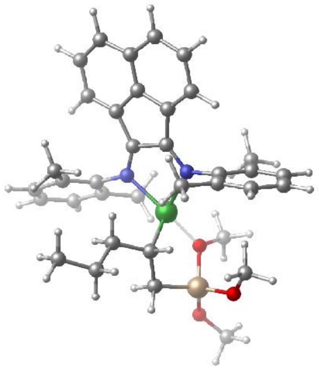

Zero-point correction= 0.717875 (Hartree/Particle)
Thermal correction to Energy= 0.763629
Thermal correction to Enthalpy= 0.764574
Thermal correction to Gibbs Free Energy= 0.635111
Sum of electronic and zero-point Energies= -2191.063223
Sum of electronic and thermal Energies= -2191.017469
Sum of electronic and thermal Enthalpies= -2191.016524
Sum of electronic and thermal Free Energies= -2191.145987

Ni	-2.51176300	3.76445900	0.20107300
N	-0.93063600	2.68726200	-0.49367700
N	-3.46212900	1.92937200	0.06374200
C	0.35008400	3.23475600	-0.80548900
C	0.64665300	3.60658400	-2.13043700
C	1.23757400	3.47126600	0.26043000
C	1.86583800	4.25367300	-2.36349100
C	2.44740000	4.11336300	-0.02182200
C	2.75782300	4.51123900	-1.32205600
H	2.11408900	4.55769100	-3.37677100
H	3.14827600	4.30106200	0.78713600
H	3.69654600	5.01776200	-1.52534900
C	-4.84099300	1.71077500	0.33276900
C	-5.73401500	1.26130800	-0.66669300
C	-5.29637000	2.07531800	1.62190600
C	-7.08811900	1.14402700	-0.32189900
C	-6.64929300	1.91873100	1.91957200
C	-7.54504900	1.45378400	0.95485000
H	-7.79034500	0.80837300	-1.08058800
H	-7.00533800	2.16951700	2.91498400
H	-8.59803000	1.34638500	1.19714500
C	0.90043300	2.99424700	1.65115400

H	1.01170100	1.90510200	1.73227200
H	1.55434200	3.45200900	2.39872600
H	-0.13838700	3.22203900	1.91140000
C	-0.30553600	3.30129600	-3.26148200
H	-0.32786300	2.22590900	-3.48000700
H	-1.32976800	3.60036200	-3.02140300
H	-0.00400100	3.81802700	-4.17649300
C	-4.34114500	2.62203600	2.65727900
H	-4.06013800	3.65995600	2.42529000
H	-4.80276900	2.63132800	3.64865100
H	-3.41649300	2.03749700	2.71881300
C	-5.32103900	0.93483700	-2.08339100
H	-4.35405500	1.36108700	-2.35275000
H	-5.25539600	-0.14871500	-2.23958900
H	-6.06073100	1.32388500	-2.78980900
C	-2.65009700	1.01974400	-0.36964700
C	-1.24815900	1.45677800	-0.71379800
C	-3.66917900	6.28075100	-0.63971900
H	-3.82480300	7.09663700	-1.36344800
C	-3.09072700	5.02490600	-1.30494400
H	-2.14934100	5.25883400	-1.82018500
Si	-2.56753100	6.86369500	0.73941000
O	-3.30631400	7.81458700	1.83776600
O	-1.14821600	7.57620200	0.32323300
O	-2.21125100	5.35999600	1.49655300
C	-2.80105100	8.98318100	2.49286700
H	-3.58645900	9.74346100	2.48607500
H	-2.55118400	8.73866200	3.53135600
H	-1.91120900	9.37224400	1.98884800
C	-0.13132100	7.01444300	-0.50872000
H	0.13131400	5.99774800	-0.19528700
H	-0.45192200	6.99175900	-1.55701100
H	0.75494100	7.64825400	-0.42645600
C	-1.63535200	5.32985900	2.81751900
H	-2.15012000	6.05275300	3.45626700
H	-1.77456600	4.32560900	3.22332900
H	-0.56796300	5.56467500	2.76907600
C	-3.34353000	-2.74924200	-0.71914500
C	-2.10271700	-3.10793100	-1.22363000
C	-1.09464200	-2.12487300	-1.42780800
C	-1.43969300	-0.80000700	-1.10208200
C	-2.71989400	-0.42488500	-0.60439300
C	-3.67492800	-1.40634900	-0.40319200
H	0.54514900	-3.33367100	-2.18671300

H	-4.09334500	-3.51837400	-0.56212900
H	-1.89293100	-4.14754900	-1.45943400
C	0.22744300	-2.33148600	-1.91242000
C	-0.52898300	0.28681300	-1.21293700
H	-4.65810300	-1.16703600	-0.01346700
C	0.75342600	0.05704700	-1.67915400
C	1.11160700	-1.26949600	-2.03235700
H	1.47359500	0.86244400	-1.77459100
H	2.11477800	-1.45555900	-2.40302200
H	-4.65690900	6.06181400	-0.20838000
C	-4.04026300	4.27202100	-2.22108800
H	-3.54369600	3.35665300	-2.57151400
H	-4.91920500	3.94074100	-1.64906800
C	-4.51629500	5.06421900	-3.45500800
H	-5.04793200	5.96625400	-3.12667300
H	-3.63887800	5.40855500	-4.02032800
C	-5.42774600	4.23022900	-4.36110900
H	-5.76266700	4.80441800	-5.23161600
H	-4.90794300	3.33676500	-4.72949200
H	-6.32050400	3.89574800	-3.81781100

UB3LYP-D3/BSII(SMD)//B3LYP-D3/BSI

HF=-2193.9736642

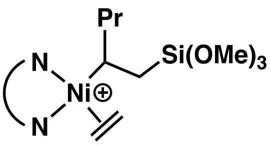

Singlet

Zero-point correction= 0.775141 (Hartree/Particle)

Thermal correction to Energy= 0.823697

Thermal correction to Enthalpy= 0.824642

Thermal correction to Gibbs Free Energy= 0.691212

Sum of electronic and zero-point Energies= -2269.611669

Sum of electronic and thermal Energies= -2269.563113

Sum of electronic and thermal Enthalpies= -2269.562169

Sum of electronic and thermal Free Energies= -2269.695598

Ni	0.34492400	1.08379400	-0.33801800
N	1.55460300	-0.52989900	-0.13043100
N	-1.14903200	-0.48338300	-0.38147400
C	2.97197900	-0.51991700	0.09373500
C	3.83361700	-0.73137200	-0.99581600
C	3.43979900	-0.26032100	1.39268500
C	5.21093500	-0.67426300	-0.75449600
C	4.82389400	-0.20909400	1.58670800
C	5.70447100	-0.41250900	0.52366900
H	5.89816100	-0.83464100	-1.58068100
H	5.21034900	-0.00712600	2.58198900
H	6.77627700	-0.36570400	0.69150200
C	-2.53233600	-0.42907600	-0.75146400
C	-2.83895800	-0.53822500	-2.12305000
C	-3.52308600	-0.25314200	0.22926900
C	-4.18254800	-0.45758700	-2.49929700
C	-4.85622900	-0.17848400	-0.19720400
C	-5.18696500	-0.27593200	-1.54719900
H	-4.43983500	-0.53744100	-3.55201000
H	-5.63902300	-0.05126800	0.54587600
H	-6.22594900	-0.21694500	-1.85744400
C	2.47107100	-0.07510600	2.53401100
H	1.99088200	-1.02472600	2.80542200
H	2.97706000	0.30919100	3.42363500
H	1.66979000	0.62021400	2.26176100
C	3.27924600	-1.03852400	-2.36508200
H	2.78569500	-2.01878200	-2.38350800
H	2.53089800	-0.29832500	-2.66778200
H	4.07195600	-1.04976700	-3.11763400
C	-3.18375500	-0.16054700	1.69927100
H	-2.95608700	0.87175700	1.99170700

H	-4.02800100	-0.48865700	2.31250900
H	-2.31833700	-0.77655100	1.96275800
C	-1.74573500	-0.73988500	-3.14350600
H	-0.98850400	0.04487800	-3.05381300
H	-1.24828800	-1.70971900	-3.01620400
H	-2.14998200	-0.70400200	-4.15857800
C	-0.57853400	-1.62691600	-0.23268100
C	0.91127700	-1.64380500	-0.01974700
C	1.30301000	2.38427100	-2.47065200
H	1.17599500	1.39273300	-2.92948000
C	1.74815100	2.25802000	-1.00138700
H	2.63441500	1.61803300	-0.95111400
Si	-0.19780900	3.39059400	-2.96645000
O	0.03549700	3.73137600	-4.56189000
O	-1.53370000	2.46078500	-2.66895500
O	-0.44883900	4.78347200	-2.10283900
C	-0.95972300	4.28570600	-5.42180000
H	-0.47486300	4.56913300	-6.35953900
H	-1.74186800	3.54741000	-5.63850500
H	-1.42445800	5.17848500	-4.98287300
C	-2.87329200	2.95416700	-2.60436900
H	-3.30744100	3.01591900	-3.60942800
H	-3.46470200	2.24848300	-2.01620500
H	-2.91030800	3.94431000	-2.13662300
C	0.23421000	6.01847500	-2.31993500
H	0.52072100	6.14137400	-3.37095100
H	-0.43687600	6.83391300	-2.03589900
H	1.13523800	6.06844900	-1.69711400
C	-2.31392900	-5.05695800	-0.23320000
C	-1.18902300	-5.81736800	0.04959000
C	0.08100000	-5.19865000	0.21996000
C	0.11466100	-3.79806100	0.08552300
C	-1.03917200	-3.01734600	-0.20418400
C	-2.26214900	-3.64509300	-0.36419400
H	1.35356200	-6.91366100	0.62716300
H	-3.27026100	-5.55474600	-0.35981500
H	-1.27436300	-6.89658300	0.14235000
C	1.32047600	-5.83385200	0.51094000
C	1.30732300	-3.03164700	0.22253600
H	-3.16209500	-3.08339700	-0.58732000
C	2.49979100	-3.67700200	0.50329300
C	2.48211300	-5.08826900	0.64683600
H	3.42887100	-3.12953500	0.61358600
H	3.41431800	-5.59789800	0.86965100

C	-1.21215100	2.65630200	0.34028400
H	-2.12298200	2.13097500	0.07182500
H	-1.00646000	3.57491200	-0.19744700
C	-0.45334500	2.27205400	1.40529000
H	0.37197000	2.87978800	1.75762100
H	-0.74213700	1.43651600	2.03812200
H	2.14509000	2.83117300	-3.02672100
C	2.06113600	3.57194400	-0.30058700
H	2.60292200	4.21550300	-1.01506500
H	1.14553500	4.12587000	-0.06289900
C	2.93746900	3.41094900	0.94814600
H	3.89060100	2.94986300	0.65679900
H	2.46932300	2.69974900	1.64012100
C	3.19478500	4.73879900	1.66534500
H	3.83629700	4.60298900	2.54259800
H	3.68782600	5.45737600	0.99960900
H	2.25573600	5.19450900	2.00455900

UB3LYP-D3/BSII(SMD)//B3LYP-D3/BSI

HF=-2272.5949902

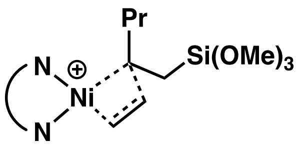

Singlet

Zero-point correction= 0.775273 (Hartree/Particle)

Thermal correction to Energy= 0.823061

Thermal correction to Enthalpy= 0.824005

Thermal correction to Gibbs Free Energy= 0.691488

Sum of electronic and zero-point Energies= -2269.590815

Sum of electronic and thermal Energies= -2269.543027

Sum of electronic and thermal Enthalpies= -2269.542083

Sum of electronic and thermal Free Energies= -2269.674600

Ni	-0.09790400	1.00545500	-0.04153600
N	1.40036700	-0.59275500	-0.10777100
N	-1.28755000	-0.58925700	-0.19175600
C	2.81699800	-0.48230700	0.00074300
C	3.63576300	-0.64345400	-1.13372900
C	3.33483000	-0.10617300	1.25768100
C	5.00851800	-0.41560900	-0.97773600
C	4.71277600	0.10248300	1.36446300
C	5.54522600	-0.04570800	0.25494900
H	5.66053700	-0.52899200	-1.83973100
H	5.13197500	0.38367300	2.32665600
H	6.61280100	0.12831900	0.35049500
C	-2.72160300	-0.55356100	-0.24231400
C	-3.34443600	-0.38945800	-1.49044100
C	-3.43719500	-0.64663700	0.96200100
C	-4.74220300	-0.32357600	-1.51019600
C	-4.83328100	-0.57455300	0.89379300
C	-5.48241900	-0.41462500	-0.33049700
H	-5.25079100	-0.20094700	-2.46277700
H	-5.41129900	-0.64401000	1.81132500
H	-6.56635800	-0.35891900	-0.36546400
C	2.41699100	0.02730500	2.44828500
H	1.97555600	-0.93973800	2.72017400
H	2.95433400	0.41110400	3.31947400
H	1.57979800	0.70558600	2.23671600
C	3.07610000	-1.07859500	-2.46770600
H	2.95605500	-2.16885100	-2.50768200
H	2.09235500	-0.64321800	-2.66779900
H	3.74543900	-0.79050500	-3.28293500
C	-2.71211700	-0.84659100	2.27065200
H	-1.88446600	-0.13525300	2.38374400

H	-3.38973300	-0.72630100	3.12006400
H	-2.27414600	-1.85146800	2.33197600
C	-2.52017300	-0.30815000	-2.75170600
H	-1.76896100	0.48806400	-2.69138000
H	-1.98363800	-1.24804300	-2.93604600
H	-3.15471000	-0.11493900	-3.62137700
C	-0.68543700	-1.72698600	-0.27775800
C	0.81523900	-1.73349200	-0.20399600
C	1.63186100	2.38527700	-1.81898300
H	1.48806800	1.34077900	-2.12166800
C	1.51704700	2.53031400	-0.30225600
H	2.04698200	1.72452300	0.20390100
Si	0.53636600	3.39503200	-2.94254400
O	1.21372500	3.38303400	-4.44351500
O	-0.96226200	2.67587000	-2.96588000
O	0.38327100	4.93630000	-2.36258700
C	0.49511000	3.37686200	-5.67542100
H	1.19106100	3.09865000	-6.47111900
H	-0.32790500	2.65189200	-5.65283300
H	0.08888600	4.37196900	-5.89968800
C	-2.18083400	3.37896400	-3.19714400
H	-2.27884300	3.67060200	-4.25070300
H	-3.00942800	2.71000000	-2.94565000
H	-2.24590400	4.27728100	-2.57136200
C	0.32164800	6.12560500	-3.15083800
H	0.99793900	6.06659800	-4.01119200
H	-0.70105300	6.30039900	-3.50883100
H	0.62102800	6.96650600	-2.51971200
C	-2.37999600	-5.16320800	-0.58190700
C	-1.22349700	-5.92727900	-0.53686600
C	0.05149800	-5.30672700	-0.41752000
C	0.05689400	-3.90057700	-0.35566500
C	-1.13067400	-3.11546800	-0.39636800
C	-2.35771700	-3.74631400	-0.50963700
H	1.37679900	-7.02983800	-0.39237300
H	-3.33984200	-5.66204800	-0.67282700
H	-1.28772600	-7.01056600	-0.59117800
C	1.32092800	-5.94562100	-0.34868900
C	1.24877300	-3.13260300	-0.23516200
H	-3.28365300	-3.18358500	-0.54288900
C	2.46932800	-3.78062200	-0.16715600
C	2.48266000	-5.19819800	-0.22463400
H	3.39822700	-3.22893500	-0.07307600
H	3.43811100	-5.71069600	-0.17116600

C	-1.61928900	2.22794500	0.24480100
H	-2.09529200	1.86281700	1.15405200
H	-2.23058900	2.17382400	-0.65291000
C	-0.59766400	3.18675800	0.32997000
H	-0.46631000	3.90261300	-0.47004700
H	-0.25733200	3.50774800	1.30677100
H	2.66926800	2.62381900	-2.10385000
C	2.07990600	3.82761300	0.27530100
H	3.02291200	4.00993200	-0.26517700
H	1.45467100	4.69787600	0.04818700
C	2.40882800	3.75550700	1.77292600
H	3.12675900	2.93978000	1.93573400
H	1.51307300	3.48674300	2.34983000
C	2.98632000	5.06888000	2.30864700
H	3.23256100	4.99132600	3.37291200
H	3.90219100	5.34392000	1.77202600
H	2.27166900	5.89191600	2.18879900

UB3LYP-D3/BSII(SMD)//B3LYP-D3/BSI

HF=-2272.5734439

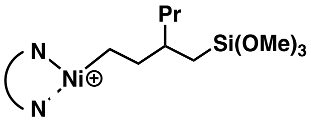

Singlet

Zero-point correction= 0.776026 (Hartree/Particle)
Thermal correction to Energy= 0.824269
Thermal correction to Enthalpy= 0.825213
Thermal correction to Gibbs Free Energy= 0.689601
Sum of electronic and zero-point Energies= -2269.620097
Sum of electronic and thermal Energies= -2269.571854
Sum of electronic and thermal Enthalpies= -2269.570910
Sum of electronic and thermal Free Energies= -2269.706522

Ni	-0.09902200	0.69515900	0.16910300
N	1.36668400	-0.73601300	0.13244800
N	-1.28726700	-0.76044800	-0.04412500
C	2.76416900	-0.47566900	0.17074100
C	3.52878500	-0.53926000	-1.01203000
C	3.29410400	0.00085000	1.38743400
C	4.86498400	-0.12689200	-0.93943100
C	4.63474100	0.39283000	1.40990500
C	5.41661300	0.33261700	0.25566600
H	5.47280100	-0.15727900	-1.83961600
H	5.06550300	0.75065800	2.34096700
H	6.45441700	0.65055500	0.28621800
C	-2.71374400	-0.67407800	-0.15008800
C	-3.28346600	-0.49190600	-1.42141700
C	-3.46967100	-0.72322300	1.03146600
C	-4.67779900	-0.39435400	-1.48887000
C	-4.85969600	-0.61900600	0.91409300
C	-5.46032200	-0.46148000	-0.33524600
H	-5.14994000	-0.25902100	-2.45815600
H	-5.47073100	-0.65748600	1.81170500
H	-6.54082300	-0.38225900	-0.40964900
C	2.43731700	0.04481800	2.62934100
H	2.14282000	-0.96283100	2.94804600
H	2.96881300	0.51973900	3.45829200
H	1.50620500	0.60220400	2.46031900
C	2.93030700	-0.99254600	-2.32272700
H	2.78929800	-2.07969800	-2.35285000
H	1.95039700	-0.53476100	-2.49782300
H	3.58033000	-0.72060400	-3.15846200
C	-2.78421500	-0.88262900	2.36550500
H	-1.98793800	-0.13634800	2.48588800
H	-3.49251200	-0.76948300	3.19032600

H	-2.31209000	-1.86902500	2.46069700
C	-2.41742400	-0.38730900	-2.65322500
H	-1.77646200	0.50307500	-2.61495900
H	-1.76313700	-1.26037700	-2.76550400
H	-3.03178600	-0.31456900	-3.55473000
C	-0.67708200	-1.89635000	-0.15514300
C	0.82542700	-1.88633000	-0.06871100
C	1.17832200	2.35831100	-1.64352400
H	0.69098200	1.39124000	-1.84900000
C	1.04857100	2.76478100	-0.14925400
H	1.35689500	1.88598600	0.48627300
Si	0.39664800	3.47810200	-2.91820100
O	1.20635200	3.25538000	-4.33651300
O	-1.17760500	2.97478400	-3.07476400
O	0.43728800	5.05417900	-2.42502600
C	0.62176000	3.35718500	-5.63401200
H	1.28044500	2.84856000	-6.34314600
H	-0.36748500	2.88391100	-5.66331600
H	0.52463500	4.40712000	-5.93902800
C	-2.31068900	3.82438400	-3.22902100
H	-2.48541400	4.05006100	-4.28866200
H	-3.18645900	3.30023000	-2.83393000
H	-2.18364300	4.76511800	-2.68026000
C	0.58390600	6.18394600	-3.28540300
H	1.36122700	6.01163300	-4.03903600
H	-0.36173000	6.41586700	-3.79264600
H	0.86950100	7.04152600	-2.67056000
C	-2.32241900	-5.32052100	-0.69500200
C	-1.15297800	-6.06702800	-0.71618100
C	0.11437000	-5.43806700	-0.56050000
C	0.09850500	-4.04164900	-0.39089200
C	-1.10306500	-3.27496500	-0.36507400
C	-2.32175900	-3.91294300	-0.51868200
H	1.46837900	-7.13489000	-0.68281000
H	-3.27463000	-5.82734200	-0.81617000
H	-1.20177300	-7.14387500	-0.85246400
C	1.39543000	-6.05837200	-0.55537500
C	1.28062600	-3.26594200	-0.22521400
H	-3.25480200	-3.36051000	-0.50579300
C	2.51252700	-3.89454300	-0.22392700
C	2.54751200	-5.30324000	-0.38990700
H	3.43249600	-3.33224100	-0.10211100
H	3.51126400	-5.80267600	-0.38902100
C	-1.38374300	2.08866100	0.13315300

H	-2.09574200	1.97979900	0.95716200
H	-1.89924900	2.05253400	-0.82959600
C	-0.38516000	3.23088000	0.27207600
H	-0.64890500	4.10872400	-0.33091600
H	-0.34073400	3.55444500	1.31533500
H	2.23565400	2.20143500	-1.88284200
C	2.12631300	3.80438700	0.23267900
H	3.07845700	3.48059800	-0.20821600
H	1.85670400	4.75151700	-0.25211300
C	2.33854500	4.00778700	1.73740400
H	2.53277500	3.03248500	2.20775200
H	1.42826600	4.40356500	2.20432000
C	3.50805400	4.95455800	2.02821400
H	3.64689900	5.09592700	3.10530600
H	4.44483000	4.55989000	1.61636600
H	3.33705100	5.94054600	1.58036000

UB3LYP-D3/BSII(SMD)//B3LYP-D3/BSI

HF=-2272.6053804
